# Organophotoredox Dioxygenation of Alkenes via ROH···F-Activated *N*‑Alkoxyphthalimides

**DOI:** 10.1021/acs.orglett.5c03734

**Published:** 2025-09-30

**Authors:** Pau Sarró, Albert Gallego-Gamo, Elies Molins, Roser Pleixats, Carolina Gimbert-Suriñach, Adelina Vallribera, Albert Granados

**Affiliations:** † Department of Chemistry and Centro de Innovación en Química Avanzada (ORFEO−CINQA), 16719 Universitat Autònoma de Barcelona, Cerdanyola del Vallès, 08193 Barcelona, Spain; ‡ 54449 Institut de Ciència de Materials de Barcelona (ICMAB-CSIC), Campus UAB, 08193 Bellaterra, Spain

## Abstract

The generation and controlled reactivity of alkoxyl radicals remain challenging due to their high energy and tendency for β-scission or hydrogen atom transfer events. Herein, we report a photoredox-catalyzed dioxygenation of alkenes enabled by the activation of fluorinated *N*-alkoxyphthalimides through hydrogen-bonded aggregates with ROH···F. This transformation proceeds under mild, metal-free conditions using an organophotocatalyst and a simple alcohol, affording a diverse range of dialkoxylated products with high functional group tolerance and good scalability. Mechanistic studies—including UV–Vis spectroscopy, NMR, cyclic voltammetry, and Stern–Volmer quenching—reveal a radical/polar crossover pathway.

## Graphical abstract

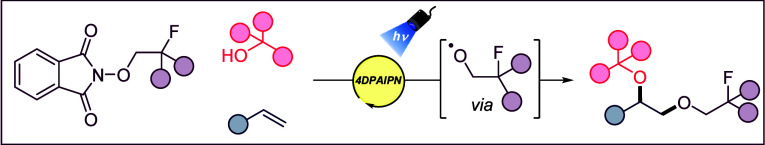

Net-neutral radical/polar crossover (RPC) reactions within photoredox catalysis involve both single-electron oxidation and reduction events between a photocatalyst and substrates and/or intermediates, without the need for external oxidants or reductants.^1^ Avoiding additional reagents, net-neutral RPC transformations offer streamlined optimization, while allowing the rapid incorporation of diverse functional groups, making it a powerful tool for alkene difunctionalization.^2^ The mild reaction conditions also enable access to complex structures that are not feasible through conventional two-electron pathways.

Alkoxy radicals (RO·) are distinguished by their pronounced electrophilic nature and typically display a well-defined reactivity profile.^3^ Predominantly, their reactivity occurs through intramolecular 1,5-hydrogen atom transfer (1,5-HAT), β-scission processes, and intramolecular addition to π systems leading to tetrahydrofuran derivatives.^4^ The generation of these open-shell species can be achieved through transition metal catalysis,^5^ photochemical methods,^6^ and electrochemical protocols.^7^ Despite significant advancements in the generation and utilization of oxygen-centered radicals,^8^ their application in dialkoxylation reactions, particularly in three component processes, remains limited. For example, Dagousset and co-workers^9^ designed a robust Ir-based photoredox protocol for the dialkoxylation of alkenes utilizing *N*-alkoxypyridinium salts as radical precursors.

Another interesting example is the electrochemical dimethoxylation protocol of olefins presented in 2019 by Zhang and Xu.^10^ The single-electron reduction of *N*-alkoxyphthalimides for the generation of alkoxy radicals is well-established, with these species known to readily undergo intermolecular HAT, intramolecular 1,5-HAT, 1,2-HAT or β-scission processes to yield highly valuable C-centered radicals.^11–13^ Of particular interest is *N*-trifluoroethoxyphthalimide (**2a**, Figure 1A), which, upon reduction, forms a radical anion species that undergoes N–O bond cleavage yielding a phthalimide anion and trifluoroethoxyl radical (·OCH_2_CF_3_). This electrophilic radical has demonstrated full utility in intermolecular HAT and 1,2-HAT pathways (Figure 1A).^12,13^

**Figure 1:**
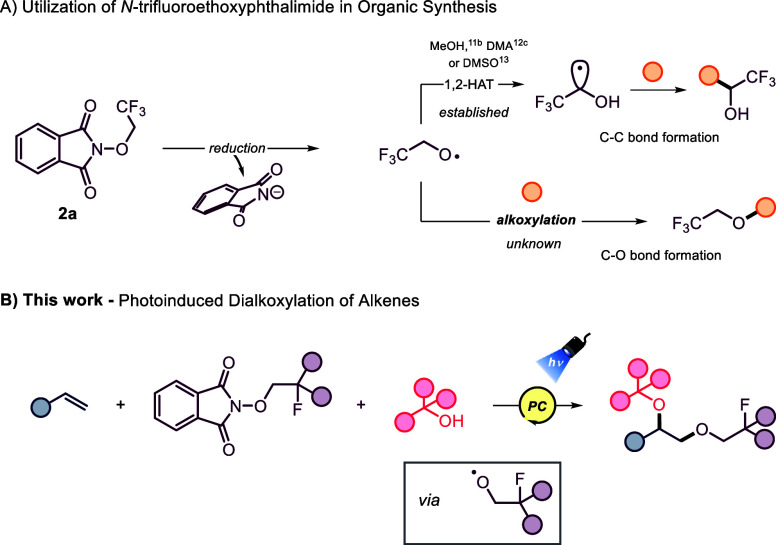
(A) Generation and utilization of trifluoroethoxyl radical from *N*-trifluoroethoxy­phthalimide. (B) This work.

In this work, we report the utilization of *N*-trifluoroethoxyphthalimide and alcohols for the dialkoxylation of alkenes under sustainable photochemical conditions (Figure 1B). This new chemical space will expedite new opportunities within organofluorine chemistry environments, which is crucial in medicinal chemistry due to the unique properties provided, such as metabolic stability, binding affinity, selectivity, membrane permeability and p*K*_a_ modulation.^14^

At the outset of our studies, the critical parameters of this photochemical transformation were explored, resulting in a suitable set of reaction conditions to explore the scope and limitations of the process (see Section 3.2. in the Supporting Information (SI) for details). We hypothesized that alkoxy radicals from reagent **2a** could be generated and utilized without highly polar solvents (DMA, DMSO), which typically promote intramolecular 1,2-HAT via hydrogen bonding (Figure 1A). Using DCM with 5 equiv of MeOH, product **3** was obtained in 77% yield (NMR yield) from 4-methoxystyrene with 4DPAIPN under 427 nm irradiation. This method directs O-centered radicals generated from alkoxyphthalimides toward intermolecular addition to π-systems, overcoming their typical 1,2-HAT behavior. Next, we elected to explore the amenability of these optimal reaction conditions to other substrates. Initial focus was aimed at exploring styrene diversity in combination with reagent **2a** and MeOH as the nucleophile (Table 1). *Para*-substituted styrenes bearing both electron-donating and electron-withdrawing groups generally afforded high yields, particularly alkyl (**5**) and halogenated derivatives (**6–8**, 70–92%). Strongly electron-withdrawing groups gave reduced efficiency (**9–10**), while a TMS-alkyne substituent was well tolerated (**11**). Additional examples, including 3,5-dimethoxystyrene (**12**, 30%), *ortho*-methoxy styrene (**13**, 47%), and a benzothiophene derivative (**14**), highlighted the influence of sterics and functional group compatibility, whereas pyridine styrenes failed. Endocyclic alkenes (**15–16**) and α-substituted styrenes (**17–18**) performed well, with α-cyclopropylstyrene yielding 77% without ring-opening. Late-stage functionalization of bioactive styrene derivatives (**19–23**) was achieved in moderate yields (34–58%), underscoring the method’s utility in drug discovery. Expanding the scope to alcohols, the reaction accommodated primary, secondary, and tertiary substrates. Various primary alcohols, including methanol, benzyl derivatives, and those bearing epoxide, alkyne, or TMS groups (**24–27**), provided products in moderate yields, while bicyclo[1.1.1]­pentyl and diol derivatives also proved compatible (**28–29**). Natural alcohols delivered more complex products (**30–31**) with lower efficiency. Secondary acyclic and cyclic alcohols (**32–36**), including cyclopropanol and (+)-menthol, reacted smoothly, while tertiary alcohols (**37–38**) were incorporated successfully. Even phenol and water served as nucleophiles, affording products **39–40** in modest yields. Overall, remarkable functional-group tolerance was observed during the selective alkene difunctionalization process, allowing the preservation of sensitive functionalities. A few styrenes delivered the product in lower yields primarily due to a less favorable polarity match between the radical acceptor and the electrophilic oxygen-centered radical, which likely contributes to partial loss of the latter. It is also important to consider that intramolecular 1,2-HAT may occur. For some alcohols, the diminished reactivity observed with more structurally complex substrates may stem from increased conformational or electronic constraints. This observation is particularly significant given the dual role of the alcohol—serving both as an activator of the *N*-alkoxyphthalimide and as the nucleophile in the subsequent trapping event.

**Table 1:**
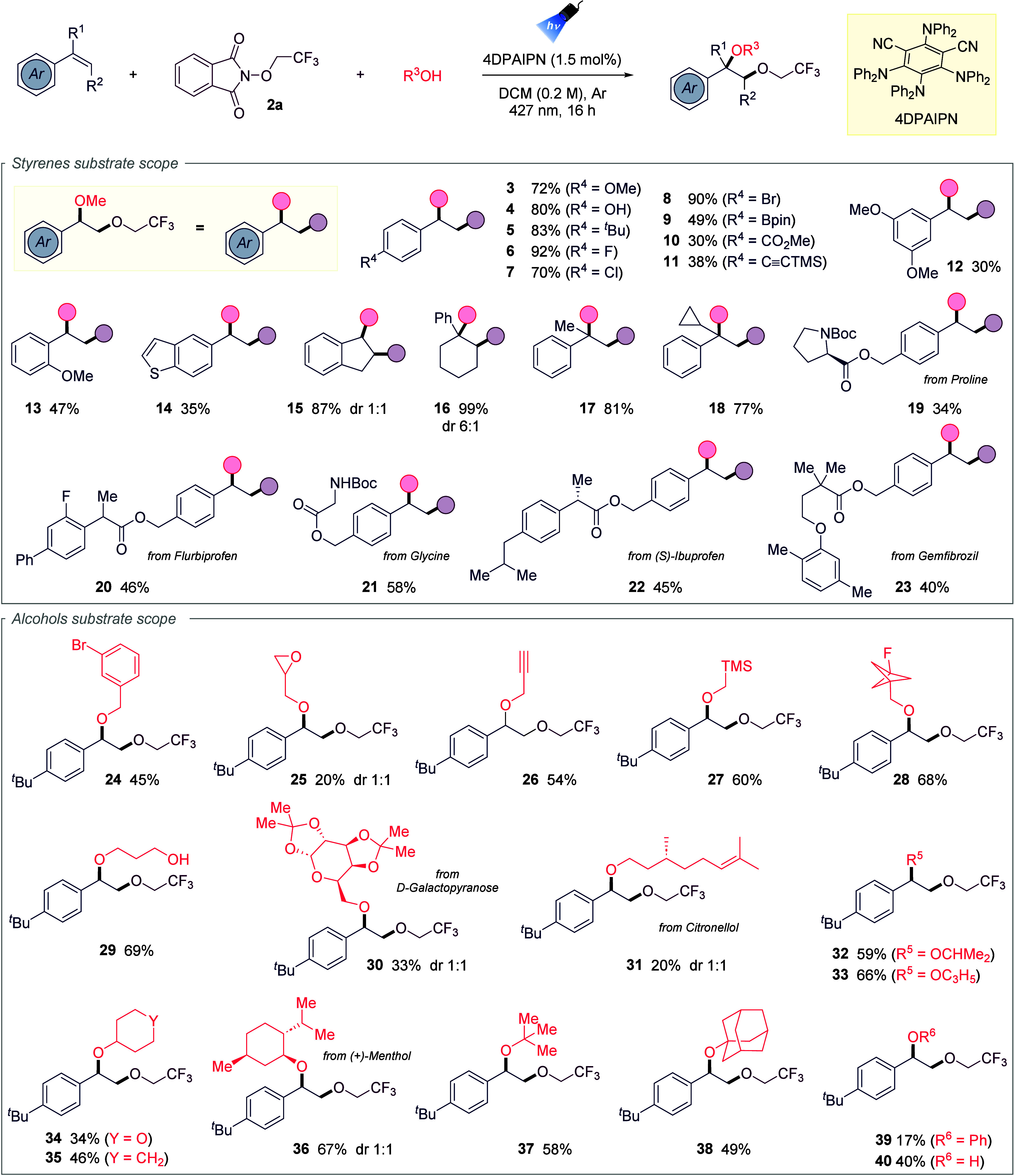
Scope of Styrenes and Alcohols

a Reaction conditions: Corresponding alkene (0.3 mmol, 1 equiv), **2a** (0.6 mmol, 2 equiv), desired alcohol (1.5 mmol, 5 equiv), 4DPAIPN (1.5 mol %) in 1.5 mL of DCM (*c* = 0.2 M) under 427 nm Kessil lamp irradiation at rt for 16 h. Referred yields after the purification process by silica gel column chromatography.

**Figure 2:**
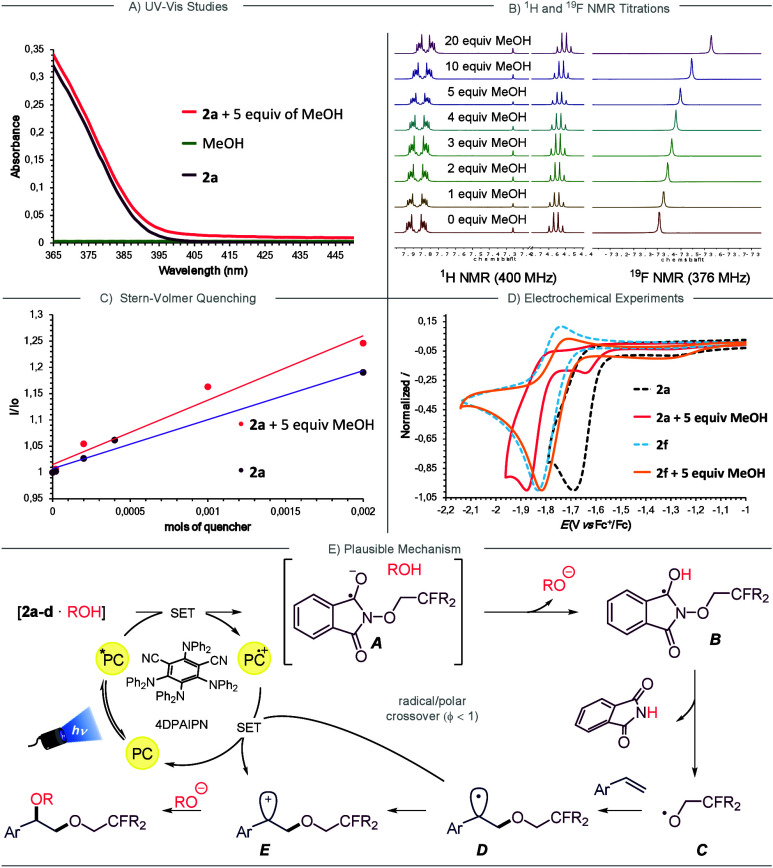
(A) UV–vis studies of the individuals **2a**, MeOH and the mixture **2a**+MeOH. (B) ^1^H and ^19^F NMR titrations of **2a** and MeOH mixtures. (C) Stern–Volmer luminescence quenching studies of **2a** and **2a**+MeOH mixture. (D) Cyclic voltammetry studies of **2a**, **2f** and mixtures **2a**+MeOH and **2f**+MeOH. (E) Proposed mechanism.

We next examined alternative redox-active *N*-alkoxyphthalimides as alkoxy radical progenitors, focusing on fluorinated motifs of medicinal relevance^15,16^ (CF_2_H, CF_2_CF_3_, and CFH_2_ analogues; Table 2). The corresponding species (**2b–d**) furnished derivatives **41–43** in good yields (58–73%), underscoring the beneficial effect of fluorination. In contrast, nonfluorinated analogues such as nitrile (**2e**), ester (**2f**), and ethyl (**2g**) failed to deliver products **44–46** in productive yields even using a more highly reducing photocatalyst (see Section 6.7 in the SI for details) ranging from full consumption (**2e**) to complete recovery (**2g**). Similar unreactivity of nonfluorinated phthalimides has been noted previously.^9^ These results highlight the key role of fluorination in enabling this transformation and prompted further mechanistic investigation. First, we analyzed the reaction components using UV–vis spectroscopy, both individually and in mixtures. Notably, when 5 equiv of MeOH were added to a DCM solution of **2a**, a bathochromic shift was observed (Figure 2A), suggesting that both reagents form a new molecular aggregate (**2a**·MeOH). Additionally, the formation of this aggregate was supported by NMR titrations, including both ^1^H and ^19^F NMR analysis (Figure 2B and Section 6.4 in the SI).

**Table 2:**
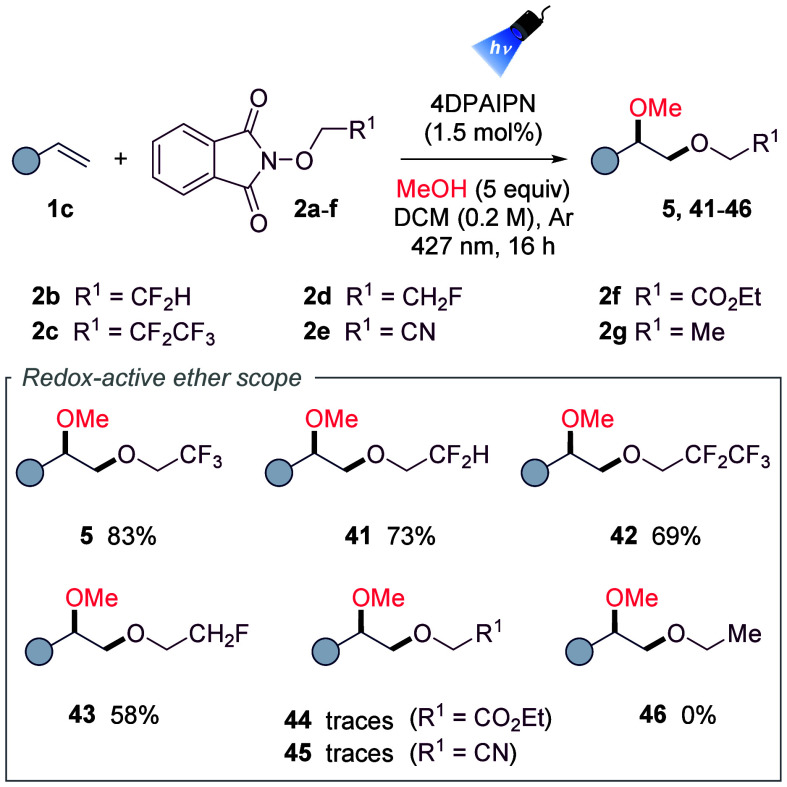
Redox-Active Ether Species Scope Evaluation

a Reaction conditions: 4-*tert*-butylstyrene (**1c**) (0.3 mmol, 1 equiv), **2a**–**g** (0.6 mmol, 2 equiv), MeOH (1.5 mmol, 5 equiv), 4DPAIPN (1.5 mol %) in 1.5 mL of DCM (*c* = 0.2 M) under 427 nm Kessil lamp irradiation at rt for 16 h. Referred yields after the purification process by silica gel column chromatography.

Subsequently, Stern–Volmer quenching studies showed that the **2a**·MeOH aggregate quenched the photocatalyst’s excited state more effectively than reagent **2a** alone, highlighting this interaction as key to the method’s success (Figure 2C and Section 6.5 in the SI). Finally, cyclic voltammetry studies further confirmed the formation of this new species, revealing a new irreversible reduction peak at *E*_p/2_ = −1.92 V vs Fc^+^/Fc (Figure 2D, pink trace). Further electrochemical experiments with **2b**·MeOH, **2c**·MeOH and **2d**·MeOH also revealed the appearance of a new, irreversible reduction peaks (see Figures S8–S10 in the SI). Collectively, these results suggest that the reaction mechanism is initiated by a SET event between the newly formed **2a**–**d**·MeOH aggregates (*E*_p/2_ between −1.87 and −1.95 V vs Fc^+^/Fc) and 4DPAIPN (*E* ^PC·+/PC*^ = −1.96 V vs Fc^+^/Fc), with the fluorine substituents playing a role in aggregate formation. This hypothesis is further supported by electrochemical studies of nonfluorinated phthalimides **2e**–**g** (see Section 6.2. in the SI for details). Overall, these results highlight the essential role of fluorine atoms in promoting the formation of reactive aggregates capable of undergoing single-electron reduction, likely through hydrogen bonding interactions between fluorine and the OH group (F···HOR). To complete the mechanistic investigation, we confirmed that reagent **2a** is capable of undergoing single-electron reduction even in the absence of methanol. Under standard irradiation conditions, 28% of **2a** was consumed after 16 h, indicating that **2a** can indeed be reduced by the excited state of 4DPAIPN, albeit at a significantly slower rate compared to conditions when methanol is present. This experiment is in good agreement with the observed Stern–Volmer studies. Altogether, these findings confirm that fluorinated **2a**–**d**·MeOH aggregates are both thermodynamically and kinetically favorable for SET, whereas in the absence or less effective aggregation, the reduction event remains thermodynamically feasible but proceed at much slower rate (see Figures S30 and S31). Lastly, initiation via a charge-transfer complex between **2a** and MeOH was ruled out, as no reaction occurred under 390 nm irradiation in the absence of the photocatalyst. Radical trapping experiments using 2,2,6,6-tetramethylpiperidine-1-oxyl (TEMPO) provided further evidence for the proposed mechanism. High-resolution mass spectrometry (HRMS) analysis confirmed the formation of a benzylic radical intermediate, as indicated by the structure of compound TEMPO-derived **47** (see the Section 6.1 in the SI).

Based on the mechanistic investigations described above, a plausible mechanistic rationale is depicted in Figure 2E. Upon photoirradiation with visible blue light, the photocatalyst is excited. Subsequently, the fluorinated aggregate formed between the alcohol and the fluorinated *N*-alkoxyphthalimide undergoes SET with PC*, resulting in the formation of an anionic radical species ***A*** and the alcohol, likely within a solvent cage. Thereafter, ***A*** undergoes protonation by the alcohol and subsequently undergoes N–O bond cleavage to afford phthalimide and the key O-centered radical ***C***, which engages in intermolecular radical addition to the olefin. The resulting C*sp* ^2^-hybridized radical ***D*** is then oxidized to carbocation ***E*** by PC^·+^, restoring the photocatalytic cycle. This net-neutral radical/polar crossover process is supported by the measured photochemical quantum yield, which resulted to be less than 1 (see the Supporting Information, Φ = 0.50). Finally, the carbocation is trapped by the alkoxide to furnish the dioxygenated product.

In summary, we present an efficient photochemical alkene difunctionalization method to access dialkoxylated compounds. The use of fluorinated *N*-alkoxyphthalimides as suitable O-centered radical precursors is demonstrated for the first time, contrasting with their conventional role as effective HAT mediators. This organophotoredox method is amenable to a broad range of styrenes and alcohols, with late-stage functionalization also being feasible. The electronic properties of the fluorine atoms and the hybridization of the carbon to which they are attached directly influence the ability of the *N*-alkoxyphthalimide scaffold to generate a new aggregate that undergo single-electron reduction by the excited-state photocatalyst. Mechanistic investigations support a net-neutral radical-polar crossover pathway. We envision that this new activation mode of fluorinated *N*-alkoxyphthalimides will open further opportunities in synthetic organic chemistry.

## Supplementary Material



## Data Availability

The data underlying this study are available in the published article and its Supporting Information.

## Notes

Preparation of starting materials, general procedures, mechanistic studies and NMR spectra (PDF)
