# Supplementary material for: Organophotoredox Dioxygenation of Alkenes via ROH···F-Activated N‑Alkoxyphthalimides
Source: Org Lett. 2025 Sep 30;27(40):11372–7. doi: 10.1021/acs.orglett.5c03734 (PMC12519466; doi:10.1021/acs.orglett.5c03734)
Supplement: Supplementary file 1 [file ol5c03734_si_001.pdf]

Supporting Information

Organophotoredox Dioxygenation of Alkenes via ROH···F-Activated *N*-Alkoxyphthalimides

Pau Sarró,[a,‡] Albert Gallego-Gamo,[a,‡] Elies Molins,[b] Roser Pleixats,[a] Carolina-Gimbert-Suriñach,[a,*] Adelina Vallribera[a,*] Albert Granados[a,*]

[a]*Departament de Química and Centro de Innovación en Química Avanzada (ORFEO-CINQA), Universitat Autònoma de Barcelona, Cerdanyola del Vallès, 08193 Barcelona, Spain*

[b]*Institut de Ciència de Materials de Barcelona (ICMAB-CSIC), Campus UAB, 08193 Bellaterra, Spain*

[‡]*These authors contributed equally*

*To whom correspondence should be addressed.

adelina.vallribera@uab.es carolina.gimbert@uab.es albert.granados@uab.es

TABLE OF CONTENT

1. General Considerations 3

2. List of Used Styrenes and Redox Active Phthalimides 4

3. Difunctionalization of Alkenes: Reaction Workflow and Compound Characterization 6

4. Large-scale synthesis of **3** 29

5. Data of X-Ray Structure of Compound **4** 30

6. Mechanistic Investigation 32

7. NMR Spectra 56

1. General Considerations

1.1 General

All chemical transformations requiring inert atmosphere were done using Schlenk line techniques with a 4- or 5-port dual-bank manifold. For purple light irradiation, a Kessil PR160-purple LED lamp (40 W High Luminous DEX 2100 LED, λ_{max} = 427 nm) was placed 4 cm away from the reaction vials. Photoinduced reactions were performed using 4 or 8 mL Chemglass vials (15-425 Green Open Top Cap, TFE Septa). Reactions were monitored by TLC or NMR. TLC analysis was performed using hexanes or pentane with EtOAc or Et_2O as the eluents and visualized using UV light and Vanillin or $KMnO_4$ solutions. The cyclic voltammetry experiments were performed with a BioLogic® SP-50 Single Channel Potentiostat, in one-compartment three-electrode set-up using glassy carbon disk as working electrode (ø = 3 mm), platinum wire as auxiliary electrode and SCE as reference electrode. Experiments were performed at room temperature, using the appropriate solvent, degassing with Ar, using $TBAPF_6$ as supporting electrolyte (0.1 M). All the experiments were referred to ferrocene as internal standard. Fluorescence measurements were obtained using septa-capped UV-Quartz cuvettes (10 mm pathlength) from Hellma Analytics and were recorded in a PerkinElmer LS 55 Fluorescence Spectrometer attached to a PTP 1 Peltier Temperature Programmer maintaining the temperature at 25ºC. The UV-Vis spectra were recorded in a UV-Vis spectrophotometer Cary 60 at room temperature, with the appropriate solvent. The NMR experiments (^{1}H, ^{13}C, ^{11}B {^{1}H decoupled} and ^{19}F {^{1}H decoupled}) were performed in the *Servei de Ressonància Magnètica Nuclear*, UAB, using NEO 300, NEO 400, NEO 500 or NEO 600. Chemical shifts are referenced to residual, nondeuterated $CHCl_3$ (δ 7.26 in ^{1}H NMR and 77.3 in ^{13}C NMR). The HRMS (ESI+) and elemental analyses were done by the *Parque Científico Tecnológico*, UBU. HRMS was done using a Bruker micrOTOF-QII mass spectrometer (fly time analyzer) through positive electrospray ionization. IR spectra were recorded on a Bruker Alpha II ATR-IR using either neat oil or solid products. Melting points (°C) were measured in an MPM-H2 Kleinfeld. The melting point values are uncorrected. Crystallographic data for **4** was collected on a Bruker APEX-II CCD diffractometer at 295 K, using a graphite monochromated Mo Kα radiation (λ = 0.71073 Å). Data reduction was performed using SAINT V6.45A and SORTAV in the diffractometer package.

1.2 Chemicals

Deuterated NMR solvents were purchased from Euroisotop. Dry solvents were obtained from Aldrich and used as received. Bulk DCM, EtOAc, pentane and hexane were purchased from VWR. Chemicals were purchased from Fluorochem and Aldrich and used as received unless specified.

2. List of Used Styrenes and Redox Active Phthalimides

Styrenes **1a-f**, **1h**, **1m**, **1n** and **1p** were obtained from commercial sources and used directly as received. **1g**, **1l**, **1q**, **1r** and **1u** were synthesized following the protocol from Molander.[1] **1i**[2], **1j**[3], **1o**[4] and **1t**[5] were synthesized following reported protocols.

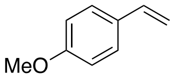

1a

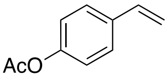

1b

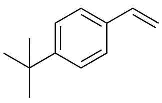
1c

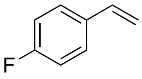

1d

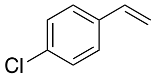

1e

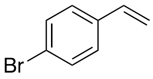

1f

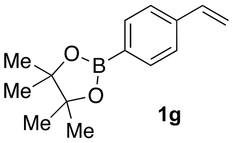

1g

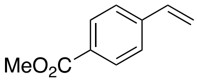

1h

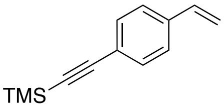

1i

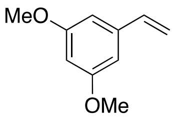

1j

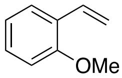

1k

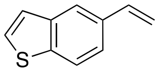

1l

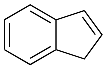
1m

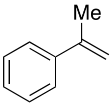

1n

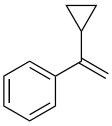
1o

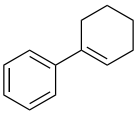
1p

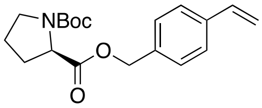

1q

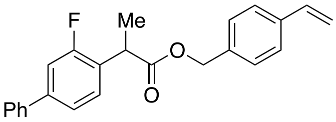

1r

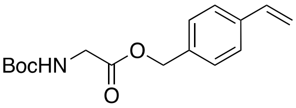

1s

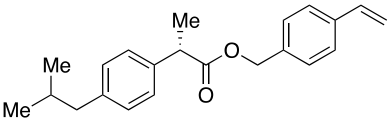

1t

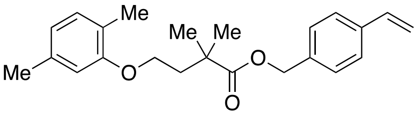

1u

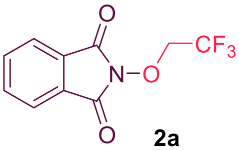

2a

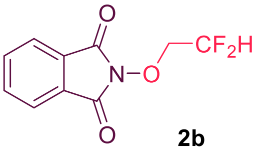

2b

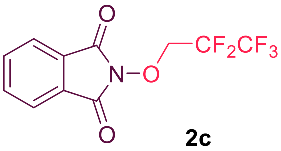

2c

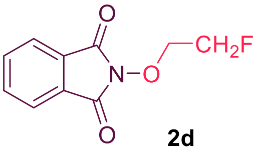

2d

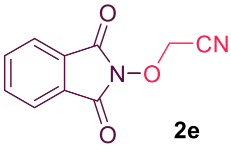

2e

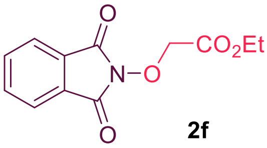

2f

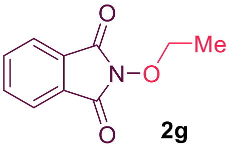

2g

---

[1] Granados, A.; Dhungana, R. K.; Sharique, M.; Majhi, J.; Molander, G. A. *Org. Lett.* **2022**, *24*, 4750–4755.
[2] Malkoch, M.; Thibault, R. J.; Drockenmuller, E.; Messerschmidt, M.; Voit, B; Russell, T. P.; Hawker, C. J. *J. Am. Chem. Soc.* **2005**, *127*, 14942–14949.
[3] W. Ning, Z. Hu, C. Tang, S. Zhang, C. Dong, J. Huang, H. Zhou, *J. Med. Chem.* 2018, **61**, 8155–8173.
[4] Cao, J.; Lv, D.; Yu, F.; Chiou, M.; Li, Y.; Bao, H. *Org. Lett.* **2021**, *23*, 3184–3189.
[5] Gallego-Gamo, A.; Sarró, P.; Ji, Y.; Pleixats, R.; Molins, E.; Gimbert-Suriñach, C.; Vallribera, A.; Granados, A. *J. Org. Chem.* **2024**, *89*, 11682–11692.

Redox active species **2a**[6] and **2b**,[5] **2c**,[7] **2d**,[8] **2e**,[9] and **2f**[10] were prepared as previously reported.

Synthesis of 2d

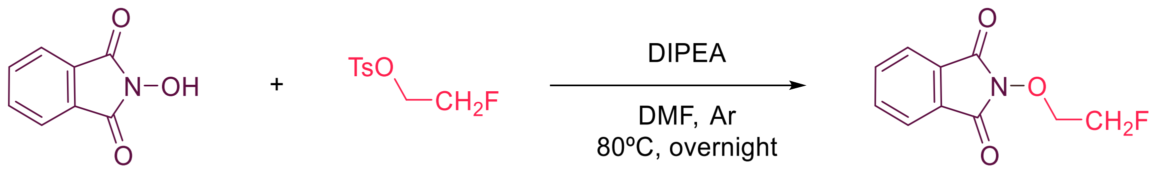

2d

In a 25 mL Schlenk flask containing a solution of *N*-hydroxyphthalimide (0.33 g, 2.0 mmol, 1 equiv) in DMF (6 mL), DIPEA (0.52 g, 4.0 mmol, 2 equiv) and 2-fluoroethyl 4-methylbenzenesulfonate (0.5 g, 2.2 mmol, 1.1 equiv) were added and kept stirring at 80ºC overnight. Subsequently, the reaction was quenched with water (10 mL) and extracted with EtOAc (3 x 10 mL). The organic layers were combined and washed with brine (3 x 10 mL), dried over Na_2SO_4, filtered, and concentrated under reduced pressure. The residue was purified by column chromatography through silica gel (hexane:EtOAc, 3:2, R_f = 0.65) to yield **2d** as a white solid (0.26 g, 1.24 mmol, 61% yield). **^{1}H NMR** (600 MHz, $CDCl_3$), δ (ppm): 7.86 (dd, *J* = 5.4, 3.1 Hz, 2H), 7.76 (dd, *J* = 5.4, 3.1 Hz, 2H), 4.84 – 4.82 (m, 1H), 4.79 – 4.72 (m, 1H), 4.51 – 4.48 (m, 1H), 4.47 – 4.44 (m, 1H). **^{13}C NMR** (75 MHz, $CDCl_3$), δ (ppm): 163.3, 134.6 (2C), 128.7, 123.6 (2C), 81.4 (d, *J* = 170.7 Hz), 76.8 (d, *J* = 19.9 Hz). **^{19}F{^{1}H} NMR** (278 MHz, $CDCl_3$), δ (ppm): -223.6. ^{1}H-NMR data in accordance with the literature.[11]

[6] Shu, C.; Noble, A.; Aggarwal, V. K. Metal-free photoinduced C(sp^3)-H borylation of alkanes. *Nature* **2020**, 586, 714–719.
[7] Lombardi, L.; Cerveri, A.; Giovanelli, R.; Castiñeira Reis, M.; Silvia López, C.; Bertuzzi, G.; Bandini, M. *Angew. Chem. Int. Ed.* **2022**, *61*, e202211732.
[8] Sharma, G. V. M.; Manohar, V.; Dutta, S. K.; Subash, V.; Kunwar, A. C. *J. Org. Chem.* **2008**, *73*, 3667–3698.
[9] Brown, M. F.; Mitton-Fry, M. J.; Arcari, J. T.; Barham, R.; Casavant, J.; Gerstenberger, B. S.; Han, S.; Hardink, J. R.; Harris, T. M.; Hoang, T.; Huband, M. D.; Lall, M. S.; Lemmon, M. M.; Li, C.; Lin, J.; McCurdy, S. P.; McElroy, E.; McPherson, C.; Marr, E. S.; Mueller, J. P.; Mullins, L.; Nikitenko, A. A.; Noe, M. C.; Penzien, J.; Plummer, M. S.; Schuff, B. P.; Shanmugasundaram, V.; Starr, J. T.; Sun, J.; Tomaras, A.; Young, J. A.; Zaniewski, R. P. *J. Med. Chem.* **2013**, *56*, 5541–5552.
[10] Ren, Z.; Mo, F.; Dong, G. *J. Am. Chem. Soc.* **2012**, *134*, 16991–16994.
[11] Ishikawa, T.; Kamiyama, K.; Matsunaga, N.; Tawada, H.; Iizawa, Y.; Okonogi, K.; Miyake, A. *J. Antibiot.* **2000**, *53*, 1071–1085.

3. Difunctionalization of Alkenes: Reaction Workflow and Compound Characterization

3.1. Reaction Workflow

All photoinduced reactions were done using a Kessil PR160L LED lamp (40 W High Luminous DEX 2100 LED, λ_{max} = 427 nm). The LED was placed 4 cm away from the reaction vial within a ventilated fume hood and using a fan to maintain the temperature approximately at 25ºC.

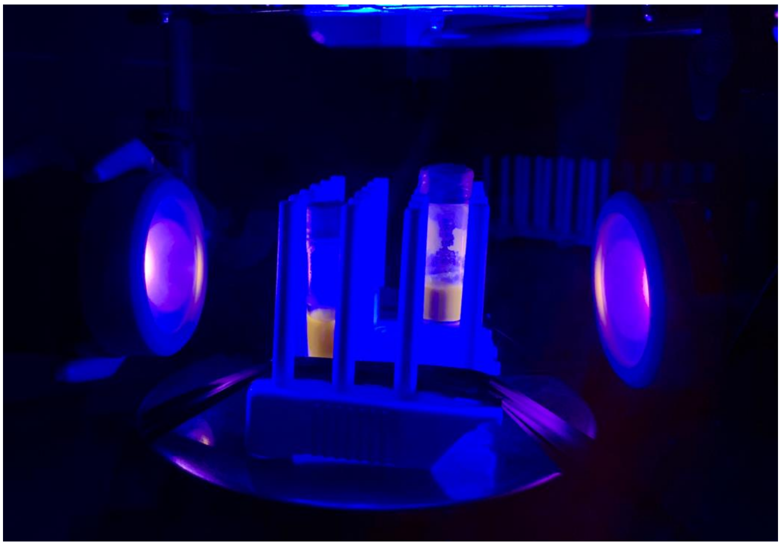

Figure S1 Reaction setup for the photoinduced difunctionalization of alkenes

3.2. Reaction Optimization

We hypothesized that alkoxy radicals from reagent **2a** could be generated and utilized without highly polar solvents (DMA, DMSO), which typically promote intramolecular 1,2-HAT via hydrogen bonding (Figure 1A). Using DCM, *p*-methoxystyrene, and methanol as model substrates, the dialkoxylated product **3** was obtained in 77% yield with 4DPAIPN under 427 nm irradiation (entry 1). Lower methanol loadings reduced efficiency, while excess MeOH or mixed solvents also diminished yields due to competing 1,2-HAT and side product formation (entries 2–8). Other solvents

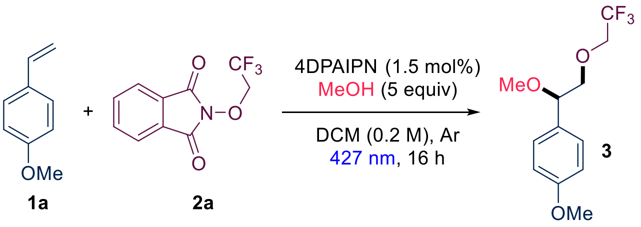

Entry	Deviation from standard conditions[a]	% Yield[b]
1	none	77 (72)
2	MeOH 1 equiv	10
3	MeOH 3 equiv	62
4	MeOH 4 equiv	70
5	MeOH 10 equiv	75
6	MeOH as solvent	37
7	MeOH as solvent and NaOMe (2 equiv)	traces
8	DCM:MeOH (1:1)	55
9	DMA	traces
10	MeCN	19
11	DME	20
12	no PC or no light	0
13	with 5 equiv of TEMPO	0

such as DMA, MeCN, and DME proved less effective (entries 9–11). Control experiments confirmed the photochemical radical nature of the process (entries 12–13).

3.3. General Procedure for the Difunctionalization of Alkenes

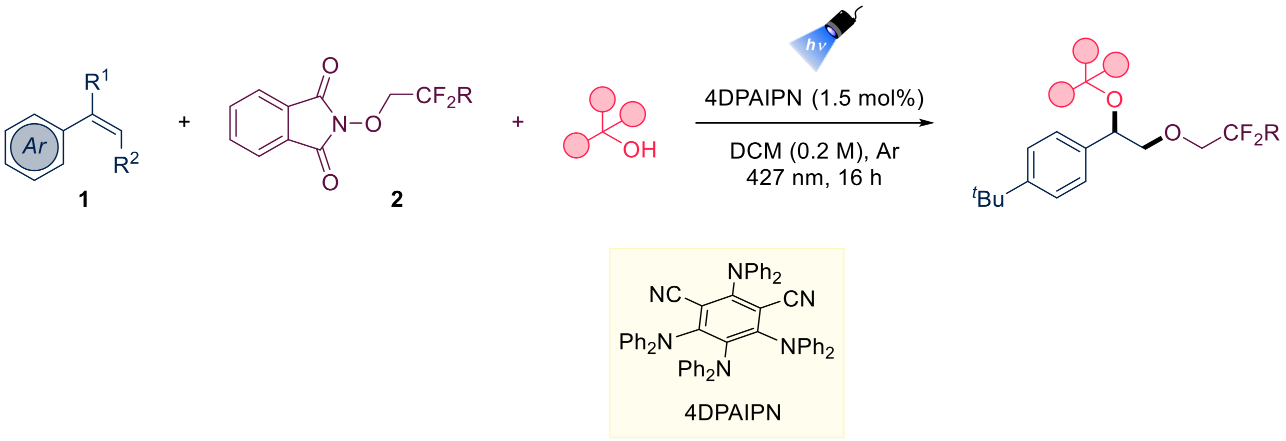

To an 8 mL vial equipped with a magnetic stir bar, styrene **1** (0.30 mmol, 1.0 equiv), **2** (0.60 mmol, 2.0 equiv), 4DPAIPN (3.5 mg, 0.0045 mmol, 1.5 mol %) and the corresponding alcohol (1.50 mmol, 5.0 equiv.) were added. Subsequently, the vial was subjected to 3 cycles of vacuum/argon degassing and then, 1.5 mL of dry and argon degassed DCM was added under inert atmosphere. The reaction mixture was irradiated overnight with a 427 nm Kessil PR160-purple LED as described in the "*Workflow*" section. The temperature of the reaction was maintained at approximately 25 °C via a fan. Upon completion, the solvent was removed under reduced pressure. The crude mixture was subjected to flash column purification using mixtures of hexanes or pentane with EtOAc, Et_2O or DCM to yield the desired product.

3.3. Compound Characterization Data

1-Methoxy-4-(1-methoxy-2-(2,2,2-trifluoroethoxy)ethyl)benzene **(3)**

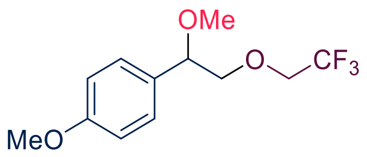

Prepared according to the *General Procedure* from the corresponding 1-methoxy-4-vinylbenzene (40.3 mg, 0.30 mmol, 1.0 equiv), **2a** (147.1 mg, 0.60 mmol, 2.0 equiv) and methanol (54.1 mg, 1.50 mmol, 5.0 equiv). After purification by flash column chromatography (hexane/AcOEt 99:1), the title compound **3** was obtained as a colorless thick oil (57.1 mg, 0.22 mmol, 72%). **R_f** = 0.50 (silica gel, hexane/AcOEt, 9:1 (v/v)). **^{1}H NMR** (500 MHz, $CDCl_3$), δ (ppm) = 7.23 (d, *J* = 8.4 Hz, 2H), 6.90 (d, *J* = 8.4 Hz, 2H), 4.36 (dd, *J* = 8.0, 3.6 Hz, 1H), 4.03 – 3.95 (m, 1H), 3.87 – 3.81 (m, 1H), 3.81 (s, 3H), 3.75 – 3.66 (m, 2H), 3.27 (s, 3H). **^{13}C{^{1}H} NMR**

(151 MHz, $CDCl_3$), δ (ppm) = 159.7, 130.2, 128.3 (2C), 124.2 (q, J = 279.9 Hz), 114.1 (2C), 83.0, 77.0, 69.2 (q, J = 33.9 Hz), 56.9, 55.4. **^{19}F NMR** (282 MHz, $CDCl_3$), δ (ppm) = -74.3. **FT-IR** (cm^{-1}, neat, ATR), ṽ = 2934, 1611, 1512, 1277, 1244, 1142, 1105, 1033, 965, 774. **HRMS (ESI)** calcd for $C_{12}H_{15}F_3O_3Na$ $[M+Na]^+$: 287.0866, found 287.0865. **MP**: < 30 ºC.

4-(1-Methoxy-2-(2,2,2-trifluoroethoxy)ethyl)phenol **(4)**

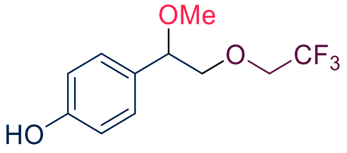

Prepared according to the *General Procedure* from the corresponding 4-vinylphenyl acetate (48.7 mg, 0.30 mmol, 1.0 equiv), **2a** (147.1 mg, 0.60 mmol, 2.0 equiv) and methanol (54.1 mg, 1.50 mmol, 5.0 equiv). After purification by flash column chromatography (hexane/AcOEt 3:1), the title compound **4** was obtained as a white solid (60.1 mg, 0.24 mmol, 80%). **R_f** = 0.29 (silica gel, hexane/AcOEt, 2:1 (v/v)). **^{1}H NMR** (500 MHz, $CDCl_3$), δ (ppm) = 7.21 – 7.16 (m, 2H), 6.86 – 6.80 (m, 2H), 4.78 (s, 1H), 4.35 (dd, J = 7.9, 3.7 Hz, 1H), 3.99 (dq, J = 12.7, 8.9 Hz, 1H), 3.85 (dq, J = 12.7, 8.7 Hz, 1H), 3.73 (dd, J = 11.0, 7.9 Hz, 1H), 3.67 (dd, J = 11.0, 3.6 Hz, 1H), 3.27 (s, 3H). **^{13}C{^{1}H} NMR** (101 MHz, $CDCl_3$), δ (ppm) = 155.7, 130.5, 128.5 (2C), 124.2 (q, J = 280.0 Hz), 115.6 (2C), 82.9, 77.0, 69.2 (q, J = 33.9 Hz), 56.9. **^{19}F NMR** (282 MHz, $CDCl_3$): -74.3. **FT-IR** (cm^{-1}, neat, ATR), ṽ = 3287, 2936, 1613, 1596, 1516, 1274, 1210, 1152, 1097, 1068, 824, 667. **HRMS (ESI)** calcd for $C_{11}H_{13}F_3O_3Na$ $[M+Na]^+$: 273.0709, found 273.0711. **MP:** 62–69ºC.

1-(Tert-butyl)-4-(1-methoxy-2-(2,2,2-trifluoroethoxy)ethyl)benzene **(5)**

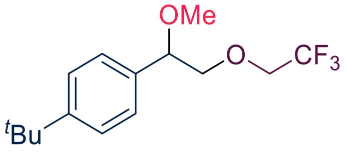

Prepared according to the *General Procedure* from the corresponding 1-(*tert*-butyl)-4-vinylbenzene (48.1 mg, 0.30 mmol, 1.0 equiv), **2a** (147.1 mg, 0.60 mmol, 2.0 equiv) and methanol (54.1 mg, 1.50 mmol, 5.0 equiv). After purification by flash column chromatography (hexane/AcOEt 99:1), the title compound **5** was obtained as a colorless oil (72.5 mg, 0.25 mmol, 83%). **R_f** = 0.80 (silica gel, hexane/AcOEt, 9:1 (v/v)).**^{1}H NMR** (300 MHz, $CDCl_3$), δ (ppm) = 7.41 – 7.35 (m, 2H), 7.25 – 7.20 (m, 2H), 4.40 (dd, J = 7.4, 4.1 Hz, 1H), 4.09 – 3.79 (m, 2H), 3.75 – 3.69 (m, 2H), 3.29 (s, 3H), 1.32 (s, 9H). **^{13}C{^{1}H} NMR** (101 MHz, $CDCl_3$), δ (ppm) = 151.3, 135.1, 126.7 (2C), 125.6 (2C), 124.2 (q, J = 279.8 Hz), 83.3, 77.1, 69.2 (q, J = 33.9 Hz), 57.1, 34.7, 31.5 (3C). **^{19}F NMR** (282 MHz, $CDCl_3$): -74.3. **FT-IR** (cm^{-1}, neat, ATR), ṽ = 2963, 2871, 1277, 1152, 1106, 966, 830, 665. **HRMS (ESI)** calcd for $C_{15}H_{21}F_3O_2Na$ $[M+Na]^+$: 313.1392, found 313.1388.

1-Fluoro-4-(1-methoxy-2-(2,2,2-trifluoroethoxy)ethyl)benzene **(6)**

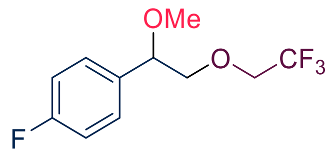

Prepared according to the *General Procedure* from the corresponding 1-fluoro-4-vinylbenzene (36.6 mg, 0.30 mmol, 1.0 equiv), **2a** (147.1 mg, 0.60 mmol, 2.0 equiv) and methanol (54.1 mg, 1.50 mmol, 5.0 equiv). The crude product was dissolved in DCM and washed with a Na_2CO_3 (2x10 mL). The title compound **6** was obtained as a colorless oil (68.1 mg, 0.28 mmol, 92%). $\mathbf{R_f}$ = 0.50 (silica gel, hexane/AcOEt, 9.5:0.5 (v/v)). **^{1}H NMR** (300 MHz, $CDCl_3$), δ (ppm) = 7.31 – 7.25 (m, 2H), 7.09 – 7.02 (m, 2H), 4.38 (dd, *J* = 7.4, 4.0 Hz, 1H), 4.04 – 3.79 (m, 2H), 3.74 – 3.67 (m, 2H), 3.27 (s, 3H). **^{13}C{^{1}H} NMR** (126 MHz, $CDCl_3$), δ (ppm) = 162.8 (d, *J* = 246.1 Hz), 134.1 (d, *J* = 3.2 Hz), 128.7 (d, *J* = 8.1 Hz, 2C), 124.1 (q, *J* = 279.8 Hz), 115.7 (d, *J* = 21.5 Hz, 2C), 82.8, 76.8, 69.2 (q, *J* = 34.0 Hz), 57.1. **^{19}F NMR** (282 MHz, $CDCl_3$): -74.3 (3F), -114.0 (1F). **FT-IR** (cm^{-1}, neat, ATR), ṽ = 2922, 2853, 1510, 1460, 1260, 1095, 1022, 800. **HRMS (ESI)** calcd for $C_{11}H_{12}F_4O_2Na$ $[M+Na]^+$: 275.0671, found 275.0675.

1-Chloro-4-(1-methoxy-2-(2,2,2-trifluoroethoxy)ethyl)benzene **(7)**

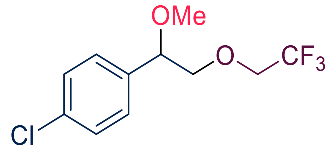

Prepared according to the *General Procedure* from the corresponding 1-chloro-4-vinylbenzene (41.4 mg, 0.30 mmol, 1.0 equiv), **2a** (147.1 mg, 0.60 mmol, 2.0 equiv) and methanol (54.1 mg, 1.50 mmol, 5.0 equiv). After purification by flash column chromatography (hexane/AcOEt 99:1), the title compound **7** was obtained as a colorless oil (56.4 mg, 0.21 mmol, 70%). $\mathbf{R_f}$ = 0.43 (silica gel, hexane/AcOEt, 9.5:0.5 (v/v)). **^{1}H NMR** (600 MHz, $CDCl_3$), δ (ppm) = 7.35 (d, *J* = 8.4 Hz, 2H), 7.26 (d, *J* = 8.4 Hz, 2H), 4.38 (dd, *J* = 7.6, 3.8 Hz, 1H), 4.02 – 3.91 (m, 1H), 3.91 – 3.78 (m, 1H), 3.76 – 3.63 (m, 2H), 3.29 (s, 3H). **^{13}C{^{1}H} NMR** (126 MHz, $CDCl_3$), δ (ppm) = 136.9, 134.2, 129.0 (2C), 128.4 (2C), 124.1 (q, *J* = 279.9 Hz), 82.8, 76.7, 69.2 (q, *J* = 34.0 Hz), 57.2. **^{19}F NMR** (282 MHz, $CDCl_3$), δ (ppm) = -74.3. **FT-IR** (cm^{-1}, neat, ATR), ṽ = 2932, 1490, 1277, 1154, 1110, 1091, 1014, 965, 825, 665. **HRMS (ESI)** calcd for $C_{11}H_{12}ClF_3O_2Na$ $[M+Na]^+$: 291.0370, found 291.0369.

1-Bromo-4-(1-methoxy-2-(2,2,2-trifluoroethoxy)ethyl)benzene **(8)**

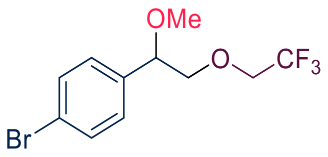

Prepared according to the *General Procedure* from the corresponding 1-bromo-4-vinylbenzene (54.9 mg, 0.30 mmol, 1.0 equiv), **2a** (147.1 mg, 0.60 mmol, 2.0 equiv) and methanol (54.1 mg, 1.50 mmol, 5.0 equiv). The crude product was dissolved in DCM and washed with a Na_2CO_3 (2x10 mL). The title compound **8** was obtained as a colorless oil (84.5 mg, 0.27 mmol, 90%). $\mathbf{R_f}$ = 0.43 (silica gel, hexane/AcOEt, 9.5:0.5 (v/v)). **^{1}H NMR** (500 MHz, $CDCl_3$), δ (ppm) = 7.50 (d, *J* = 8.0 Hz, 2H), 6.7.20 (d, *J* = 8.4 Hz, 2H), 4.36 (dd, *J* = 7.4, 3.9 Hz, 1H), 4.00 – 3.82 (m, 2H), 3.70 (qd, *J* = 11.0, 5.7 Hz, 2H), 3.29 (s, 3H). **^{13}C{^{1}H} NMR** (126 MHz, $CDCl_3$), δ (ppm) = 137.4, 131.9 (2C), 128.7 (2C), 124.1 (q, *J* = 279.8 Hz), 120.7, 82.8, 76.6, 69.2 (q, *J* = 34.0 Hz), 57.2. **^{19}F NMR** (282 MHz, $CDCl_3$): -74.3. **FT-IR** (cm^{-1}, neat, ATR), ṽ = 2988, 2932, 1478, 1275, 1150, 1121, 1095, 1020, 1005, 987, 883, 704. **HRMS (ESI)** calcd for $C_{11}H_{12}BrF_3O_2Na$ $[M+Na]^+$: 334.9865, found 334.9875.

2-(4-(1-Methoxy-2-(2,2,2-trifluoroethoxy)ethyl)phenyl)-4,4,5,5-tetramethyl-1,3,2-dioxaborolane **(9)**

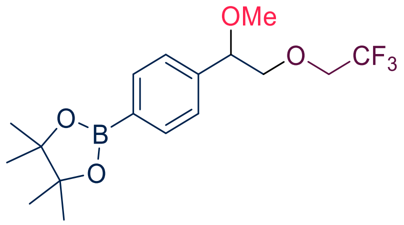

Prepared according to the *General Procedure* from the corresponding 4,4,5,5-tetramethyl-2-(4-vinylphenyl)-1,3,2-dioxaborolane (69.0 mg, 0.30 mmol, 1.0 equiv), **2a** (147.1 mg, 0.60 mmol, 2.0 equiv) and methanol (54.1 mg, 1.50 mmol, 5.0 equiv). After purification by flash column chromatography (hexane/AcOEt 95:5), the title compound **9** was obtained as a colorless oil (52.9 mg, 0.15 mmol, 49%). $\mathbf{R_f}$ = 0.25 (silica gel, hexane/AcOEt, 9.5:0.5 (v/v)). **^{1}H NMR** (400 MHz, $CDCl_3$), δ (ppm) = 7.82 (d, *J* = 8.0 Hz, 2H), 7.32 (d, *J* = 8.0 Hz, 1H), 4.42 (dd, *J* = 7.6, 3.8 Hz, 1H), 3.99 (dq, *J* = 12.7, 8.9 Hz, 1H), 3.84 (dq, *J* = 12.6, 8.7 Hz, 1H), 3.78 – 3.62 (m, 2H), 3.29 (s, 3H), 1.34 (s, 12H). **^{13}C{^{1}H} NMR** (151 MHz, $CDCl_3$), δ (ppm) = 141.4, 135.2 (2C), 126.4 (2C), 124.1 (q, *J* = 279.8 Hz), 84.0, 83.6, 76.9, 69.2 (q, *J* = 34.0 Hz), 57.2, 25.0 (4C). *C–B not observed*. **^{19}F NMR** (377 MHz, $CDCl_3$), δ (ppm) = -74.3. **^{11}B NMR** (128 MHz, CDC13), δ (ppm) = 31.1. **FT-IR** (cm^{-1}, neat, ATR), ṽ = 2980, 2933, 1613, 1359, 1275, 1140, 1088, 962, 858, 658. **HRMS (ESI)** calcd for $C_{17}H_{24}BF_3O_4Na$ $[M+Na]^+$: 383.1615, found 383.1625.

Methyl 4-(1-Methoxy-2-(2,2,2-trifluoroethoxy)ethyl)benzoate **(10)**

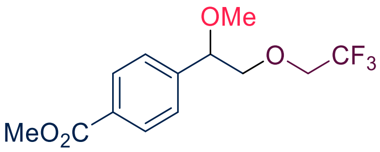

Prepared according to the *General Procedure* from the corresponding methyl 4-vinylbenzoate (48.7 mg, 0.30 mmol, 1.0 equiv), **2a** (147.1 mg, 0.60 mmol, 2.0 equiv) and methanol (54.1 mg, 1.50 mmol, 5.0 equiv). After purification by flash column chromatography (hexane/AcOEt 95:5), the title compound **10** was obtained as a colorless oil (26.3 mg, 0.09 mmol, 30%). $\mathbf{R_f}$ = 0.80 (silica gel, hexane/AcOEt, 8:2 (v/v)). **^{1}H NMR** (500 MHz, $CDCl_3$), δ (ppm) = 8.04 (d, J = 8.0 Hz, 2H), 7.40 (d, J = 8.0 Hz, 2H), 4.46 (dd, J = 7.3, 3.9 Hz, 1H), 4.04 – 3.95 (m, 1H), 3.92 (s, 3H), 3.88 – 3.81 (m, 1H), 3.73 (qd, J = 11.0, 5.7 Hz, 2H), 3.31 (s, 3H). **^{13}C{^{1}H} NMR** (126 MHz, $CDCl_3$), δ (ppm) = 166.9, 143.6, 130.3, 130.0 (2C), 126.9 (2C), 124.1 (q, J = 279.8 Hz), 83.1, 76.6, 69.3 (q, J = 34.0 Hz), 57.4, 52.3. **^{19}F NMR** (282 MHz, $CDCl_3$), δ (ppm) = -74.3. **FT-IR** (cm^{-1}, neat, ATR), ṽ = 2929, 1720, 1437, 1274, 1154, 1102, 965, 770, 707. **HRMS (ESI)** calcd for $C_{13}H_{16}F_3O_4$ [M+H]$^+$: 293.0995, found 293.1011.

((4-(1-Methoxy-2-(2,2,2-trifluoroethoxy)ethyl)phenyl)ethynyl)trimethylsilane **(11)**

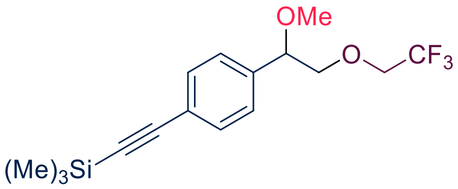

Prepared according to the *General Procedure* from the corresponding trimethyl((4-vinylphenyl)ethynyl)silane (60.1 mg, 0.30 mmol, 1.0 equiv), **2a** (147.1 mg, 0.60 mmol, 2.0 equiv) and methanol (54.1 mg, 1.50 mmol, 5.0 equiv). After purification by flash column chromatography (pentane/Et_2O 97:3), the title compound **11** was obtained as a colorless oil (37.1 mg, 0.11 mmol, 38 %). $\mathbf{R_f}$ = 0.56 (silica gel, hexane/AcOEt 10% (v/v)). **^{1}H NMR** (600 MHz, $CDCl_3$), δ (ppm) = 7.46 (d, J = 8.3 Hz, 2H), 7.25 (d, J = 8.2 Hz, 2H), 4.38 (dd, J = 7.7, 3.7 Hz, 1H), 3.96 (dq, J = 12.7, 8.8 Hz, 1H), 3.83 (dq, J = 12.7, 8.7 Hz, 1H), 3.72 (dd, J = 11.0, 7.6 Hz, 1H), 3.67 (dd, J = 11.0, 3.7 Hz, 1H), 3.27 (s, 3H), 0.25 (s, 9H). **^{13}C{^{1}H} NMR** (151 MHz, $CDCl_3$), δ (ppm) = 138.7, 132.3 (2C), 126.9 (2C), 124.1 (q, J = 279.4 Hz), 123.2, 104.8, 94.8, 83.2, 76.7, 69.2 (q, J = 34.1 Hz), 57.2, 0.1 (3C). **^{19}F NMR** (282 MHz, $CDCl_3$): -74.3. **FT-IR** (cm^{-1}, neat, ATR), ṽ = 2959, 2935, 2158, 1278, 1250, 1155, 1105, 862, 838, 759. **HRMS (ESI)** calcd for $C_{16}H_{21}F_3O_2SiNa$ [M+Na]$^+$: 353.1155, found 353.1161.

1,3-Dimethoxy-5-(1-methoxy-2-(2,2,2-trifluoroethoxy)ethyl)benzene **(12)**

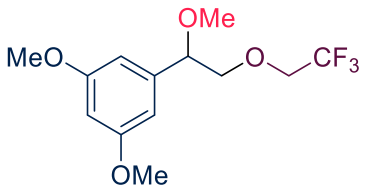

Prepared according to the *General Procedure* from the corresponding 1,3-dimethoxy-5-vinylbenzene (49.3 mg, 0.30 mmol, 1.0 equiv), **2a** (147.1 mg, 0.60 mmol, 2.0 equiv) and methanol (54.1 mg, 1.50 mmol, 5.0 equiv). After purification by flash column chromatography (hexane/AcOEt 99:1), the title compound **12** was obtained as a colorless oil (26.5 mg, 0.09 mmol, 30%). **R**$_f$ = 0.45 (silica gel, hexane/AcOEt, 9:1 (v/v)). ^{1}**H NMR** (400 MHz, $CDCl_3$), δ (ppm) = 6.47 (d, *J* = 2.3 Hz, 2H), 6.41 (t, *J* = 2.3 Hz, 1H), 4.34 (dd, *J* = 7.2, 4.1 Hz, 1H), 4.00 (dq, *J* = 12.7, 8.9 Hz, 1H), 3.91 – 3.82 (m, 1H), 3.80 (s, 6H), 3.71 (dd, *J* = 5.8, 3.9 Hz, 2H), 3.31 (s, 3H). ^{13}**C{**^{1}**H} NMR** (126 MHz, $CDCl_3$), δ (ppm) = 161.2 (2C), 140.8, 124.1 (q, *J* = 279.8 Hz), 104.9 (2C), 100.2, 83.6, 77.0, 69.2 (q, *J* = 34.0 Hz), 57.2, 55.5 (2C). ^{19}**F NMR** (282 MHz, $CDCl_3$): -74.3. **FT-IR** (cm^{-1}, neat, ATR), ṽ = 2936, 2841, 1596, 1461, 1429, 1278, 1149, 1108, 1052, 836. **HRMS (ESI)** calcd for $C_{13}H_{18}F_3O_4$ $[M+H]^+$: 295.1152, found 295.1157.

1-Methoxy-2-(1-methoxy-2-(2,2,2-trifluoroethoxy)ethyl)benzene **(13)**

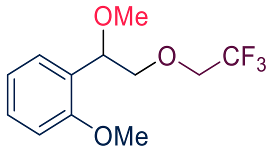

Prepared according to the *General Procedure* from the corresponding 1-methoxy-2-vinylbenzene (40.3 mg, 0.30 mmol, 1.0 equiv), **2a** (147.1 mg, 0.60 mmol, 2.0 equiv) and methanol (54.1 mg, 1.50 mmol, 5.0 equiv). After purification by flash column chromatography (hexane/AcOEt 99:1), the title compound **13** was obtained as a colorless oil (37.3 mg, 0.14 mmol, 47%). **R**$_f$ = 0.73 (silica gel, hexane/AcOEt, 9:1 (v/v)). ^{1}**H NMR** (400 MHz, $CDCl_3$), δ (ppm) = 7.36 (dd, *J* = 7.6, 1.8 Hz, 1H), 7.32 – 7.26 (m, 1H), 6.99 (td, *J* = 7.5, 1.1 Hz, 1H), 6.88 (dd, *J* = 8.2, 1.1 Hz, 1H), 4.88 (dd, *J* = 8.1, 2.6 Hz, 1H), 4.09 – 3.98 (m, 1H), 3.95 – 3.86 (m, 1H), 3.84 (s, 3H), 3.76 (dd, *J* = 11.3, 2.6 Hz, 1H), 3.63 (dd, *J* = 11.3, 8.1 Hz, 1H), 3.33 (s, 3H). ^{13}**C{**^{1}**H} NMR** (126 MHz, $CDCl_3$), δ (ppm) = 157.1, 129.0, 127.1, 126.0, 124.2, (q, *J* = 279.8 Hz), 120.8, 110.4, 77.8, 76.0, 69.1 (q, *J* = 33.9 Hz), 57.4, 55.4. ^{19}**F NMR** (282 MHz, $CDCl_3$), δ (ppm) = -74.4. **FT-IR** (cm^{-1}, neat, ATR), ṽ = 2932, 1602, 1490, 1278, 1240, 1149, 1103, 966, 755. **HRMS (ESI)** calcd for $C_{12}H_{15}F_3O_3Na$ $[M+Na]^+$: 287.0866, found 287.0865.

5-(1-Methoxy-2-(2,2,2-trifluoroethoxy)ethyl)benzo[b]thiophene **(14)**

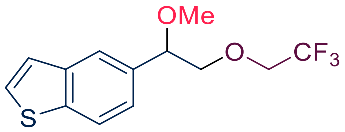

Prepared according to the *General Procedure* from the corresponding 5-vinylbenzo[*b*]thiophene (48.1 mg, 0.30 mmol, 1.0 equiv), **2a** (147.1 mg, 0.60 mmol, 2.0 equiv) and methanol (54.1 mg, 1.50 mmol, 5.0 equiv). After purification by flash column chromatography (hexane/AcOEt 99:1), the title compound **14** was obtained as a colorless oil (29.1 mg, 0.11 mmol, 35%). $\mathbf{R_f}$ = 0.65 (silica gel, hexane/AcOEt, 9:1 (v/v)). **^{1}H NMR** (500 MHz, $CDCl_3$), δ (ppm) = 7.88 (d, *J* = 8.3 Hz, 1H), 7.78 (d, *J* = 1.7 Hz, 1H), 7.48 (d, *J* = 5.5 Hz, 1H), 7.34 (d, *J* = 5.5 Hz, 1H), 7.30 (dd, *J* = 8.4, 1.7 Hz, 1H), 4.54 (dd, *J* = 7.9, 3.6 Hz, 1H), 4.02 (dq, *J* = 12.7, 8.9 Hz, 1H), 3.88 (dq, *J* = 12.7, 8.9 Hz, 1H), 3.85 – 3.71 (m, 2H), 3.32 (s, 3H). **^{13}C{^{1}H} NMR** (126 MHz, $CDCl_3$), δ (ppm) = 140.0, 139.8, 134.5, 127.3, 124.1 (q, *J* = 280.0 Hz), 123.9, 123.2, 122.9, 122.2, 83.6, 77.2, 69.3 (q, *J* = 33.9 Hz), 57.2. **^{19}F NMR** (282 MHz, $CDCl_3$), δ (ppm) = -74.2. **FT-IR** (cm^{-1}, neat, ATR), ṽ = 2930, 1436, 1276, 1148, 1107, 965, 813, 701. **HRMS (ESI)** calcd for $C_{13}H_{13}F_3O_2SNa$ $[M+Na]^+$: 313.0481, found 313.0482.

1-Methoxy-2-(2,2,2-trifluoroethoxy)-2,3-dihydro-1H-indene **(15)**

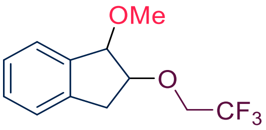

Prepared according to the *General Procedure* from the corresponding 1*H*-indene (34.8 mg, 0.30 mmol, 1.0 equiv), **2a** (147.1 mg, 0.60 mmol, 2.0 equiv) and methanol (54.1 mg, 1.50 mmol, 5.0 equiv). After purification by flash column chromatography (hexane/AcOEt 95:5), the title compound **15** was obtained as a yellow oil (64.2 mg, 0.26 mmol, 87%). The product was obtained as a mixture of separable diastereoisomers in a 1:1 diastereomeric ratio determined by ^{1}H-NMR of the crude. $\mathbf{R_f}$ = 0.50 (silica gel, hexane/AcOEt, 9:1 (v/v)). **FT-IR** (cm^{-1}, neat, ATR), ṽ = 2935, 1718, 1273, 1154, 1127, 963, 748, 735.

Data for *trans*-isomer: **^{1}H NMR** (500 MHz, $CDCl_3$), δ (ppm) = 7.38 (d, *J* = 7.4 Hz, 1H), 7.32 – 7.22 (m, 3H), 4.65 (d, *J* = 4.9 Hz, 1H), 4.39 (td, *J* = 6.7, 4.9 Hz, 1H), 4.09 – 3.91 (m, 2H), 3.45 (s, 3H), 3.13 (qd, *J* = 15.5, 6.7 Hz, 2H). **^{13}C{^{1}H} NMR** (126 MHz, $CDCl_3$), δ (ppm) = 140.2, 139.4, 129.4, 127.0, 125.9, 124.1 (q, *J* = 251.6 Hz), 82.2, 82.0, 77.4, 67.1 (q, *J* = 34.1 Hz), 56.7, 36.0. **^{19}F NMR** (377 MHz, $CDCl_3$): -74.1. **HRMS (ESI)** calcd for $C_{12}H_{13}F_3O_2Na$ $[M+Na]^+$: 269.0760, found 269.0764.

Data for *cis*-isomer: **^{1}H NMR** (500 MHz, $CDCl_3$), δ (ppm) = 7.38 (d, *J* = 7.4 Hz, 1H), 7.31 – 7.23 (m, 3H), 4.65 (d, *J* = 4.9 Hz, 1H), 4.39 (q, *J* = 6.7 Hz, 1H), 4.10 – 3.92 (m, 2H), 3.45 (s, 3H), 3.13 (qd, *J* = 15.5, 6.7 Hz,

2H). ^{13}**C{^{1}H} NMR** (126 MHz, $CDCl_3$), δ (ppm) = 140.0, 139.1, 129.0, 127.3, 125.1, 124.0 (q, J = 279.0 Hz), 88.5, 87.7, 77.4, 67.3 (q, J = 34.4 Hz), 57.6, 36.1. ^{19}**F NMR** (377 MHz, $CDCl_3$): -74.3. **HRMS (ESI)** calcd for $C_{12}H_{13}F_3O_2Na$ $[M+Na]^+$: 269.0760, found 269.0764.

(1-Methoxy-2-(2,2,2-trifluoroethoxy)cyclohexyl)benzene **(16)**

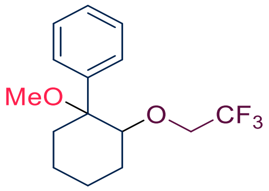

Prepared according to the *General Procedure* from the corresponding 1-phenyl-1-cyclohexene (47.5 mg, 0.30 mmol, 1.0 equiv), **2a** (147.1 mg, 0.60 mmol, 2.0 equiv) and methanol (54.1 mg, 1.50 mmol, 5.0 equiv). After purification by flash column chromatography (hexane/AcOEt 95:5), the title compound **16** was obtained as a colorless oil (86.4 mg, 0.30 mmol, 99%). The product was obtained as a mixture of diastereoisomers in a 6:1 diastereomeric ratio determined by ^{19}F-NMR of the crude. $\mathbf{R_f}$ = 0.56 (silica gel, hexane/AcOEt, 9:1 (v/v)). ^{1}**H NMR** (400 MHz, $CDCl_3$), δ (ppm) = 7.40 – 7.35 (m, 2H), 7.33 – 7.23 (m, 2H), 7.24 – 7.16 (m, 1H), 3.36 – 3.24 (m, 1H), 3.20 (dd, J = 11.1, 4.2 Hz, 1H), 3.09 (s, 2.60x3, 3H), 3.07 – 2.97 (m, 1H), 2.90 (s, 0.46x3, 3H), 2.06 (dd, 14.5, 2.8 Hz, 1H), 1.95 – 1.73 (m, 4H), 1.56 – 1.43 (m, 2H), 1.36 – 1.23 (m, 1H). ^{13}**C{^{1}H} NMR** (101 MHz, $CDCl_3$), δ (ppm) = 143.2, 141.7, 128.3, 128.2, 127.4, 127.4, 127.3, 123.9 (q, J = 280.1 Hz), 88.1, 84.5, 80.2, 79.0, 68.4 (q, J = 33.7 Hz), 67.4 (q, J = 33.7 Hz), 50.0, 49.2, 30.8, 27.9, 26.6, 24.8, 24.6, 20.7, 20.5, 19.4. ^{19}**F NMR** (377 MHz, $CDCl_3$): -75.0 (2.60x3, 3F), -75.2 (0.43x3, 3F). **FT-IR** (cm^{-1}, neat, ATR), ṽ = 2937, 2861, 1446, 1277, 1149, 1119, 1075, 976, 700. **HRMS (ESI)** calcd for $C_{15}H_{19}F_3O_2Na$ $[M+Na]^+$: 311.1229, found 311.1233.

(2-Methoxy-1-(2,2,2-trifluoroethoxy)propan-2-yl)benzene **(17)**

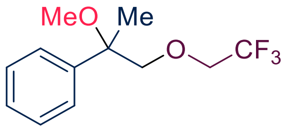

Prepared according to the *General Procedure* from the corresponding prop-1-en-2-ylbenzene (35.5 mg, 0.30 mmol, 1.0 equiv), **2a** (147.1 mg, 0.60 mmol, 2.0 equiv) and methanol (54.1 mg, 1.50 mmol, 5.0 equiv). After purification by flash column chromatography (hexane/Et_2O 95:5), the title compound **17** was obtained as a white solid (60.1 mg, 0.24 mmol, 81%). $\mathbf{R_f}$ = 0.50 (silica gel, hexane/EtOAc, 9:1 (v/v)). ^{1}**H NMR** (400 MHz, $CDCl_3$), δ (ppm) = 7.38 – 7.30 (m, 4H), 7.28 – 7.20 (m, 1H), 3.88 (dq, J = 12.7, 8.9 Hz, 1H), 3.74 (dq, J = 12.8, 8.8 Hz, 1H), 3.67 (d, J = 10.4 Hz, 1H), 3.53 (d, J = 10.4 Hz, 1H), 3.08 (s, 3H), 1.60 (s, 3H). ^{13}**C{^{1}H} NMR**(101 MHz, $CDCl_3$), δ (ppm) = 141.9, 128.5 (2C), 127.7, 126.7 (2C), 124.2 (q, J = 279.9 Hz), 80.8, 79.6,

69.3 (q, J = 33.8 Hz), 50.7, 19.4. **^{19}F NMR** (377 MHz, $CDCl_3$): -74.3. **FT-IR** (cm^{-1}, neat, ATR), ṽ = 2934, 1447, 1278, 1144, 1072, 762, 701. **HRMS (ESI)** calcd for $C_{12}H_{15}F_3O_2Na$ $[M+Na]^+$: 271.0922, found 271.0930.

(1-Cyclopropyl-1-methoxy-2-(2,2,2-trifluoroethoxy)ethyl)benzene **(18)**

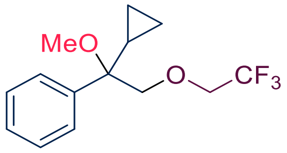

Prepared according to the *General Procedure* from the corresponding (1-cyclopropylvinyl)benzene (43.3 mg, 0.30 mmol, 1.0 equiv), **2a** (147.1 mg, 0.60 mmol, 2.0 equiv) and methanol (54.1 mg, 1.50 mmol, 5.0 equiv). After purification by flash column chromatography (hexane/DCM 3:1), the title compound **18** was obtained as a white solid (63.0 mg, 0.23 mmol, 77%). **R_f** = 0.29 (silica gel, hexane/DCM, 2:1 (v/v)). **^{1}H NMR** (500 MHz, $CDCl_3$), δ (ppm) = 7.48 – 7.42 (m, 2H), 7.36 (dd, J = 8.5, 6.9 Hz, 2H), 7.32 – 7.26 (m, 1H), 3.92 – 3.80 (m, 3H), 3.77 (dt, J = 12.7, 8.8 Hz, 1H), 3.26 (s, 3H), 1.15 – 0.68 (m, 1H), 0.61 (ddt, J = 10.1, 5.9, 3.0 Hz, 2H), 0.48 (dddd, J = 16.9, 8.9, 5.9, 4.3 Hz, 2H). **^{13}C{^{1}H} NMR** (126 MHz, $CDCl_3$), δ (ppm) = 140.9, 128.2 (2C), 127.5, 127.3 (2C), 124.2 (q, J = 280.1 Hz), 80.5, 77.0, 69.3 (q, J = 33.9 Hz), 51.3, 17.8, 2.5, 1.3. **^{19}F NMR** (377 MHz, $CDCl_3$): -74.00. **FT-IR** (cm^{-1}, neat, ATR), ṽ = 2927, 1411, 1277, 1154, 1132, 1082, 966, 702. **HRMS (ESI)** calcd for $C_{14}H_{17}F_3O_2Na$ $[M+Na]^+$: 297.1078, found 297.1071.

1-(Tert-butyl) 2-(4-(1-methoxy-2-(2,2,2-trifluoroethoxy)ethyl)benzyl) pyrrolidine-1,2-dicarboxylate **(19)**

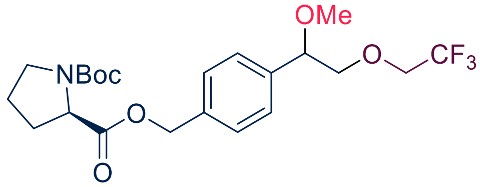

Prepared according to the *General Procedure* from the corresponding 1-(*tert*-butyl)-2-(4-vinylbenzyl) pyrrolidine-1,2-dicarboxylate (99.4 mg, 0.30 mmol, 1.0 equiv), **2a** (147.1 mg, 0.60 mmol, 2.0 equiv) and methanol (54.1 mg, 1.50 mmol, 5.0 equiv). After purification by flash column chromatography (hexane/AcOEt 9:1), the title compound **19** was obtained as a yellow oil (47.1 mg, 0.10 mmol, 34%). The product was obtained as a mixture of non-separable rotamers in a 1:1.6 ratio determined by H-NMR of the crude. **R_f** = 0.50 (silica gel, hexane/AcOEt, 7:3 (v/v)). **^{1}H NMR** (400 MHz, $CDCl_3$), δ (ppm) = 7.39 – 7.33 (m, 2H), 7.33 – 7.29 (m, 2H), 5.27 – 5.07 (m, 2H), 4.46 – 4.22 (m, 2H), 3.92 (ddd, J = 55.3, 12.6, 8.8 Hz, 2H), 3.76 – 3.64 (m, 2H), 3.59 – 3.35 (m, 2H), 3.28 (s, 3H), 2.29 – 2.12 (m, 1H), 2.02 – 1.80 (m, 3H), 1.45 (s, 3.55x1, 9H), 1.33 (s, 5.64x1, 9H). **^{13}C{^{1}H} NMR** (101 MHz, $CDCl_3$), δ (ppm) = 173.1, 172.8, 154.4, 153.8, 138.4, 138.1, 136.0, 135.8, 128.6 (2C), 128.1 (2C), 127.3 (2C), 127.1 (2C), 124.0 (q, J = 279.8 Hz), 83.1, 79.9, 79.8, 69.1 (q, J = 33.9 Hz), 66.3, 66.3, 59.2, 58.9, 57.1, 46.6, 46.3, 30.9, 29.9, 28.4 (3C), 28.3 (3C), 24.3, 23.6. **^{19}F NMR** (377

MHz, $CDCl_3$): -74.3. **FT-IR** (cm^{-1}, neat, ATR), ṽ = 2977, 2934, 2882, 1746, 1695, 1393, 1277, 1152, 1114, 919, 666. **HRMS (ESI)** calcd for $C_{22}H_{30}F_3NO_6Na$ $[M+Na]^+$: 484.1917, found 484.1926.

4-(1-Methoxy-2-(2,2,2-trifluoroethoxy)ethyl)benzyl 2-(3-fluoro-[1,1'-biphenyl]-4-yl)propanoate **(20)**

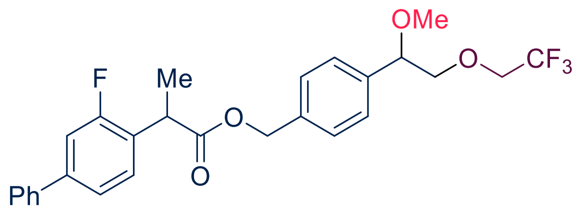

Prepared according to the *General Procedure* from the corresponding 4-vinylbenzyl 2-(3-fluoro-[1,1'-biphenyl]-4-yl)propanoate (108.1 mg, 0.30 mmol, 1.0 equiv), **2a** (147.1 mg, 0.60 mmol, 2.0 equiv) and methanol (54.1 mg, 1.50 mmol, 5.0 equiv). After purification by flash column chromatography (hexane/AcOEt 95:5), the title compound **20** was obtained as a yellow oil (67.7 mg, 0.14 mmol, 46%). **R$_f$** = 0.56 (silica gel, hexane/AcOEt, 9:1 (v/v)). **^{1}H NMR** (500 MHz, $CDCl_3$), δ (ppm) = 7.55 – 7.48 (m, 2H), 7.43 (t, *J* = 7.6 Hz, 2H), 7.36 (q, *J* = 7.6 Hz, 2H), 7.26 (s, 4H), 7.13 (dd, *J* = 7.9, 1.8 Hz, 1H), 7.08 (dd, *J* = 11.5, 1.8 Hz, 1H), 5.17 – 5.07 (m, 2H), 4.38 (dd, *J* = 7.7, 3.7 Hz, 1H), 3.96 (dq, *J* = 12.7, 8.9 Hz, 1H), 3.87 – 3.75 (m, 2H), 3.68 (dtd, *J* = 14.8, 10.9, 3.1 Hz, 2H), 3.26 (s, 3H), 1.54 (s, 3H). **^{13}C{^{1}H} NMR** (126 MHz, $CDCl_3$), δ (ppm) = 173.9, 159.8 (d, *J* = 248.3 Hz), 141.8 (d, *J* = 7.8 Hz), 138.4, 136.0, 135.6, 130.9 (d, *J* = 3.8 Hz), 129.1 (d, *J* = 2.8 Hz, 2C), 128.6 (2C), 128.4 (2C), 128.0 (d, *J* = 13.5 Hz), 127.9, 127.2 (2C), 124.1 (q, *J* = 279.8 Hz), 123.7 (d, *J* = 3.3 Hz), 115.4 (d, *J* = 23.8 Hz), 83.2, 76.9, 69.2 (q, *J* = 33.9 Hz), 66.5, 57.2, 45.2 (d, *J* = 1.4 Hz), 18.4. **^{19}F NMR** (282 MHz, $CDCl_3$): -74.3 (3F), -117.6 (1F). **FT-IR** (cm^{-1}, neat, ATR), ṽ = 2936, 1733, 1277, 1146, 1107, 964, 827, 766, 698. **HRMS (ESI)** calcd for $C_{27}H_{26}F_4O_4Na$ $[M+Na]^+$: 513.1659, found 513.1670.

4-(1-Methoxy-2-(2,2,2-trifluoroethoxy)ethyl)benzyl (tert-butoxycarbonyl)glycinate **(21)**

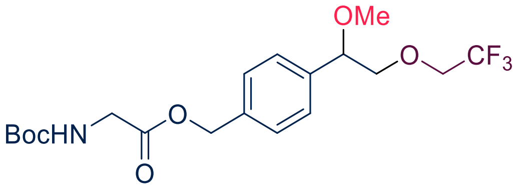

Prepared according to the *General Procedure* from the corresponding 4-vinylbenzyl (*tert*-butoxycarbonyl)glycinate (87.4 mg, 0.30 mmol, 1.0 equiv), **2a** (147.1 mg, 0.60 mmol, 2.0 equiv) and methanol (54.1 mg, 1.50 mmol, 5.0 equiv). After purification by flash column chromatography (hexane/AcOEt 8:2), the title compound **21** was obtained as an orange oil (73.3 mg, 0.17 mmol, 58%). **R$_f$** = 0.38 (silica gel, hexane/AcOEt, 7.5:2.5 (v/v)).**^{1}H NMR** (600 MHz, $CDCl_3$), δ (ppm) = 7.35 (d, *J* = 7.9 Hz, 2H), 7.31 (d, *J* = 8.0 Hz, 2H), 5.17 (s, 2H), 5.03 (bs, 1H), 4.41 (dd, *J* = 7.7, 3.7 Hz, 1H), 4.06 – 3.92 (m, 3H), 3.85 (dq, *J* = 12.7,

8.7 Hz, 1H), 3.70 (qd, J = 11.0, 5.7 Hz, 2H), 3.29 (s, 3H), 1.44 (s, 9H). **^{13}C{^{1}H} NMR** (151 MHz, $CDCl_3$), δ (ppm) = 170.4, 155.8, 138.7, 135.5 (2C), 128.8 (2C), 127.3, 124.1 (q, J = 279.9 Hz), 83.2, 80.2, 76.9, 69.2 (q, J = 34.0 Hz), 66.8, 57.2, 42.6, 28.4 (3C). **^{19}F NMR** (282 MHz, $CDCl_3$), δ (ppm) = -74.3. **FT-IR** (cm^{-1}, neat, ATR), ṽ = 3370, 2979, 2934, 1750, 1711, 1513, 1278, 1151, 1107, 965, 825, 666. **HRMS (ESI)** calcd for $C_{19}H_{26}F_3NO_6Na$ [M+Na]$^+$: 444.1604, found 444.1614.

4-(1-Methoxy-2-(2,2,2-trifluoroethoxy)ethyl)benzyl 2-(4-isobutylphenyl)propanoate **(22)**

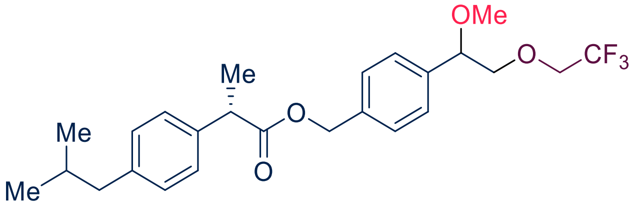

Prepared according to the *General Procedure* from the corresponding 4-vinylbenzyl 2-(4-isobutylphenyl)propanoate (96.7 mg, 0.30 mmol, 1.0 equiv), **2a** (147.1 mg, 0.60 mmol, 2.0 equiv) and methanol (54.1 mg, 1.50 mmol, 5.0 equiv). After purification by flash column chromatography (hexane/AcOEt 95:5), the title compound **22** was obtained as a colorless oil (61.1 mg, 0.14 mmol, 45%). **R_f** = 0.58 (silica gel, hexane/AcOEt, 9:1 (v/v)). **^{1}H NMR** (600 MHz, $CDCl_3$), δ (ppm) = 7.26 – 7.18 (m, 6H), 7.09 (d, J = 8.1 Hz, 2H), 5.11 (d, J = 2.3 Hz, 2H), 4.39 (dd, J = 7.8, 3.6 Hz, 1H), 3.99 (dq, J = 12.7, 8.9 Hz, 1H), 3.85 (dq, J = 12.7, 8.7 Hz, 1H), 3.79 – 3.65 (m, 3H), 3.28 (s, 3H), 2.46 (d, J = 7.2 Hz, 2H), 1.85 (hept, J = 6.8 Hz, 1H), 1.52 (d, J = 7.2 Hz, 3H), 0.91 (d, J = 6.7 Hz, 6H). **^{13}C{^{1}H} NMR** (101 MHz, $CDCl_3$), δ (ppm) = 174.7, 140.8, 138.1, 137.7, 136.4, 129.5 (2C), 128.1 (2C), 127.4 (2C), 127.1 (2C), 124.1 (q, J = 279.8 Hz), 83.2, 76.9, 69.2 (q, J = 33.9 Hz), 57.2, 45.3, 45.2, 30.3, 22.5 (2C), 18.6. **^{19}F NMR** (282 MHz, $CDCl_3$): -74.3. **FT-IR** (cm^{-1}, neat, ATR), ṽ = 2955, 2929, 1735, 1278, 1152, 1108, 965, 824. **HRMS (ESI)** calcd for $C_{25}H_{31}F_3O_4Na$ [M+Na]$^+$: 475.2072, found 475.2080.

4-(1-Methoxy-2-(2,2,2-trifluoroethoxy)ethyl)benzyl 4-(2,5-dimethylphenoxy)-2,2-dimethylbutanoate **(23)**

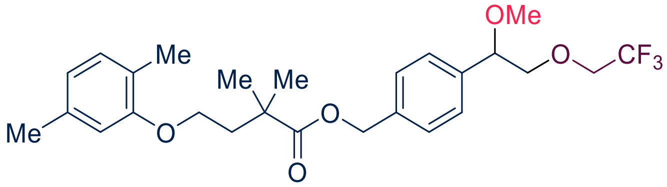

Prepared according to the *General Procedure* from the corresponding 4-vinylbenzyl 4-(2,5-dimethylphenoxy)-2,2-dimethylbutanoate (105.7 mg, 0.30 mmol, 1.0 equiv), **2a** (147.1 mg, 0.60 mmol, 2.0 equiv) and methanol (54.1 mg, 1.50 mmol, 5.0 equiv). After purification by flash column chromatography (hexane/AcOEt 95:5), the title compound **23** was obtained as a colorless oil (57.9 mg, 0.12 mmol, 40%). **R_f** = 0.56 (silica gel,

hexane/AcOEt, 9:1 (v/v)). **^{1}H NMR** (600 MHz, $CDCl_3$), δ (ppm) = 7.35 (d, J = 8.0 Hz, 2H), 7.30 (d, J = 8.0 Hz, 2H), 7.00 (d, J = 7.4 Hz, 1H), 6.66 (d, J = 7.4 Hz, 1H), 6.59 (s, 1H), 5.11 (s, 2H), 4.41 (dd, J = 7.8, 3.6 Hz, 1H), 3.98 (dq, J = 12.6, 8.8 Hz, 1H), 3.91 – 3.86 (m, 2H), 3.83 (dq, J = 12.7, 8.7 Hz, 1H), 3.74 – 3.65 (m, 2H), 3.29 (s, 3H), 2.30 (s, 3H), 2.15 (s, 3H), 1.75 – 1.72 (m, 2H), 1.25 (s, 6H). **$^{13}C\{^1H\}$ NMR** (126 MHz, $CDCl_3$), δ (ppm) = 177.7, 157.1, 138.1, 136.6, 136.6, 130.4, 128.2 (2C), 127.2 (2C), 124.1 (q, J = 279.9 Hz), 123.6, 120.9, 112.1, 83.2, 76.9, 69.2 (q, J = 33.9 Hz), 68.0, 65.9, 57.2, 42.3, 37.3, 29.8, 25.3 (2C), 21.5, 15.9. **^{19}F NMR** (377 MHz, $CDCl_3$): -74.3. **FT-IR** (cm^{-1}, neat, ATR), ṽ = 2926, 1727, 1509, 1277, 1129, 1046, 966, 804, 667. **HRMS (ESI)** calcd for $C_{26}H_{33}F_3O_5Na$ $[M+Na]^+$: 505.2178, found 505.2169.

1-Bromo-3-((1-(4-(tert-butyl)phenyl)-2-(2,2,2-trifluoroethoxy)ethoxy)methyl)benzene **(24)**

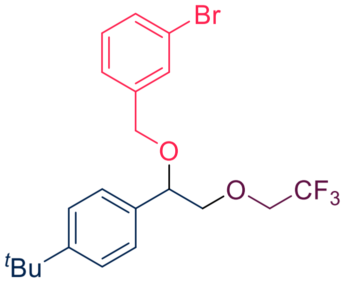

Prepared according to the *General Procedure* from the corresponding 1-(*tert*-butyl)-4-vinylbenzene (48.1 mg, 0.30 mmol, 1.0 equiv), **2a** (147.1 mg, 0.60 mmol, 2.0 equiv) and (3-bromophenyl)methanol (280.6 mg, 1.50 mmol, 5.0 equiv). After purification by flash column chromatography (hexane/AcOEt 99:1), the title compound **24** was obtained as a yellow oil (60.1 mg, 0.14 mmol, 45%). **R_f** = 0.60 (silica gel, hexane/AcOEt, 9:1 (v/v)). **^{1}H NMR** (500 MHz, $CDCl_3$), δ (ppm) = 7.49 – 7.48 (m, 1H), 7.41 – 7.39 (m, 3H), 7.26 – 7.23 (m, 3H), 7.20 (dd, J = 8.7, 6.8 Hz, 1H), 4.62 – 4.57 (m, 1H), 4.53 – 4.49 (m, 1H), 4.36 – 4.33 (m, 1H), 4.03 – 3.91 (m, 1H), 3.92 – 3.84 (m, 1H), 3.83 – 3.79 (m, 1H), 3.75 – 3.71 (m, 1H), 1.33 (s, 9H). **$^{13}C\{^1H\}$ NMR** (126 MHz, $CDCl_3$), δ (ppm) = 151.6, 140.8, 134.9, 130.8, 130.7, 130.1, 126.8 (2C), 126.2, 125.8 (2C), 124.1 (q, J = 279.6 Hz), 122.6, 81.1, 77.1, 70.1, 69.2 (q, J = 34.0 Hz), 34.8, 31.5 (3C). **^{19}F NMR** (377 MHz, $CDCl_3$): -74.3. **FT-IR** (cm^{-1}, neat, ATR), ṽ = 2961, 2868, 1726, 1277, 1154, 1105, 1082, 1019, 967, 829, 776, 732. **HRMS (ESI)** calcd for $C_{21}H_{24}BrF_3O_2Na$ $[M+Na]^+$: 467.0804, found 467.0809.

2-((1-(4-(Tert-butyl)phenyl)-2-(2,2,2-trifluoroethoxy)ethoxy)methyl)oxirane **(25)**

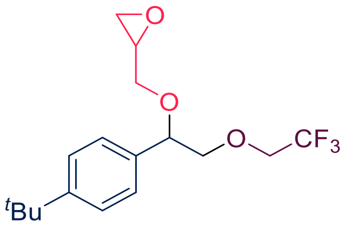

Prepared according to the *General Procedure* from the corresponding 1-(*tert*-butyl)-4-vinylbenzene (48.1 mg, 0.30 mmol, 1.0 equiv), **2a** (147.1 mg, 0.60 mmol, 2.0 equiv) and oxiran-2-ylmethanol (100 µL, 1.50 mmol, 5.0 equiv). After purification by flash column chromatography (hexane/AcOEt 99:1), the title compound **25** was obtained as a yellow oil (19.9 mg, 0.06 mmol, 20%). The product was obtained as a mixture of non-separable diastereoisomers in a 1:1 diastereomeric ratio determined by ^{19}F-NMR of the crude. $\mathbf{R_f}$ = 0.38 (silica gel, hexane/AcOEt, 9:1 (v/v)). ^{1}**H NMR** (400 MHz, $CDCl_3$), δ (ppm) = 7.37 (d, *J* = 8.3 Hz, 2H), 7.25 – 7.22 (m, 2H), 4.59 (ddd, *J* = 14.4, 7.6, 3.9 Hz, 1H), 4.09 – 3.96 (m, 1H), 3.89 (dq, *J* = 12.6, 8.7 Hz, 1H), 3.81 – 3.66 (m, 2.50x1, 5H), 3.63 (dd, *J* = 11.4, 3.2 Hz, 0.50x1, 1H), 3.43 (dd, *J* = 11.4, 5.5 Hz, 0.50x1, 1H), 3.34 (dd, *J* = 11.6, 6.0 Hz, 0.50x1, 1H), 3.15 (ddt, *J* = 7.1, 6.0, 3.0 Hz, 1H), 2.79 (dd, *J* = 5.1, 4.2 Hz, 0.50x1, 1H), 2.75 (dd, *J* = 5.0, 4.2 Hz, 0.50x1, 1H), 2.62 (dd, *J* = 5.1, 2.7 Hz, 0.50x1, 1H), 2.54 (dd, *J* = 5.0, 2.7 Hz, 0.50x1, 1H), 1.31 (s, 9H). ^{13}**C{**^{1}**H} NMR** (126 MHz, $CDCl_3$), δ (ppm) = 151.5, 151.5, 135.0, 134.9, 126.8 (2C), 126.7 (2C), 125.7 (2C), 125.7 (2C), 124.2 (q, *J* = 279.6 Hz), 77.1, 77.0, 69.9, 69.8, 69.2 (q, *J* = 34.0 Hz), 69.2 (q, *J* = 34.0 Hz), 51.1, 50.9, 44.6, 44.3, 34.7, 31.5 (3C). ^{19}**F NMR** (377 MHz, $CDCl_3$): -74.4 (1.50x1, 3F), -74.4 (1.50x1, 3F). **FT-IR** (cm^{-1}, neat, ATR), ṽ = 2961, 2870, 1277, 1153, 1106, 968, 831, 665. **HRMS (ESI)** calcd for $C_{17}H_{24}F_3O_3$ $[M+H]^+$: 333.1672, found 333.1671.

(1-(Tert-butyl)-4-(1-(prop-2-yn-1-yloxy)-2-(2,2,2-trifluoroethoxy)ethyl)benzene **(26)**

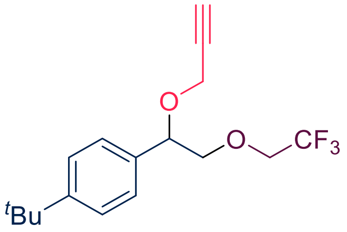

Prepared according to the *General Procedure* from the corresponding 1-(*tert*-butyl)-4-vinylbenzene (48.1 mg, 0.30 mmol, 1.0 equiv), **2a** (147.1 mg, 0.60 mmol, 2.0 equiv) and propargyl alcohol (84.1 mg, 1.50 mmol, 5.0 equiv). After purification by flash column chromatography (hexane/AcOEt 95:5), the title compound **26** was obtained as a colorless oil (51.3 mg, 0.16 mmol, 54 %). $\mathbf{R_f}$ = 0.46 (silica gel, hexane/AcOEt 10% (v/v)). ^{1}**H NMR** (500 MHz, $CDCl_3$), δ (ppm) = 7.38 (d, *J* = 8.3 Hz, 2H), 7.25 (d, *J* = 8.1 Hz, 2H), 4.77 (dd, *J* = 7.9, 3.6 Hz, 1H), 4.19 (dd, *J* = 15.7, 2.5 Hz, 1H), 4.03 (dq, *J* = 11.9, 8.9 Hz, 1H), 3.95 (dd, *J* = 15.7, 2.4 Hz, 1H), 3.92 – 3.86 (m, 1H), 3.82 – 3.74 (m, 2H), 2.41 (s, 1H), 1.32 (s, 9H). ^{13}**C{**^{1}**H} NMR** (101 MHz, $CDCl_3$), δ (ppm) = 151.7, 134.2, 127.0 (2C), 125.7 (2C), 124.2 (q, *J* = 279.8 Hz), 80.1, 79.7, 76.6, 74.6, 69.2 (q, *J* = 33.9 Hz), 56.1, 34.7, 31.4 (3C). ^{19}**F NMR** (282 MHz, $CDCl_3$): -74.3. **FT-IR** (cm^{-1}, neat, ATR), ṽ = 3325, 2977, 2875, 2130, 1252, 1124, 1100, 1090, 840, 666, 580. **HRMS (ESI)** calcd for $C_{17}H_{21}F_3O_2Na$ $[M+Na]^+$: 337.1386, found 337.1387.

((1-(Tert-butyl)phenyl)-2-(2,2,2-trifluoroethoxy)ethoxy)methyl)trimethylsilane **(27)**

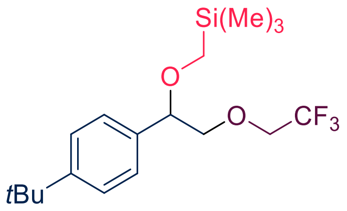

Prepared according to the *General Procedure* from the corresponding 1-(*tert*-butyl)-4-vinylbenzene (48.1 mg, 0.30 mmol, 1.0 equiv), **2a** (147.1 mg, 0.60 mmol, 2.0 equiv) and (trimethylsilyl)methanol (156.3 mg, 1.50 mmol, 5.0 equiv). After purification by flash column chromatography (pentane/Et_2O 98:2), the title compound **27** was obtained as a colorless oil (66.3 mg, 0.18 mmol, 60 %). **R_f** = 0.78 (silica gel, hexane/AcOEt 10% (v/v)). **^{1}H NMR** (500 MHz, $CDCl_3$), δ (ppm) = 7.38 (d, *J* = 8.0 Hz, 2H), 7.24 (d, *J* = 8.0 Hz, 2H), 4.49 (t, *J* = 5.8 Hz, 1H), 4.02 (dq, *J* = 12.5, 8.9 Hz, 1H), 3.87 (dq, *J* = 12.7, 8.8 Hz, 1H), 3.74 – 3.69 (m, 2H), 3.53 (td, *J* = 9.7, 6.0 Hz, 1H), 3.44 (td, *J* = 9.8, 5.9 Hz, 1H), 1.33 (s, 9H), -0,01 (s, 9H). **^{13}C{^{1}H} NMR** (126 MHz, $CDCl_3$), δ (ppm) = 151.0, 136.0, 126.6 (2C), 125.5 (2C), 124.2 (q, *J* = 279.7 Hz), 81.3, 77.9, 69.2 (q, *J* = 33.8 Hz), 66.7, 34.7, 31.5 (3C), -1.2 (3C). **^{19}F NMR** (282 MHz, $CDCl_3$): -74.4. **FT-IR** (cm^{-1}, neat, ATR), ṽ = 2956, 2870, 1278, 1249, 1153, 1093, 969, 831, 693, 665, 580. **HRMS (ESI)** calcd for $C_{18}H_{29}F_3O_2SiNa$ $[M+Na]^+$: 385.1781, found 385.1789.

1-((1-(4-(*Tert*-butyl)phenyl)-2-(2,2,2-trifluoroethoxy)ethoxy)methyl)-3-fluorobicyclo[1.1.1]pentane **(28)**

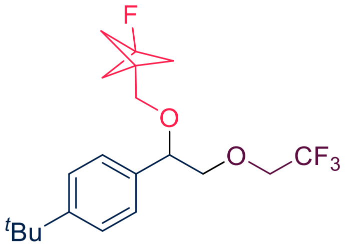

Prepared according to the *General Procedure* from the corresponding 1-(*tert*-butyl)-4-vinylbenzene (48.1 mg, 0.30 mmol, 1.0 equiv), **2a** (147.1 mg, 0.60 mmol, 2.0 equiv) and (3-fluorobicyclo[1.1.1]pentan-1-yl)methanol[12] (116.1 mg, 1.50 mmol, 5.0 equiv). After purification by flash column chromatography (hexane/DCM 3:1), the title compound **28** was obtained as a colorless oil (76.0 mg, 0.20 mmol, 68 %). **R_f** = 0.36 (silica gel, hexane/DCM 2:1 (v/v)). **^{1}H NMR** (500 MHz, $CDCl_3$), δ (ppm) = 7.37 (d, *J* = 8.4 Hz, 2H), 7.19 (d, *J* = 8.3 Hz, 2H), 4.51 (dd, *J* = 8.0, 3.6 Hz, 1H), 4.04 (dq, *J* = 12.4, 8.9 Hz, 1H), 3.89 (dq, *J* = 12.5, 8.7 Hz, 1H), 3.77 – 3.65 (m, 2H), 3.60 (dd, *J* = 33.7, 10.7 Hz, 2H), 2.01 (d, *J* = 2.7 Hz, 6H), 1.32 (s, 9H). **^{13}C{^{1}H} NMR** (126 MHz, $CDCl_3$), δ (ppm) = 151.4, 135.3, 127.5 (2C), 126.7 (2C), 124.1 (q, *J* = 278.9 Hz), 82.2, 77.1,

[12] Ripenko, V.; Vysochyn, D.; Klymov, I.; Zhersh, S.; Mykhailiuk, P. M. *J. Org. Chem.* **2021**, *86*, 14061–14068.

74.5, 69.3 (q, J = 27.2 Hz), 66.6 (d, J = 27.2 Hz), 53.1 (d, J = 20.7 Hz, 3C), 34.7, 31.5 (3C), 27.5 (d, J = 42.3 Hz). **^{19}F NMR** (377 MHz, $CDCl_3$): -74.6 (3F), -144.6 (1F). **FT-IR** (cm^{-1}, neat, ATR), ṽ = 2963, 2922, 2881, 1278, 1238, 1152, 1109, 1251, 1022, 967, 928, 830, 664, 607. **HRMS (ESI)** calcd for $C_{20}H_{26}F_4O_2Na$ $[M+Na]^+$: 397.1761, found 397.1774.

3-(1-(4-(*Tert*-butyl)phenyl)-2-(2,2,2-trifluoroethoxy)ethoxy)propan-1-ol **(29)**

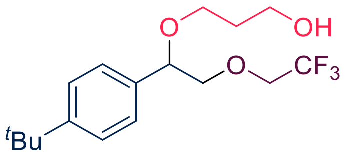

Prepared according to the *General Procedure* from the corresponding 1-(*tert*-butyl)-4-vinylbenzene (48.1 mg, 0.30 mmol, 1.0 equiv), **2a** (147.1 mg, 0.60 mmol, 2.0 equiv) and 1,3-propanediol (114.2 mg, 1.50 mmol, 5.0 equiv). After purification by flash column chromatography (hexane/AcOEt 95:5–90:10), the title compound **29** was obtained as a yellow oil (69.2 mg, 0.21 mmol, 69 %). **R_f** = 0.2 (silica gel, hexane/AcOEt 10% (v/v)). **^{1}H NMR** (500 MHz, $CDCl_3$), δ (ppm) = 7.38 (d, J = 8.1 Hz, 2H), 7.23 (d, J = 8.1 Hz, 2H), 4.51 (dd, J = 7.8, 4.0 Hz, 1H), 3.97 (dq, J = 12.7, 8.8 Hz, 1H), 3.91 – 3.81 (m, 1H), 3.79 (q, J = 5.3 Hz, 2H), 3.76 – 3.67 (m, 2H), 3.64 (dt, J = 9.5, 5.6 Hz, 1H), 3.53 (dt, J = 9.4, 5.8, 1H), 2.44 (t, J = 5.5 Hz, 1H), 1.83 (p, J = 5.7 Hz, 2H), 1.32 (s, 9H). **^{13}C{^{1}H} NMR** (126 MHz, $CDCl_3$), δ (ppm) = 151.5, 135.0, 126.6 (2C), 125.7 (2C), 124.1 (q, J = 279.8 Hz), 81.7, 77.1, 69.1 (q, J = 34.1 Hz), 68.6, 62.1, 34.7, 32.2, 31.5 (3C). **^{19}F NMR** (282 MHz, $CDCl_3$): -74.2. **FT-IR** (cm^{-1}, neat, ATR), ṽ = 3396, 2960, 2871, 1277, 1152, 1105, 1067, 967, 829, 665, 580. **HRMS (ESI)** calcd for $C_{17}H_{25}F_3O_3Na$ $[M+Na]^+$: 357.1648, found 357.1656.

5-((1-(4-(Tert-butyl)phenyl)-2-(2,2,2-trifluoroethoxy)ethoxy)methyl)-2,2,7,7-tetramethyltetrahydro-5H-bis([1,3]dioxolo)[4,5-b:4',5'-d]pyran **(30)**

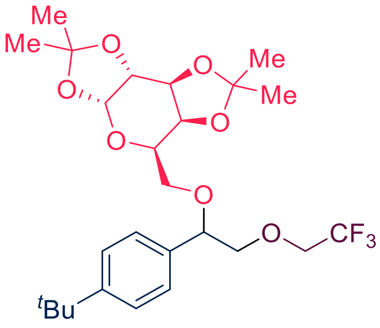

Prepared according to the *General Procedure* from the corresponding 1-(*tert*-butyl)-4-vinylbenzene (48.1 mg, 0.30 mmol, 1.0 equiv), **2a** (147.1 mg, 0.60 mmol, 2.0 equiv) and (2,2,7,7-tetramethyltetrahydro-5*H*-bis([1,3]dioxolo)[4,5-*b*:4'.5'-*d*]pyran-5-yl)methanol (390.4 mg, 1.50 mmol, 5.0 equiv). After purification by flash column chromatography (hexane/AcOEt 9:1), the title compound **30** was obtained as a yellow oil (51.0

mg, 0.10 mmol, 33 %). The product was obtained as a mixture of non-separable diastereoisomers in a 1:1 diastereomeric ratio determined by ^{19}F-NMR of the crude. **R$_f$** = 0.23 (silica gel, hexane/AcOEt 10% (v/v)). **^{1}H NMR** (500 MHz, $CDCl_3$), δ (ppm) = 7.35 (dd, *J* = 8.4, 2.7 Hz, 2H), 7.23 (dd, *J* = 8.3, 2.7 Hz, 2H), 5.52 (dd, *J* = 9.4, 5.0 Hz, 1H), 4.64 – 4.53 (m, 2H), 4.33 – 4.26 (m, 1.50x1, 3H), 4.24 (dd, *J* = 7.9, 1.9 Hz, 0.50x1, 1H), 4.15 (dqd, *J* = 12.1, 9.0, 2.9 Hz, 1H), 4.05 – 3.88 (m, 2H), 3.72 (dd, *J* = 9.8, 3.2 Hz, 2H), 3.66 (dd, *J* = 10.8, 5.0 Hz, 0.50x1, 1H), 3.61 (dd, *J* = 10.1, 6.6 Hz, 0.50x1, 1H), 3.49 (ddd, *J* = 18.0, 10.4, 6.8 Hz, 1H), 1.54 (d, *J* = 11.0 Hz, 3H), 1.40 (d, *J* = 11.1 Hz, 3H), 1.36 – 1.27 (m, 15H). **^{13}C{^{1}H} NMR** (101 MHz, $CDCl_3$), δ (ppm) = 151.3, 151.2, 135.3, 135.2, 126.8, 126.7, 125.6, 125.5, 125.3 (q, *J* = 279.0 Hz), 124.3 (q, *J* = 279.5 Hz), 109.4, 109.3, 108.7, 108.7, 96.5, 96.5, 82.8, 82.7, 76.8, 76.8, 71.3, 71.1, 70.8, 70.8, 70.8, 70.7, 69.4 (q, *J* = 15.0 Hz), 69.1 (q, *J* = 14.9 Hz), 68.3, 67.8, 67.4, 66.5, 34.7, 34.7, 31.5 (3C), 31.5 (3C), 26.2, 26.1, 26.1, 26.1, 25.1, 25.1, 24.5, 24.4. **^{19}F NMR** (282 MHz, $CDCl_3$): -74.6 (1.50x1, 3F), -74.7 (1.50x1, 3F). **FT-IR** (cm^{-1}, neat, ATR), ṽ = 2964, 1382, 1278, 1255, 1210, 1153, 1105, 1068, 1001, 977, 891, 829, 663, 580. **HRMS (ESI)** calcd for $C_{26}H_{37}O_7F_3Na$ $[M+Na]^+$: 541.2384, found 541.2392.

1-(Tert-butyl)-4-(1-(((R)-3,7-dimethyloct-6-en-1-yl)oxy)-2-(2,2,2-trifluoroethoxy)ethyl)benzene **(31)**

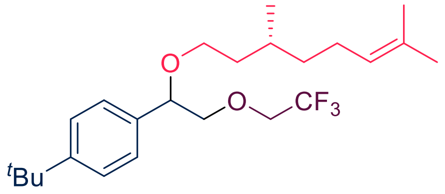

Prepared according to the *General Procedure* from the corresponding 1-(*tert*-butyl)-4-vinylbenzene (48.1 mg, 0.30 mmol, 1.0 equiv), **2a** (147.1 mg, 0.60 mmol, 2.0 equiv) and (*S*)-3,7-dimethyloct-6-en-1-ol (234.4 mg, 1.50 mmol, 5.0 equiv). After purification by flash column chromatography (hexane/Et_2O 99:1), the title compound **31** was obtained as a colorless oil (24.9 mg, 0.06 mmol, 20%). The product was obtained as a mixture of non-separable diastereoisomers in a 1:1 diastereomeric ratio determined by ^{19}F-NMR of the crude. **R$_f$** = 0.35 (silica gel, hexane/Et_2O, 98:2 (v/v)). **^{1}H NMR** (400 MHz, $CDCl_3$), δ (ppm) = 7.37 (d, *J* = 8.2 Hz, 2H), 7.22 (d, *J* = 8.1 Hz, 2H), 5.13 – 5.02 (m, 1H), 4.50 – 4.44 (m, 1H), 4.03 (dqd, *J* = 15.2, 8.9, 2.7 Hz, 1H), 3.93 – 3.81 (m, 1H), 3.70 (d, *J* = 5.3 Hz, 3H), 3.50 – 3.31 (m, 2H), 2.05 – 1.85 (m, 3H), 1.70 – 1.65 (m, 4H), 1.59 (s, 4H), 1.32 (s, 9H), 0.85 (dd, *J* = 14.0, 6.5 Hz, 4H). **^{13}C{^{1}H} NMR** (126 MHz, $CDCl_3$), δ (ppm) = 151.1, 151.1, 135.9, 135.9, 131.3, 131.2, 126.6, 126.6, 125.5, 125.3, 125.0, 124.2 (q, *J* = 279.9 Hz), 82.0, 81.9, 69.3 (q, *J* = 33.7 Hz), 67.8, 67.8, 37.4, 37.2, 37.1, 37.0, 34.7, 31.5, 29.6 (3C), 25.9, 25.6, 25.6, 19.7, 19.6, 17.8. **^{19}F NMR** (282 MHz, $CDCl_3$): -74.5 (1.50x1, 3F), -74.5 (1.50x1, 3F). **FT-IR** (cm^{-1}, neat, ATR), ṽ = 2960, 2926, 2869, 1278, 1155, 1103, 1018, 970, 829, 664. **HRMS (ESI)** calcd for $C_{24}H_{37}F_3O_2Na$ $[M+Na]^+$: 437.2638, found 437.2646.

1-(Tert-butyl)-4-(1-isopropoxy-2-(2,2,2-trifluoroethoxy)ethyl)benzene **(32)**

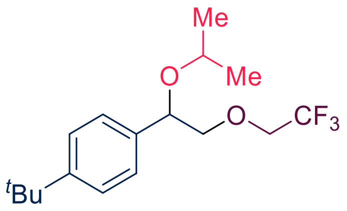

Prepared according to the *General Procedure* from the corresponding 1-(*tert*-butyl)-4-vinylbenzene (48.1 mg, 0.30 mmol, 1.0 equiv), **2a** (147.1 mg, 0.60 mmol, 2.0 equiv) and 2-propanol (90.2 mg, 1.50 mmol, 5.0 equiv). After purification by flash column chromatography (hexane/Et_2O 95:5), the title compound **32** was obtained as a colorless oil (56.9 mg, 0.18 mmol, 59 %). **R_f** = 0.68 (silica gel, hexane/Et_2O 10% (v/v)). **^{1}H NMR** (400 MHz, $CDCl_3$), δ (ppm) = 7.30 – 7.26 (m, 2H), 7.20 – 7.15 (m, 2H), 4.53 (dd, *J* = 7.2, 4.5 Hz, 1H), 3.96 (dq, *J* = 12.5, 8.9 Hz, 1H), 3.79 (dq, *J* = 12.6, 8.7 Hz, 1H), 3.63 – 3.58 (m, 2H), 3.49 (hept, *J* = 6.1 Hz, 1H), 1.24 (s, 9H), 1.10 (d, *J* = 6.1 Hz, 3H), 1.04 (d, *J* = 6.2 Hz, 3H). **^{13}C{^{1}H} NMR** (101 MHz, $CDCl_3$), δ (ppm) = 151.0, 136.6, 126.6 (2C), 125.5 (2C), 124.2 (q, *J* = 279.3 Hz), 79.0, 77.3, 69.3 (q, *J* = 33.7 Hz), 34.7, 31.5 (3C), 23.6, 21.3 (2C). **^{19}F NMR** (377 MHz, $CDCl_3$): -74.5. **FT-IR** (cm^{-1}, neat, ATR), ṽ = 2966, 2872, 1278, 1155, 1121, 1091, 1018, 992, 967, 828, 804, 664, 580. **HRMS (ESI)** calcd for $C_{17}H_{25}F_3O_2Na$ $[M+Na]^+$: 341.1699, found 341.1702.

1-(Tert-butyl)-4-(1-cyclopropoxy-2-(2,2,2-trifluoroethoxy)ethyl)benzene **(33)**

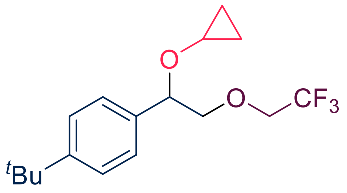

Prepared according to the *General Procedure* from the corresponding 1-(*tert*-butyl)-4-vinylbenzene (48.1 mg, 0.30 mmol, 1.0 equiv), **2a** (147.1 mg, 0.60 mmol, 2.0 equiv) and cyclopropanol (95 µL, 1.50 mmol, 5.0 equiv). After purification by flash column chromatography (hexane/Et_2O 98:2), the title compound **33** was obtained as a colorless oil (62.6 mg, 0.20 mmol, 66%). **R_f** = 0.25 (silica gel, hexane/Et_2O, 98:2 (v/v)).**^{1}H NMR** (600 MHz, $CDCl_3$), δ (ppm) = 7.39 (d, *J* = 8.3 Hz, 2H), 7.28 (d, *J* = 8.2 Hz, 2H), 4.61 (dd, *J* = 7.4, 4.4 Hz, 1H), 3.96 (dq, *J* = 12.6, 8.9 Hz, 1H), 3.83 (dq, *J* = 12.5, 8.7 Hz, 1H), 3.72 – 3.67 (m, 2H), 3.29 (tt, *J* = 6.1, 3.0 Hz, 1H), 1.33 (s, 9H), 0.70 – 0.58 (m, 2H), 0.50 – 0.36 (m, 2H). **^{13}C{^{1}H} NMR** (101 MHz, $CDCl_3$), δ (ppm) = 151.2, 135.6, 126.7 (2C), 125.6 (2C), 124.1 (q, *J* = 279.5 Hz), 81.9, 76.8, 69.2 (q, *J* = 33.9 Hz), 52.3, 31.5, 6.5, 5.6. **^{19}F NMR** (377 MHz, $CDCl_3$): -74.4. **FT-IR** (cm^{-1}, neat, ATR), ṽ = 2963, 2871, 1278, 1211, 1155, 1081, 1016, 965, 829, 665, 580. **HRMS (ESI)** calcd for $C_{17}H_{23}F_3O_2Na$ $[M+Na]^+$: 339.1542, found 339.1544.

4-(1-(Tert-butyl)phenyl)-2-(2,2,2-trifluoroethoxy)ethoxy)tetrahydro-2H-pyran **(34)**

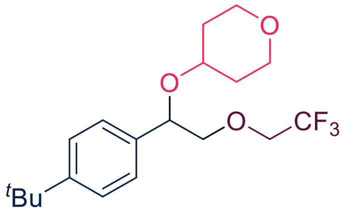

Prepared according to the *General Procedure* from the corresponding 1-(*tert*-butyl)-4-vinylbenzene (48.1 mg, 0.30 mmol, 1.0 equiv), **2a** (147.1 mg, 0.60 mmol, 2.0 equiv) and tetrahydro-2*H*-pyran-4-ol (153.2 mg, 1.50 mmol, 5.0 equiv). After purification by flash column chromatography (hexane/AcOEt 95:5), the title compound **34** was obtained as a colorless oil (37.0 mg, 0.10 mmol, 34 %). $\mathbf{R_f}$ = 0.17 (silica gel, hexane/AcOEt 10% (v/v)). **^{1}H NMR** (600 MHz, $CDCl_3$), δ (ppm) = 7.37 (d, *J* = 8.0 Hz, 2H), 7.25 (d, *J* = 8.1 Hz, 2H), 4.67 (dd, *J* = 7.8, 4.0 Hz, 1H), 4.03 (dq, *J* = 12.5, 8.8 Hz, 1H), 3.95 (dt, *J* = 11.6, 4.5 Hz, 1H), 3.92 – 3.84 (m, 2H), 3.68 (h, *J* = 7.4 Hz, 2H), 3.52 (tt, *J* = 8.6, 4.0 Hz, 1H), 3.42 – 3.35 (m, 2H), 1.99 – 1.89 (m, 1H), 1.79 – 1.71 (m, 1H), 1.69 – 1.57 (m, 2H), 1.32 (s, 9H). **^{13}C{^{1}H} NMR** (151 MHz, $CDCl_3$), δ (ppm) = 151.2, 136.1, 126.9 (2C), 126.5 (2C), 124.1 (q, *J* = 279.4 Hz), 78.7, 77.3, 72.2, 69.3 (q, *J* = 33.9 Hz), 65.9, 65.7, 34.7, 33.7, 31.8, 31.5 (3C). **^{19}F NMR** (377 MHz, $CDCl_3$): -74.5. **FT-IR** (cm^{-1}, neat, ATR), ṽ = 2958, 2867, 1277, 1153, 1090, 998, 968, 827, 665, 580. **HRMS (ESI)** calcd for $C_{19}H_{27}F_3O_3Na$ $[M+Na]^+$: 383.1805, found 383.1812.

1-(Tert-butyl)-4-(1-(cyclohexyloxy)-2-(2,2,2-trifluoroethoxy)ethyl)benzene **(35)**

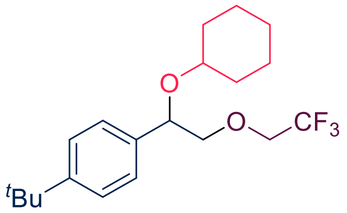

Prepared according to the *General Procedure* from the corresponding 1-(*tert*-butyl)-4-vinylbenzene (48.1 mg, 0.30 mmol, 1.0 equiv), **2a** (147.1 mg, 0.60 mmol, 2.0 equiv) and propargyl cyclohexanol (150.2 mg, 1.50 mmol, 5.0 equiv). After purification by flash column chromatography (hexane/Et_2O 97:3), the title compound **35** was obtained as a colorless oil (59.0 mg, 0.14 mmol, 46 %). $\mathbf{R_f}$ = 0.71 (silica gel, hexane/AcOEt 10% (v/v)). **^{1}H NMR** (600 MHz, $CDCl_3$), δ (ppm) = 7.38 (d, *J* = 8.3 Hz, 2H), 7.27 (d, *J* = 8.3 Hz, 2H), 4.68 (dd, *J* = 8.0, 3.8 Hz, 1H), 4.07 (dq, *J* = 12.6, 8.9 Hz, 1H), 3.91 (dq, *J* = 12.5, 8.7 Hz, 1H), 3.69 (qd, *J* = 11.3, 5.9 Hz, 2H), 3.28 (tt, *J* = 9.4, 3.8 Hz, 1H), 2.04 – 1.93 (m, 1H), 1.81 – 1.73 (m, 2H), 1.73 – 1.67 (m, 1H), 1.50 (t, *J* = 4.5 Hz, 1H), 1.40 – 1.25 (m, 10H), 1.25 – 1.18 (m, 4H). **^{13}C{^{1}H} NMR** (151 MHz, $CDCl_3$), δ (ppm) = 150.9, 136.7, 125.4 (2C), 123.3 (2C), 124.2 (q, *J* = 279.6 Hz), 78.6, 77.4, 75.7, 69.3 (q, *J* = 33.8 Hz), 34.7, 33.7, 31.5 (3C), 25.9, 24.4, 24.1. **^{19}F NMR** (282 MHz, $CDCl_3$): -74.4. **FT-IR** (cm^{-1}, neat, ATR), ṽ = 2932, 2859, 1277, 1154, 1089, 968, 828, 665, 580. **HRMS (ESI)** calcd for $C_{20}H_{29}F_3O_2Na$ $[M+Na]^+$: 381.2017, found 381.2019.

1-(Tert-butyl)-4-(1-(((1S,2R,5S)-2-isopropyl-5-methylcyclohexyl)oxy)-2-(2,2,2-trifluoroethoxy)ethyl)benzene **(36)**

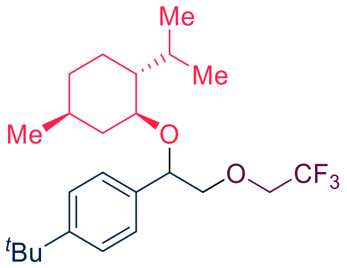

Prepared according to the *General Procedure* from the corresponding 1-(*tert*-butyl)-4-vinylbenzene (48.1 mg, 0.30 mmol, 1.0 equiv), **2a** (147.1 mg, 0.60 mmol, 2.0 equiv) and (1*S*,2*R*,5*S*)-2-isopropyl-5-methylcyclohexan-1-ol (234.4 mg, 1.50 mmol, 5.0 equiv). After purification by flash column chromatography (hexane/AcOEt 95:5), the title compound **36** was obtained as a colorless oil (83.3 mg, 0.20 mmol, 67%). The product was obtained as a mixture of non-separable diastereoisomers in a 1:1 diastereomeric ratio determined by ^{19}F-NMR of the crude. **R**$_f$ = 0.50 (silica gel, hexane/AcOEt, 9:1 (v/v)).**^{1}H NMR** (400 MHz, $CDCl_3$), δ (ppm) = 7.33 – 7.28 (m, 2H), 7.24 – 7.17 (m, 2H), 4.59 (dd, *J* = 8.0, 3.9 Hz, 0.50x1, 1H), 4.51 (dd, *J* = 7.8, 3.8 Hz, 0.50x1, 1H), 4.04 – 3.92 (m, 0.50x1, 1H), 3.91 – 3.78 (m, 1H), 3.78 – 3.57 (m, 2.50x1, 5H), 3.20 (td, *J* = 10.5, 4.2 Hz, 0.50x1, 1H), 2.93 (td, *J* = 10.5, 4.1 Hz, 0.50x1, 1H), 2.46 – 2.33 (m, 0.50x1, 1H), 2.23 – 2.11 (m, 1H), 1.70 – 1.60 (m, 0.50x1, 1H), 1.60 – 1.51 (m, 2H), 1.27 (d, *J* = 2.8 Hz, 11H), 0.91 – 0.86 (m, 3.50x1, 7H), 0.84 – 0.76 (m, 5H), 0.74 – 0.70 (m, 2H), 0.22 (d, *J* = 6.9 Hz, 1.50x1, 3H). **^{13}C{^{1}H} NMR** (101 MHz, $CDCl_3$), δ (ppm) = 151.2, 150.8, 137.7, 136.1, 127.4 (2C), 126.7 (2C), 125.3 (2C), 125.3 (2C), 124.2 (q, *J* = 268.9 Hz), 124.1 (q, *J* = 271.1 Hz), 80.6, 79.8, 77.9, 76.8, 75.7, 69.3 (q, *J* = 33.4 Hz), 69.1 (q, *J* = 33.9 Hz), 49.4, 48.6, 42.5, 40.1, 34.7, 34.7, 34.7, 34.6, 31.7, 31.6, 31.5 (3C), 31.5 (3C), 25.1, 25.0, 23.2, 22.9, 22.5, 22.4, 21.5, 21.4, 16.2, 15.5. **^{19}F NMR** (377 MHz, $CDCl_3$): -74.3 (1.50x1, 3F), -74.5 (1.50x1, 3F). **FT-IR** (cm^{-1}, neat, ATR), ṽ = 2955, 2924, 2870, 1457, 1278, 1154, 1106, 1082, 1021, 829, 666, 580. **HRMS (ESI)** calcd for $C_{24}H_{37}F_3O_2Na$ $[M+Na]^+$: 437.2638, found 437.2645.

1-(1-(Tert-butoxy)-2-(2,2,2-trifluoroethoxy)ethyl)-4-(tert-butyl)benzene **(37)**

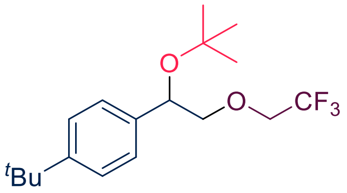

Prepared according to the *General Procedure* from the corresponding 1-(*tert*-butyl)-4-vinylbenzene (48.1 mg, 0.30 mmol, 1.0 equiv), **2a** (147.1 mg, 0.60 mmol, 2.0 equiv) and *tert*-butanol (111.2 mg, 1.50 mmol, 5.0 equiv). After purification by flash column chromatography (pentane/Et_2O 98:2), the title compound **37** was obtained

as a colorless oil (58.3 mg, 0.17 mmol, 58 %). **R$_f$** = 0.34 (silica gel, hexane/EtOAc, 5% (v/v)). **^{1}H NMR** (600 MHz, $CDCl_3$), δ (ppm) = 7.34 (d, *J* = 8.4 Hz, 2H), 7.28 (d, *J* = 8.2 Hz, 2H), 4.71 (dd, *J* = 8.3, 3.9 Hz, 1H), 3.99 (dq, *J* = 12.5, 8.9 Hz, 1H), 3.86 (dq, *J* = 12.5, 8.7 Hz, 1H), 3.64 (dd, *J* = 11.0, 3.9 Hz, 1H), 3.57 (dd, *J* = 11.0, 8.3 Hz, 1H), 1.33 (s, 9H), 1.18 (s, 9H). **^{13}C{^{1}H} NMR** (101 MHz, $CDCl_3$), δ (ppm) = 150.3, 139.1, 126.2 (2C), 125.2 (2C), 124.2 (q, *J* = 279.6 Hz), 78.0, 74.8, 74.0, 69.4 (q, *J* = 33.7 Hz), 34.6, 31.5 (3C), 28.8 (3C). **^{19}F NMR** (282 MHz, $CDCl_3$): -74.6. **FT-IR** (cm^{-1}, neat, ATR), ṽ = 2965, 2870, 1365, 1277, 1155, 1082, 1017, 967, 828, 664, 580. **HRMS (ESI)** calcd for $C_{18}H_{27}F_3O_2Na$ [M+Na]$^+$: 355.1855, found 355.1856.

1-(1-(Tert-butyl)phenyl)-2-(2,2,2-trifluoroethoxy)ethoxy)adamantane **(38)**

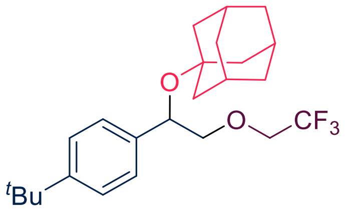

Prepared according to the *General Procedure* from the corresponding 1-(*tert*-butyl)-4-vinylbenzene (48.1 mg, 0.30 mmol, 1.0 equiv), **2a** (147.1 mg, 0.60 mmol, 2.0 equiv) and 1-adamantanol (204.4 mg, 1.50 mmol, 5.0 equiv). After purification by flash column chromatography (pentane/Et_2O 97:3), the title compound **38** was obtained as a colorless oil (61.2 mg, 0.15 mmol, 49 %). **R$_f$** = 0.68 (silica gel, hexane/AcOEt 10% (v/v)). **^{1}H NMR** (400 MHz, $CDCl_3$), δ (ppm) = 7.32 (d, *J* = 8.2 Hz, 2H), 7.26 (d, *J* = 8.2 Hz, 2H), 4.85 (dd, *J* = 8.1, 4.0 Hz, 1H), 3.98 (dq, *J* = 12.5, 8.9 Hz, 1H), 3.85 (dq, *J* = 12.4, 8.7 Hz, 1H), 3.62 (dd, *J* = 11.0, 4.0 Hz, 1H), 3.55 (dd, *J* = 11.0, 8.1 Hz, 1H), 2.10 – 2.06 (m, 3H), 1.77 – 1.64 (m, 6H), 1.64 – 1.55 (m, 6H), 1.31 (s, 9H). **^{13}C{^{1}H} NMR** (101 MHz, $CDCl_3$), δ (ppm) = 150.3, 139.2, 126.2 (2C), 125.2 (2C), 124.0 (q, *J* = 262.3 Hz), 78.0, 74.1, 72.0, 69.4 (q, *J* = 33.8 Hz), 42.7 (3C), 36.5 (3C), 34.6, 31.5 (3C), 30.7 (3C). **^{19}F NMR** (377 MHz, $CDCl_3$): -74.49. **FT-IR** (cm^{-1}, neat, ATR), ṽ = 2906, 2853, 1355, 1305, 1277, 1149, 1110, 1083, 968, 827, 664, 579. **HRMS (ESI)** calcd for $C_{24}H_{33}F_3O_2Na$ [M+Na]$^+$: 433.2325, found 433.2335.

1-(Tert-butyl)-4-(1-phenoxy-2-(2,2,2-trifluoroethyl)benzene **(39)**

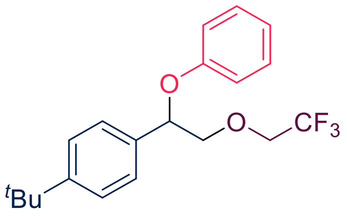

Prepared according to the *General Procedure* from the corresponding 1-(*tert*-butyl)-4-vinylbenzene (48.1 mg, 0.30 mmol, 1.0 equiv), **2a** (147.1 mg, 0.60 mmol, 2.0 equiv) and phenol (141.2 mg, 1.50 mmol, 5.0 equiv). After purification by flash column chromatography (pentane/Et_2O 97:3), the title compound **39** was obtained

as a colorless oil (18.0 mg, 0.05 mmol, 17 %). R_f = 0.6 (silica gel, hexane/AcOEt 10% (v/v)). **^{1}H NMR** (600 MHz, $CDCl_3$), δ (ppm) = 7.36 (d, J = 8.4 Hz, 2H), 7.29 (d, J = 8.3 Hz, 2H), 7.20 (dd, J = 8.7, 7.3 Hz, 2H), 6.92 – 6.85 (m, 3H), 5.33 (dd, J = 7.6, 3.8 Hz, 1H), 4.09 – 4.00 (m, 1H), 3.98 – 3.89 (m, 3H), 1.29 (s, 9H). **^{13}C{^{1}H} NMR** (151 MHz, $CDCl_3$), δ (ppm) = 158.0, 151.3, 134.7, 129.5 (2C), 126.1 (2C), 125.8 (2C), 123.9 (q, J = 262.3 Hz), 121.2, 116.1 (2C), 80.1, 77.1, 69.4 (q, J = 34.0 Hz), 34.7, 31.4 (3C). **^{19}F NMR** (282 MHz, $CDCl_3$): -74.4. **FT-IR** (cm^{-1}, neat, ATR), ṽ = 2962, 2870, 1598, 1589, 1493, 1277, 1236, 1152, 967, 829, 752, 690, 665, 570. **HRMS (ESI)** calcd for $C_{20}H_{23}F_3O_2Na$ $[M+Na]^+$: 375.1542, found 375.1550.

1-(4-(Tert-butyl)phenyl)-2-(2,2,2-trifluoroethoxy)ethan-1-ol **(40)**

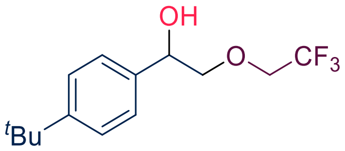

Prepared according to the *General Procedure* from the corresponding 1-(*tert*-butyl)-4-vinylbenzene (48.1 mg, 0.30 mmol, 1.0 equiv), **2a** (147.1 mg, 0.60 mmol, 2.0 equiv) and water (27 µL, 1.50 mmol, 5.0 equiv). After purification by flash column chromatography (hexane/AcOEt 95:5), the title compound **40** was obtained as a yellow oil (33.2 mg, 0.12 mmol, 40%). R_f = 0.45 (silica gel, hexane/AcOEt, 9:1 (v/v)). **^{1}H NMR** (600 MHz, $CDCl_3$), δ (ppm) = 7.39 (d, J = 8.3 Hz, 2H), 7.31 (d, J = 8.3 Hz, 2H), 4.92 (dt, J = 9.0, 2.9 Hz, 1H), 4.01 – 3.87 (m, 2H), 3.80 (dd, J = 9.9, 3.1 Hz, 1H), 3.66 (t, J = 9.4 Hz, 1H), 2.53 (d, J = 2.5 Hz, 1H), 1.31 (s, 9H). **^{13}C{^{1}H} NMR** (151 MHz, $CDCl_3$), δ (ppm) = 151.4, 136.6, 126.7 (2C), 126.1 (2C), 124.0 (q, J = 279.7 Hz), 78.1, 72.9, 69.0 (q, J = 34.3 Hz), 34.7, 31.5 (3C). **^{19}F NMR** (282 MHz, $CDCl_3$): -74.1. **FT-IR** (cm^{-1}, neat, ATR), ṽ = 3397, 2962, 2871, 1276, 1156, 1136, 994, 967, 829, 580. **HRMS (ESI)** calcd for $C_{14}H_{19}F_3O_2Na$ $[M+Na]^+$: 299.1229, found 299.1230.

1-(Tert-butyl)-4-(2-(2,2-difluoroethoxy)-1-methoxyethyl)benzene **(41)**

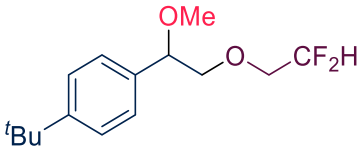

Prepared according to the *General Procedure* from the corresponding 1-(*tert*-butyl)-4-vinylbenzene (48.1 mg, 0.30 mmol, 1.0 equiv), **2b** (136.6 mg, 0.60 mmol, 2.0 equiv) and methanol (54.1 mg, 1.50 mmol, 5.0 equiv). After purification by flash column chromatography (pentane/Et_2O 95:5), the title compound **41** was obtained as a colorless oil (59.6 mg, 0.22 mmol, 73%). R_f = 0.15 (silica gel, hexane/EtOAc, 5% (v/v)). **^{1}H NMR** (400 MHz, $CDCl_3$), δ (ppm) = 7.41 – 7.35 (m, 2H), 7.26 – 7.20 (m, 2H), 5.89 (tt, J = 55.5, 4.2 Hz, 1H), 4.39 (dd, J = 8.2, 3.3 Hz, 1H), 3.86 – 3.74 (m, 1H), 3.74 – 3.65 (m, 2H), 3.62 (dd, J = 10.9, 3.3 Hz, 1H), 3.29 (s, 3H), 1.32

(s, 9H). **^{13}C{^{1}H} NMR** (101 MHz, $CDCl_3$), δ (ppm) = 151.3, 135.3, 126.7 (2C), 125.6 (2C), 114.7 (t, *J* = 241.4 Hz), 83.1, 76.9, 70.9 (d, *J* = 27.8 Hz), 57.1, 34.7, 31.5 (3C). **^{19}F NMR** (282 MHz, $CDCl_3$): -124.9 (s, 1F), -124.9 (s, 1F). **FT-IR** (cm^{-1}, neat, ATR), ṽ = 2962, 2870, 1408, 1318, 1100, 831, 577. **HRMS (ESI)** calcd for $C_{15}H_{22}F_2O_2Na$ [M+Na]$^+$: 295.1480, found 295.1487.

1-(Tert-butyl)-4-(1-methoxy-2-(2,2,3,3,3-pentafluoropropoxy)ethyl)benzene **(42)**

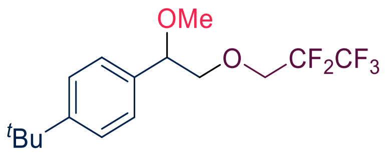

Prepared according to the *General Procedure* from the corresponding 1-(*tert*-butyl)-4-vinylbenzene (48.1 mg, 0.30 mmol, 1.0 equiv), **2c** (177.1 mg, 0.60 mmol, 2.0 equiv) and methanol (54.1 mg, 1.50 mmol, 5.0 equiv). After purification by flash column chromatography (pentane/Et_2O 100:0–97:3), the title compound **42** was obtained as a colorless oil (70.1 mg, 0.21 mmol, 69 %). **R_f** = 0.68 (silica gel, hexane/EtOAc, 9:1 (v/v)). **^{1}H NMR** (500 MHz, $CDCl_3$), δ (ppm) = 7.39 (d, *J* = 8.0 Hz, 2H), 7.23 (d, *J* = 7.9 Hz, 2H), 4.39 (dd, *J* = 7.0, 4.6 Hz, 1H), 4.13 (q, *J* = 13.5 Hz, 1H), 3.94 (q, *J* = 12.9 Hz, 1H), 3.76 – 3.65 (m, 2H), 3.30 (s, 3H), 1.32 (s, 9H). **^{13}C{^{1}H} NMR** (126 MHz, $CDCl_3$), δ (ppm) = 151.4, 135.1, 126.7 (2C), 125.6 (2C), 118.9 (qt, *J* = 286.0, 35.0 Hz), 113.3 (tq, *J* = 255.2, 36.8 Hz), 83.4, 77.4, 68.4 (t, *J* = 25.9 Hz), 57.1, 34.7, 31.5 (3C). **^{19}F NMR** (282 MHz, $CDCl_3$): -83.6 (s, 3F), -123.6 (d, *J* = 3.8 Hz, 2F). **FT-IR** (cm^{-1}, neat, ATR), ṽ = 2964, 2907, 1193, 1141, 1100, 1034, 831, 727, 580. **HRMS (ESI)** calcd for $C_{16}H_{21}F_5O_2Na$ [M+Na]$^+$: 363.1354, found 363.1355.

1-(Tert-butyl)-4-(2-(2-fluoroethoxy)-1-methoxyethyl)benzenebenzene **(43)**

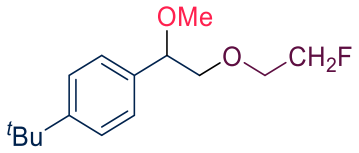

Prepared according to the *General Procedure* from the corresponding 1-(*tert*-butyl)-4-vinylbenzene (48.1 mg, 0.30 mmol, 1.0 equiv), **2d** (125.5 mg, 0.60 mmol, 2.0 equiv) and methanol (54.1 mg, 1.50 mmol, 5.0 equiv). After purification by flash column chromatography (hexane/EtOAc from 100:0 to 4:1), the title compound **43** was obtained as a colorless oil (44.3 mg, 0.17 mmol, 58 %). **R_f** = 0.40 (silica gel, hexane/EtOAc, 4:1 (v/v)). **^{1}H NMR** (400 MHz, $CDCl_3$), δ (ppm) = 7.37 (d, *J* = 8.3 Hz, 2H), 7.24 (d, *J* = 8.4 Hz, 2H), 4.67 – 4.60 (m, 1H), 4.55 – 4.47 (m, 1H), 4.41 (dd, *J* = 8.4, 3.3 Hz, 1H), 3.87 – 3.66 (m, 3H), 3.57 (dd, *J* = 10.8, 3.3 Hz, 1H), 3.29 (s, 3H), 1.32 (s, 9H). **^{13}C{^{1}H} NMR** (101 MHz, $CDCl_3$), δ (ppm) = 151.0, 135.6, 126.6 (2C), 125.4 (2C), 83.2 (d, *J* = 169.0 Hz), 83.0, 76.2, 70.6 (d, *J* = 19.8 Hz), 57.0, 34.6, 31.4 (3C). **^{19}F NMR** (376 MHz, $CDCl_3$):

-222.8 (s, 1F). **FT-IR** (cm^{-1}, neat, ATR), ṽ = 2962, 2870, 1410, 1319, 1105, 831, 577. **HRMS (ESI)** calcd for $C_{15}H_{23}FO_2Na$ $[M+Na]^+$: 277.1574, found 277.1575.

4. Large-scale synthesis of 3

To a 25 mL round bottomed Schlenk flask equipped with a magnetic stir bar, 1-methoxy-4-vinylbenzene (0.24 g, 1.5 mmol, 1.0 equiv), **2a** (0.73 g, 3.0 mmol, 2.0 equiv), 4DPAIPN (18.0 mg, 0.023 mmol, 1.5 mol %) and methanol (0.24 g, 7.5 mmol, 5.0 equiv) were added. Subsequently, the flask was subjected to 3 cycles of vacuum/argon degassing and then, 7.5 mL of dry and argon degassed DCM was added under inert atmosphere. The reaction mixture was irradiated overnight with a 427 nm Kessil PR160-purple LED as described in the "*Workflow*" section. The temperature of the reaction was maintained at approximately 25 °C via a fan. Upon completion, the solvent was removed under reduced pressure. After purification by flash column chromatography (hexane/AcOEt 99:1), the title compound **3** was obtained as a colorless oil (0.31 g, 1.05 mmol, 70 %). **^{1}H NMR** (500 MHz, $CDCl_3$), δ (ppm) = 7.23 (d, J = 8.4 Hz, 2H), 6.90 (d, J = 8.4 Hz, 2H), 4.36 (dd, J = 8.0, 3.6 Hz, 1H), 4.03 – 3.95 (m, 1H), 3.87 – 3.81 (m, 1H), 3.81 (s, 3H), 3.75 – 3.66 (m, 2H), 3.27 (s, 3H). **^{13}C{^{1}H} NMR** (151 MHz, $CDCl_3$), δ (ppm) = 159.7, 130.2, 128.3 (2C), 124.2 (q, J = 279.9 Hz), 114.1 (2C), 83.0, 77.0, 69.2 (q, J = 33.9 Hz), 56.9, 55.4. **^{19}F NMR** (282 MHz, $CDCl_3$), δ (ppm) = -74.3. **FT-IR** (cm^{-1}, neat, ATR), ṽ = 2934, 1611, 1512, 1277, 1244, 1142, 1105, 1033, 965, 774. **HRMS (ESI)** calcd for $C_{12}H_{15}F_3O_3Na$ $[M+Na]^+$: 287.0866, found 287.0865.

5. Data of X-Ray Structure of Compound 4

Crystals of **4** were obtained by dissolving the sample in a 1:10 DCM/Et_2O mixture and allowing the solution to slowly evaporate at room temperature, obtaining good quality crystals after 2 weeks.

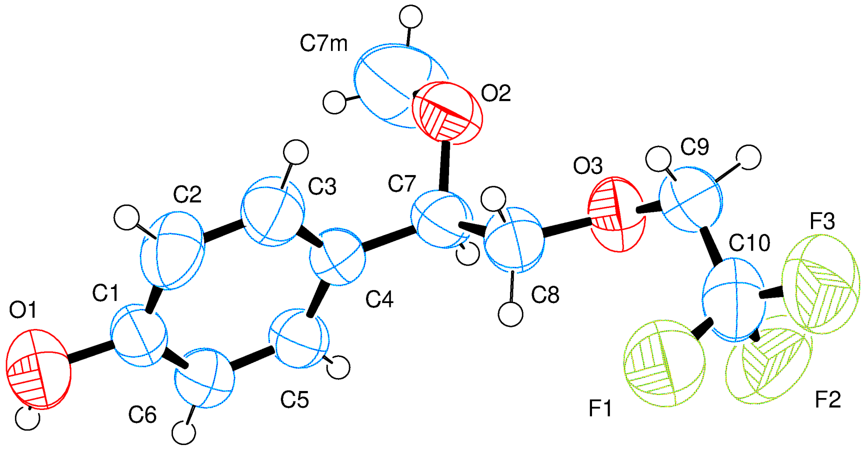

***Figure S2** Ortep view of **4** showing the atom labelling and the thermal vibration ellipsoids at 50% probability.*

***Table S1.** Crystal data and structure refinement for **4**.*

Identification code	**4**	
Empirical formula	$C_{11}H_{13}F_3O_3$	
Formula weight	250.21	
Temperature	295(2) K	
Wavelength	0.71073 Å	
Crystal system	Monoclinic	
Space group	P 21/n	
Unit cell dimensions	a = 8.241(6) Å	α= 90°.
	b = 26.361(17) Å	β= 93.066(14)°.
	c = 22.159(15) Å	γ= 90°.
Volume	4807(6) Å^3	
Z	16	
Density (calculated)	1.383 Mg/m^3	
Absorption coefficient	0.129 mm^{-1}	
F(000)	2080	
Crystal size	0.410 x 0.080 x 0.080 mm^3	
Theta range for data collection	1.545 to 28.556°.	
Index ranges	-10<=h<=11, -35<=k<=35, -29<=l<=29	
Reflections collected	141656	

Independent reflections	12127
Completeness to theta = 25.242°	100.0 %
Refinement method	Full-matrix least-squares on F^2
Data / restraints / parameters	12127 / 0 / 649
Goodness-of-fit on F^2	0.743
Final R indices [I>2sigma(I)]	R1 = 0.0642, wR2 = 0.1473
R indices (all data)	R1 = 0.3270, wR2 = 0.2232
Largest diff. peak and hole	0.305 and -0.308 e.Å^{-3}

6. Mechanistic Investigation

6.1. TEMPO experiment

To a 4 mL vial equipped with a magnetic stir bar, **2a** (49.0 mg, 0.2 mmol, 2.0 equiv), 4DPAIPN (1.3 mg, 0.0015 mmol, 1.5 mol %) and TEMPO (78.1 mg, 0.5 mmol, 5.0 equiv) were added. Subsequently, the flask was kept under vacuum for 10 minutes prior to being subjected to 3 cycles of vacuum/argon degassing. Then, 1-(*tert*-butyl)-4-vinylbenzene (16.0 mg, 0.1 mmol, 1.0 equiv), 0.5 mL of dry and argon degassed DCM and methanol (18.0 mg, 0.5 mmol, 5.0 equiv) were added under inert atmosphere. The reaction mixture was irradiated overnight with a 427 nm Kessil PR160-purple LED as described in the "*Workflow*" section. The temperature of the reaction was maintained at approximately 25 °C via a fan. Upon completion, the solvent was removed under reduced pressure. The crude mixture was analyzed by ^{1}H-NMR using 1,3,5-trimethoxybenzene as internal standard resulting in 0% yield of compound **3**. The mixture was also analyzed by HRMS, indicating the formation of a TEMPO adduct identified as **47**.

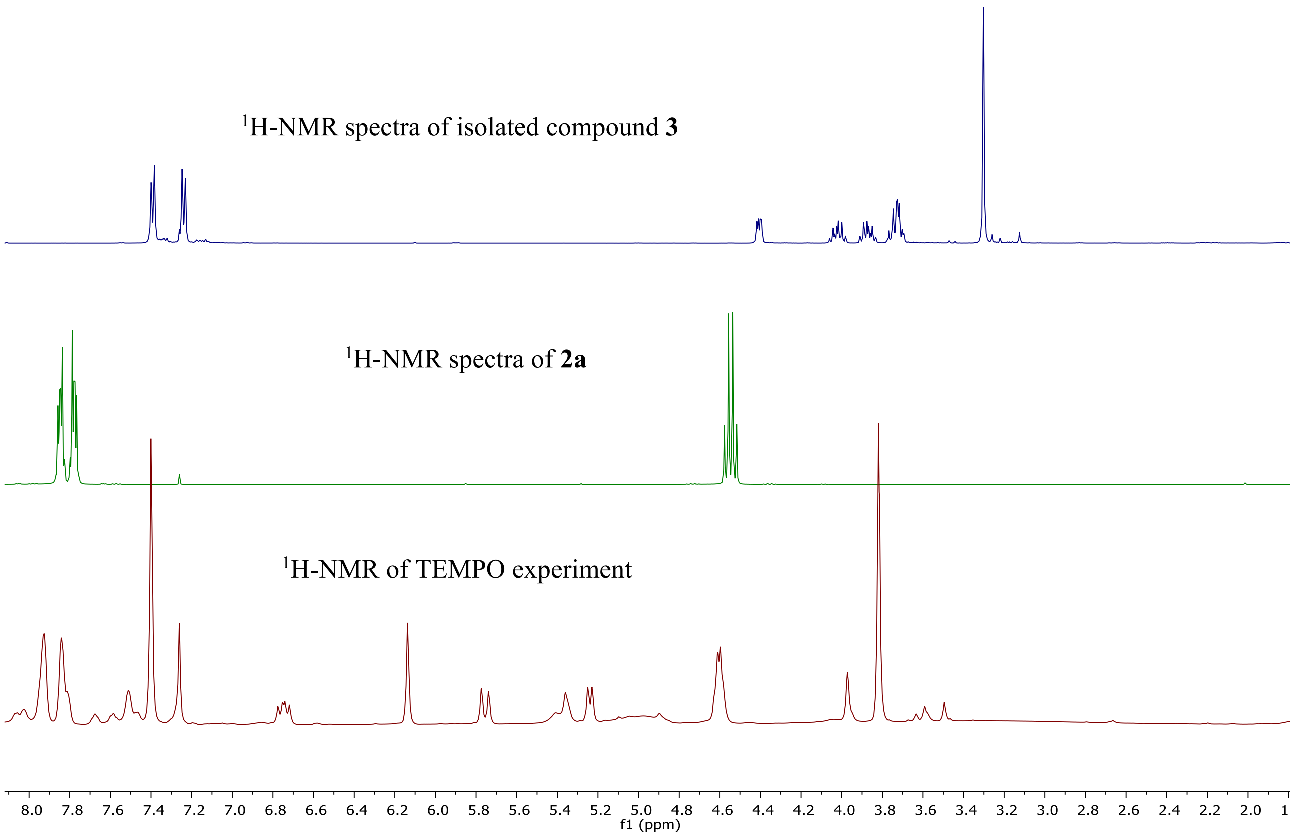

***Figure S3** Comparison between ^{1}H NMR (300 MHz, $CDCl_3$) of the TEMPO experiment reaction using 1,3,5-trimethoxybenzene as internal standard and the pure spectra of **2a** and **3**.*

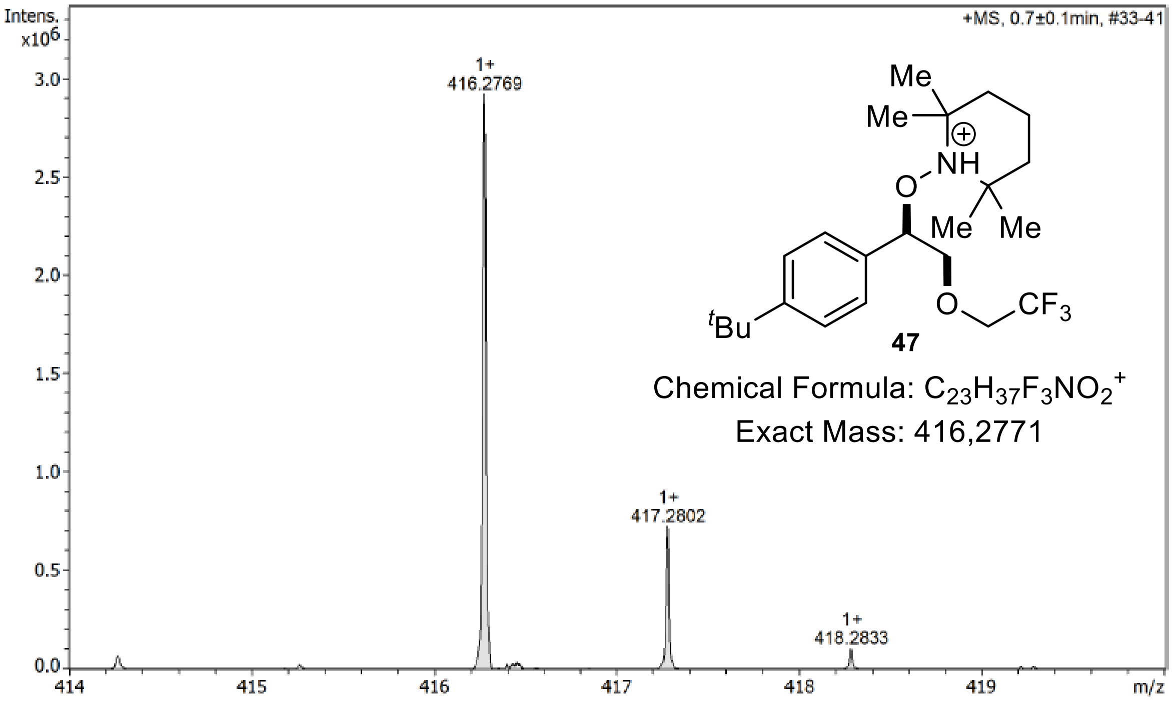

#	m/z	I	I %
1	416.2769	2923127	100.0
2	417.2802	724044	24.8
3	418.2833	102693	3.5

6.2. Cyclic voltammetry

Cyclic voltammetry of 4DPAIPN has been conducted in order to obtain reversible or quasi-reversible waves as an exact value of $E_{1/2}$ to be used in the mechanistic insight.

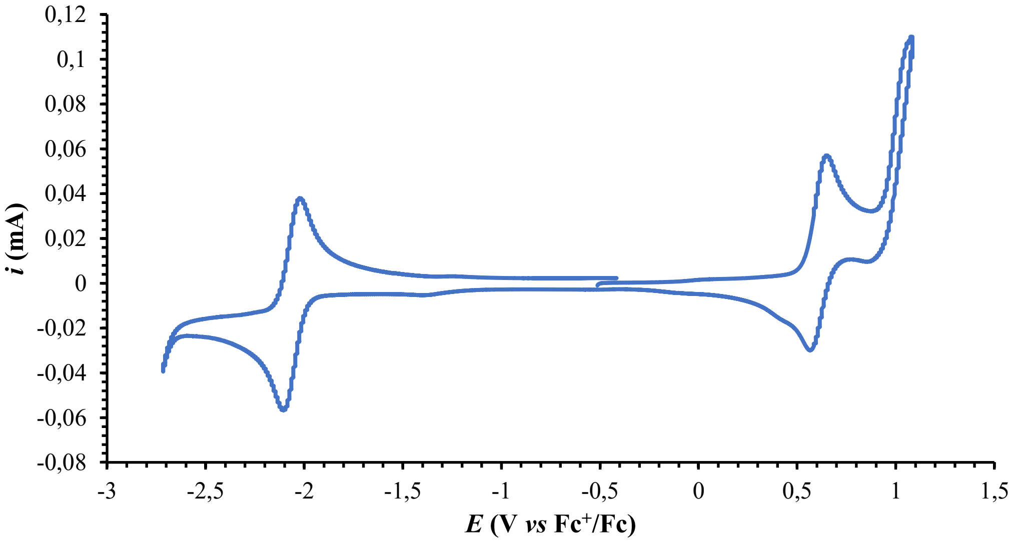

Figure S4 *Cyclic voltammetry of 4DPAIPN. Conditions: 4DPAIPN 2.0 mM (100 mV/s) in MeCN, TBAPF$_6$ 0.1 M, rt. Start at 0.0 V, scan direction to negative potentials. Glassy carbon disk as working electrode, platinum wire as auxiliary electrode and SCE as reference electrode. IUPAC plotting.*

Cyclic voltammetry of styrene **1c** has been recorded through oxidative waves to discard possible reductive quenching with 4DPAIPN.

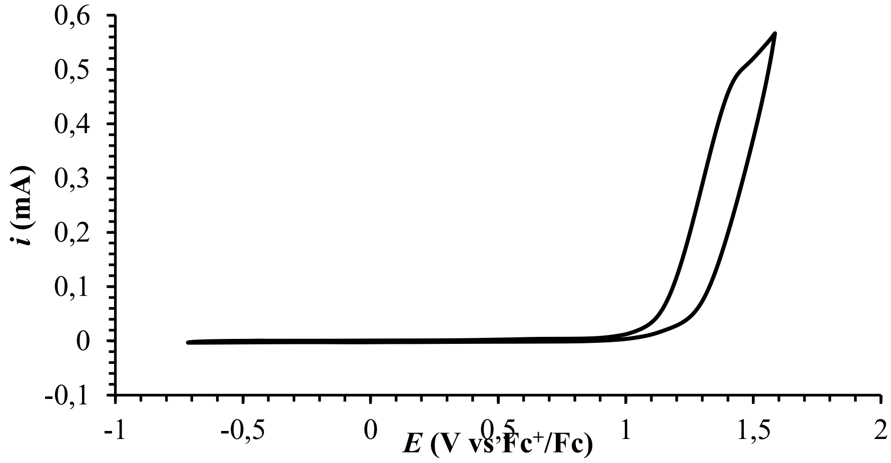

Figure S5 *Cyclic voltammetry of styrene **1c**. Conditions: **1c** 5.0 mM (100 mV/s) in MeCN, TBAPF$_6$ 0.1 M, rt. Start at 0.0 V, scan direction to positive potentials. Glassy carbon disk as working electrode, platinum wire as auxiliary electrode and SCE as reference electrode. IUPAC plotting.*

Cyclic voltammetry experiments of each fluorinated redox active species (**2a-d**) were recorded. Different non-fluorinated analogues of redox active species **2e-g** were recorded to evaluate fluorine's influence.

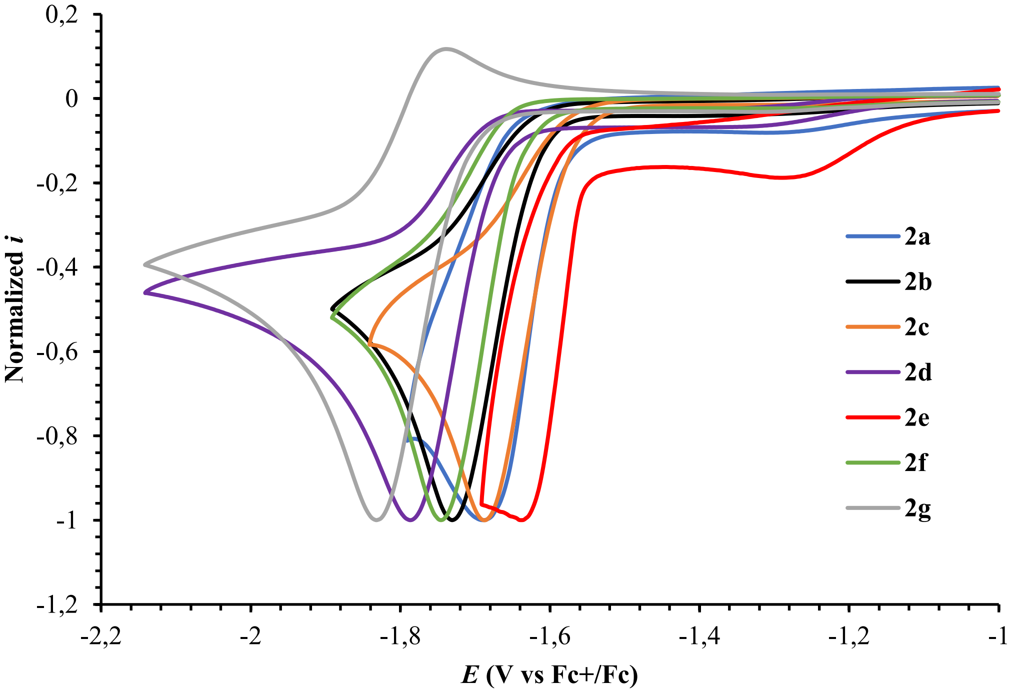

Figure S6 *Cyclic voltammetry of redox active species **2a** (blue), **2b** (black), **2c** (orange), **2d** (purple), **2e** (red), **2f** (green) and 2g (grey). Conditions: **2a** 2.5 mM (100 mV/s), **2b** 2.7 mM (100 mV/s), **2c** 2.6 mM (100 mV/s), **2d** 2.8 mM (100 mV/s), **2e** 2.5 mM (100 mV/s), **2f** 2.6 mM (100 mV/s) and **2g** 2.5 mM (100 mV/s) in MeCN, TBAPF$_6$ 0.1 M, rt. Start at 0.0 V, scan direction to negative potentials. Glassy carbon disk as working electrode, platinum wire as auxiliary electrode and SCE as reference electrode. IUPAC plotting.*

Cyclic voltammetry experiments of compound **2a** and a mixture of **2a** with 5 equiv of MeOH were conducted to evaluate changes in electrochemical properties. A clear cathodic shift (~200 mV) was observed, suggesting the formation of a new species with higher electron density (**2a**·MeOH, Figure S7).

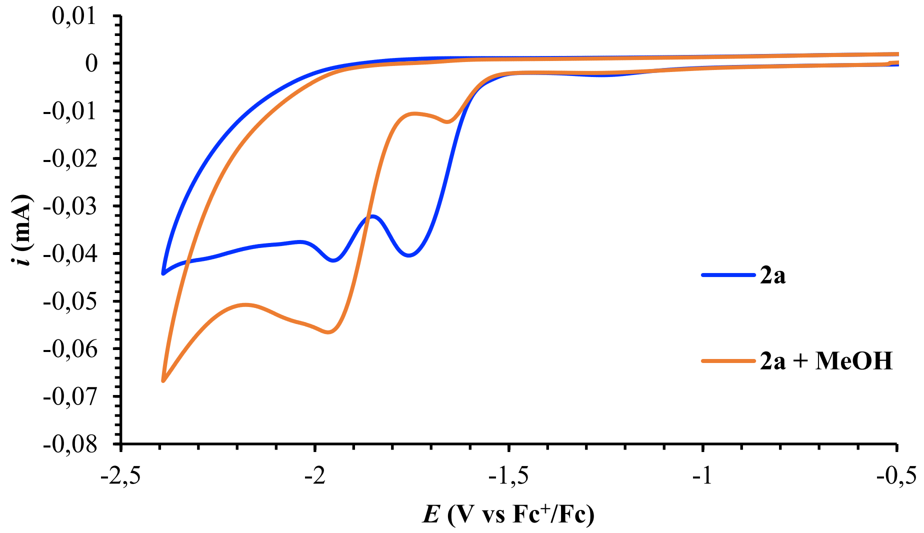

Figure S7 *Cyclic voltammetry of redox active species **2a** (blue), and after adding 5 equiv of MeOH (orange). Conditions: **2a** 2.5 mM (100 mV/s) in MeCN, TBAPF$_6$ 0.1 M, rt. Start at 0.0 V, scan direction to negative potentials. Glassy carbon disk as working electrode, platinum wire as auxiliary electrode and SCE as reference electrode. IUPAC plotting.*

Cyclic voltammetry experiments of compound **2b** and a mixture of **2b** with 5 equiv of MeOH were conducted to evaluate changes in electrochemical properties. An apparition of an additional wave with a cathodic shift (~200 mV) was observed (Figure S8).

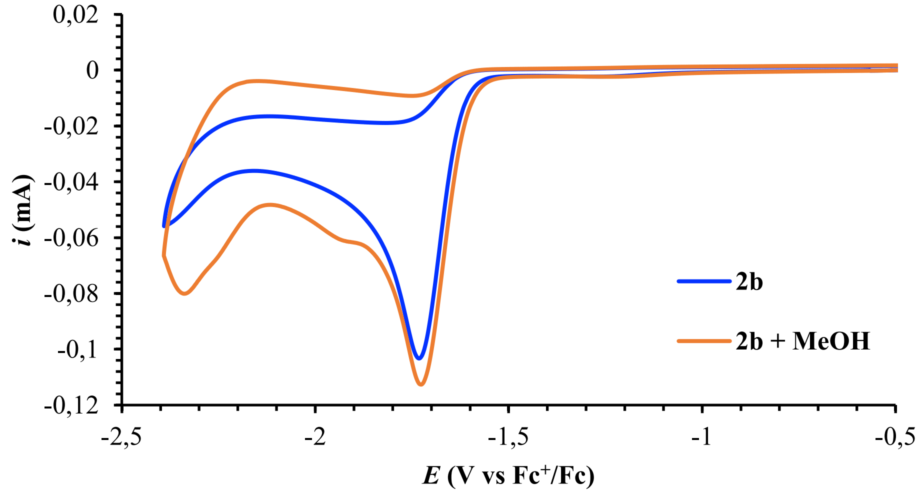

Figure S8 *Cyclic voltammetry of redox active species **2b** (blue), and after adding 5 equiv of MeOH (orange). Conditions: **2b** 2.5 mM (100 mV/s) in MeCN, TBAPF$_6$ 0.1 M, rt. Start at 0.0 V, scan direction to negative potentials. Glassy carbon disk as working electrode, platinum wire as auxiliary electrode and SCE as reference electrode. IUPAC plotting.*

Cyclic voltammetry experiments of compound **2c** and a mixture of **2c** with 5 equiv of MeOH were conducted to evaluate changes in electrochemical properties. An apparition of an additional wave with a cathodic shift (~250 mV) was observed (Figure S9).

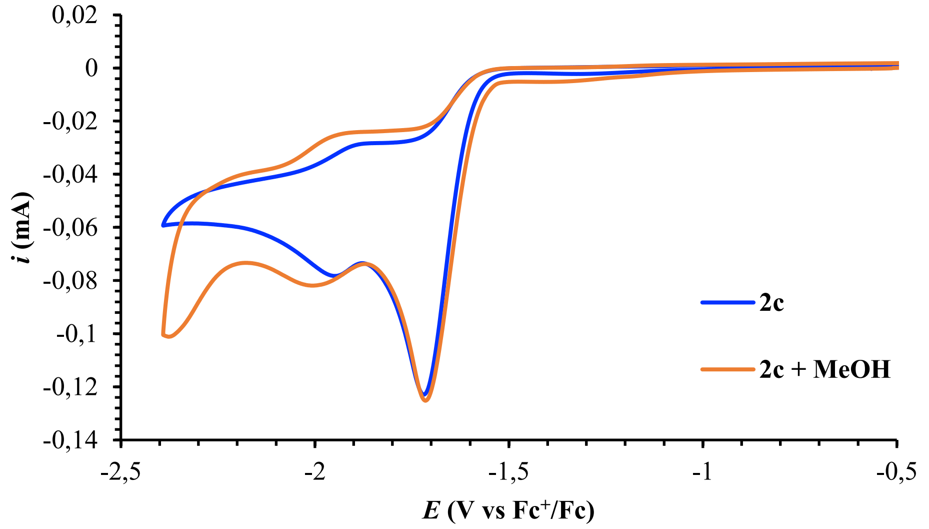

Figure S9 *Cyclic voltammetry of redox active species* ***2c*** *(blue), and after adding 5 equiv of MeOH (orange). Conditions:* ***2c*** *2.5 mM (100 mV/s) in MeCN, TBAPF$_6$ 0.1 M, rt. Start at 0.0 V, scan direction to negative potentials. Glassy carbon disk as working electrode, platinum wire as auxiliary electrode and SCE as reference electrode. IUPAC plotting.*

Cyclic voltammetry experiments of compound **2d** and a mixture of **2d** with 5 equiv of MeOH were conducted to evaluate changes in electrochemical properties. An apparition of an additional wave with a cathodic shift was observed (Figure S10).

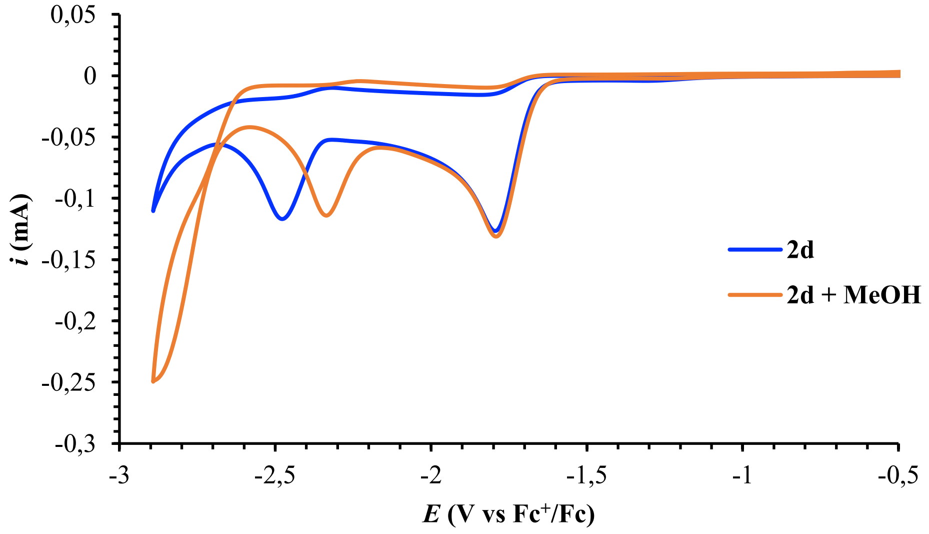

Figure S10 *Cyclic voltammetry of redox active species* ***2d*** *(blue), and after adding 5 equiv of MeOH (orange). Conditions:* ***2d*** *2.5 mM (100 mV/s) in MeCN, TBAPF$_6$ 0.1 M, rt. Start at 0.0 V, scan direction to negative potentials. Glassy carbon disk as working electrode, platinum wire as auxiliary electrode and SCE as reference electrode. IUPAC plotting.*

Cyclic voltammetry experiments of non-fluorous **2e** and its mixture with 5 equiv of MeOH revealed a small anodic shift (Figure S11).

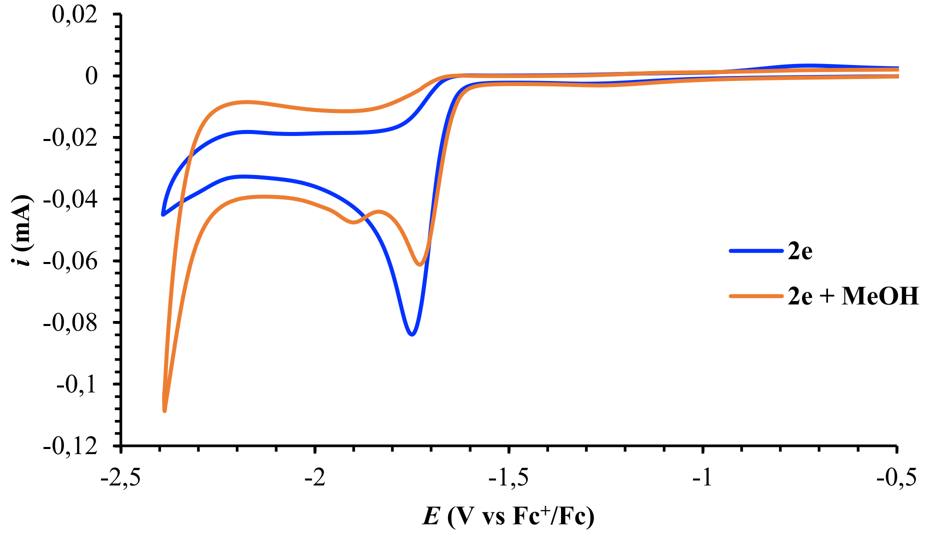

Figure S11 *Cyclic voltammetry of redox active species* ***2e*** *(blue) and after adding 5 equiv of MeOH (orange). Conditions:* ***2e*** *2.5 mM (100 mV/s) in MeCN, TBAPF$_6$ 0.1 M, rt. Start at 0.0 V, scan direction to negative potentials. Glassy carbon disk as working electrode, platinum wire as auxiliary electrode and SCE as reference electrode. IUPAC plotting.*

Cyclic voltammetry experiments of non-fluorous **2f** and its mixture with 5 equiv of MeOH revealed a small anodic shift (Figure S12).

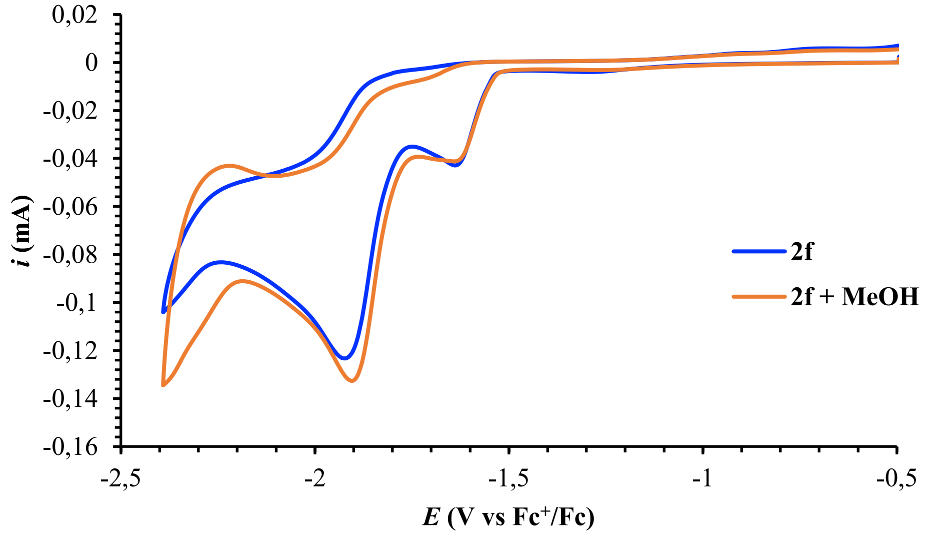

Figure S12 *Cyclic voltammetry of redox active species* ***2f*** *(blue) and after adding 5 equiv of MeOH (orange). Conditions:* ***2f*** *2.5 mM (100 mV/s) in MeCN, TBAPF$_6$ 0.1 M, rt. Start at 0.0 V, scan direction to negative potentials. Glassy carbon disk as working electrode, platinum wire as auxiliary electrode and SCE as reference electrode. IUPAC plotting.*

Cyclic voltammetry experiments of non-fluorous **2g** and its mixture with 5 equiv of MeOH revealed a small anodic shift (Figure S13).

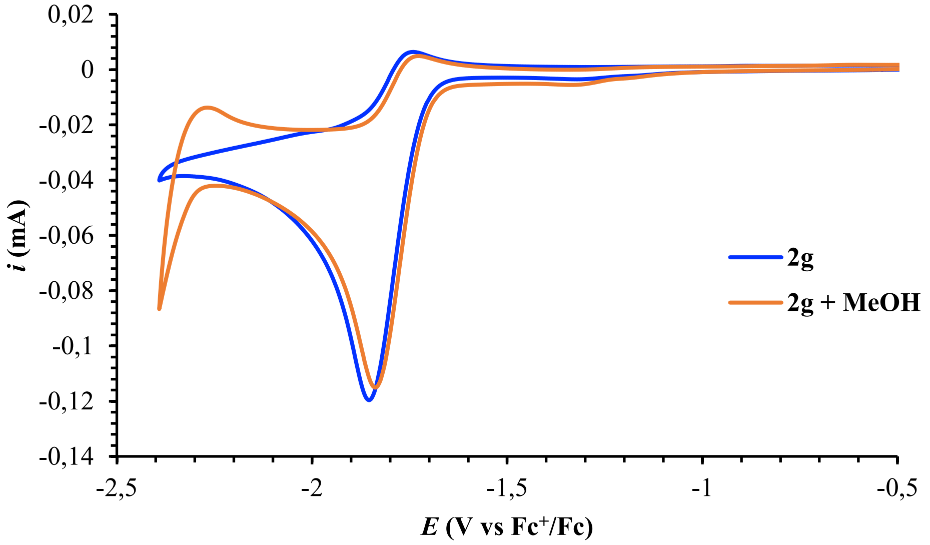

***Figure S13** Cyclic voltammetry of redox active species **2g** (blue) and after adding 5 equiv of MeOH (orange). Conditions: **2g** 2.5 mM (100 mV/s) in MeCN, $TBAPF_6$ 0.1 M, rt. Start at 0.0 V, scan direction to negative potentials. Glassy carbon disk as working electrode, platinum wire as auxiliary electrode and SCE as reference electrode. IUPAC plotting.*

***Table S2** Collected redox data of the presented work relevant compounds.*

Compound	$E_{1/2}$ (P^+/P)	$E_{1/2}$ (P/P^-)	E_a	E_c	E (P^+/*P)	E (*P/P^-)
4DPAIPN	0.66	-2.07	-	-	-1.96	0.55
1c	-	-	1.29	-	-	-
2a	-	-	-	-1.63	-	-
2b	-	-	-	-1.67	-	-
2c	-	-	-	-1.63	-	-
2d	-	-	-	-1.72	-	-
2e	-	-	-	-1.69	-	-
2f	-	-	-	-1.59	-	-
2g				-1.76		

All values are given in V *vs* Fc^+/Fc. For those that presented irreversible waves, E_a (oxidation) or E_c (reduction) are extracted as an approximation of the thermodynamic $E_{1/2}$.

Calculus for the Excited State potentials were performed with the following equation:[13]

1) $E_{1/2}(P^+/{}^*P) = E_{1/2}(P^+/P) - E_{0\text{-}0}$
2) $E_{1/2}({}^*P/P^-) = E_{1/2}(P/P^-) + E_{0\text{-}0}$
3) E (eV) = 1.2398/λ (in µm), then $E_{0\text{-}0}$ is when $\lambda = (\lambda_{max,abs} + \lambda_{max,em}) / 2$ (*Rehem-Weller equation)*. $E_{0\text{-}0}$ = 2.62 eV used as described in previous reports.[14]

6.3. UV-Vis studies

A 0.1 M solution of redox active species **2a**, a 0.5 M solution of MeOH and a mixture 2:5 of **2a**/MeOH in DCM were prepared. UV-Vis absorption spectra were measured in a 1 cm quartz cuvette. Absorption spectra of individual reaction components and mixtures thereof were recorded. A slight bathochromic shift was observed for a mixture of **2a** and MeOH in DCM. This indicates the interaction between the two reagents.

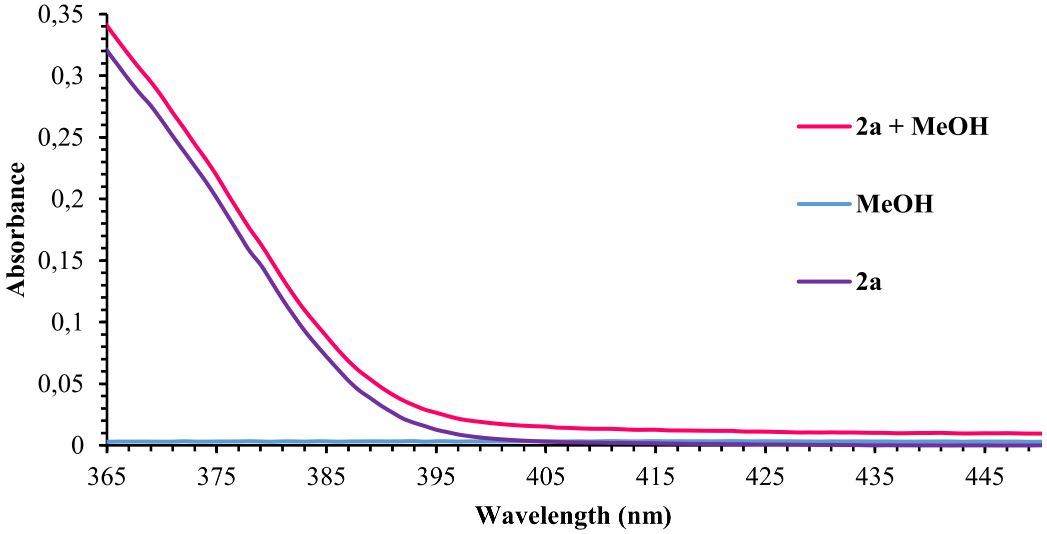

Figure S14 *UV-Vis absorption of individual components and a combination thereof. All spectra were measured in DCM and with a concentration of 0.2 M of redox active species* ***2a*** *and 0.5 M of MeOH. The stoichiometric and concentration of samples reflects the used reaction conditions.*

A 0.003 M solution of 4DPAIPN in DCM was prepared. UV-Vis absorption spectra were measured in a 1 cm quartz cuvette. Absorption spectra of the photocatalyst and mixtures with increasing equivalents of **2a** (1, 10, 20, 50, 100 and 150 equiv) were recorded. No clear red shift was observed while adding the redox compounds, thus indicating the null interaction with 4DPAIPN and discarding the formation of an EDA complex.

[13] Tucker, J. W.; Stephenson, C. R. *J. Org. Chem.* **2012**, *77*, 1617-1622.
[14] Singh, P. P.; Srivastava, V. *Org. Biomol. Chem.* **2021**, *19*, 313-321.

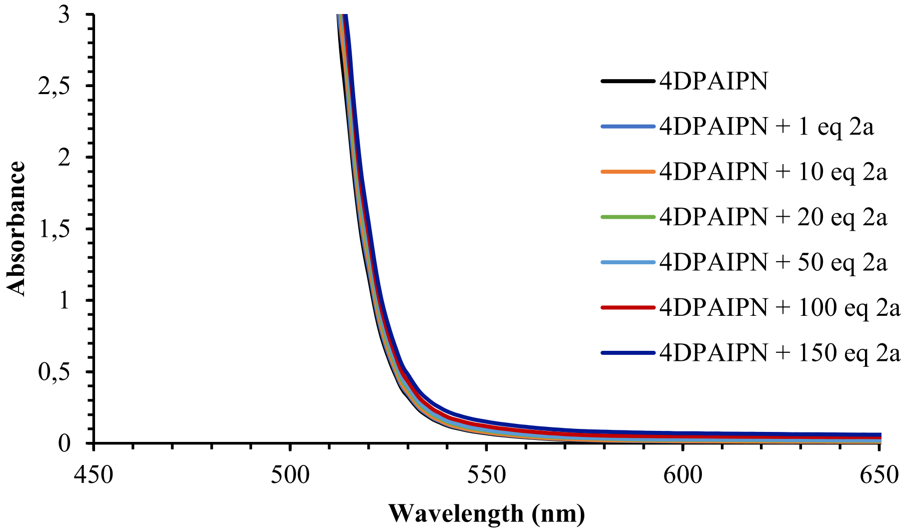

Figure S15 *UV-Vis absorption of 4DPAIPN and a combination thereof. All spectra were measured in DCM and with a concentration of 0.003 M 4DPAIPN. The stoichiometric when at 150 equivalents and concentration of samples reflects the used reaction conditions.*

6.4. Titration experiments

^{1}H-NMR and ^{19}F-NMR of mixtures of **2a** and MeOH in $CDCl_3$, were recorded at 298 K. In an NMR tube, the total volume of the mixture was 0.6 mL, the concentration of **2a** (0.06 mmol) was kept constant at 0.1 M, and that of MeOH was varied from 0.0 to 2.0 M. The signals of both ^{1}H-NMR and ^{19}F-NMR presented an upfield shift, demonstrating the interaction between reagent **2a** and MeOH.

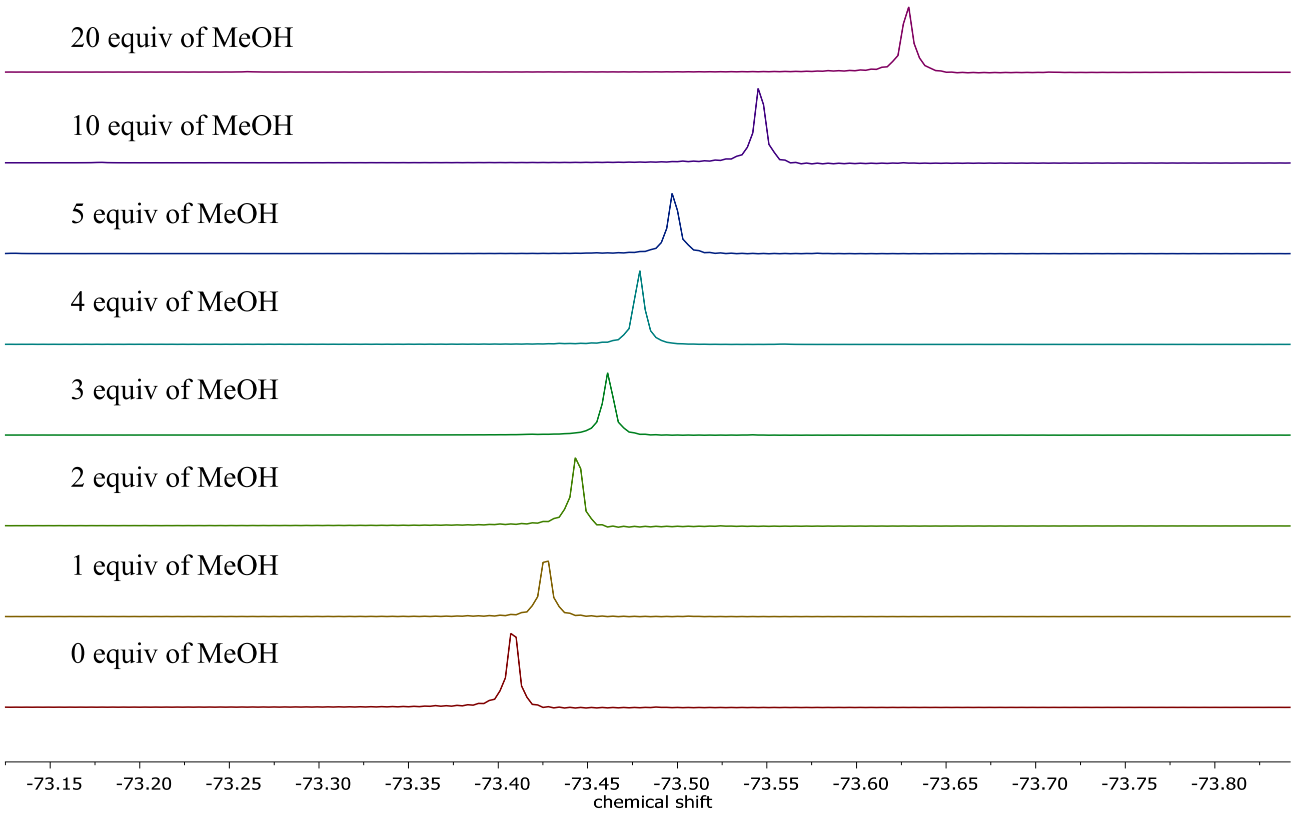

Figure S16 *^{19}F NMR shift of mixtures of* ***2a*** *with increasing amounts of MeOH (0, 1, 2, 3, 4, 5, 10, 20 equiv).*

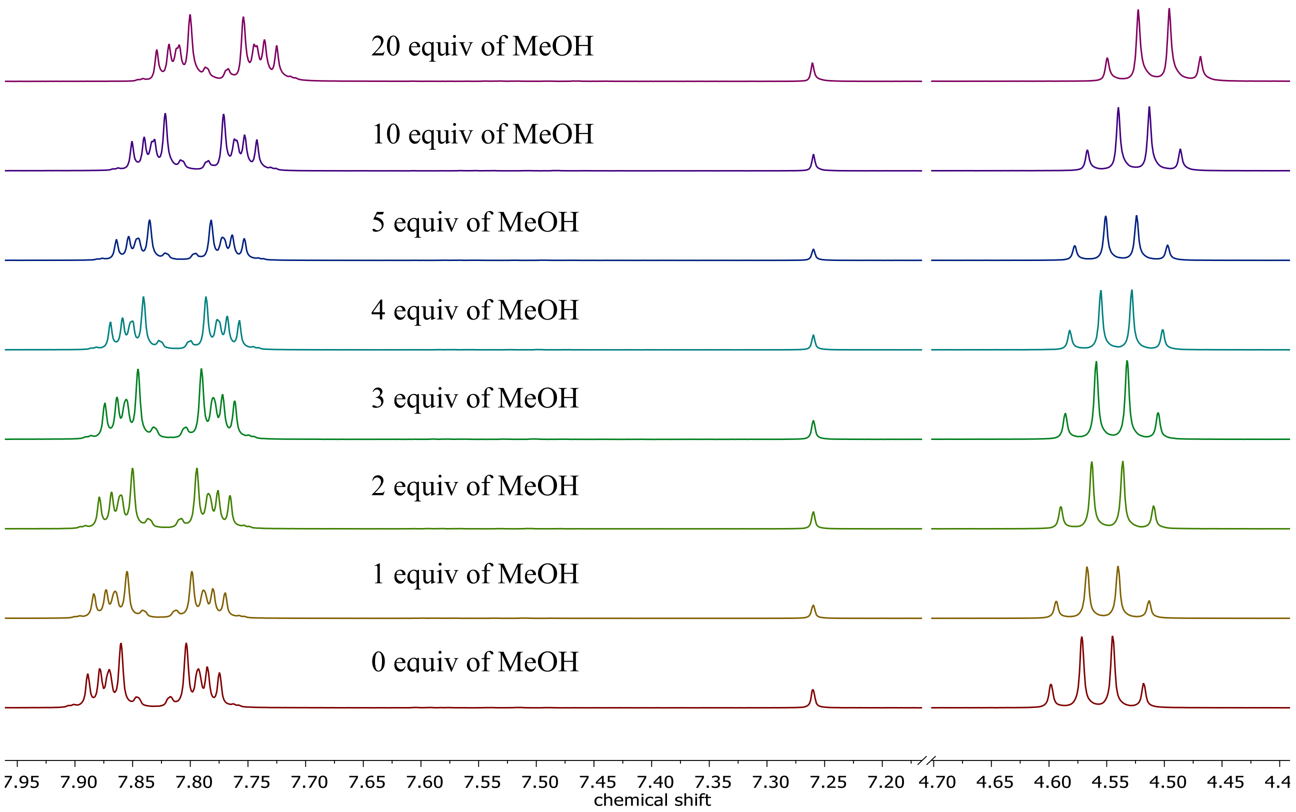

Figure S17 *^{1}H NMR (400 MHz, $CDCl_3$) shift of mixtures of **2a** with increasing amounts of MeOH (0, 1, 2, 3, 4, 5, 10, 20 equiv).*

^{1}H-NMR of mixtures of **2e** and MeOH in $CDCl_3$, were recorded at 298 K. In an NMR tube, the total volume of the mixture was 0.6 mL, the concentration of **2e** (0.06 mmol) was kept constant at 0.1 M, and MeOH was 0.0 and 2.0 M. The signals of ^{1}H-NMR presented an upfield shift, demonstrating the interaction between reagent **2e** and MeOH, likely through the nitrile group.

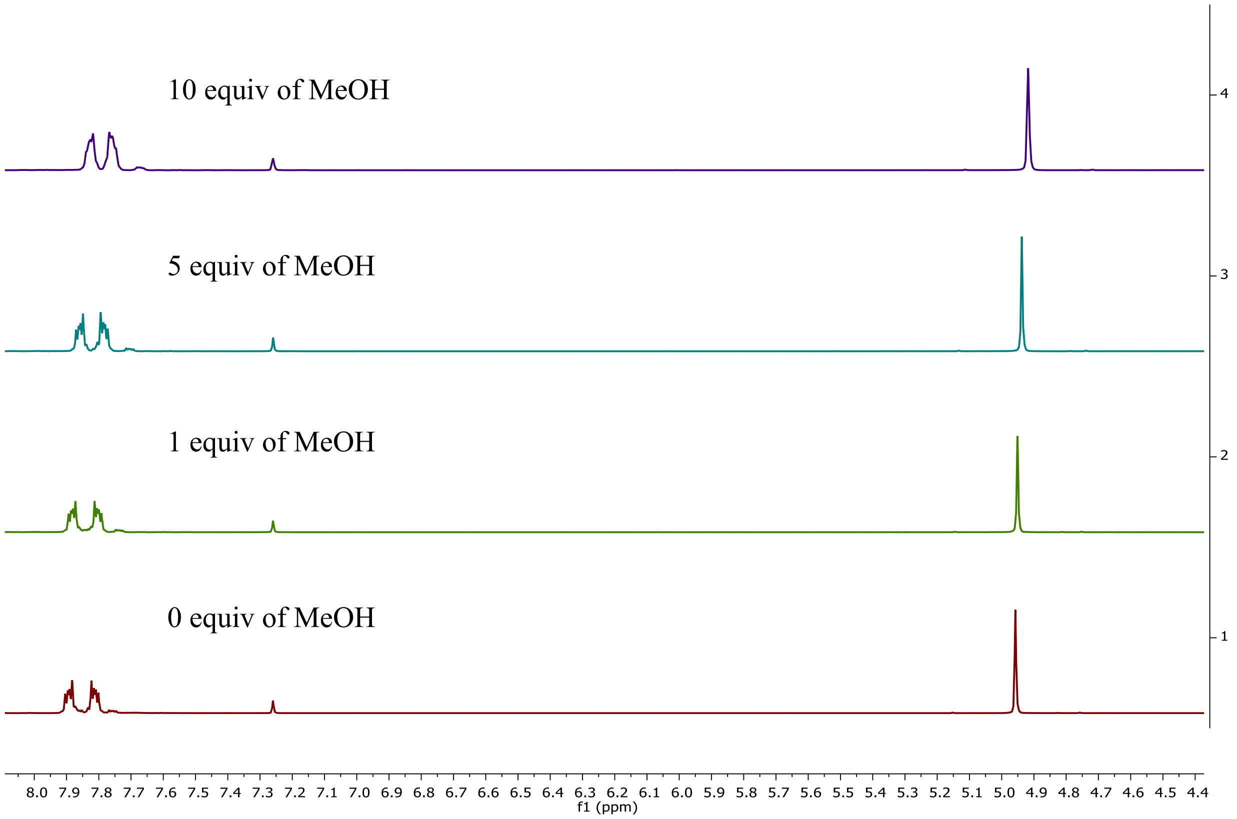

Figure S18 *^{1}H NMR shift of mixtures of* ***2e*** *with 0, 1, 5 and 10 equiv of MeOH.*

^{1}H-NMR of mixtures of **2g** and MeOH in $CDCl_3$, were recorded at 298 K. In an NMR tube, the total volume of the mixture was 0.6 mL, the concentration of **2g** (0.06 mmol) was kept constant at 0.1 M, and MeOH was 0.0 and 2.0 M. The signal of ^{1}H-NMR presented no clear shift, showing no interaction between reagent **2f** and MeOH.

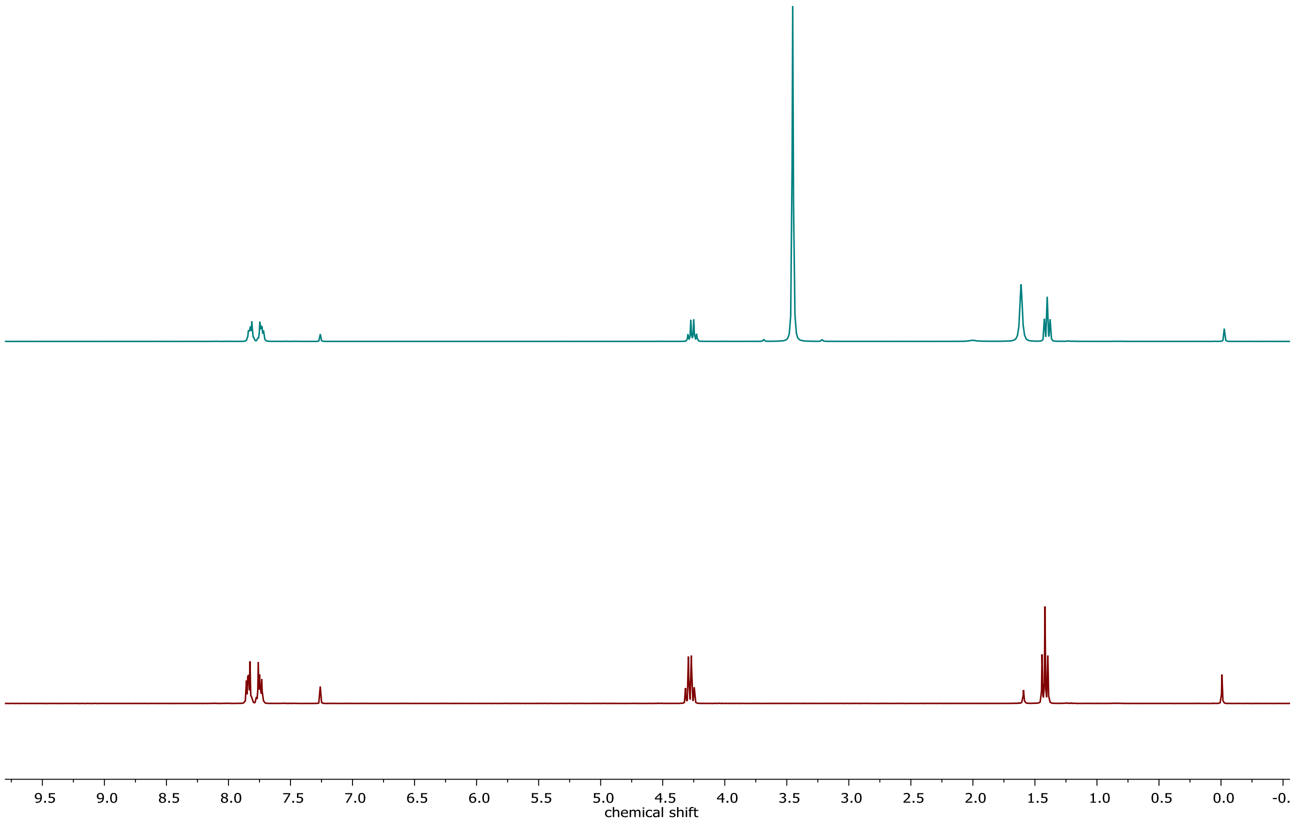

Figure S19 *^{1}H NMR shift of mixtures of **2g** with 0 and 10 equiv of MeOH.*

6.5. *Stern-Volmer quenching studies*

Fluorescence measurements were obtained using septa-capped UV-Quartz cuvettes (10 mm pathlength) obtained from Hellma Analytics. Excitation was performed at 400 nm; fluorescence spectra were obtained from 300-700 nm. The stock solutions were prepared as follows:

1. Photocatalyst 4DPAIPN solution (0.000002 M): To a 25 mL volumetric flask was added 3.90 mg of 4DPAIPN and was dissolved in DCM. Subsequently, 100 µL of the previous solution was added to a 10 mL volumetric flask and was diluted with DCM obtaining a 2.00×10^{-5} M solution of 4DPAIPN.
2. Styrene **1c** solution (0.004 M): To a 10 mL volumetric flask was added 5.4 mg of **1c** and was dissolved in DCM obtaining a 4.00×10^{-3} M solution of **1c**.

3. Redox active specie **2a** solution (0.004 M): To a 10 mL volumetric flask was added 10.3 mg of **2a** and was dissolved in DCM obtaining a $4.00x10^{-3}$ M solution of **2a**.
4. Redox active specie **2a** + MeOH solution (0.004 M): To a 10 mL volumetric flask was added 10.3 mg of **2a** and 4 μL of MeOH and was dissolved in DCM obtaining a $4.00x10^{-3}$ M solution of **2a** with 2.5 equiv of MeOH (reaction equiv).
5. Redox active specie **2g** solution (0.004 M): To a 10 mL volumetric flask was added 7.6 mg of **2f** and was dissolved in DCM obtaining a $4.00x10^{-3}$ M solution of **2a**.
6. Redox active species **2g** + MeOH solution (0.004 M): To a 10 mL volumetric flask was added 7.6 mg of **2f** and 4 μL of MeOH and was dissolved in DCM obtaining a $4.00x10^{-3}$ M solution of **2a** with 2.5 equiv of MeOH (reaction equiv).

Following preparation, the solutions were allocated to the cuvettes and fluorescence quenching was determined with individual quencher mixtures. 1 mL of the Photocatalyst solution was added together with 1 mL of DCM for the initial measurement. For the experiments with quencher, 1 mL of Photocatalyst solution was added together with increasing amounts of quencher (0, 10, 100, 200, 500 and 1000 μL as indicated) and adjusting concentration with volume of DCM to reach 2 mL. Degassing of each individual solution for 20 seconds was performed prior to recording the data. Linear regression of I_0/I against concentration was carried out to yield the Stern-Volmer quenching rate constant (K_{SV}). The following Stern-Volmer plots for luminescence quenching of 4DPAIPN (2.0×10^{-5} M in degassed DCM) by quenchers were obtained. The excited catalyst is only quenched by the *redox active specie* **2a** with a Stern-Volmer quenching rate constant of 93.2 M^{-1} for Solution 3 and 122.4 M^{-1} for Solution 4, demonstrating a better quenching by the MeOH presence.

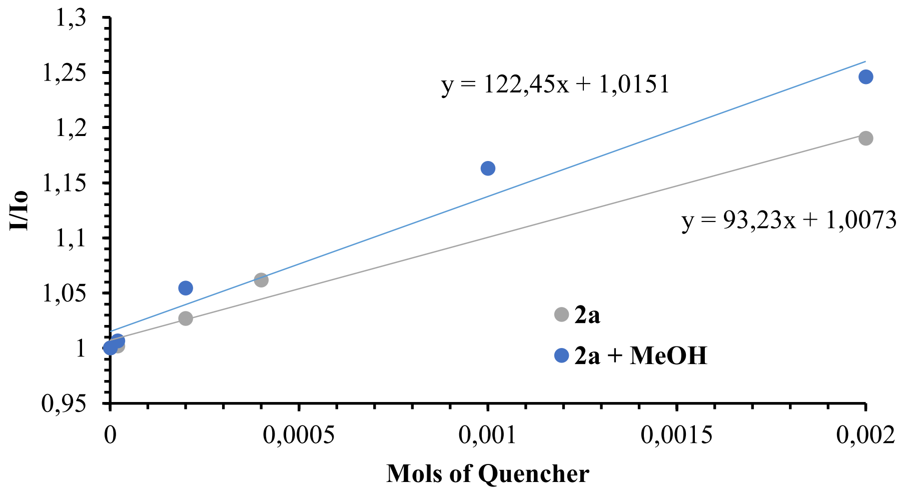

***Figure S20** Stern-Volmer plots for luminescence quenching of 4DPAIPN ($2.0 \ x \ 10^{-5}$ M in degassed DCM) by Solution 3 (grey) and Solution 4 (blue), $\lambda_{exc.}$ = 400 nm, $\lambda_{em.}$ = 530 nm, K_{sv} = Stern-Volmer constant.*

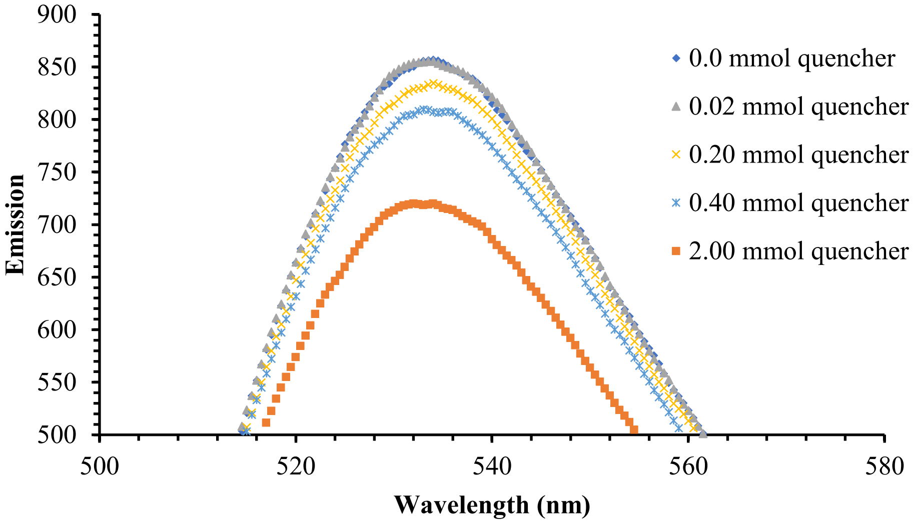

Figure S21 *Raw data for luminescence quenching of 4DPAIPN (2.0 x 10^{-5} M in degassed DCM) by Solution 3, $\lambda_{exc.}$ = 400 nm, $\lambda_{em.}$ = 530 nm.*

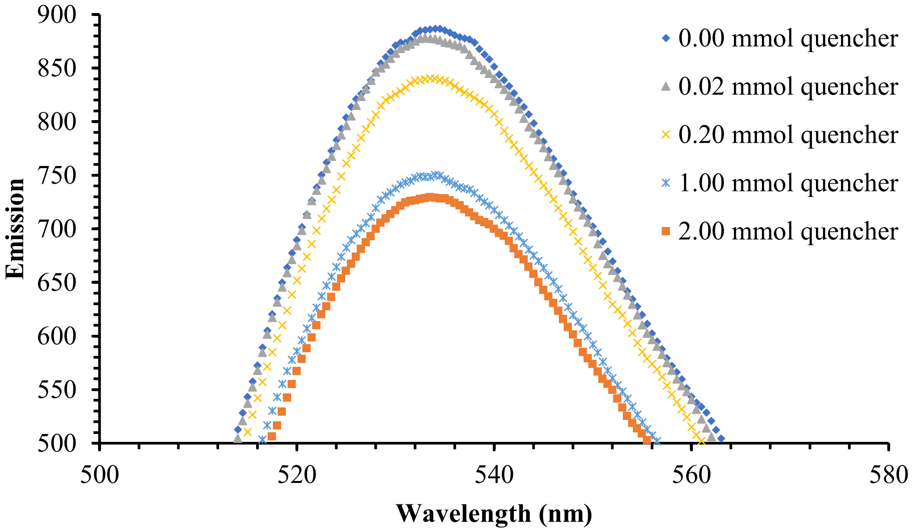

Figure S22 *Raw data for luminescence quenching of 4DPAIPN (2.0 x 10^{-5} M in degassed DCM) by Solution 4, $\lambda_{exc.}$ = 400 nm, $\lambda_{em.}$ = 530 nm.*

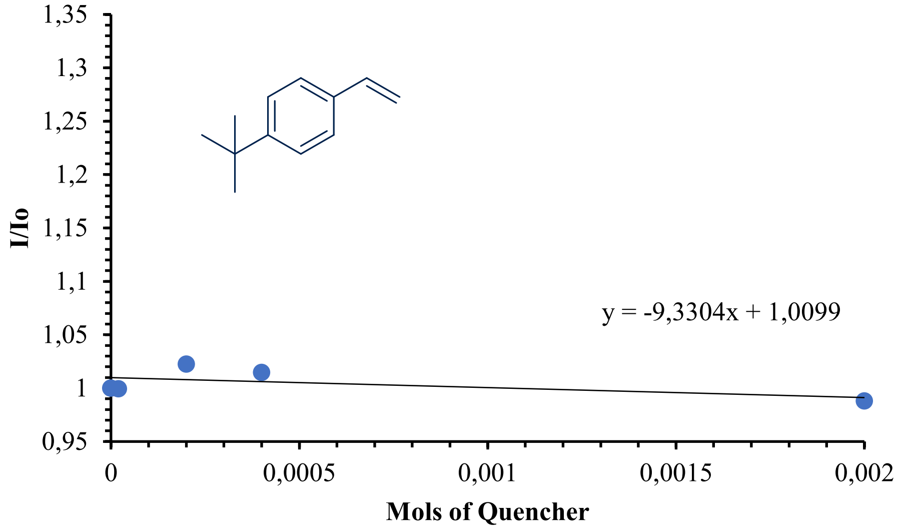

Figure S23 *Stern-Volmer plots for luminescence quenching of 4DPAIPN (2.0 x 10^{-5} M in degassed DCM) by Solution 2, $\lambda_{exc.}$ = 400 nm, $\lambda_{em.}$ = 530 nm, K_{sv} = Stern-Volmer constant.*

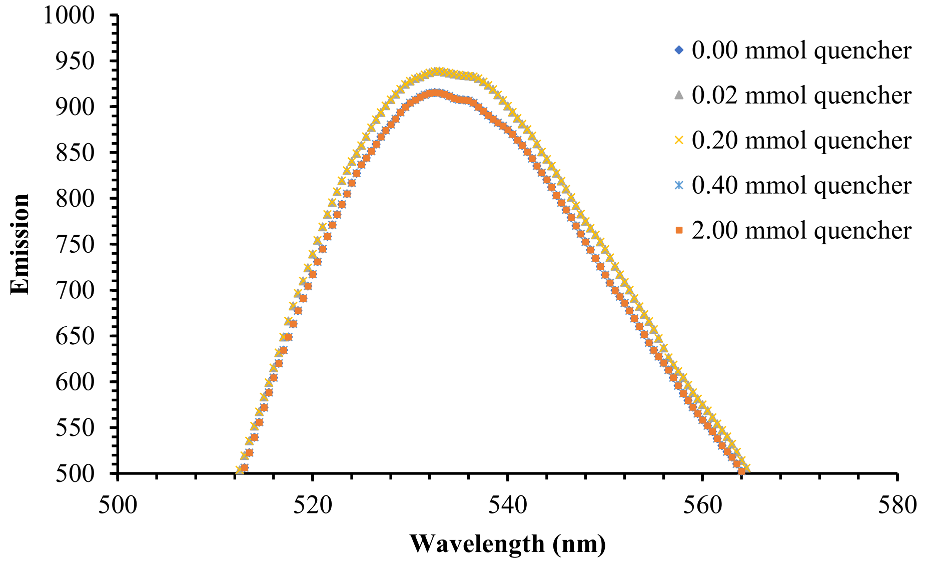

Figure S24 *Raw data for luminescence quenching of 4DPAIPN (2.0 x 10^{-5} M in degassed DCM) by Solution 2, $\lambda_{exc.}$ = 400 nm, $\lambda_{em.}$ = 530 nm.*

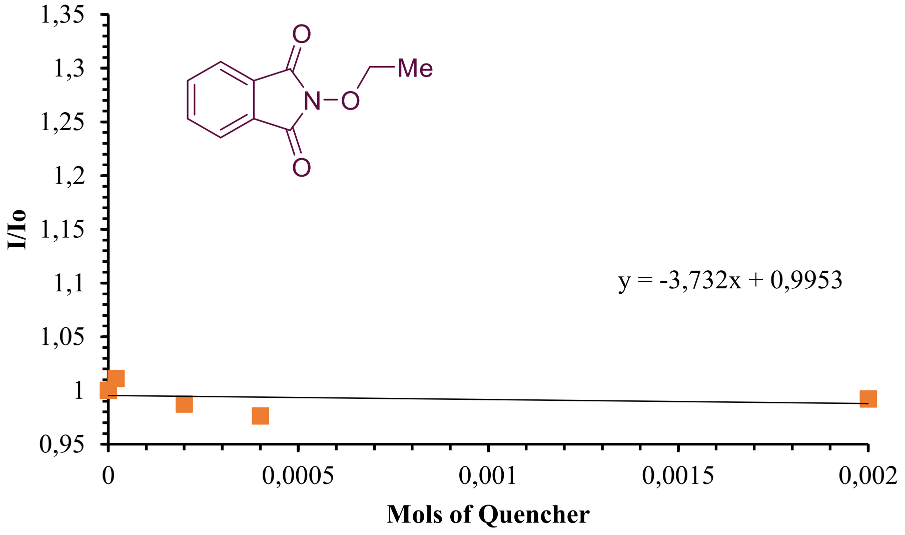

Figure S25 *Stern-Volmer plots for luminescence quenching of 4DPAIPN (2.0 x 10^{-5} M in degassed DCM) by Solution 5, $\lambda_{exc.}$ = 400 nm, $\lambda_{em.}$ = 530 nm, K_{sv} = Stern-Volmer constant.*

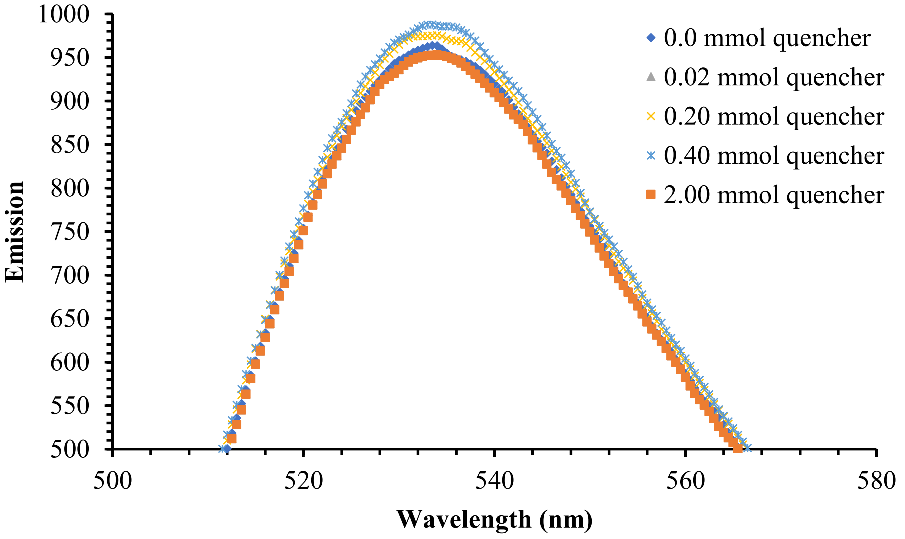

Figure S26 *Raw data for luminescence quenching of 4DPAIPN (2.0 x 10^{-5} M in degassed DCM) by Solution 5, $\lambda_{exc.}$ = 400 nm, $\lambda_{em.}$ = 530 nm.*

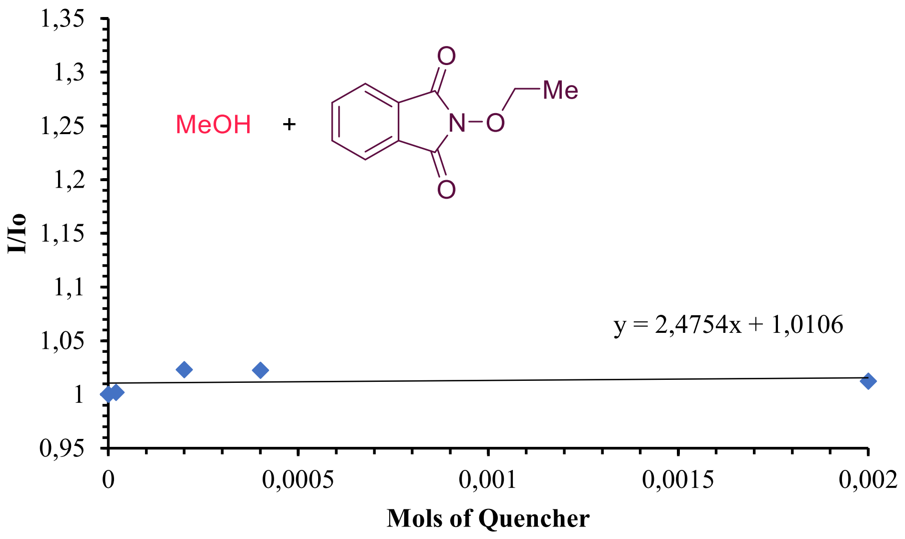

Figure S27 *Stern-Volmer plots for luminescence quenching of 4DPAIPN (2.0 x 10*$^{-5}$ *M in degassed DCM) by Solution 6,* $\lambda_{exc.}$ *= 400 nm,* $\lambda_{em.}$ *= 530 nm,* K_{sv} *= Stern-Volmer constant.*

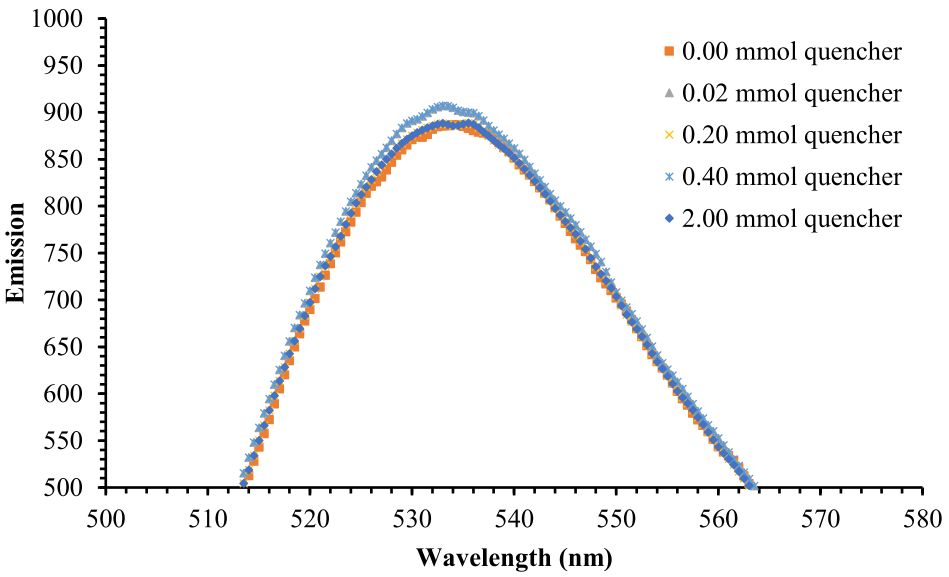

Figure S28 *Raw data for luminescence quenching of 4DPAIPN (2.0 x 10*$^{-5}$ *M in degassed DCM) by Solution 6,* $\lambda_{exc.}$ *= 400 nm,* $\lambda_{em.}$ *= 530 nm.*

6.6. Photochemical Quantum Yield

The quantum yield for our model photochemical reaction was determined by using previously reported methods using equation 1.[15] Styrene **1a** and Redox active species **2a** were used as model substrates to determine the photochemical quantum yield of the transformation, using 1,3,5-trimethoxybenzene as internal standard in a 1:1 ratio with **1a**.

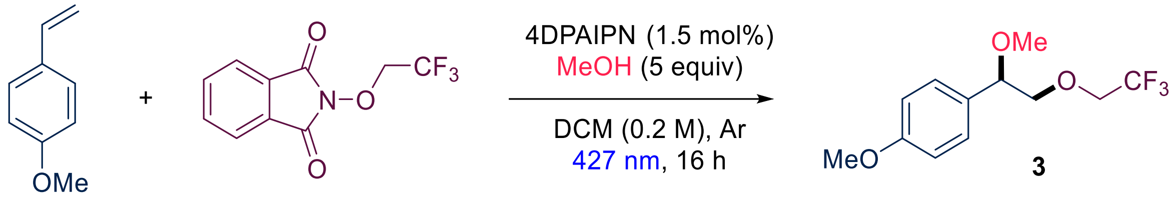

$$\Phi \text{ (reaction at 427 nm)} = \frac{\text{mol of formed product}}{\text{mol of photon flux} \cdot \text{t} \cdot \text{f}} \qquad (1)$$

where Φ is the quantum yield of the reaction, t is the time of the reaction (s), f is the incident light absorbed by the organophotocatalyst at 427 nm and the photon flux is calculated by standard ferrioxalate actinometry.

Incident of light absorbed by the organophotocatalyst

The fraction of light, f, absorbed was determined according to equation 2:

$$\text{f} = 1 - 10^{-\text{A}} \qquad (2)$$

where A is the absorbance of the organophotocatalyst in DCM at 427 nm. The wavelength of 427 nm was chosen based on the known absolute $\Phi(Fe^{+2})$ value[16] and is the wavelength we are using in our reaction. The absorbance of the mixture was recorded. The absorbance (A) at 427 nm was determined to be >2, thus indicating the fraction of light absorbed is 0.99 according to equation 2.

[15] a) El Khatib, M. Serafim, ; R. A. M.; Molander, G. A. *Angew. Chem. Int. Ed.*, **2016**, *55*, 254.
b) Cismesia, M. T.; Yoon, T. P. *Chem. Sci.*, **2015**, *6*, 5426.

[16] He, G.-C.; Guo, S.-Y.; Zheng, H.; Liu, C.-H.; Li, Y.M Min, X.-T. Ji, D. W.; Q.-A. Chen, *Cell Rep. Phys. Sci.* **2022**, *3*, 100768.

The photochemical reaction

The reaction was developed using the general procedure for 90 min. Subsequently, 1,3,5-trimethoxybenzene was added as internal standard, and the solvent was evaporated. The yield of the reaction was determined by ^{1}H NMR, where 0.028 mmol (28%) of the desired product were obtained.

Photon flux sample calculation

Standard ferrioxalate actinometry was used to determine the photon flux of the spectrophotometer using equations 3 and 4. For the ferrioxalate actinometer the production of Fe^{+2} ions proceed by the following reactions:

$$[Fe(C_2O_4)n]^{+(3-2n)} \xrightarrow{\text{light}} Fe^{+2} + (n-1)(C_2O_4)^{-2} + C_2O_4^{-}$$

$$[Fe(C_2O_4)n]^{+(3-2n)} + C_2O_4^{-} \longrightarrow Fe^{+2} + n(C_2O_4)^{-2} + 2\,CO_2$$

The moles of Fe^{+2} formed are determined spectrophotometrically by development with 1,10-phenanthroline (phen) to form the red $[Fe(phen)_3]^{+2}$ moiety (λ = 510 nm).[17] The photon flux is defined as shown in equation 3:

$$\text{Photon Flux} = \frac{\text{mol Fe}^{+2}}{\Phi(\text{Fe}^{+2}) \cdot \text{t} \cdot \text{f}} \qquad (3)$$

Where Φ is the quantum yield for the ferrioxalate actinometer (1.01 at λ = 438 nm), t is the time (s), and f ~1, and the mols of Fe^{+2} are calculated according to equation 4.

$$\text{mol (Fe}^{+2}) = \frac{\text{V} \cdot \Delta\text{A}}{\text{l} \cdot \epsilon} \qquad (4)$$

Where V is the total volume of the solution, ΔA is the difference in absorbance between irradiated and nonirradiated solutions, l is the path length (1.0 cm), ε is the molar absorptivity at 510 nm (11110 L mol^{-1}cm^{-1}).

The following solutions were prepared in the dark (flasks were wrapped in aluminum foil) and stored in the dark at room temperature:

[17] Demas, J. N.; Bowman, W. D.; Zalewski, E. F.; Velapoldi, R. *J. Phys. Chem.* **1981**, *85*, 2766.

– Ferrioxalate solution (0.15 M): Ammonium ferrioxalate hydrate (1.605 g) was added to a flask wrapped in aluminum foil containing H_2SO_4 (25 mL, 0.05 M). The flask was stirred for complete dissolution of the green solid in complete darkness. It is noteworthy that the solution should not be exposed to any incident light.

– Developer solution: 1,10-Phenanthroline (50 mg) and sodium acetate (11.26 g) were added to a flask containing H_2SO_4 (50 mL, 0.5 M) and sonicated until completely dissolved.

The absorbance of the non-irradiated sample. The buffered solution of phen (350 μL) was added to a ferrioxalate solution (2.0 mL) in a vial that had been covered with aluminum foil and with the lights of the laboratory switched off. The vial was capped and allowed to rest for 30 min and then transferred to a cuvette. The absorbance of the non-irradiated sample was measured at 510 nm to be 0.03.

The absorbance of the irradiated sample

In a cuvette equipped with a stir bar was added the ferrioxalate solution (2.0 mL), and the stirred solution was irradiated for different times at λ = 427 nm with an excitation slit width = 10.0 nm. After irradiation, the buffered phen solution (350 μL) was added to the cuvette and allowed to rest for 60 min in the dark to allow the ferrous ions to coordinate completely to phen. The absorbance of the samples was measured at 510 nm and the mols of Fe^{2+}calculated using the equation (4).

$$\text{mol}\ (\text{Fe}^{+2}) = \frac{\text{V} \cdot \Delta\text{A}}{\text{l} \cdot \epsilon} \qquad (4)$$

Time (s)	10	16	20	32	48
[Fe(II)]	1,18x10^{-7}	2,37x10^{-7}	2,52x10^{-7}	3,77x10^{-7}	5,65x10^{-7}

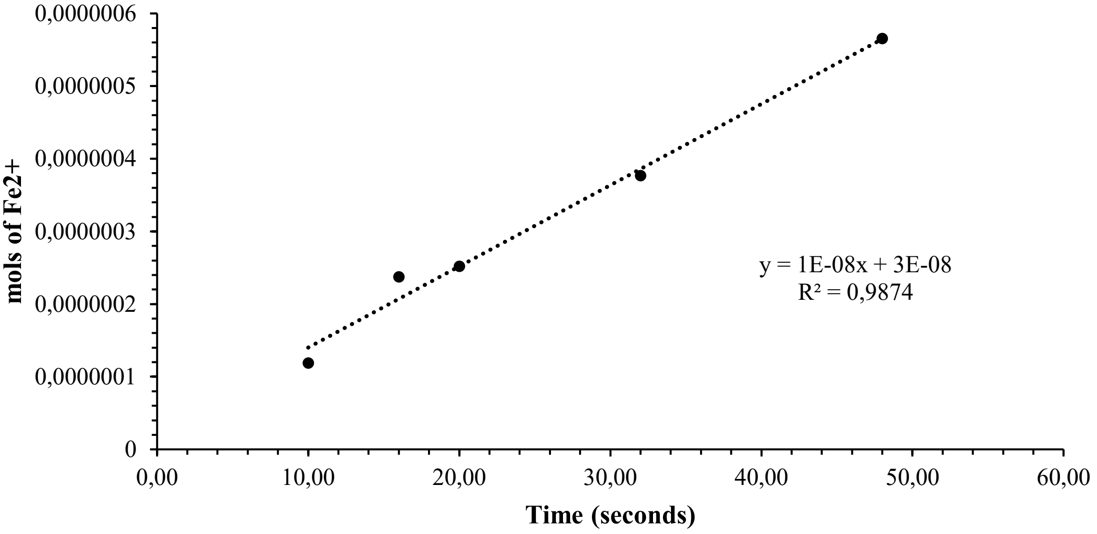

Photon flux sample calculation:

$$\text{Photon flux} = \frac{\text{dx/dt}}{\Phi(\text{Fe}^{+2}) \cdot \text{f}} \qquad (3)$$

$$\text{Photon flux} = \frac{1.0\text{x}10^{-8}\text{mol}}{1.01 \cdot 1} = 9.90\text{x}10^{-9}\ \text{einstein s}^{-1}$$

Quantum yield of the photoinduced transformation

Therefore, the quantum yield of the reaction was determined to be:

$$\Phi(\text{reaction at 427 nm}) = \frac{\text{mol of formed product}}{\text{mol of photon flux} \cdot \text{t} \cdot \text{f}} \qquad (1)$$

$$\boldsymbol{\Phi}(\textbf{reaction at 427 nm}) = \frac{2.9x10^{-5}\ \text{mol}}{9.90\text{x}10^{-7}\ \text{einstein s}^{-1} \cdot 5400\ \text{s} \cdot 0.99} = \mathbf{0.5}$$

6.7. Experiment using Ir(ppy)$_3$ with non-fluorinated ***2e, 2f*** *and* ***2g***

Additional mechanistic investigations were conducted employing the more reducing photocatalyst Ir(ppy)$_3$ to further elucidate the observed differences in reactivity among reagents **2a-2g**. Notably, the reaction with nitrile **2e** afforded the desired product, albeit in low yield (*Figure S29*), as previously noted with 4DPAIPN. Importantly, **2e** was also totally consumed as it happened with 4DPAIPN. This outcome suggests that while the reduction of **2e** is both thermodynamically and kinetically favored, the overall process is hindered by the reactivity of the ·OCH$_2$CN species. In addition, we observed a lack of reactivity with **2f** and with the alkyl-substituted derivative **2g**, which failed to furnish any product under same conditions (*Figures S30 and S31*). These findings support the hypothesis that the presence of a fluorine atom promotes a strong F···HOR interaction that facilitates the single-electron transfer (SET) event. In contrast, weaker interactions such as CO···HOR appear insufficient to drive efficient SET.

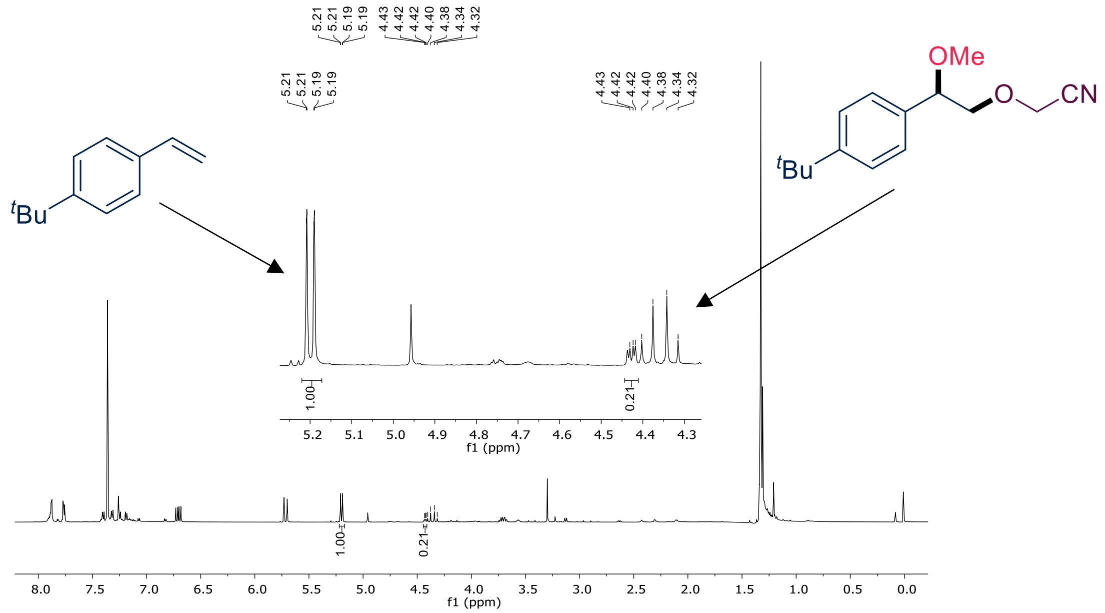

Figure S29

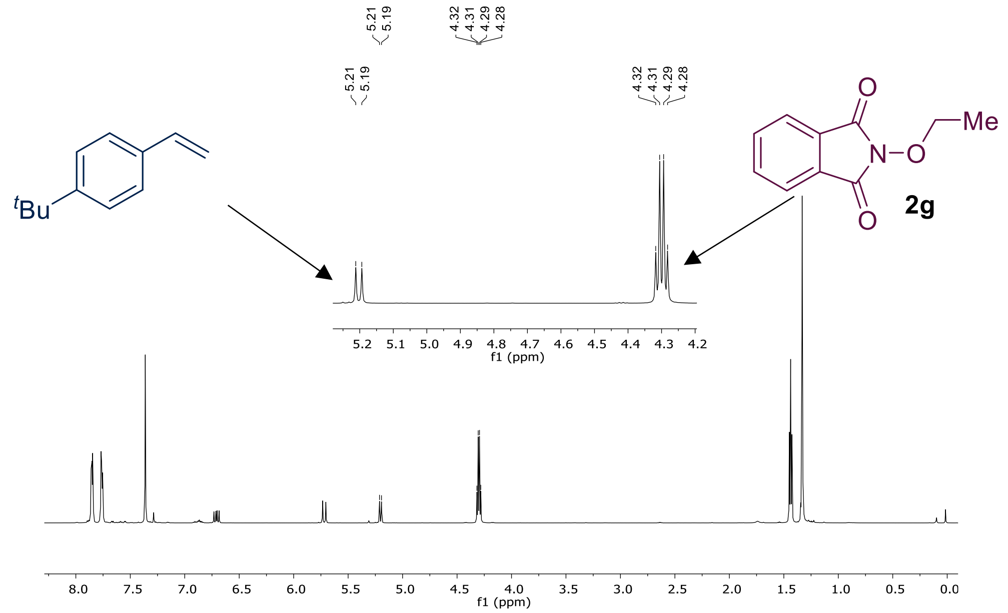

Figure S30

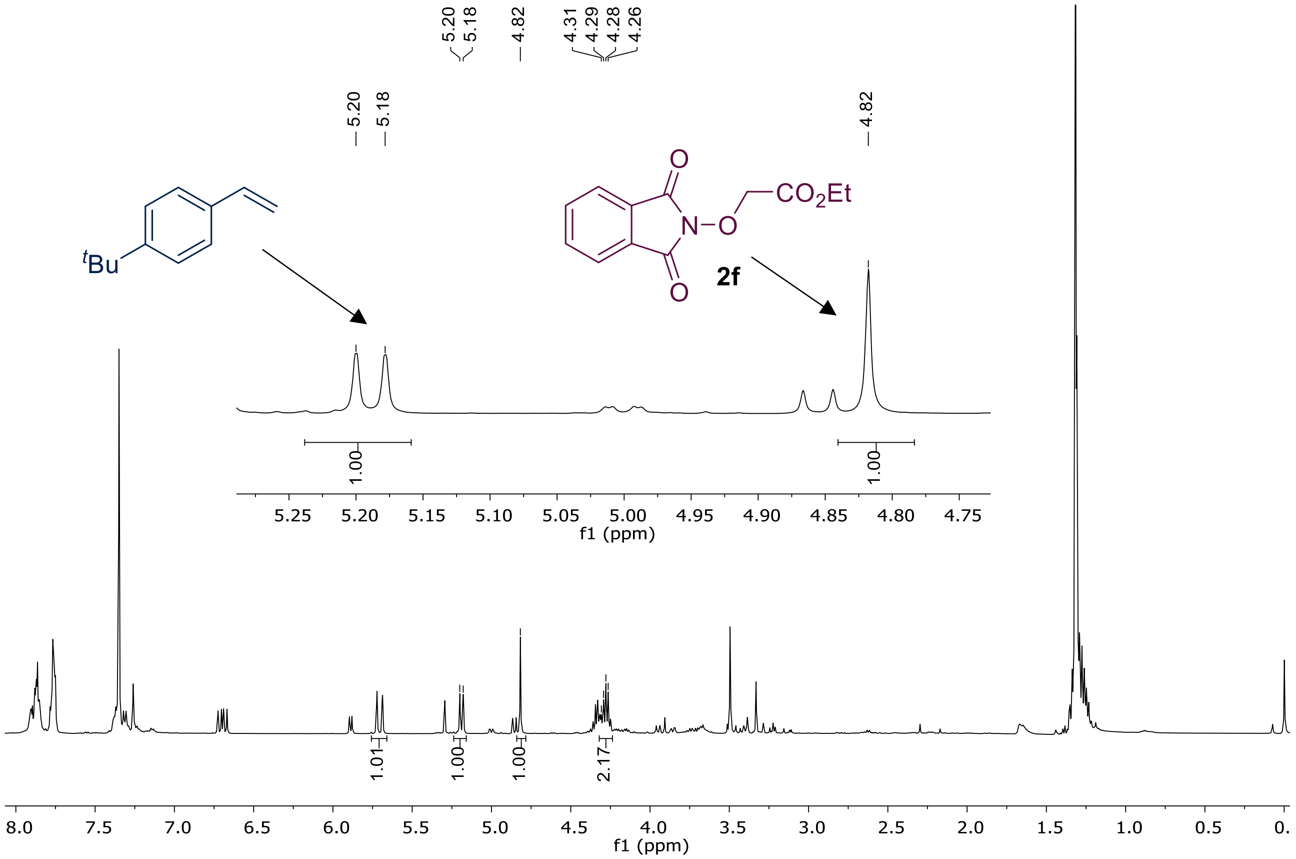

Figure S31

6.8. *Unsuccessful substrates*

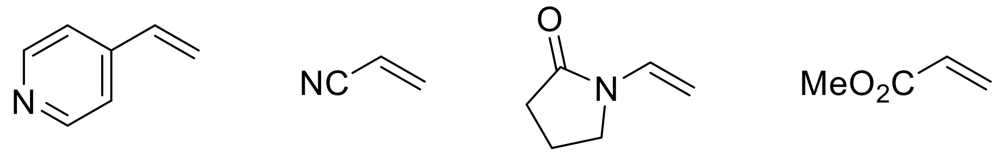

7. NMR Spectra

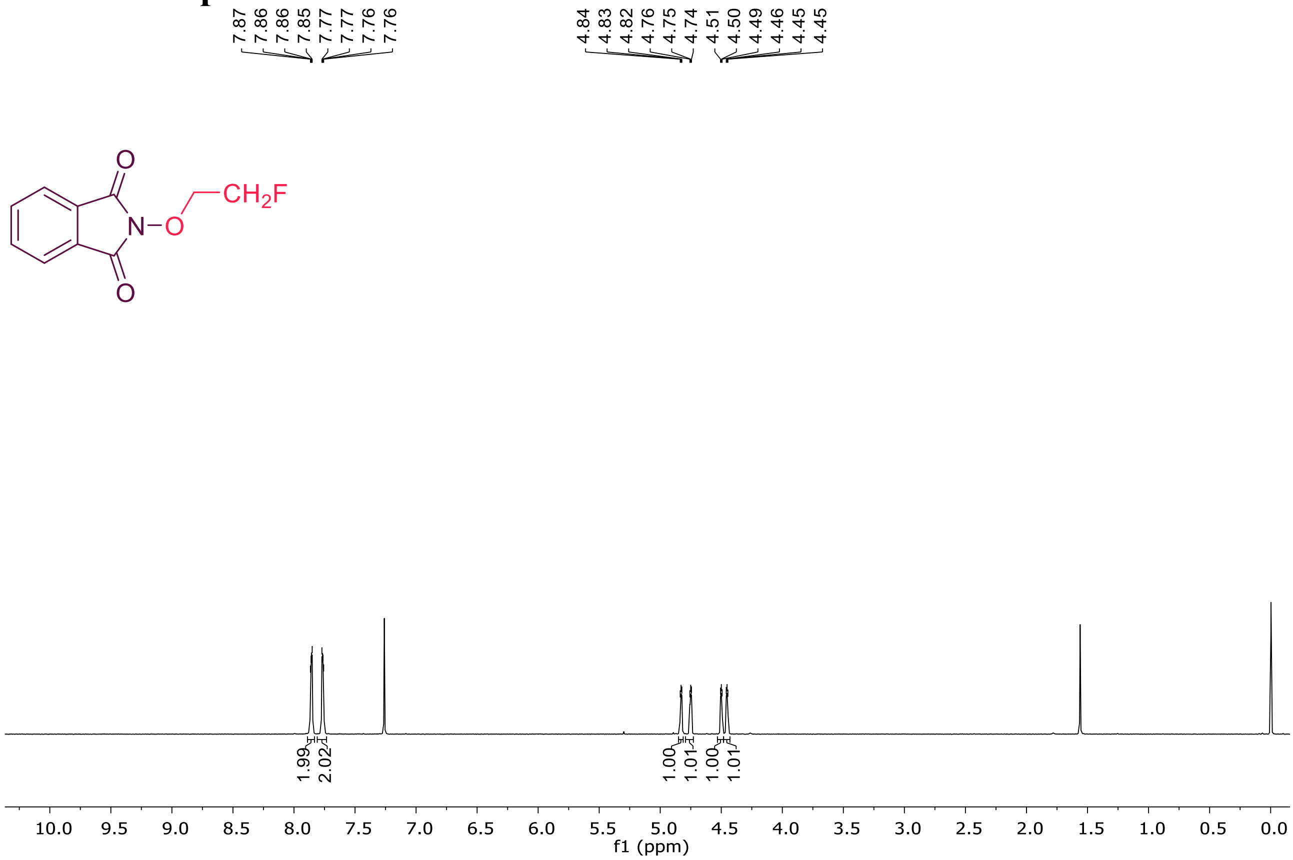

^{1}H NMR (600 MHz, $CDCl_3$) of compound **2d**.

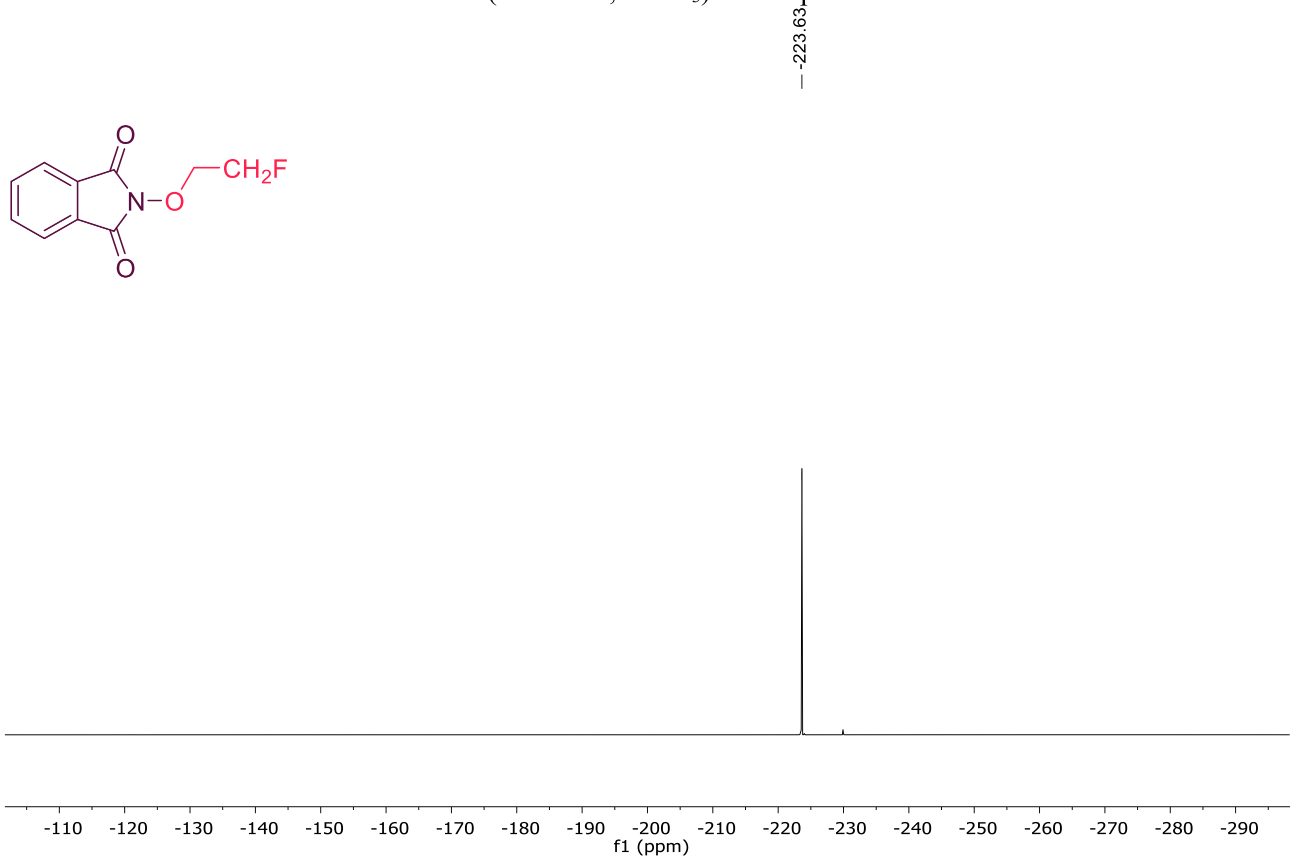

^{19}F NMR (282 MHz, $CDCl_3$) of compound **2d**.

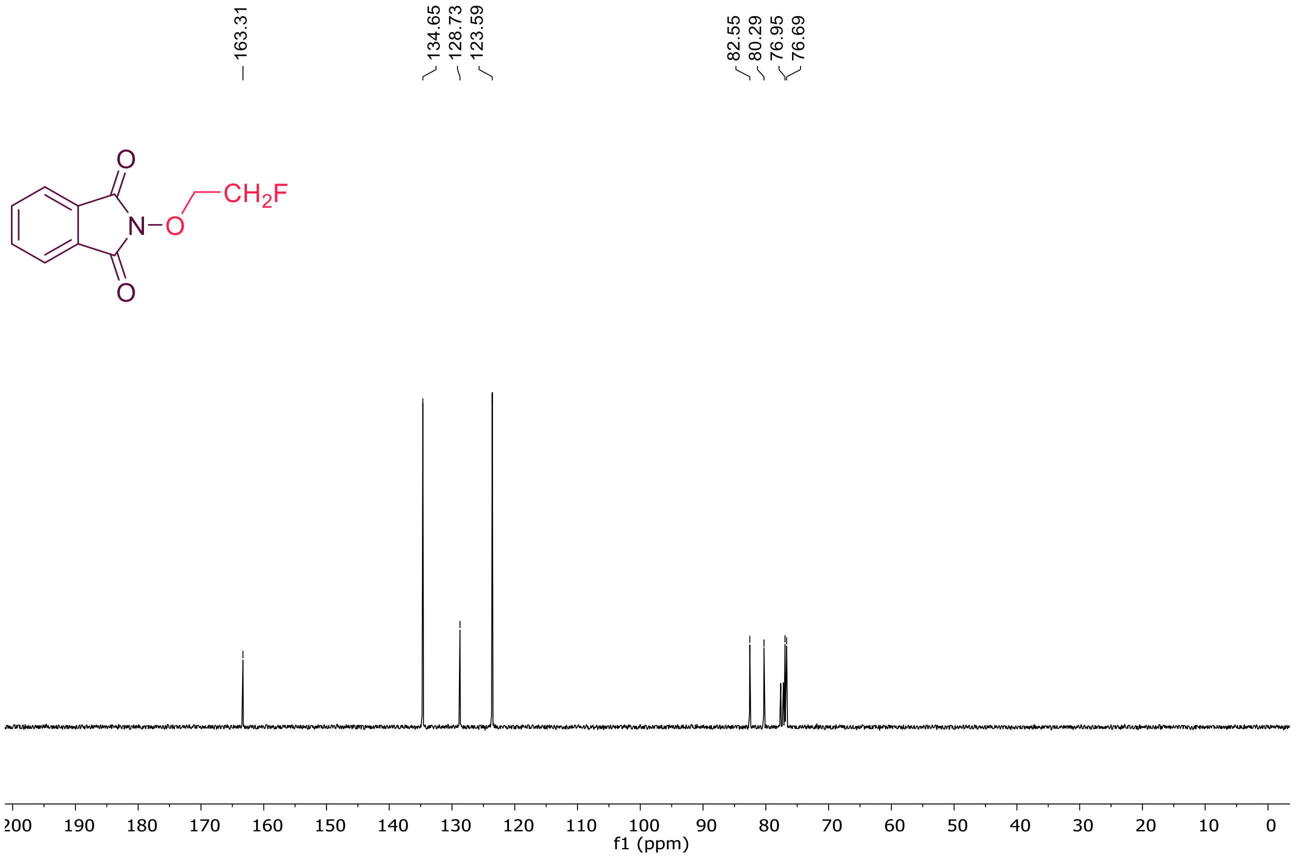

^{13}C NMR (75 MHz, $CDCl_3$) of compound **2d**.

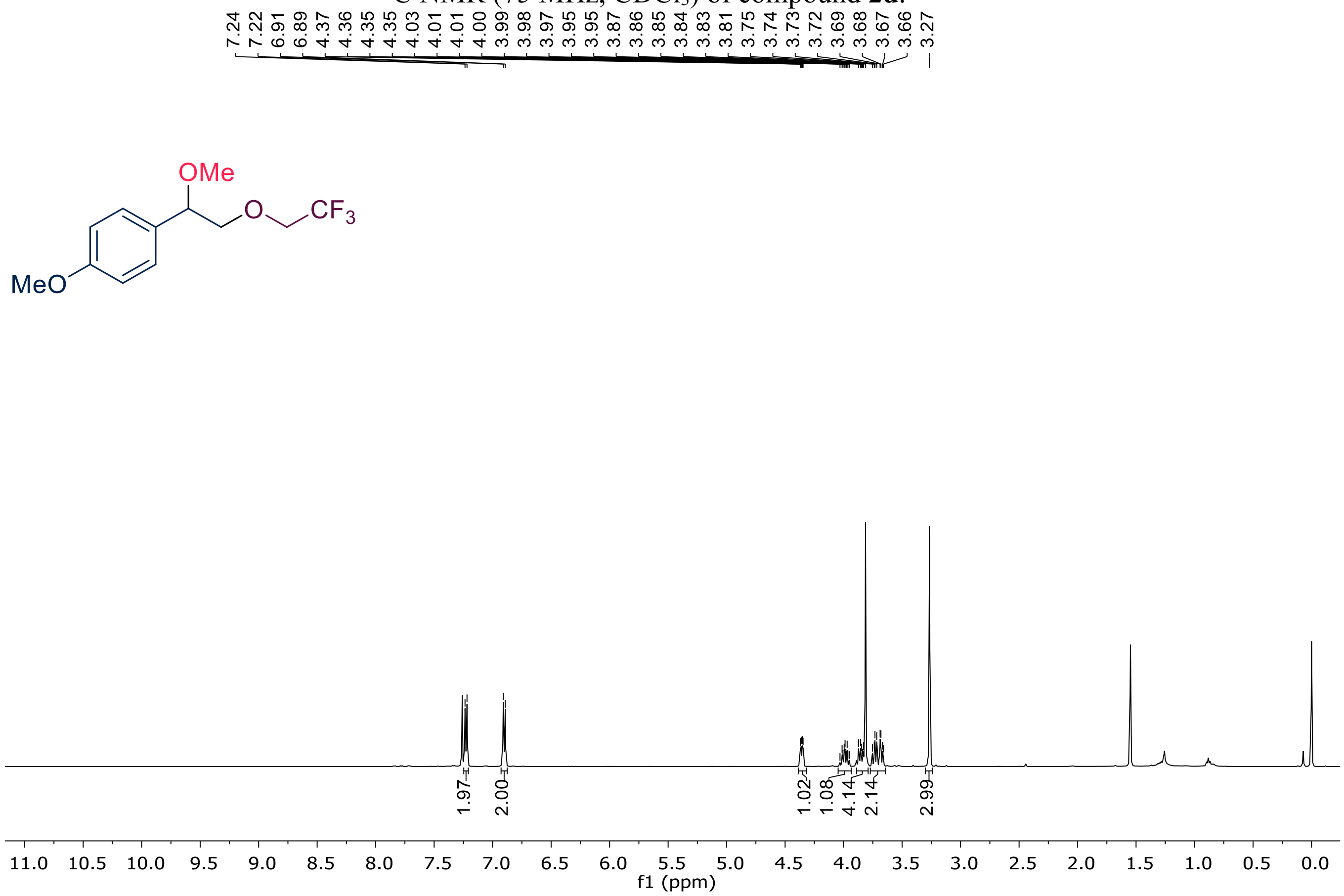

^{1}H NMR (500 MHz, $CDCl_3$) of compound **3**.

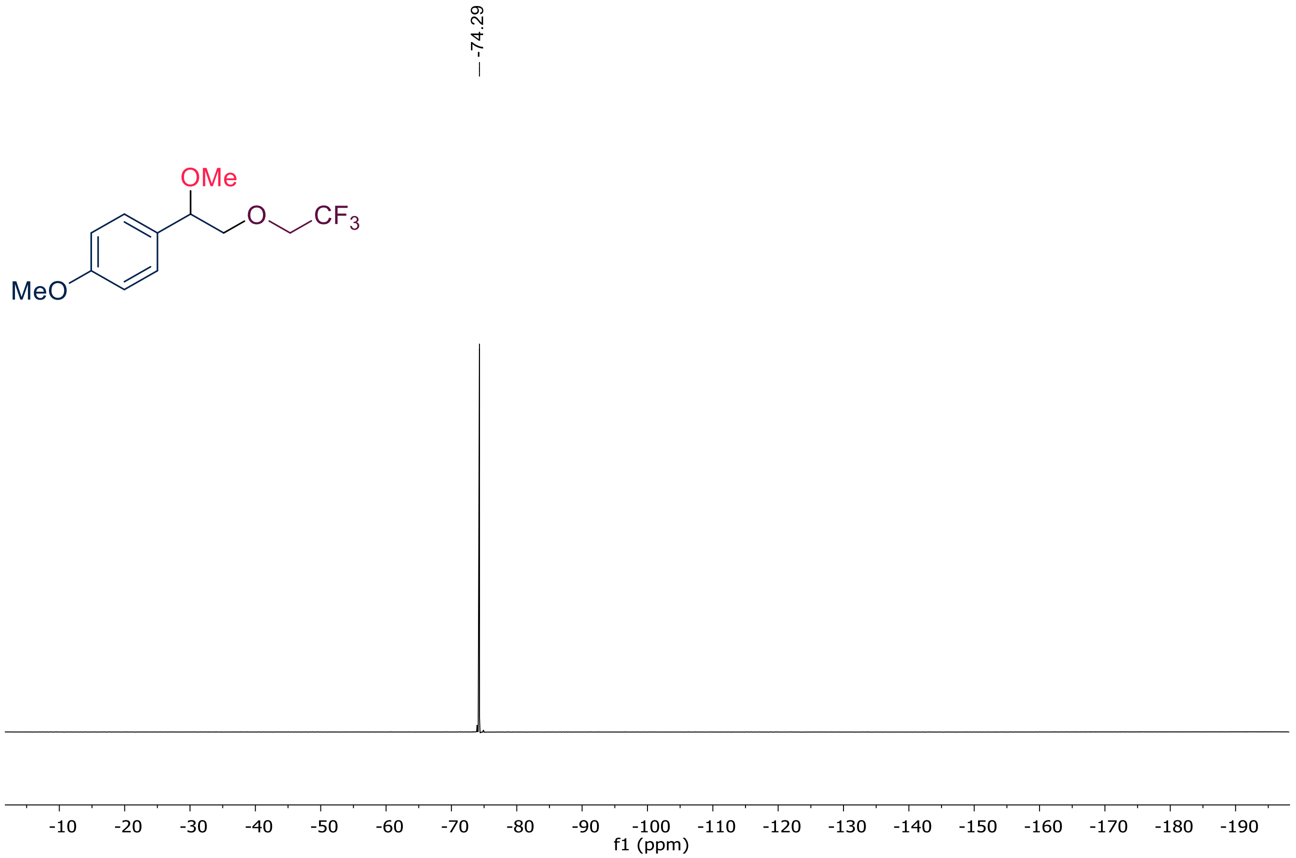

^{19}F NMR (282 MHz, $CDCl_3$) of compound **3**.

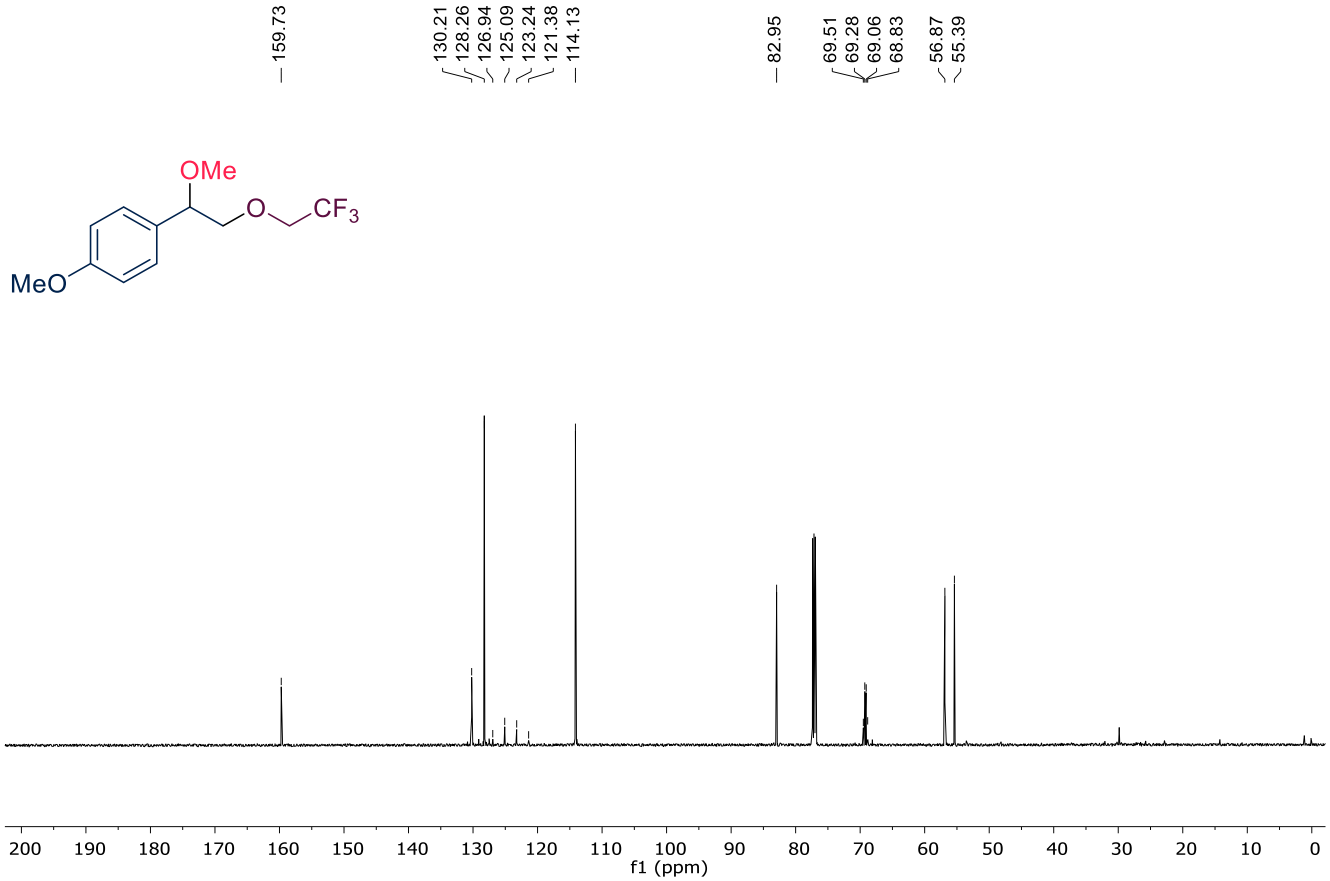

^{13}C NMR (151 MHz, $CDCl_3$) of compound **3**.

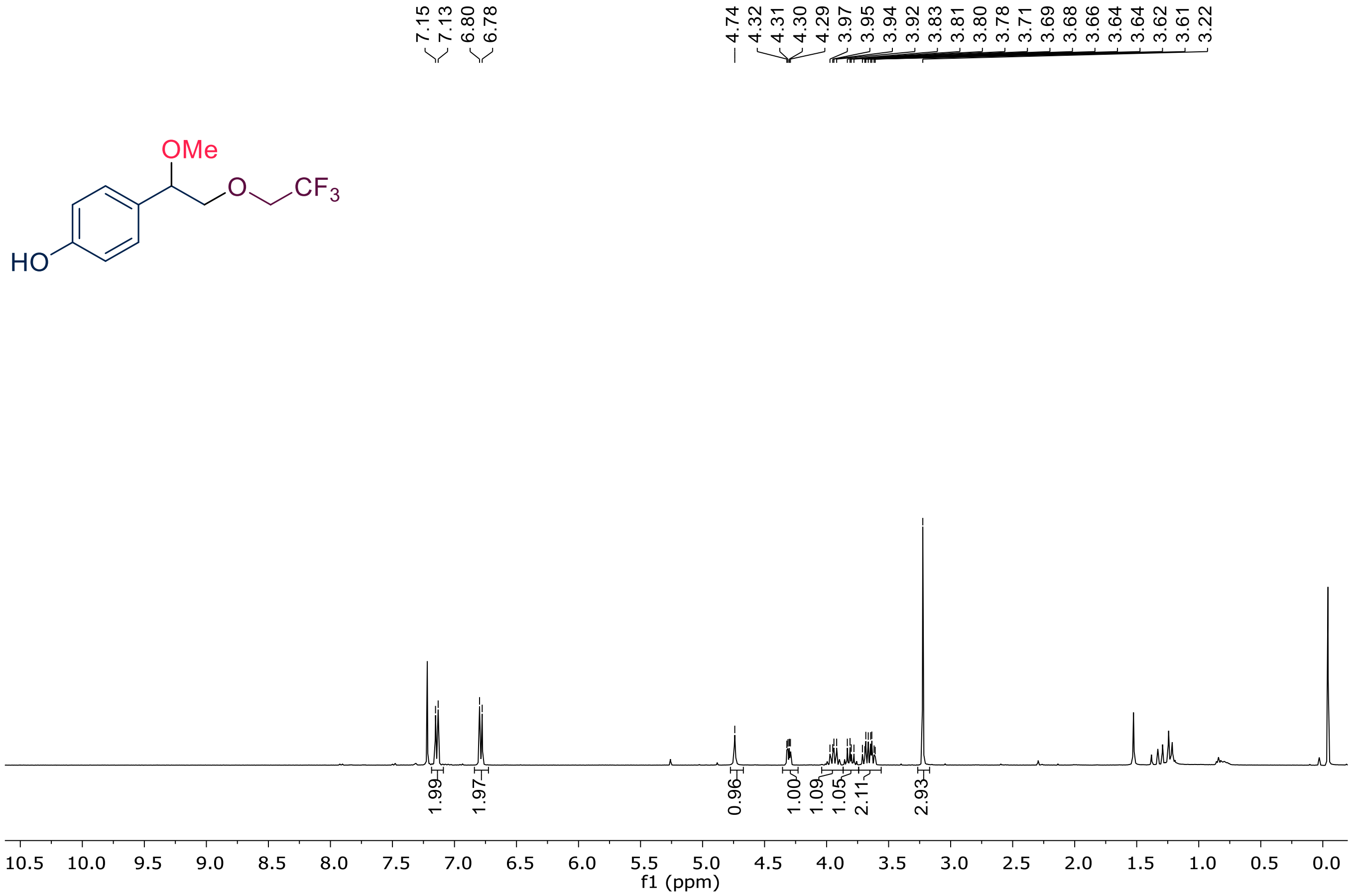

^{1}H NMR (500 MHz, $CDCl_3$) of compound **4**.

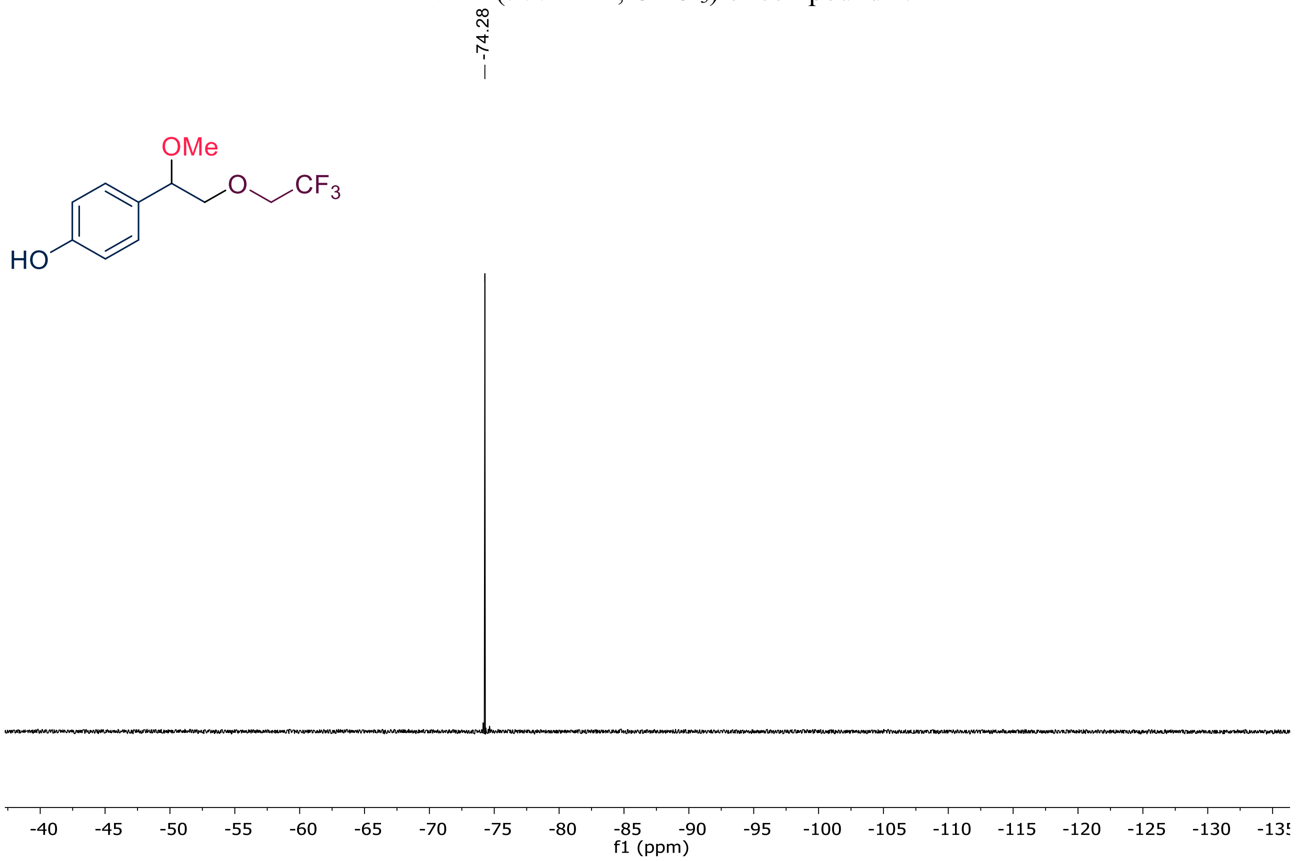

^{19}F NMR (282 MHz, $CDCl_3$) of compound **4**.

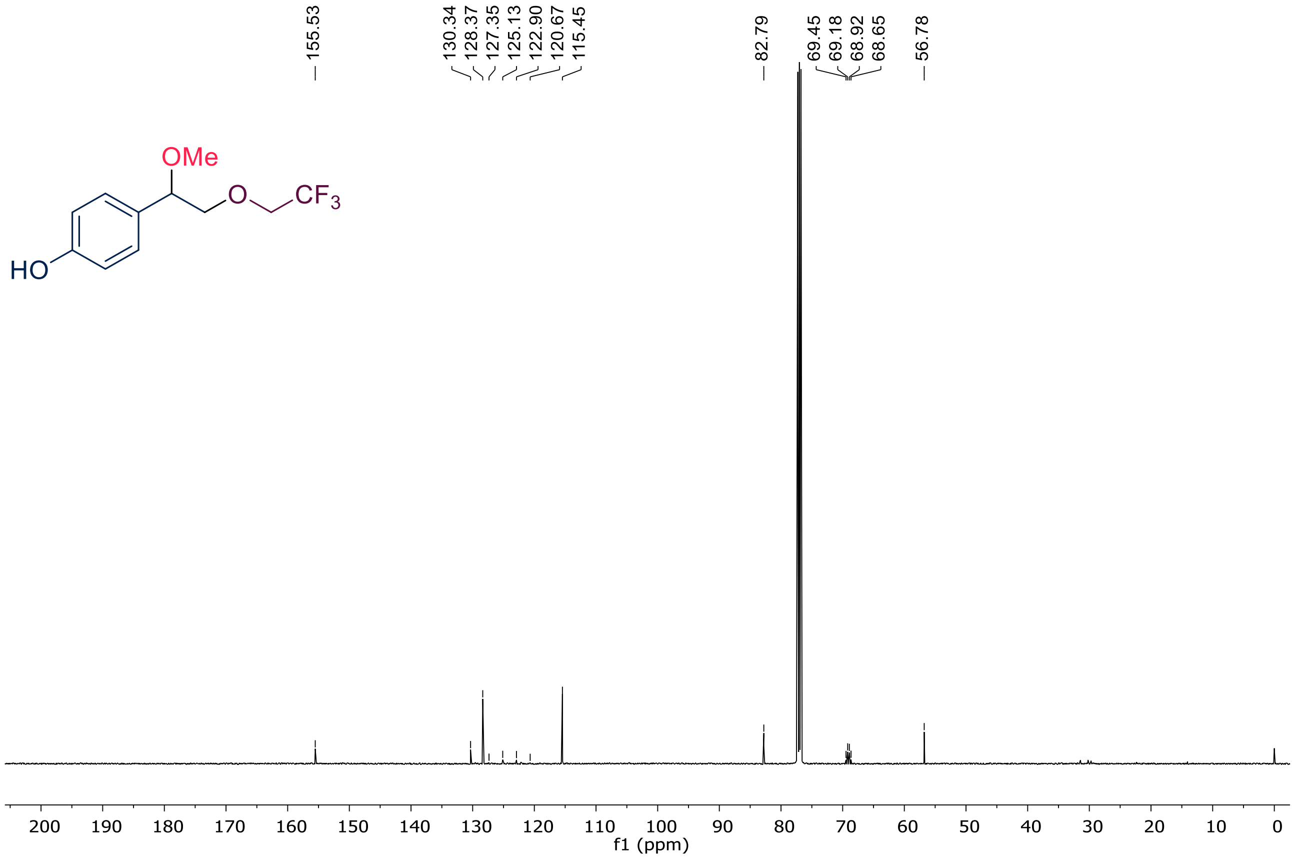

^{13}C NMR (101 MHz, $CDCl_3$) of compound **4**.

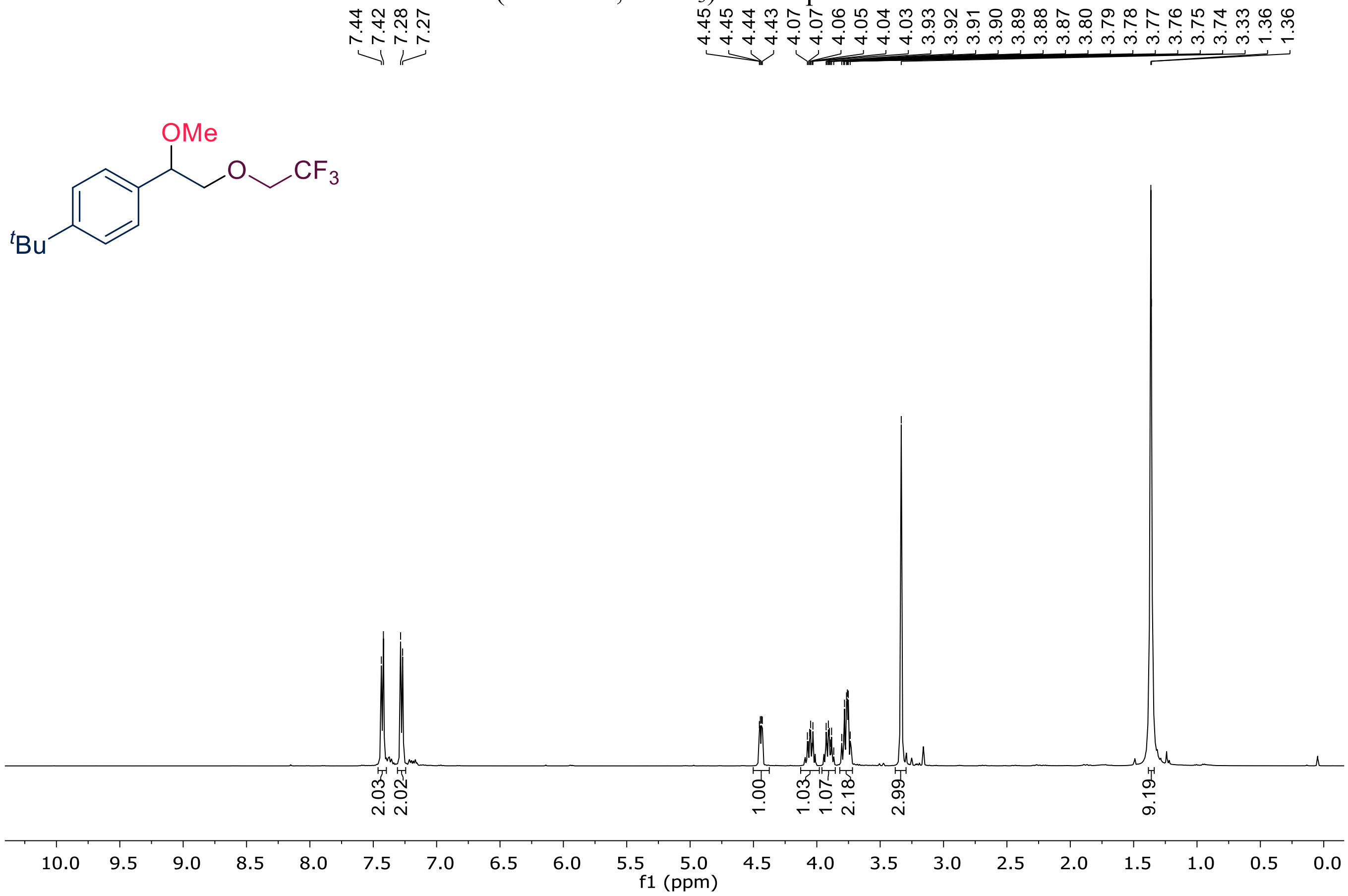

^{1}H NMR (300 MHz, $CDCl_3$) of compound **5**.

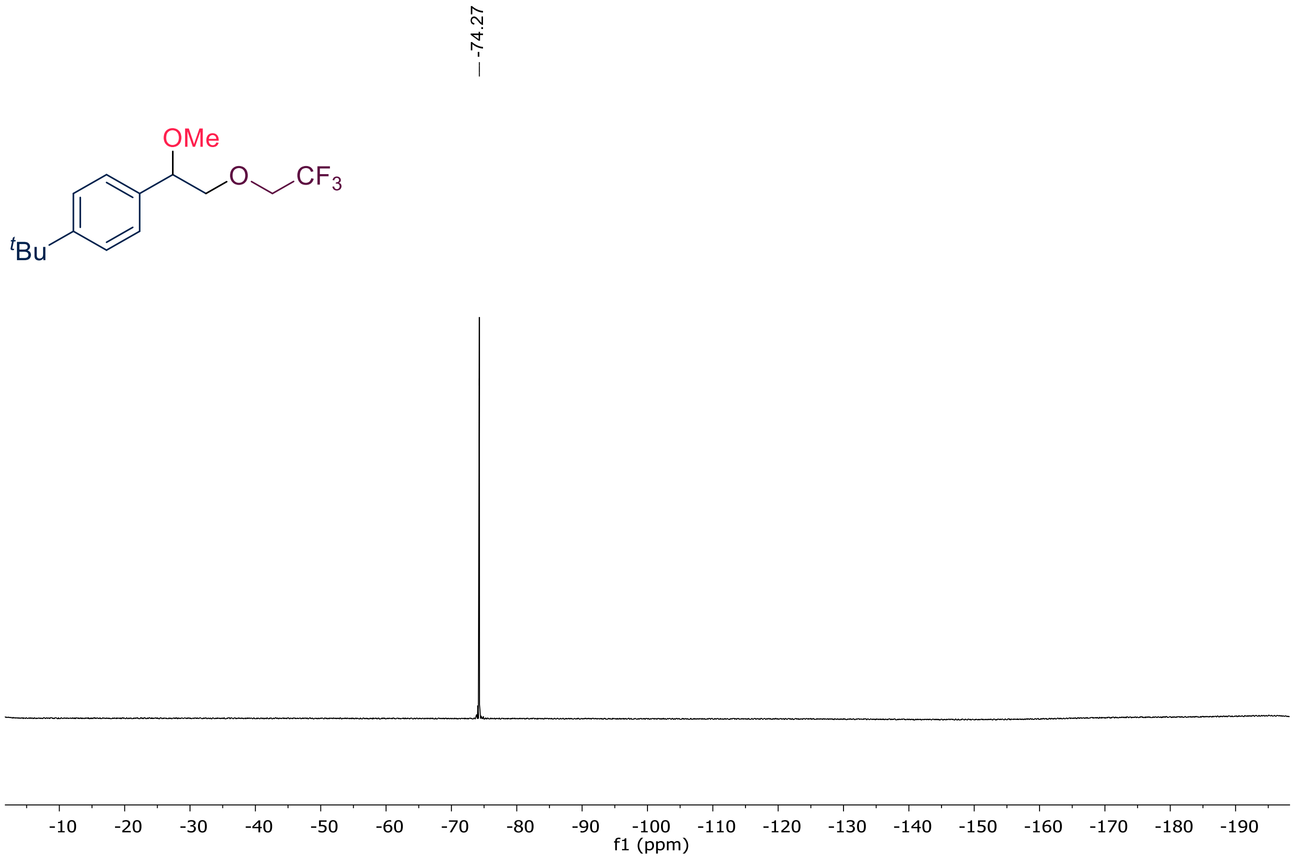

^{19}F NMR (282 MHz, $CDCl_3$) of compound **5**.

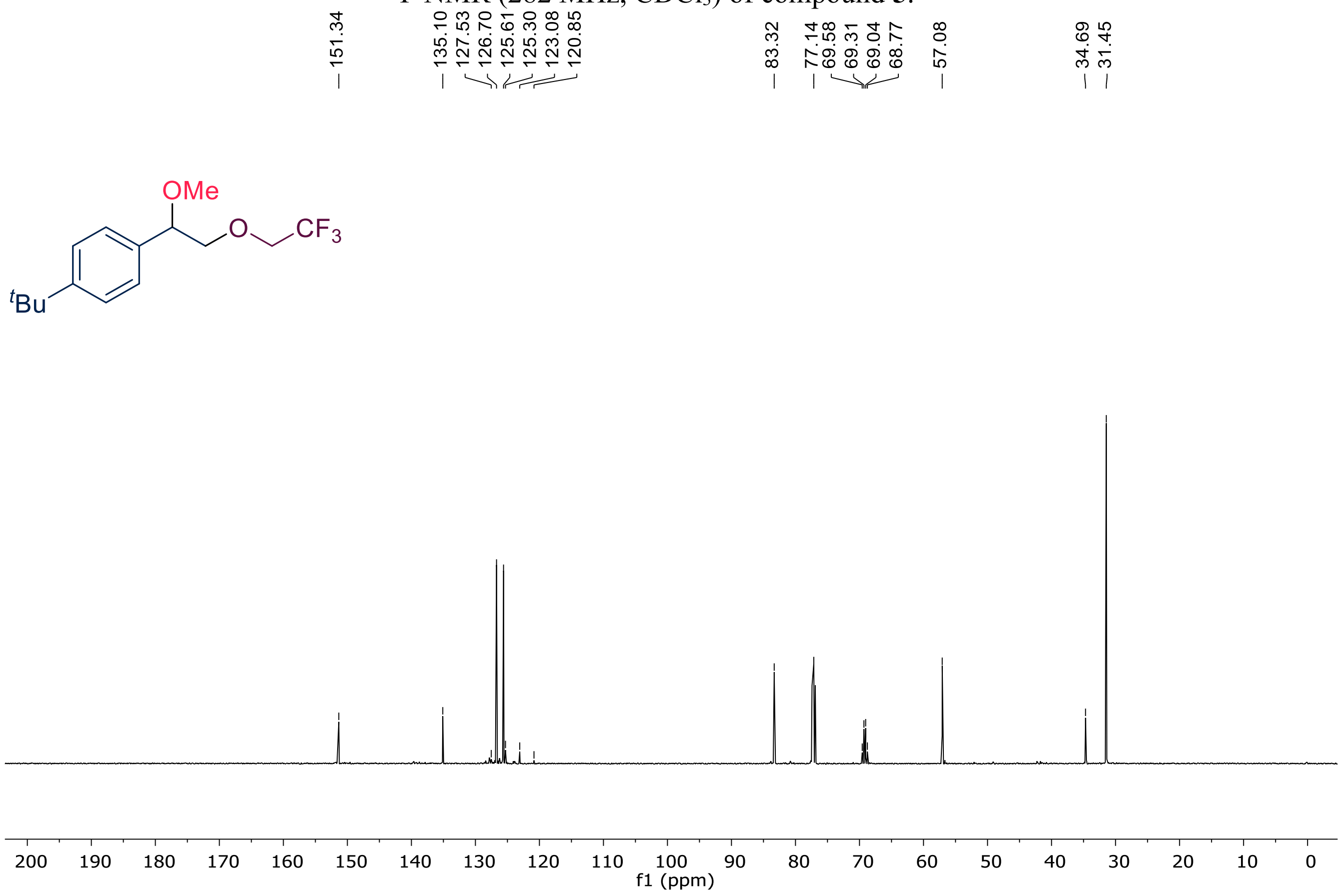

^{13}C NMR (101 MHz, $CDCl_3$) of compound **5**.

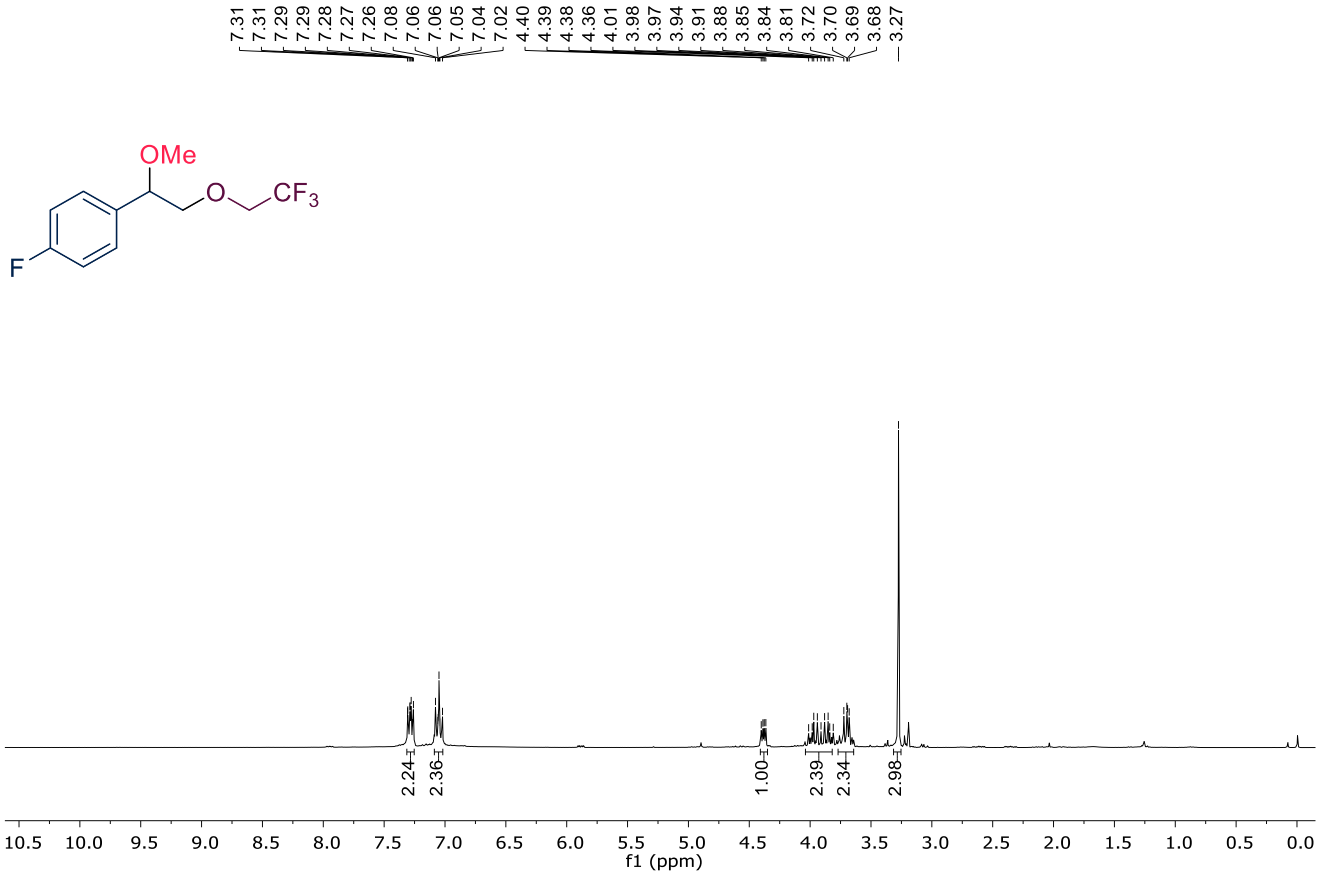

^{1}H NMR (300 MHz, $CDCl_3$) of compound **6**.

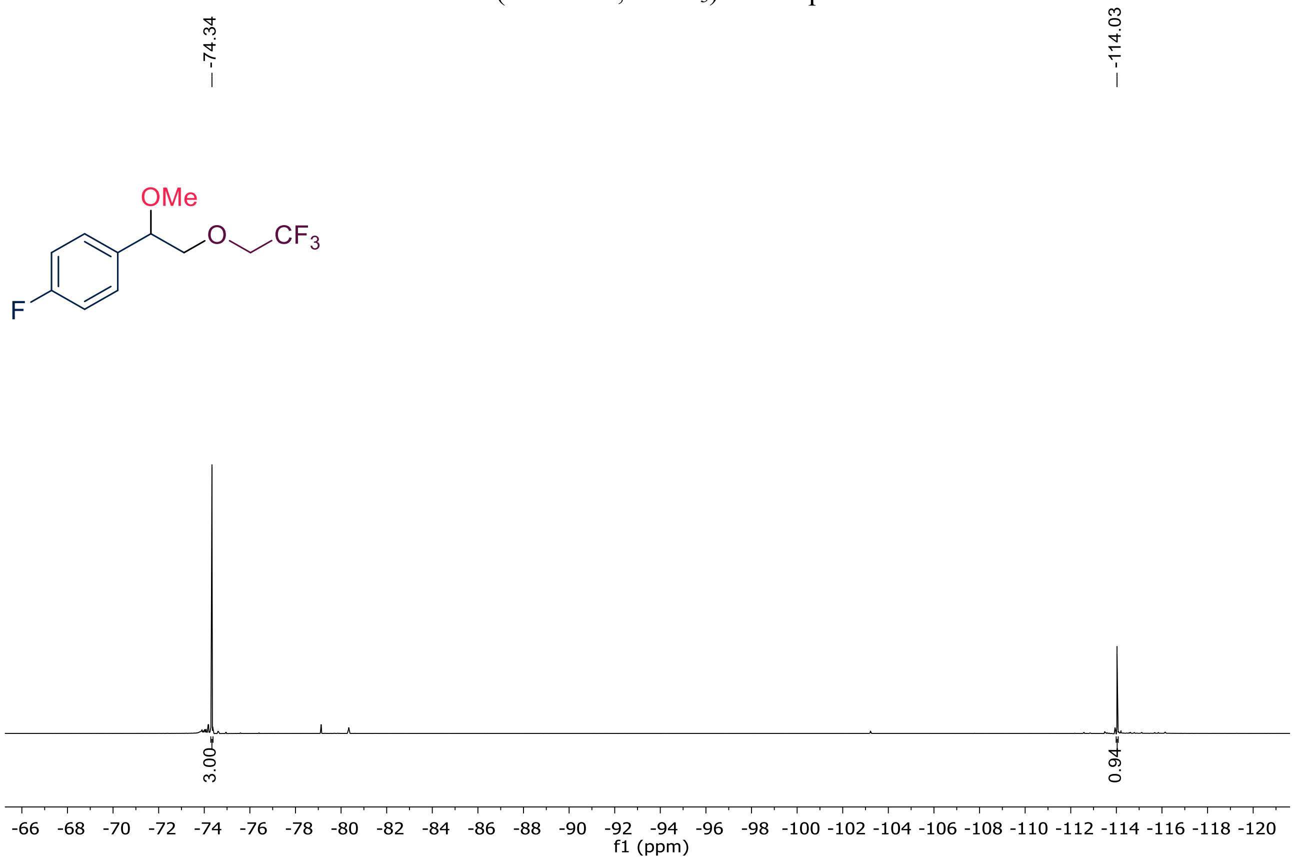

^{19}F NMR (282 MHz, $CDCl_3$) of compound **6**.

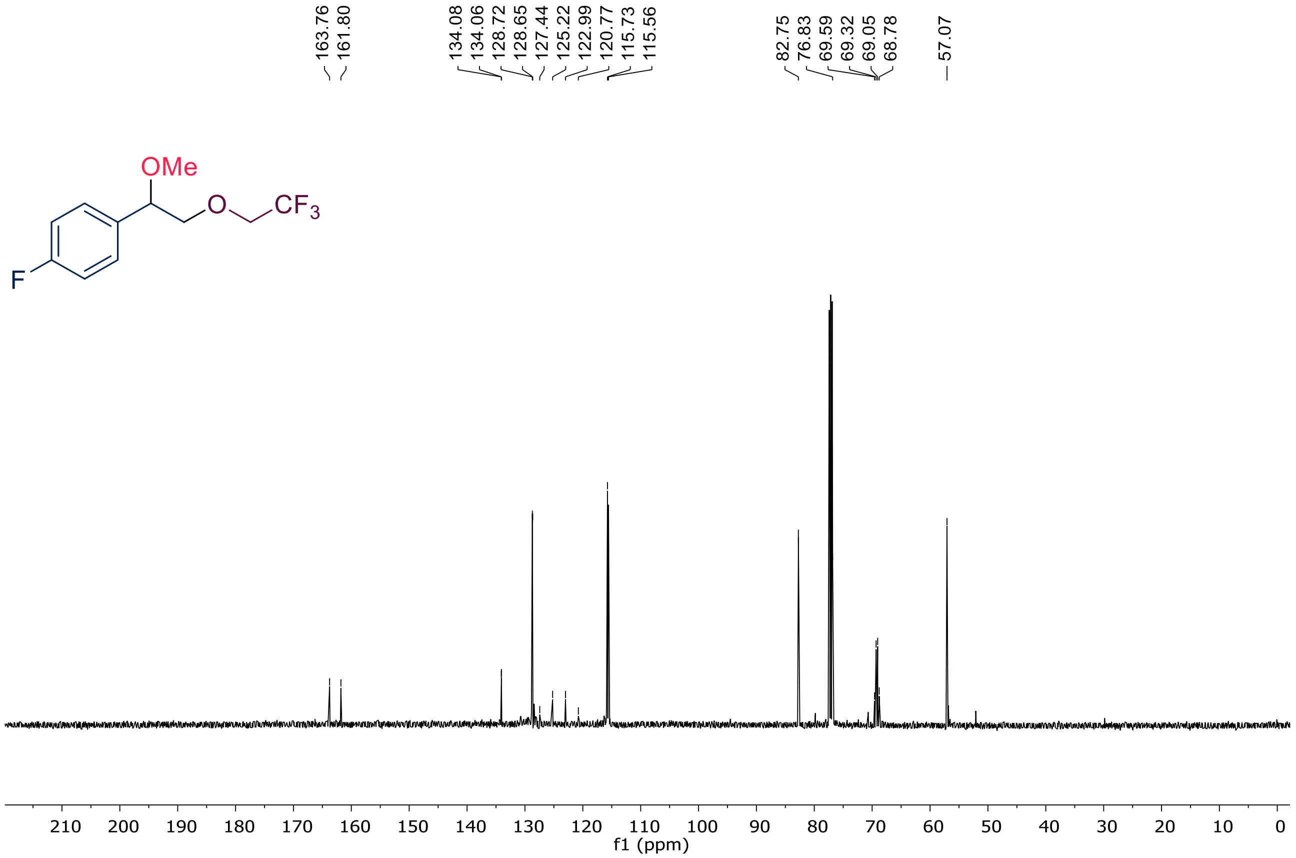

^{13}C NMR (126 MHz, $CDCl_3$) of compound **6**.

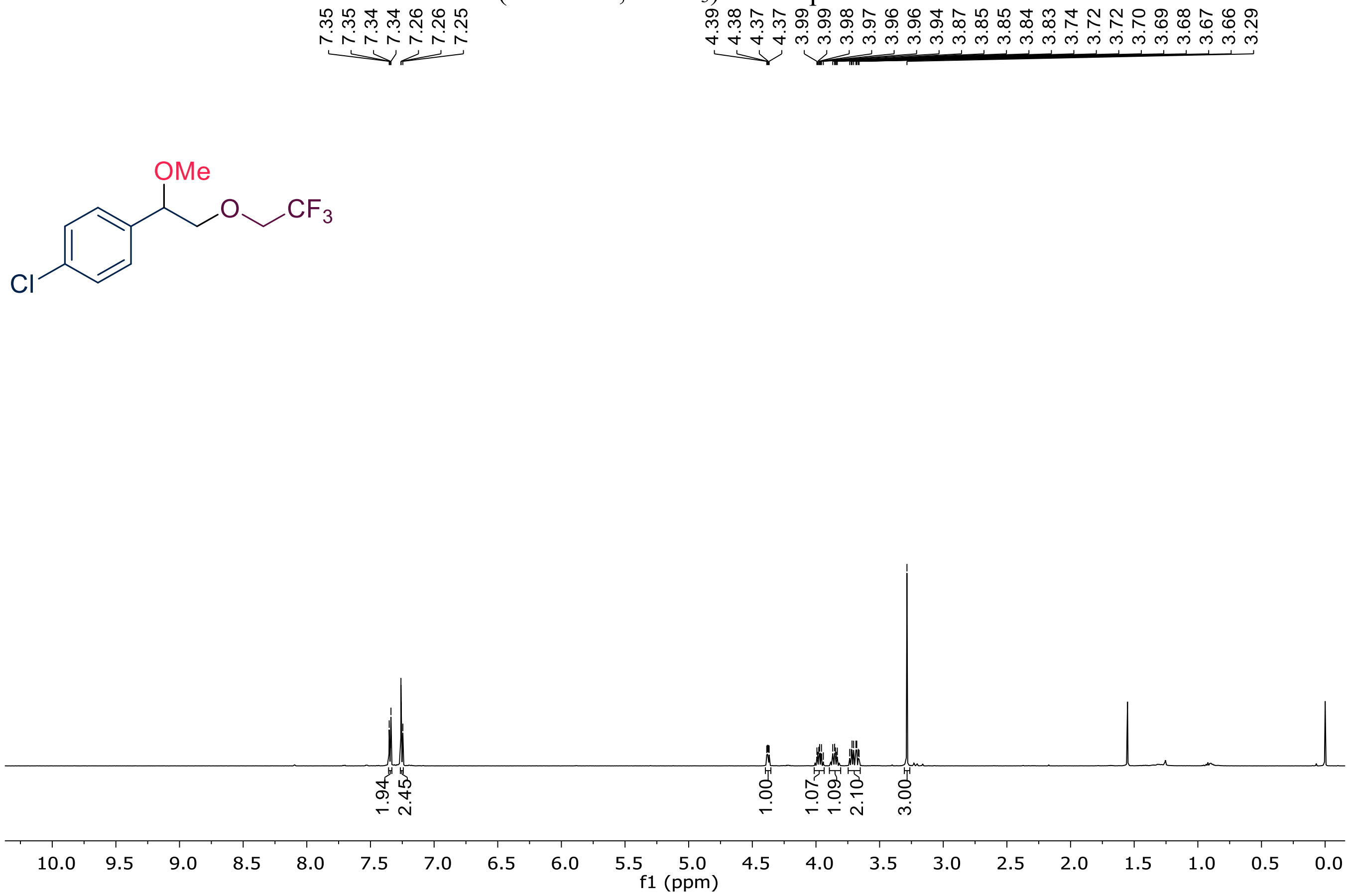

^{1}H NMR (600 MHz, $CDCl_3$) of compound **7**.

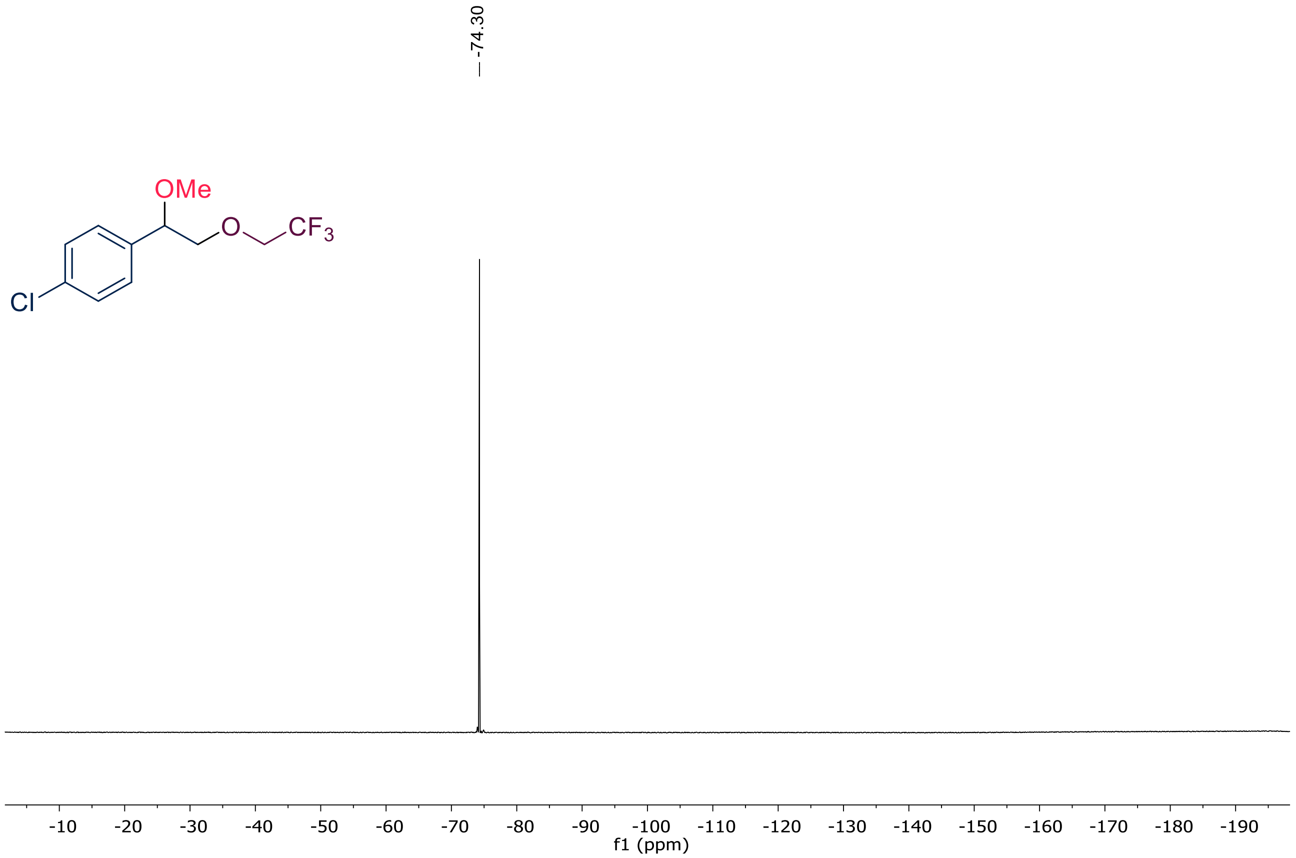

^{19}F NMR (282 MHz, $CDCl_3$) of compound **7**.

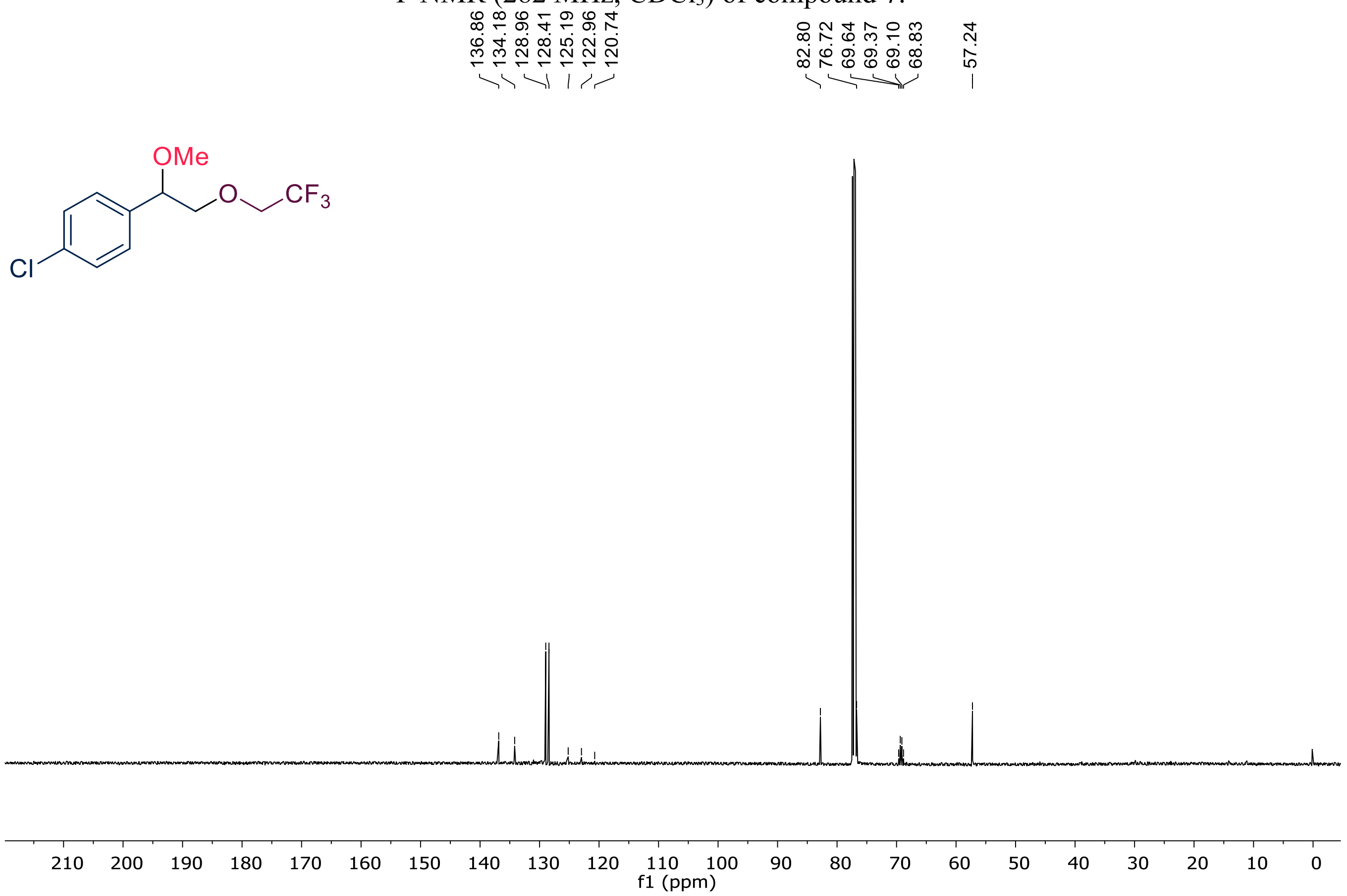

^{13}C NMR (126 MHz, $CDCl_3$) of compound **7**.

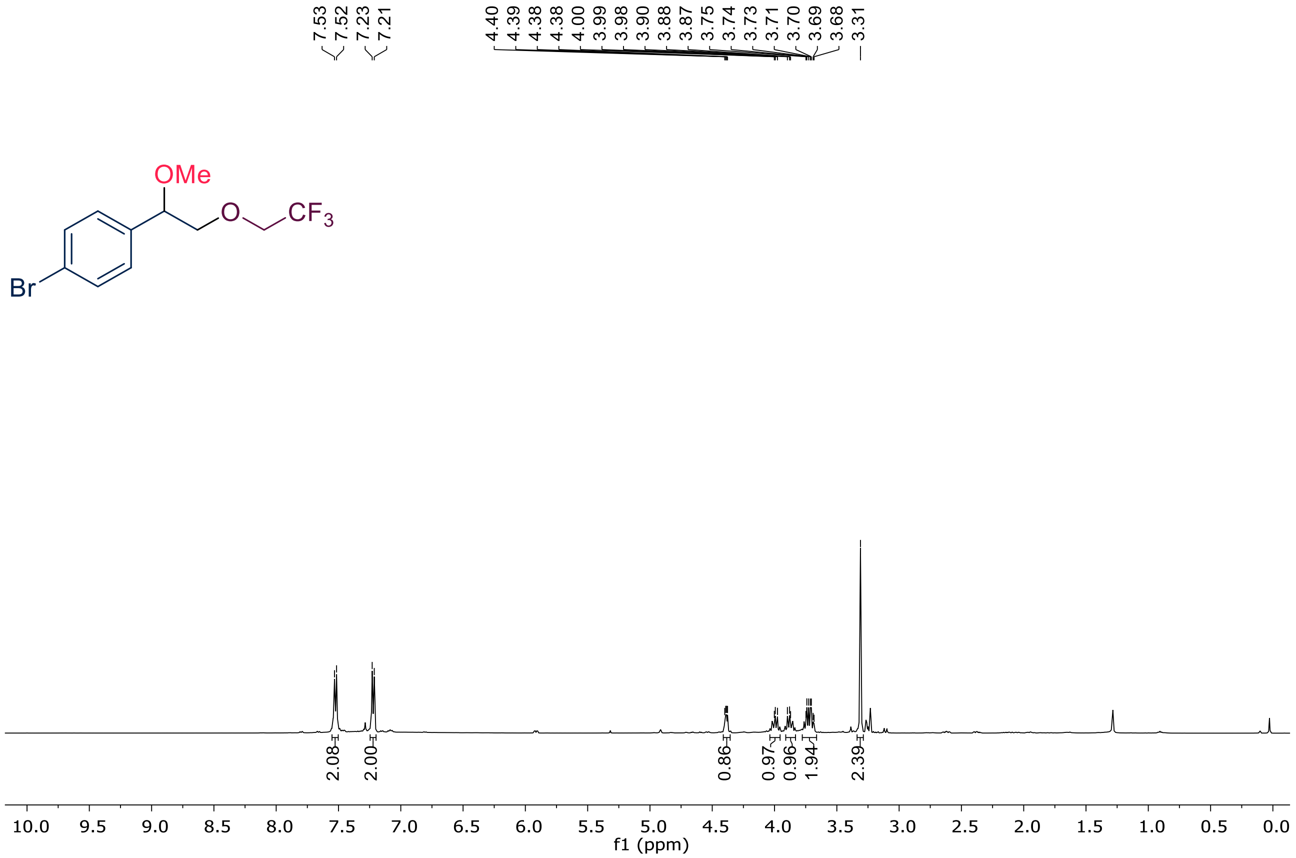

^{1}H NMR (500 MHz, $CDCl_3$) of compound **8**.

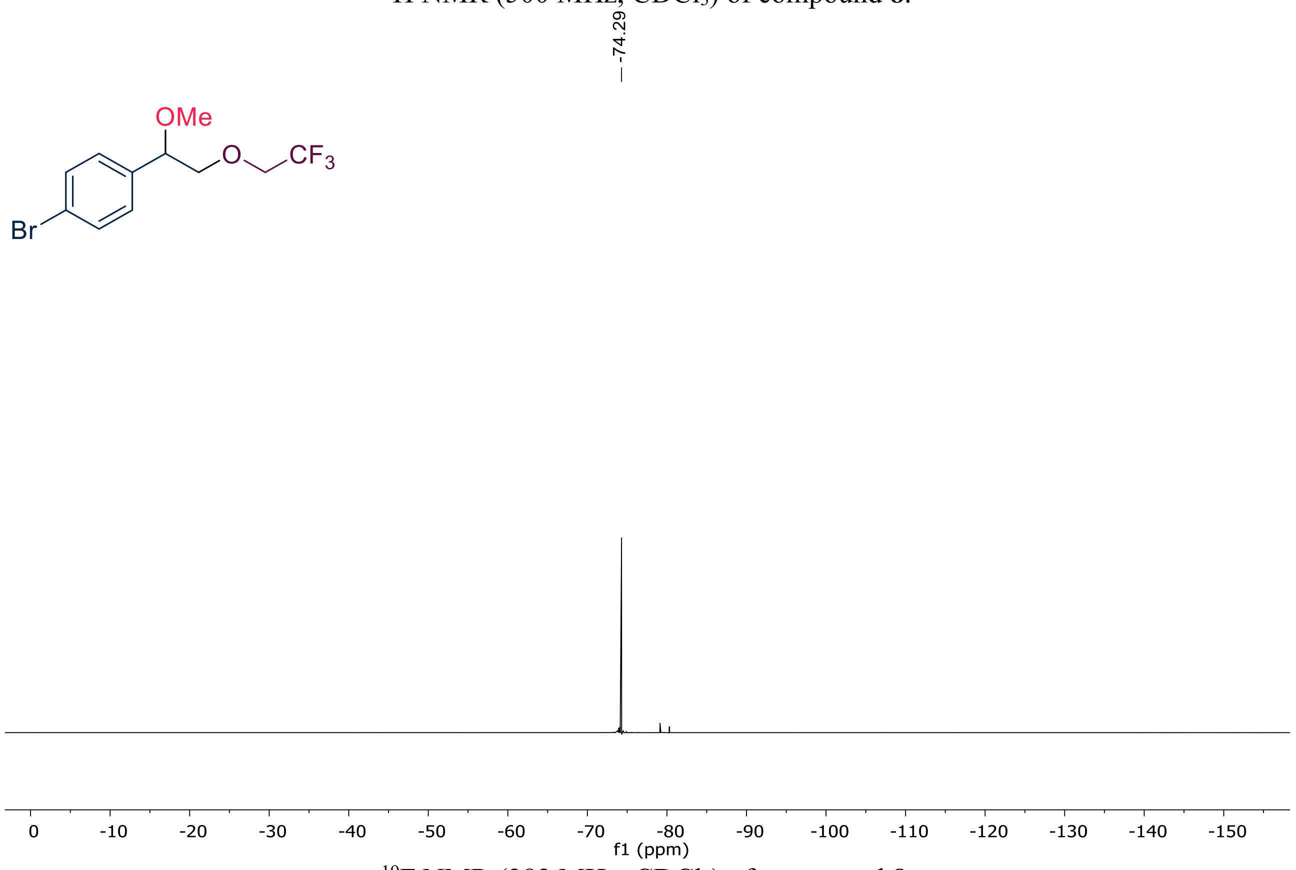

^{19}F NMR (282 MHz, $CDCl_3$) of compound **8**.

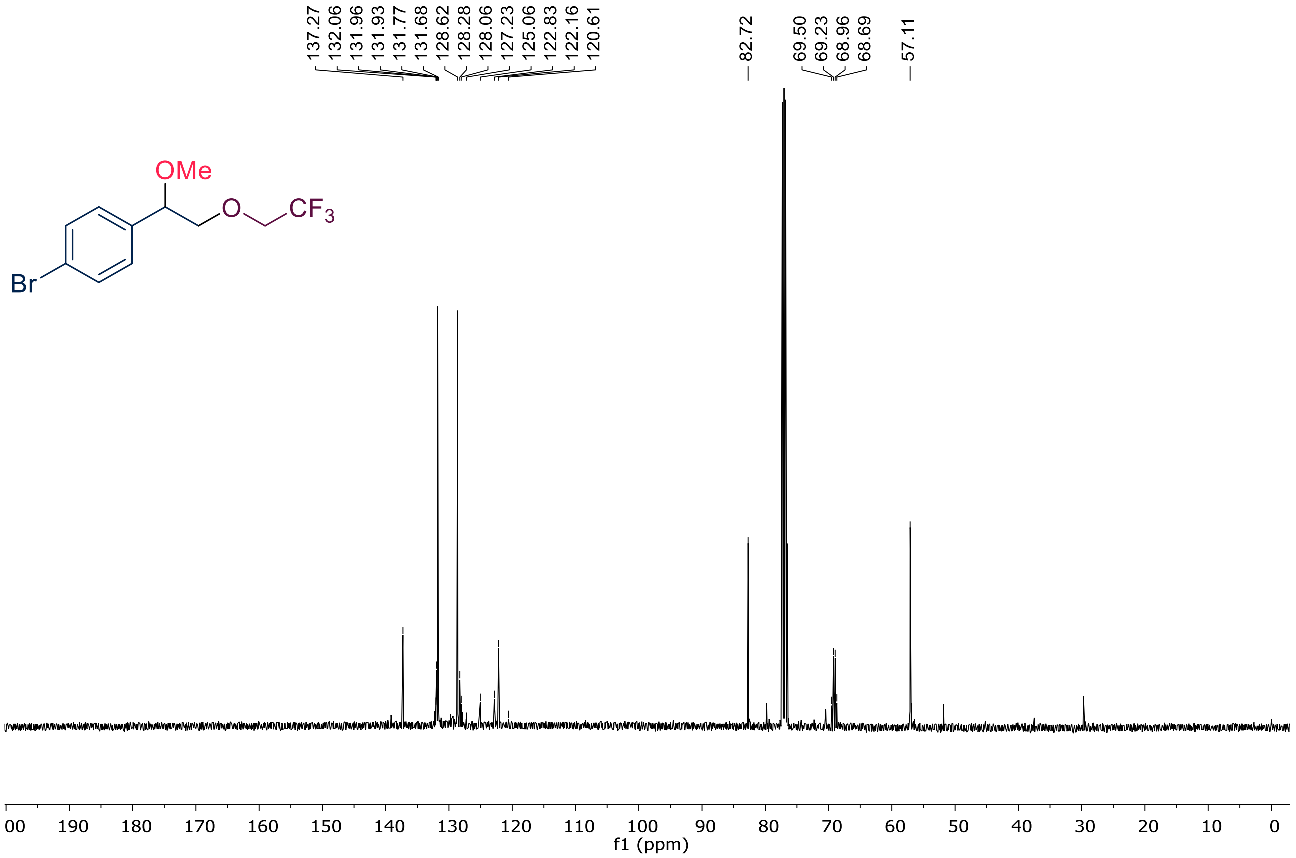

^{13}C NMR (126 MHz, $CDCl_3$) of compound **8**.

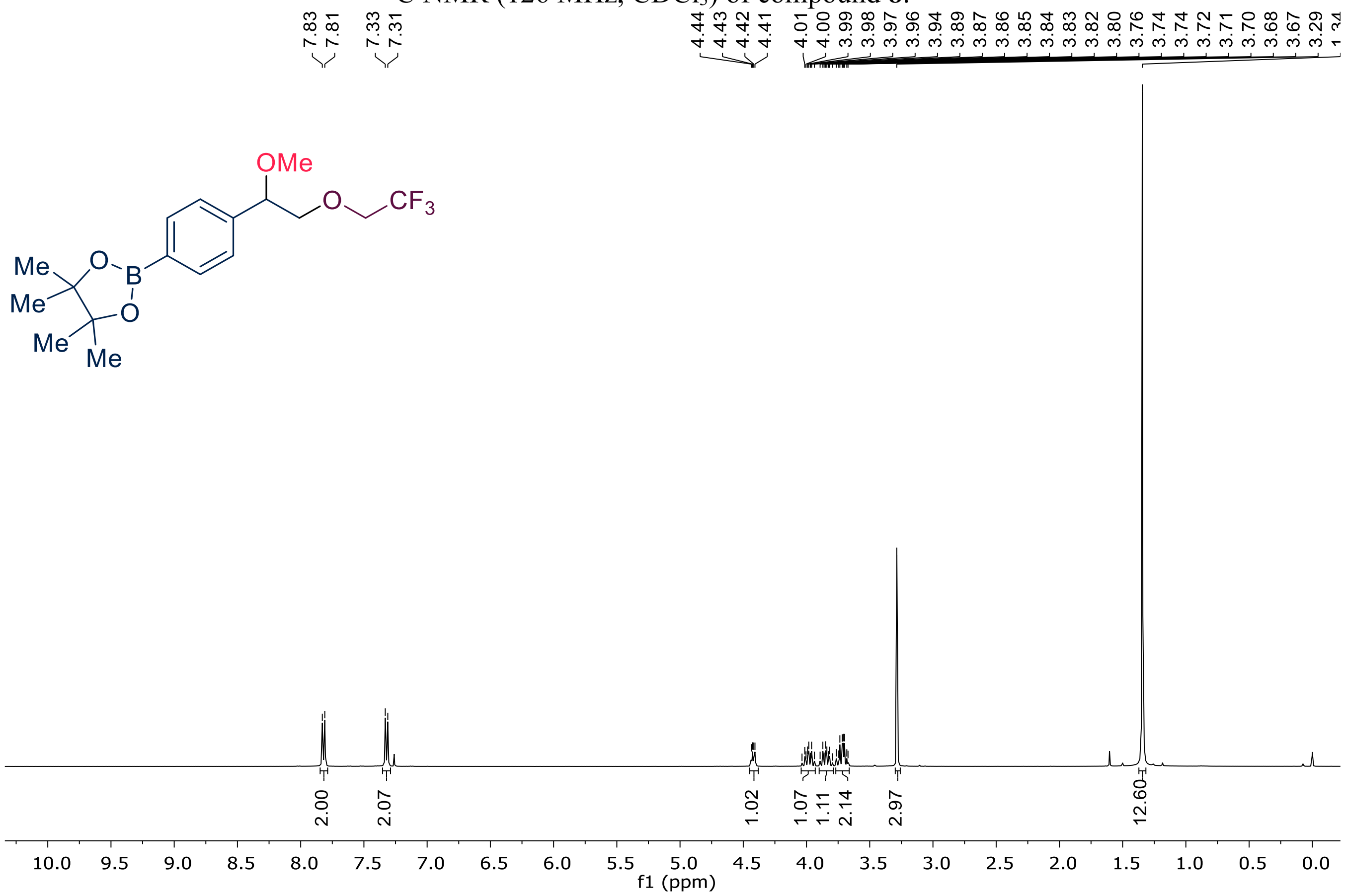

^{1}H NMR (400 MHz, $CDCl_3$) of compound **9**.

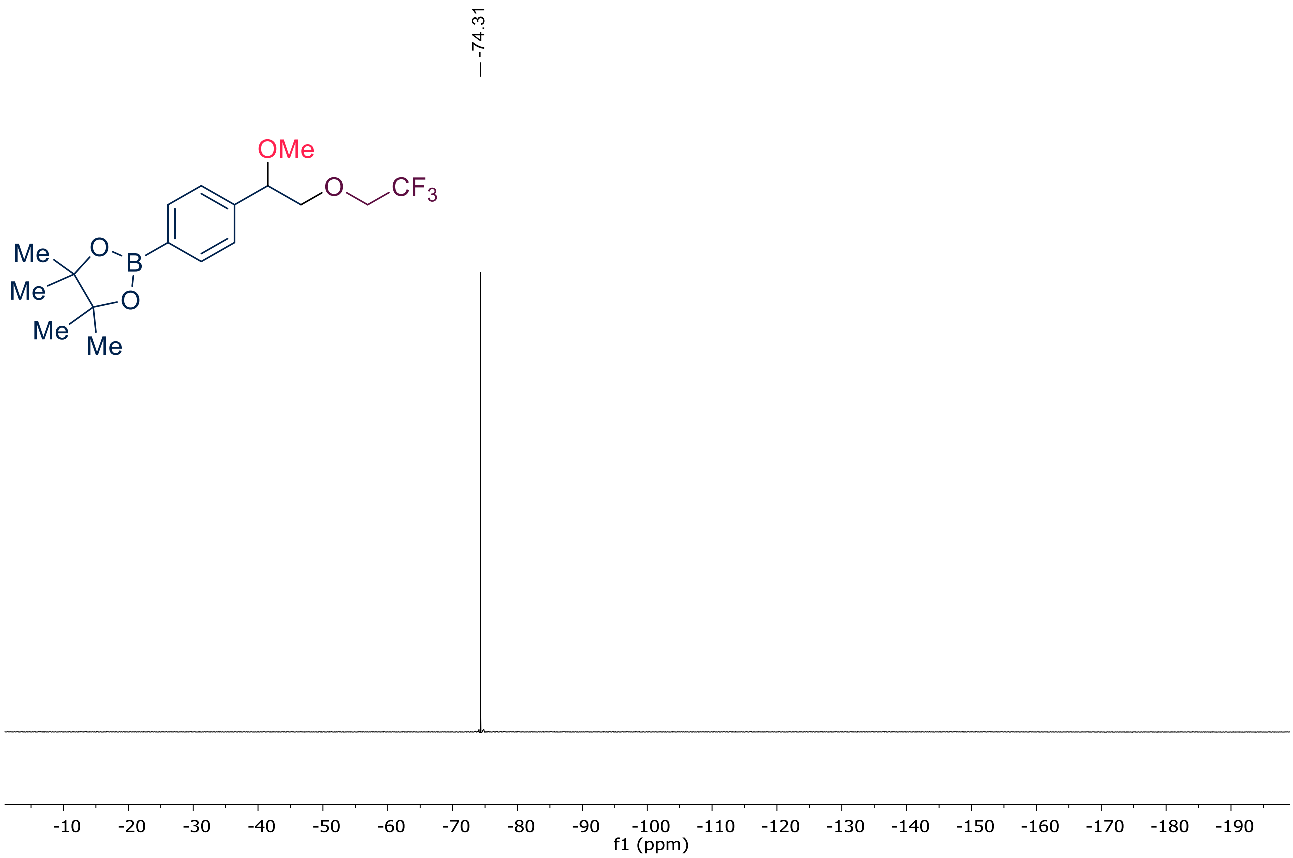

^{19}F NMR (377 MHz, $CDCl_3$) of compound **9**.

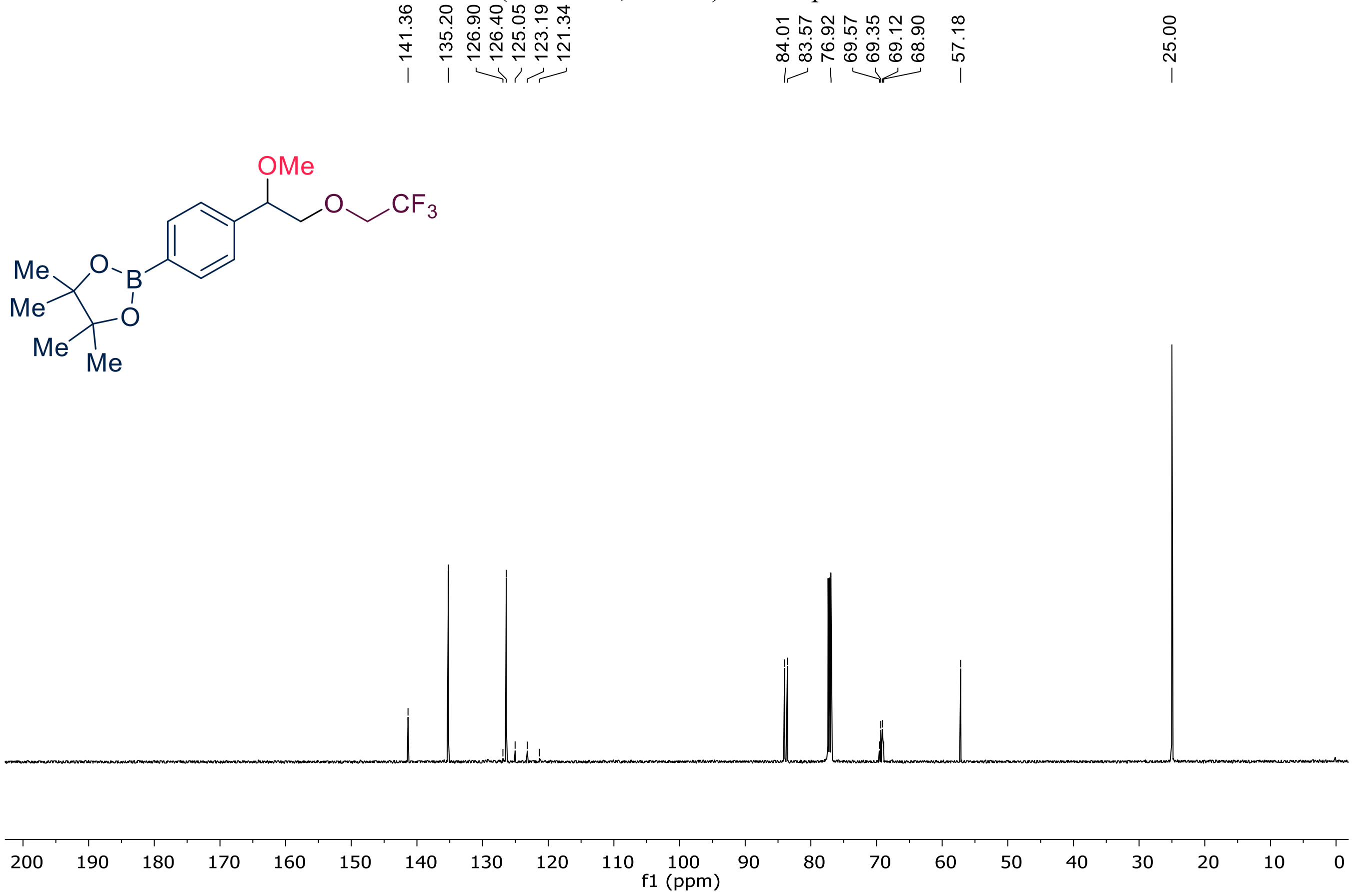

^{13}C NMR (151 MHz, $CDCl_3$) of compound **9**.

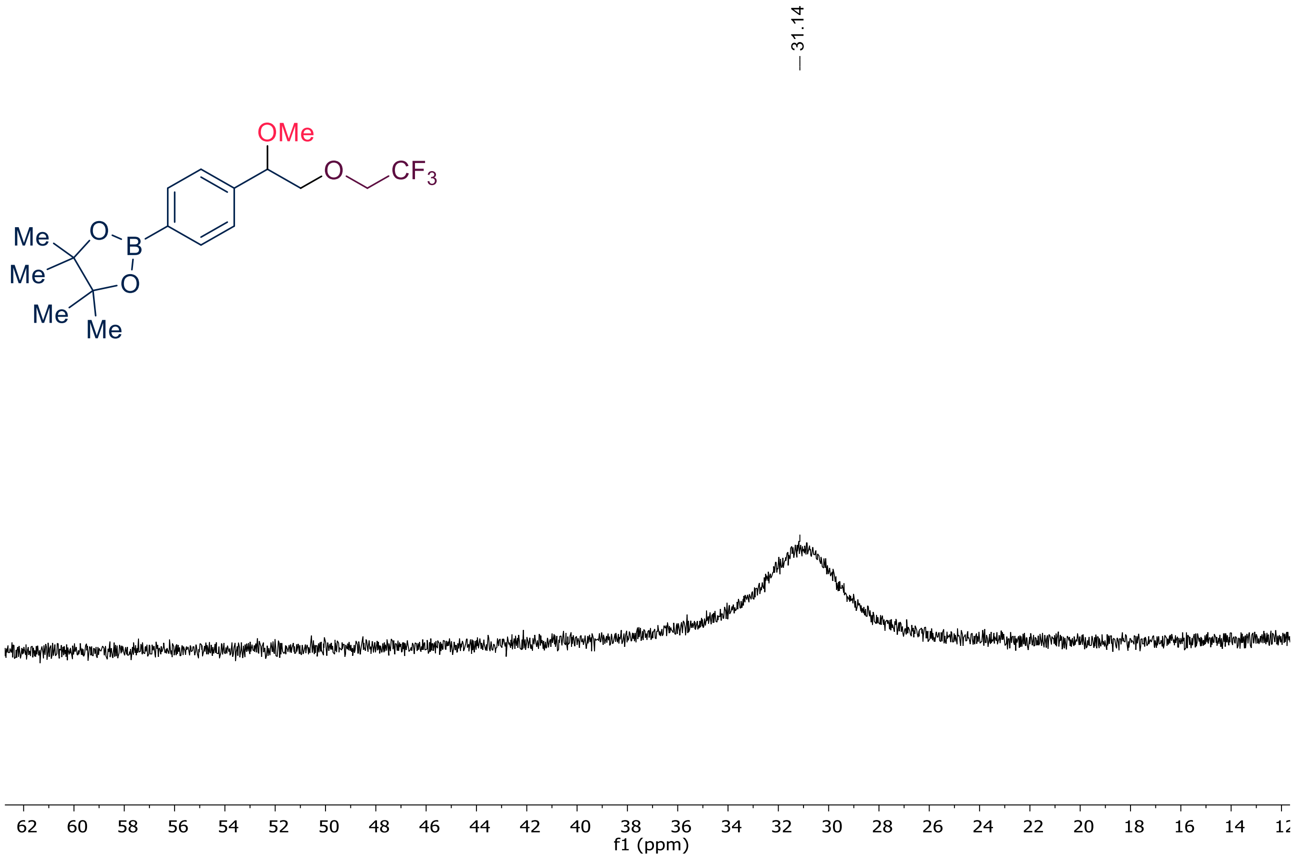

^{11}B NMR (128 MHz, $CDCl_3$) of compound **9**.

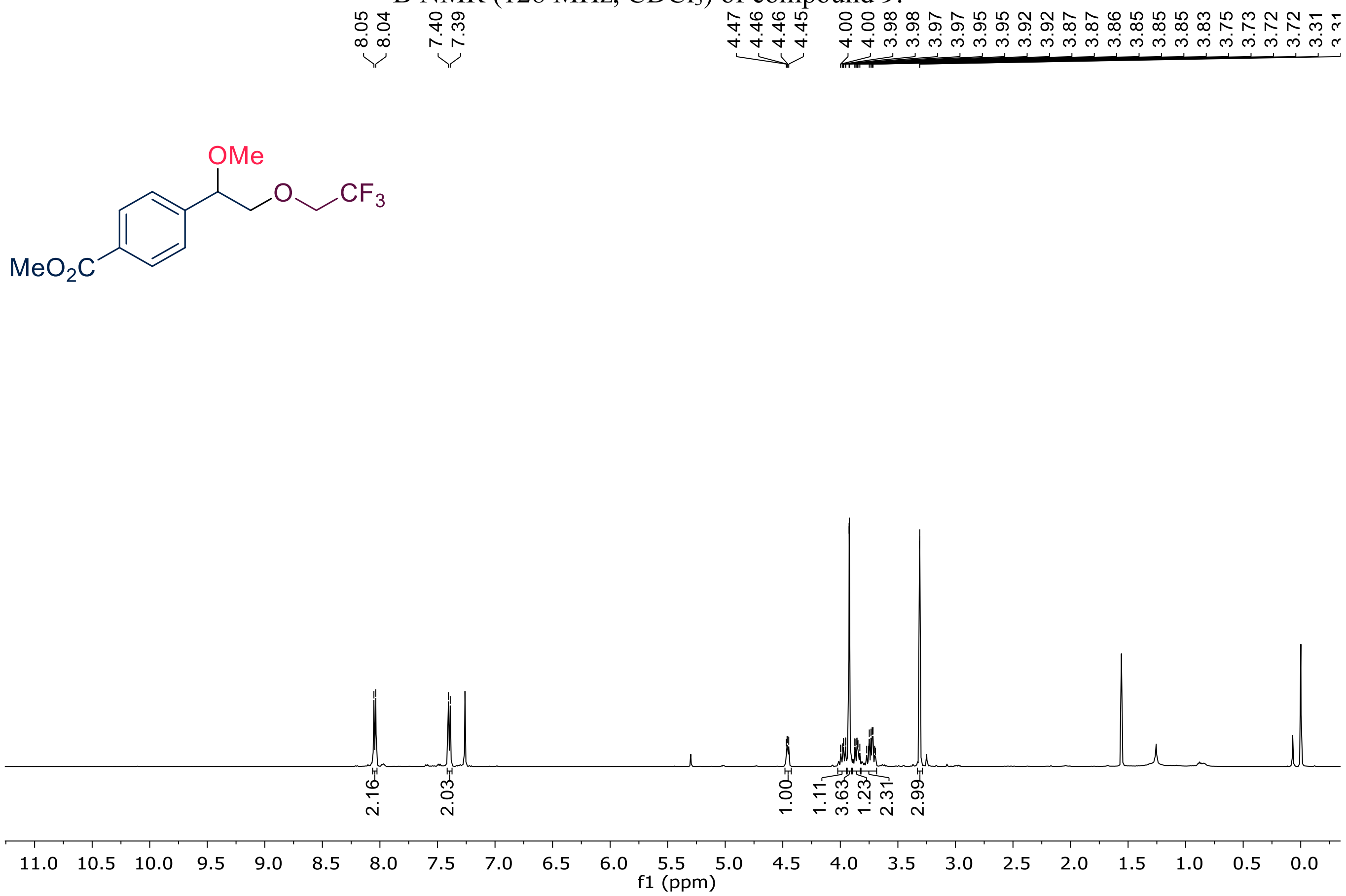

^{1}H NMR (500 MHz, $CDCl_3$) of compound **10**.

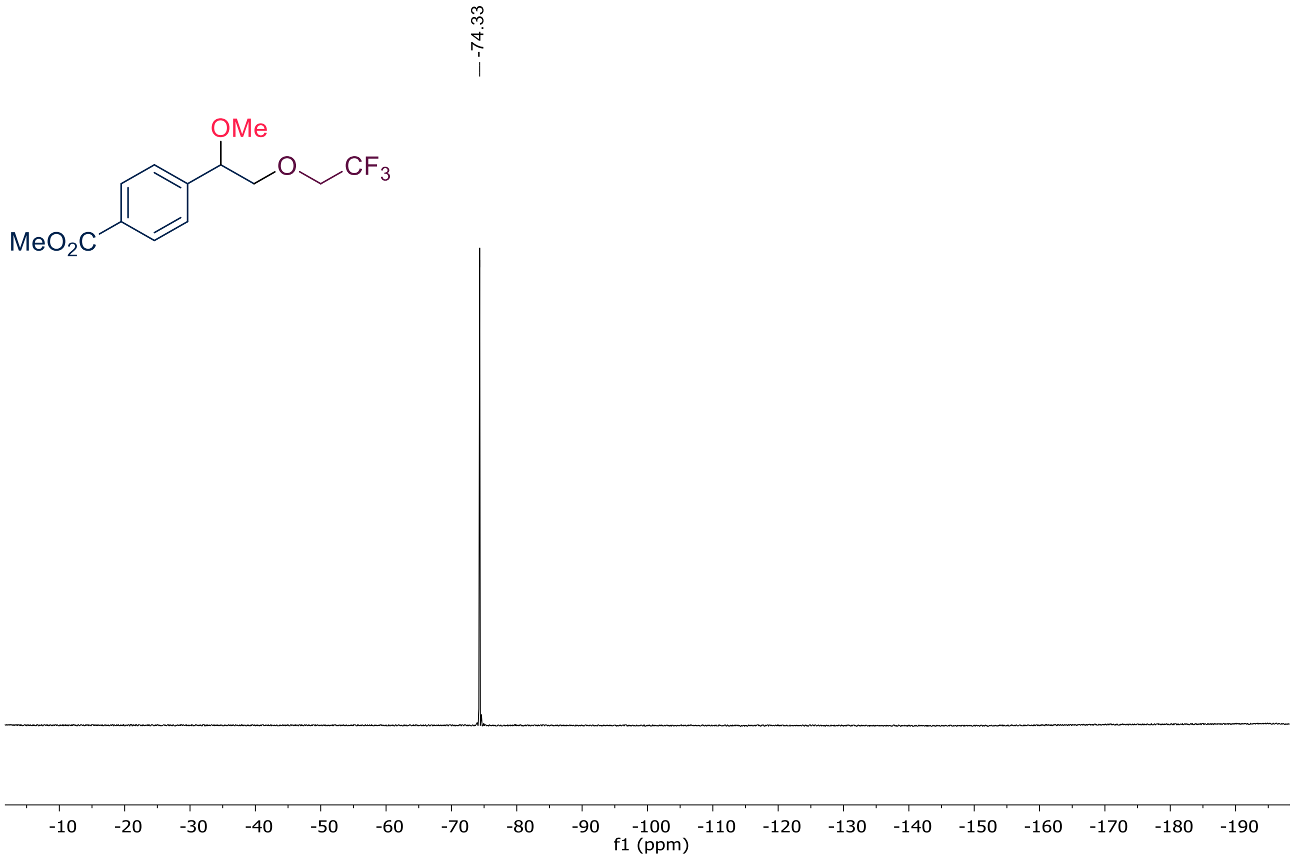

^{19}F NMR (282 MHz, $CDCl_3$) of compound **10**.

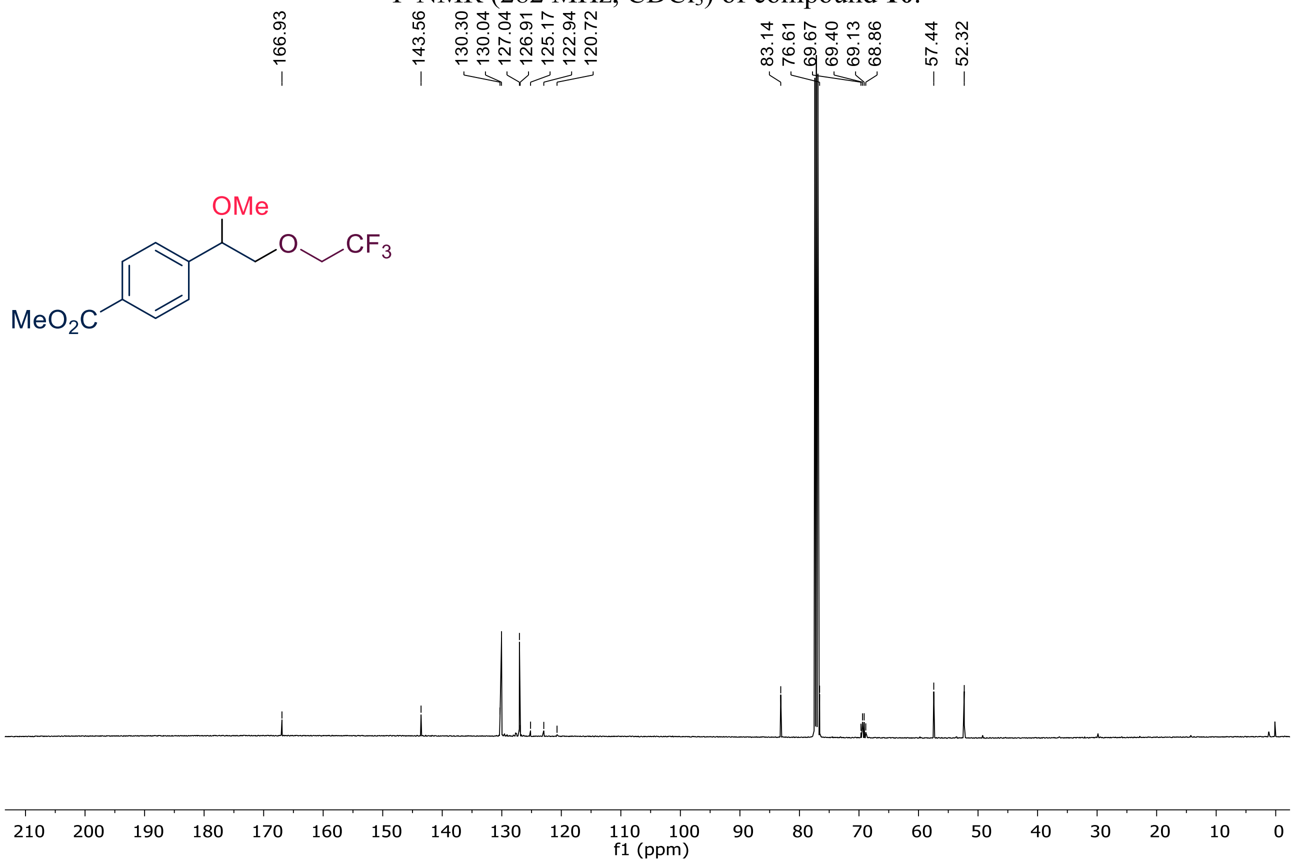

^{13}C NMR (126 MHz, $CDCl_3$) of compound **10**.

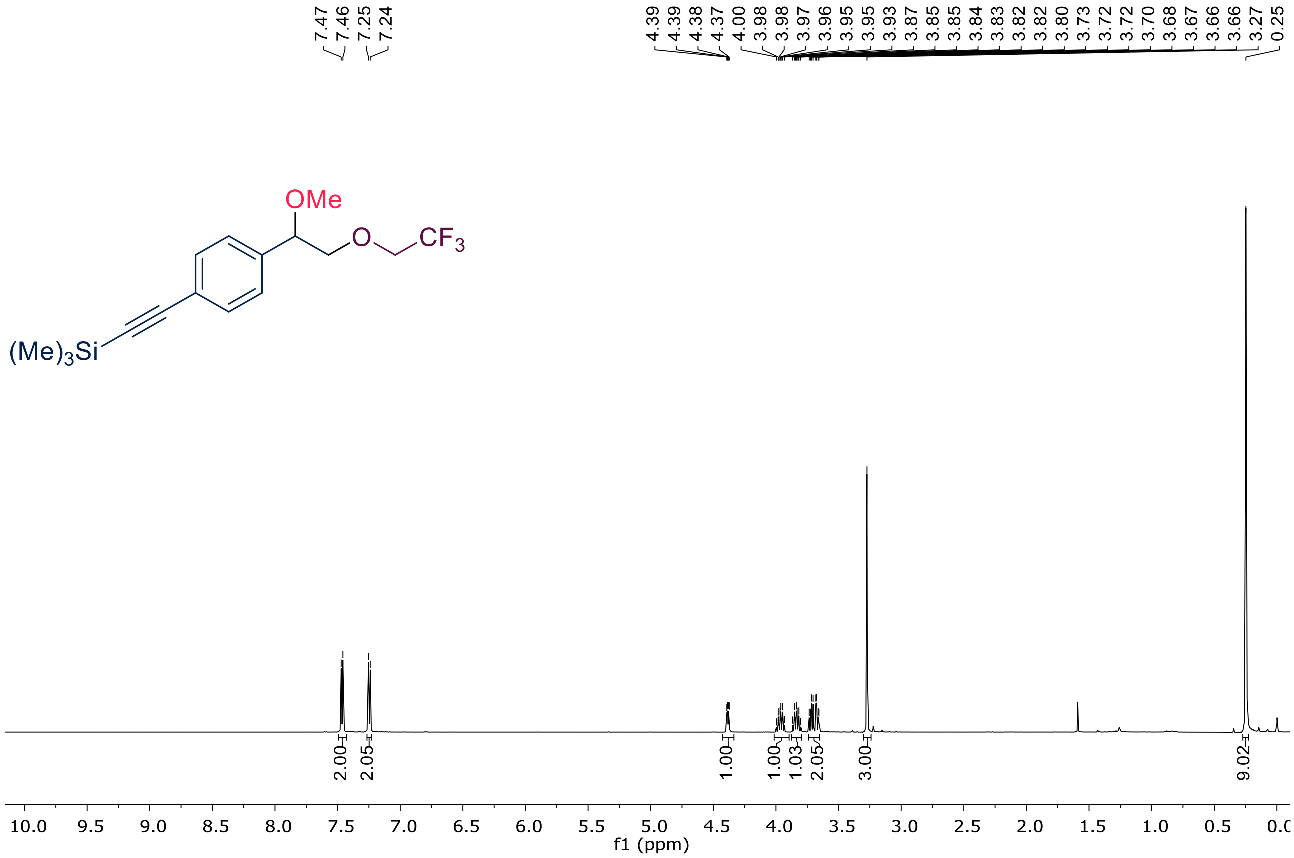

^{1}H NMR (600 MHz, $CDCl_3$) of compound **11**.

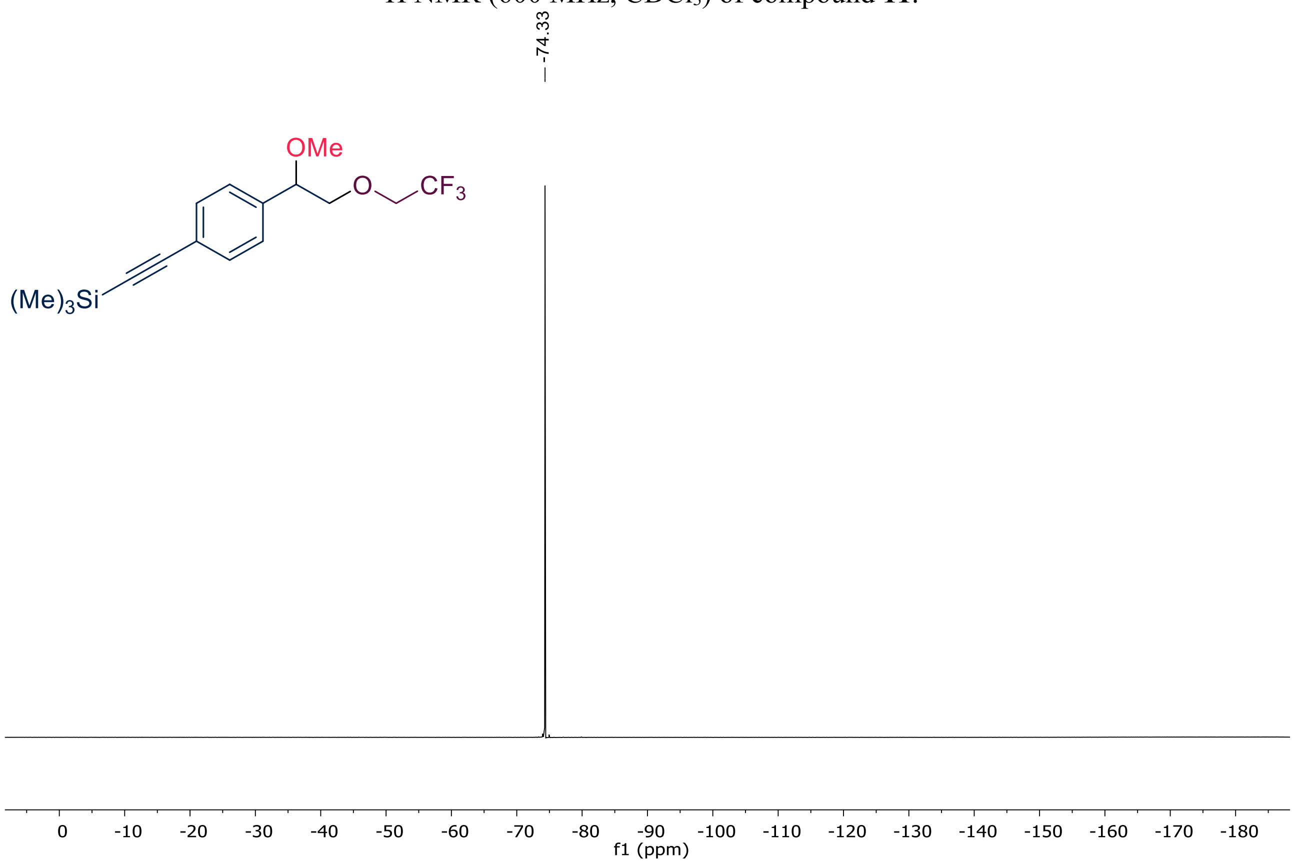

^{19}F NMR (282 MHz, $CDCl_3$) of compound **11**.

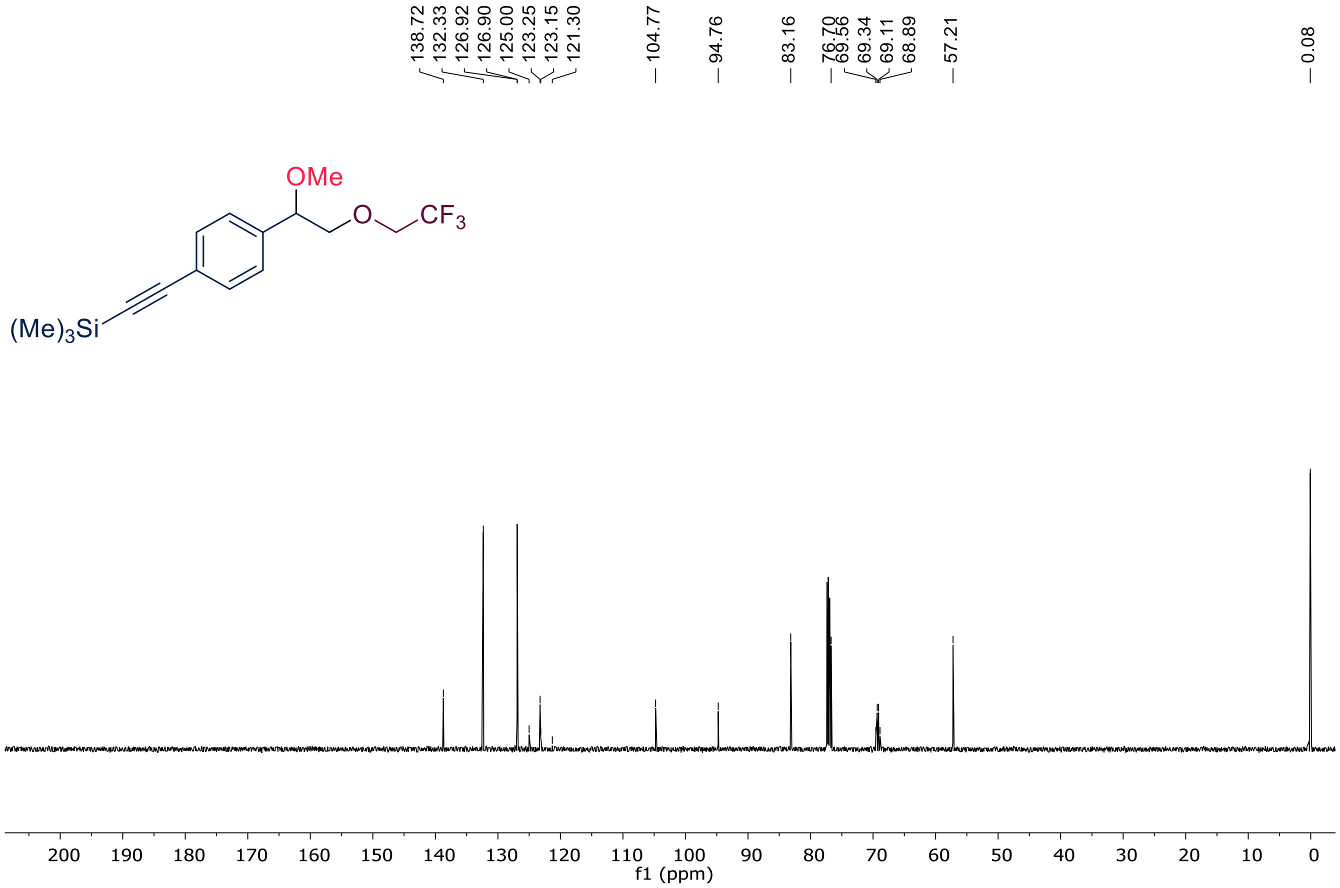

^{13}C NMR (151 MHz, $CDCl_3$) of compound **11**.

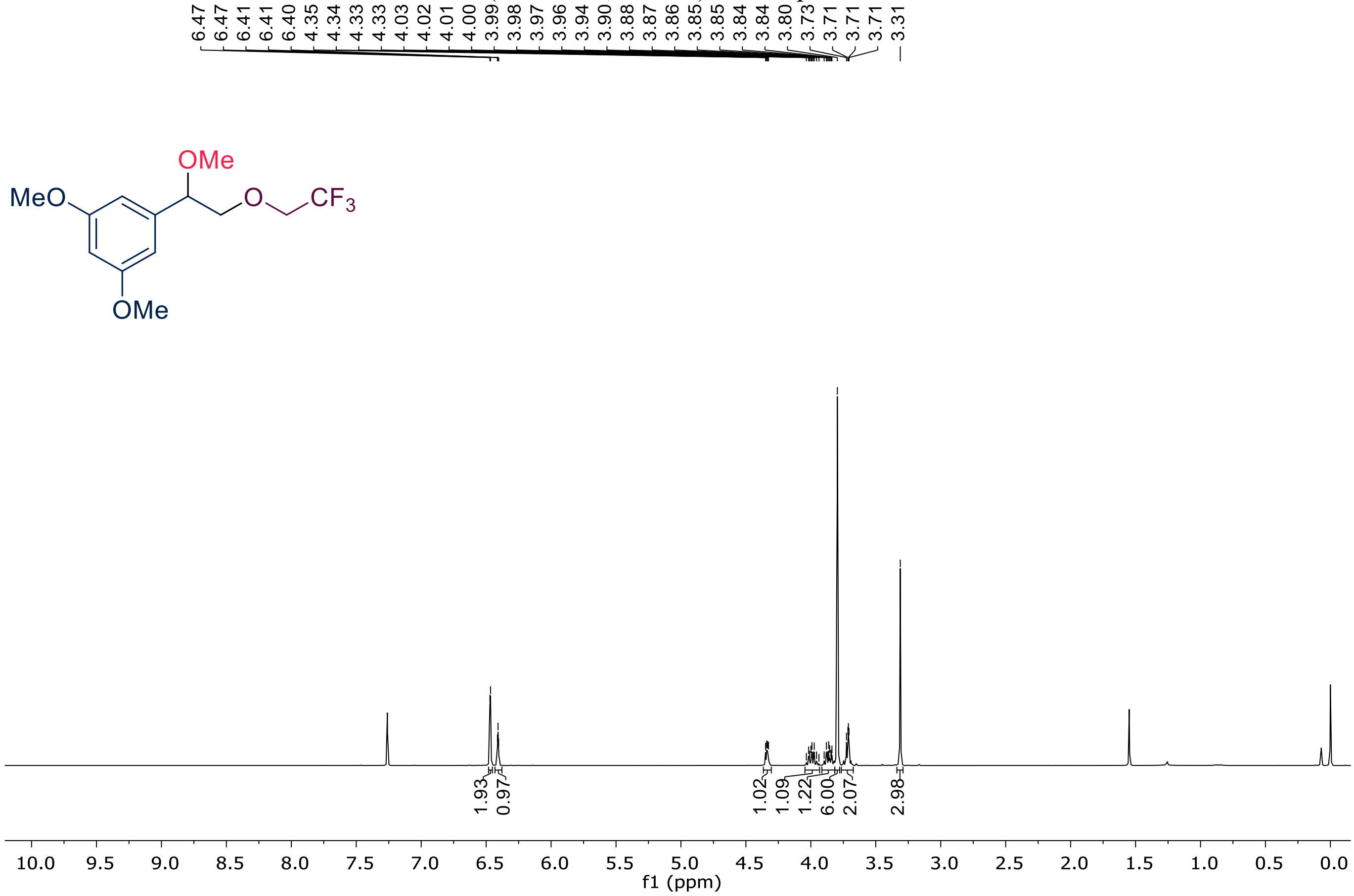

^1H NMR (400 MHz, $CDCl_3$) of compound **12**.

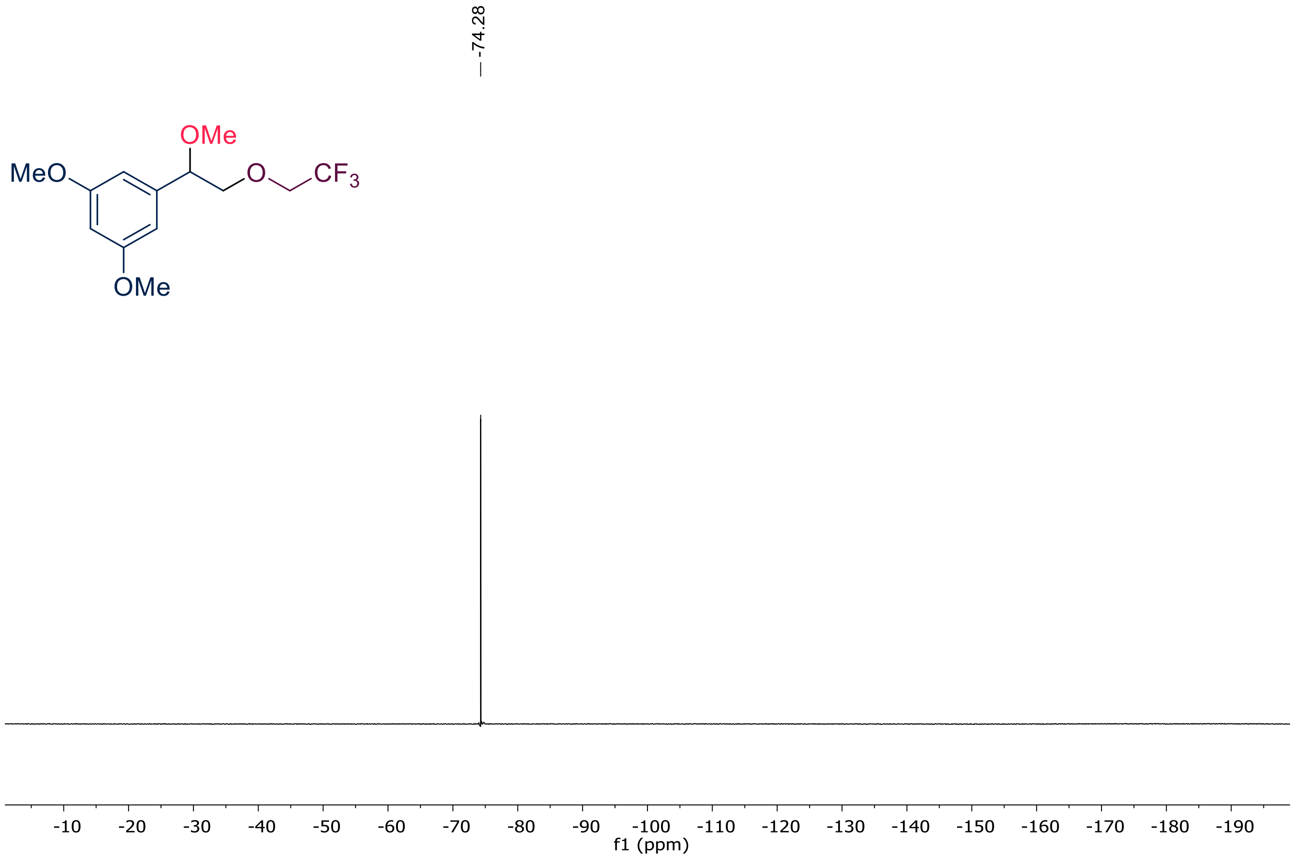

^{19}F NMR (282 MHz, $CDCl_3$) of compound **12**.

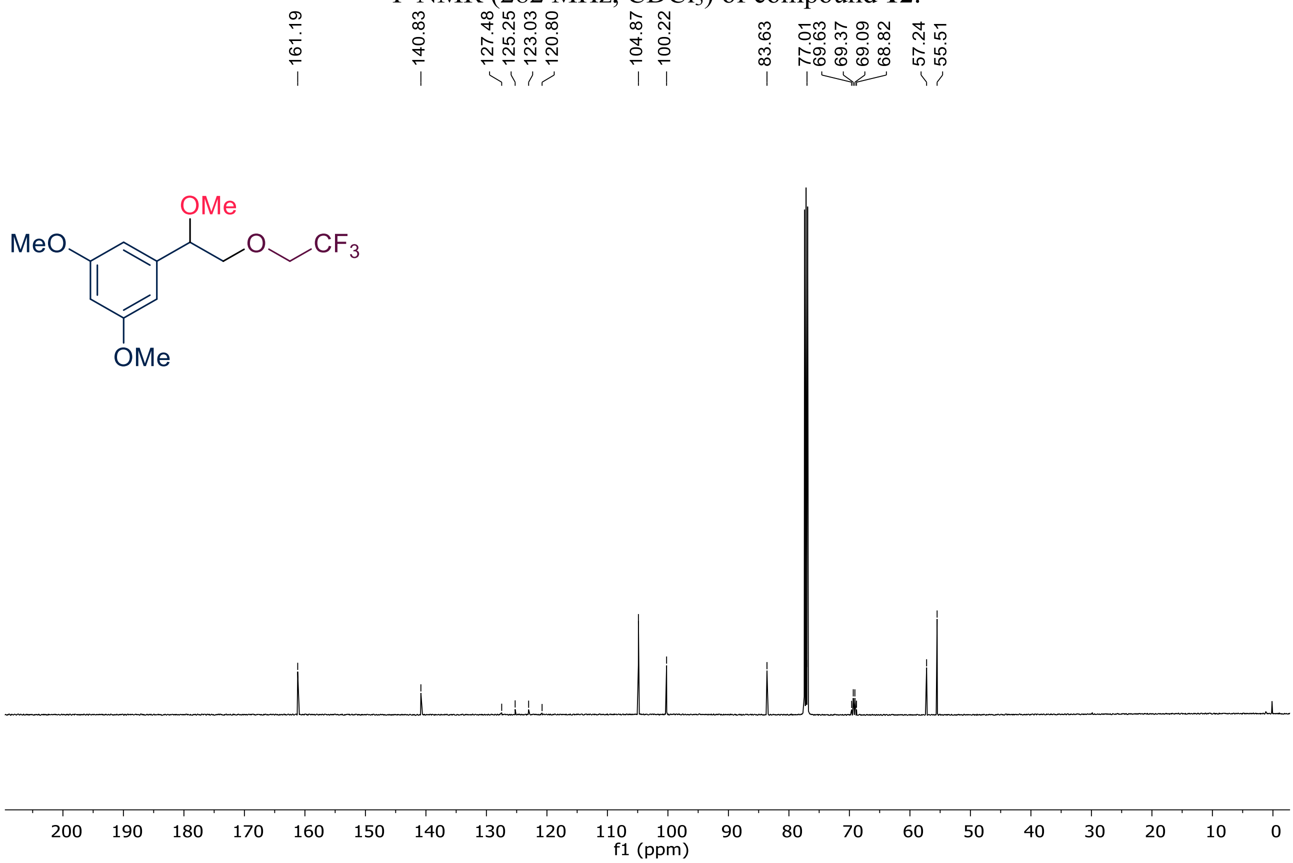

^{13}C NMR (126 MHz, $CDCl_3$) of compound **12**.

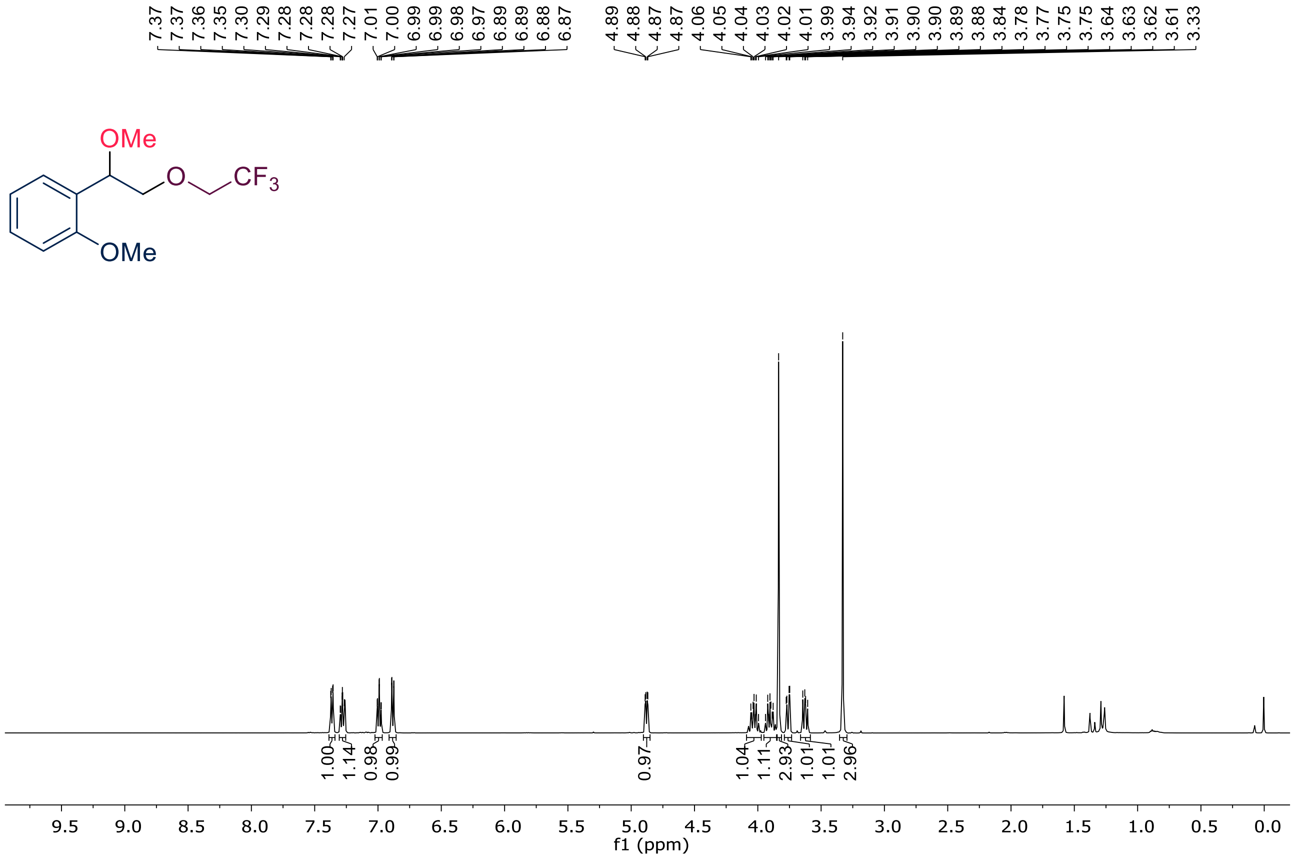

^{1}H NMR (400 MHz, $CDCl_3$) of compound **13**.

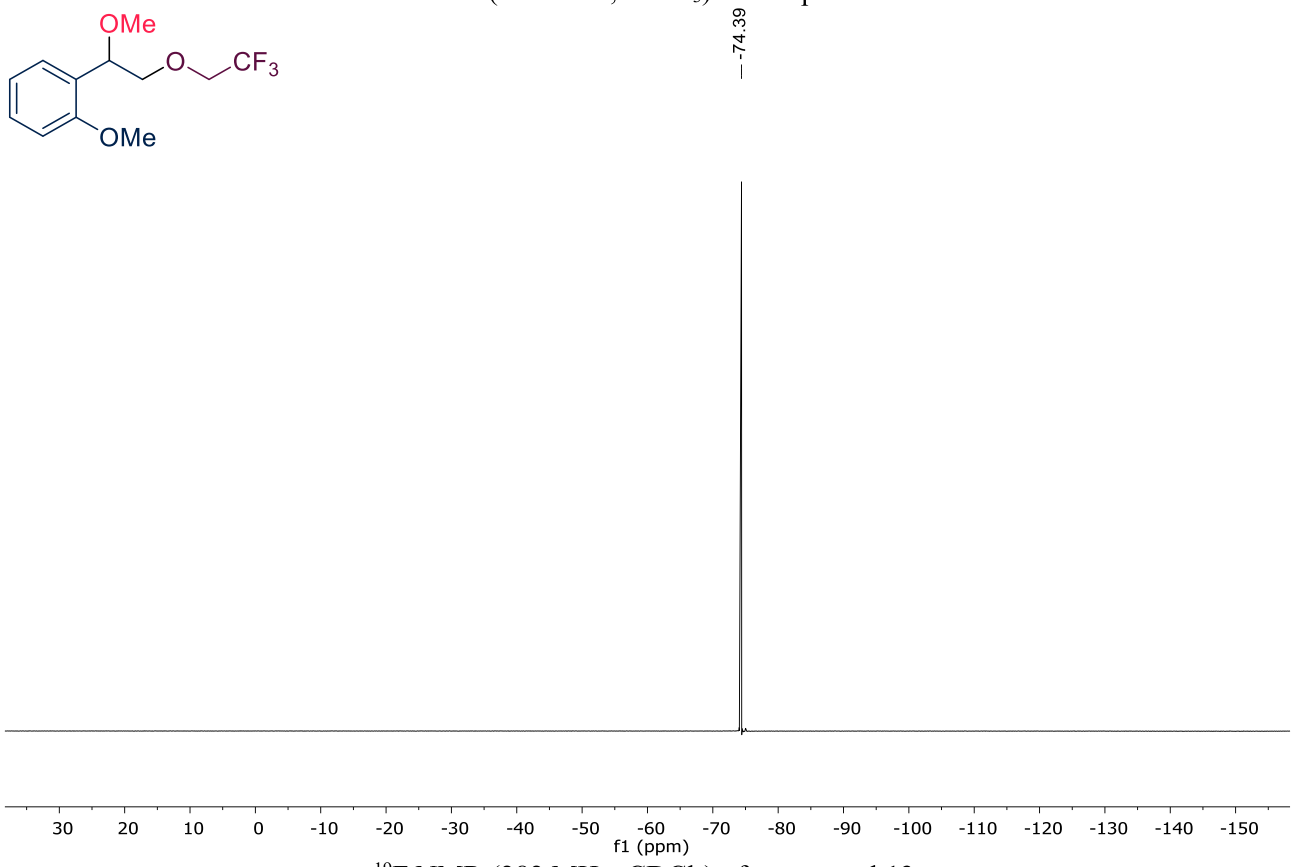

^{19}F NMR (282 MHz, $CDCl_3$) of compound **13**.

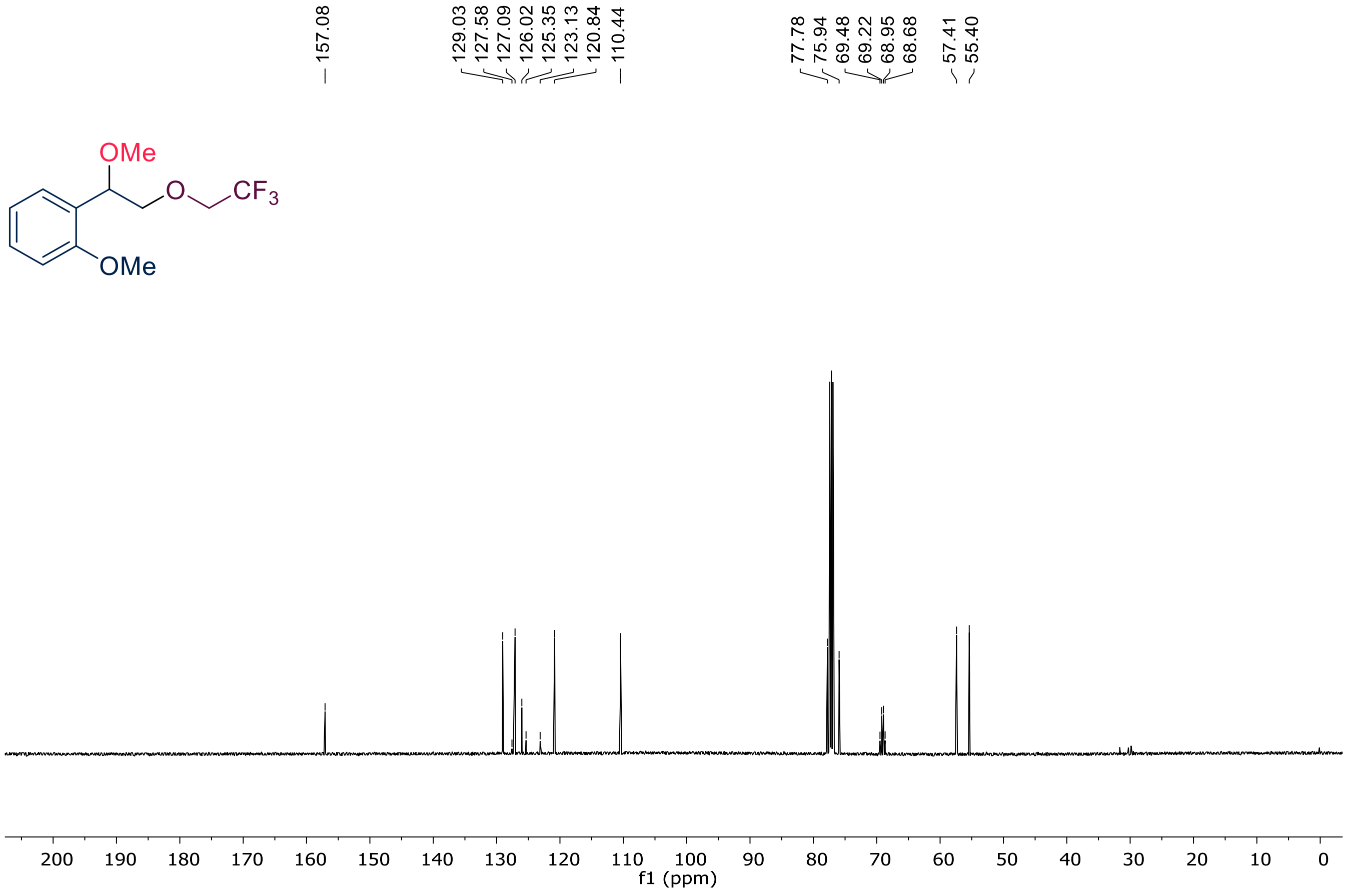

^{13}C NMR (126 MHz, $CDCl_3$) of compound **13**.

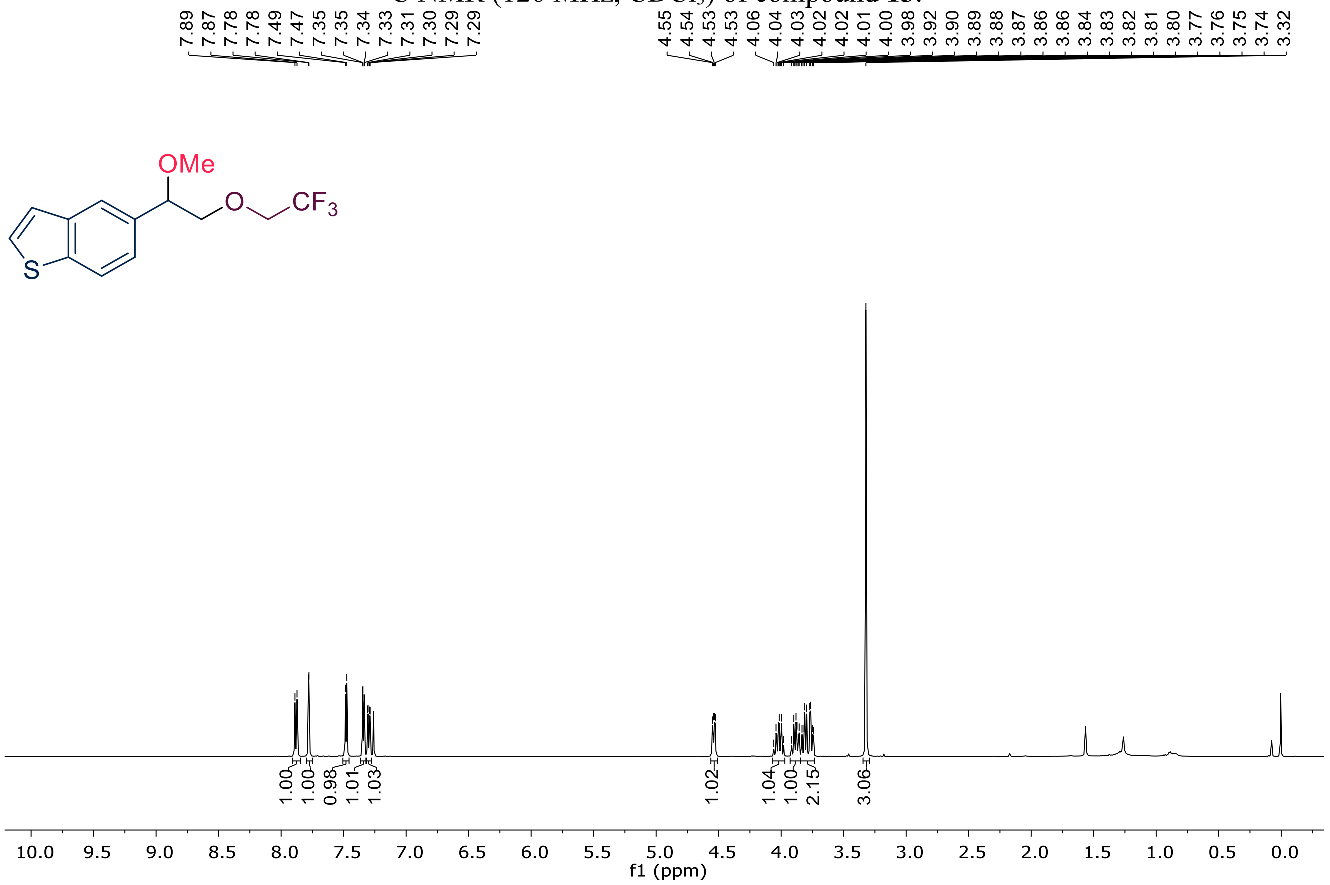

^{1}H NMR (500 MHz, $CDCl_3$) of compound **14**.

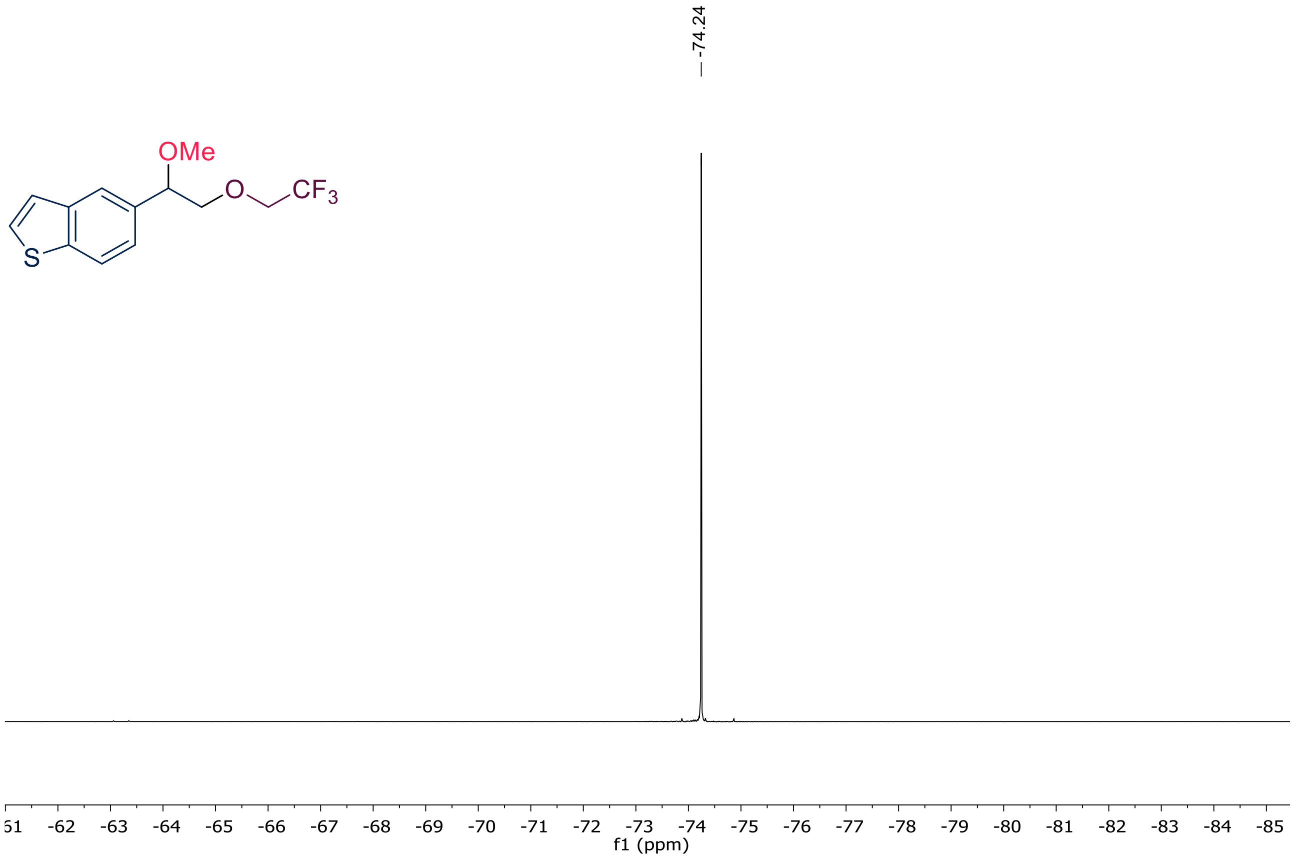

^{19}F NMR (282 MHz, $CDCl_3$) of compound **14**.

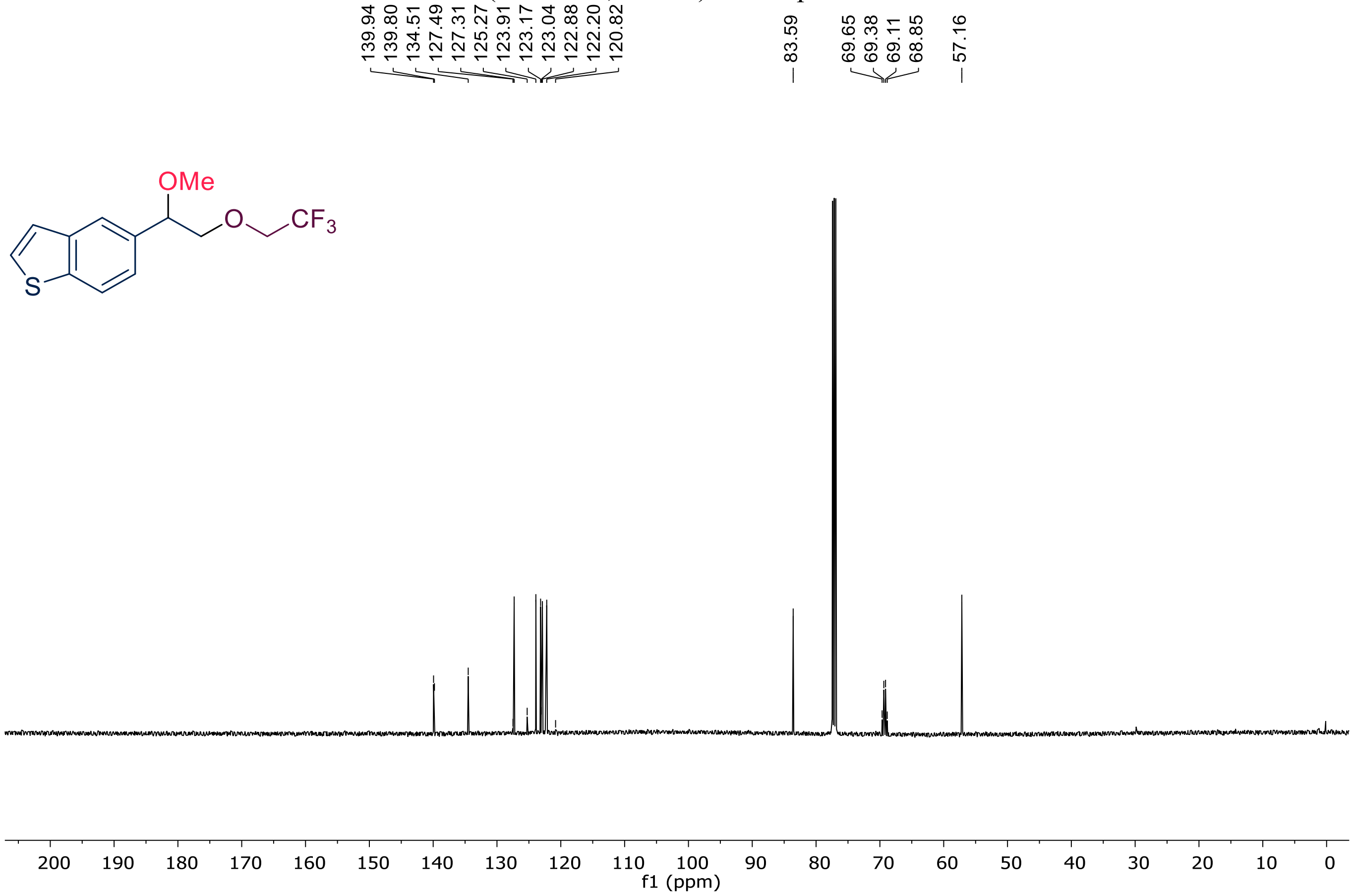

^{13}C NMR (126 MHz, $CDCl_3$) of compound **14**.

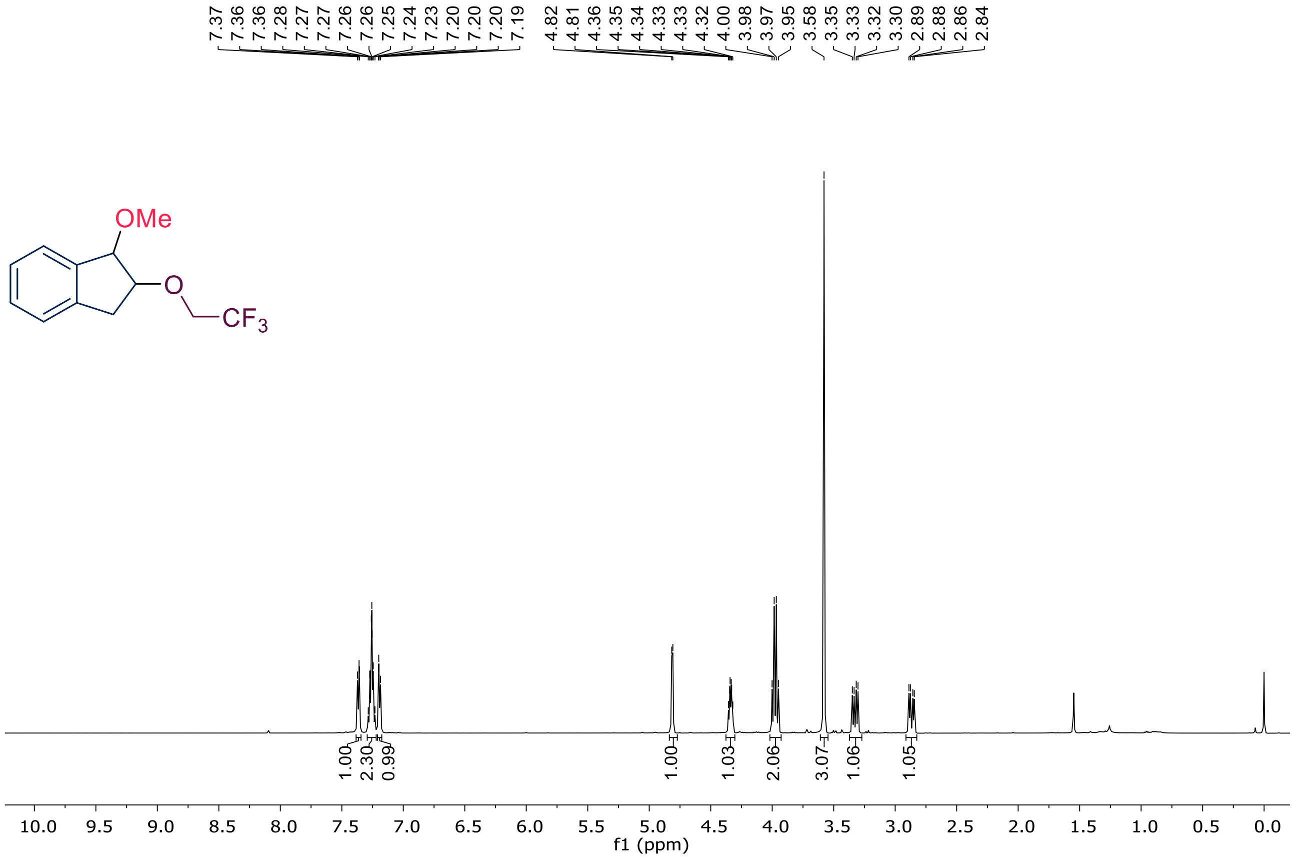

^{1}H NMR (500 MHz, $CDCl_3$) of compound **15-*trans***.

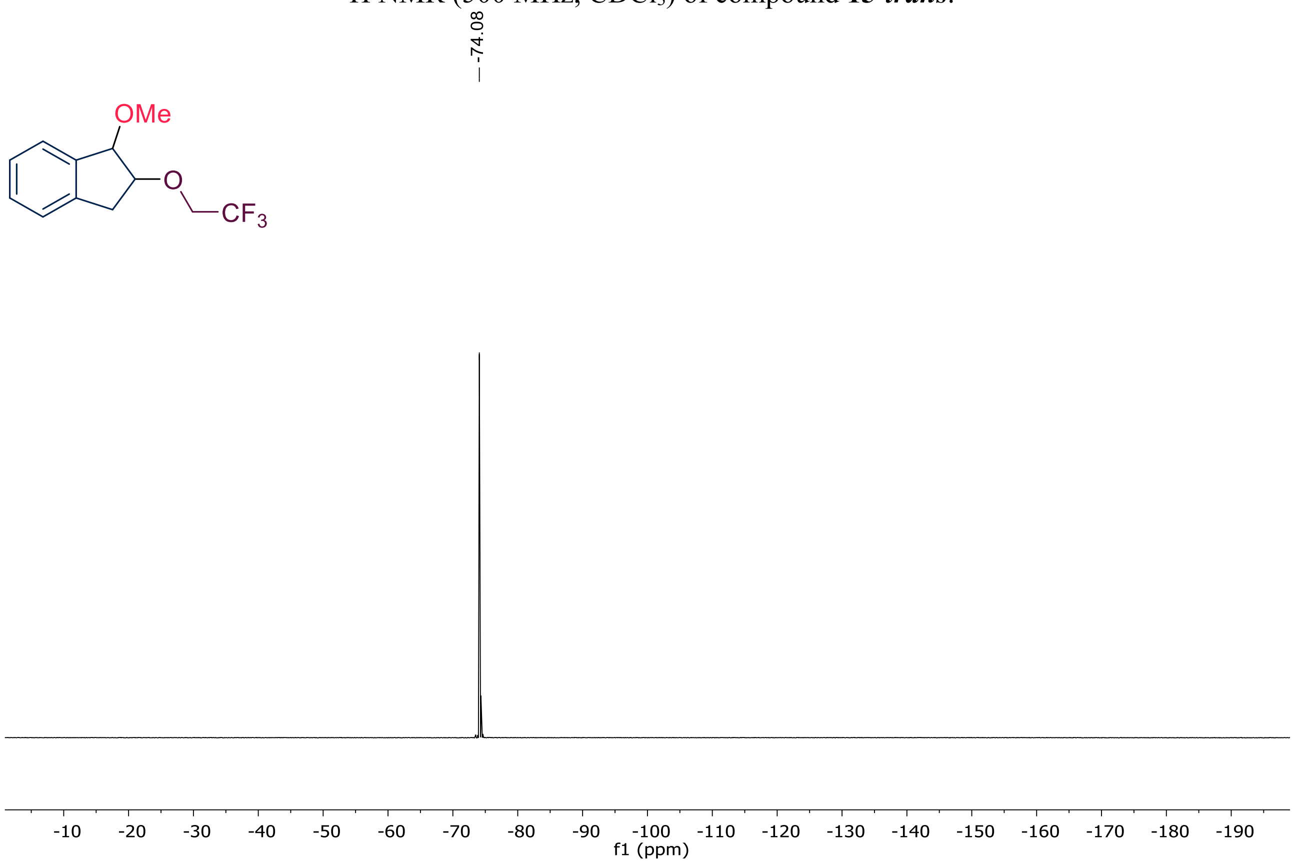

^{19}F NMR (377 MHz, $CDCl_3$) of compound **15-*trans***.

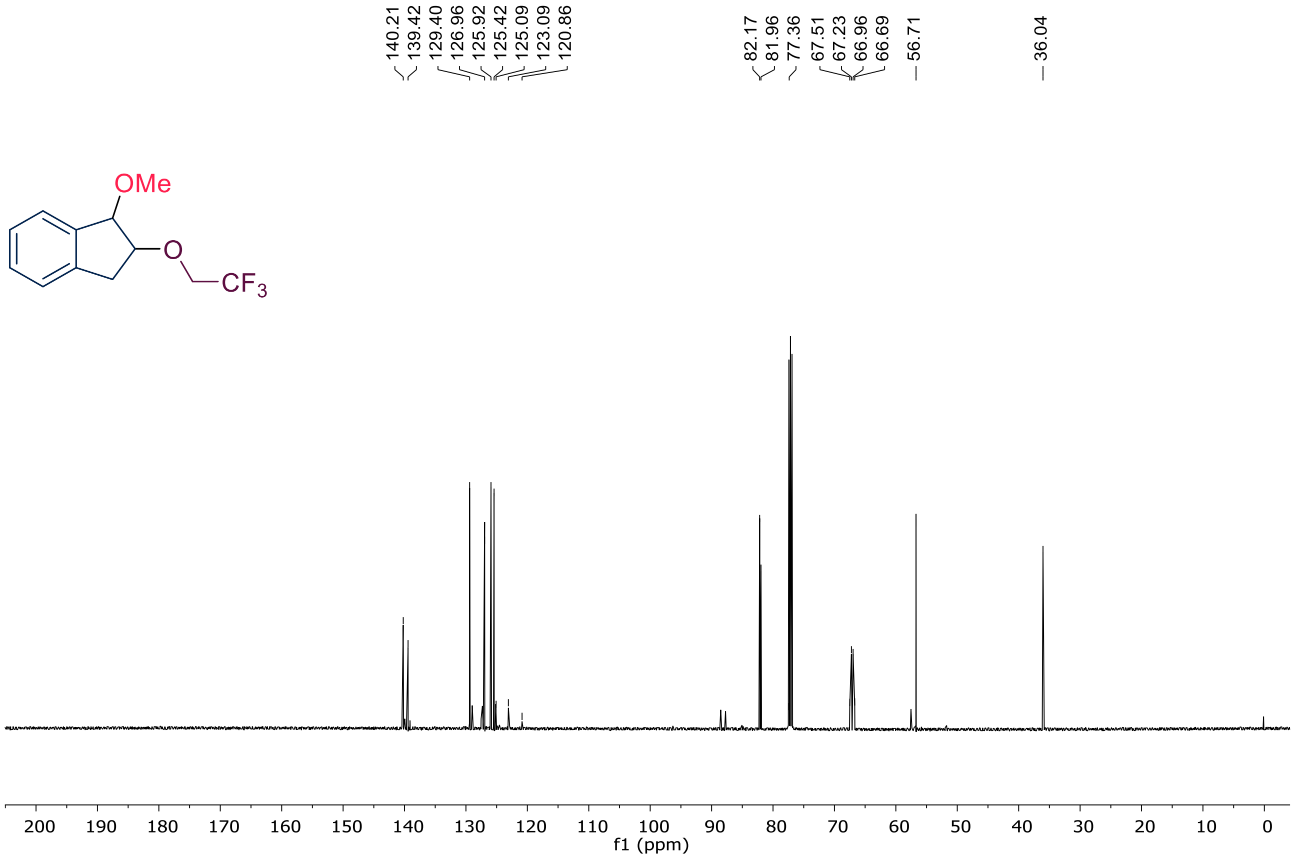

^{13}C NMR (126 MHz, CDCl$_3$) of compound **15-*trans***.

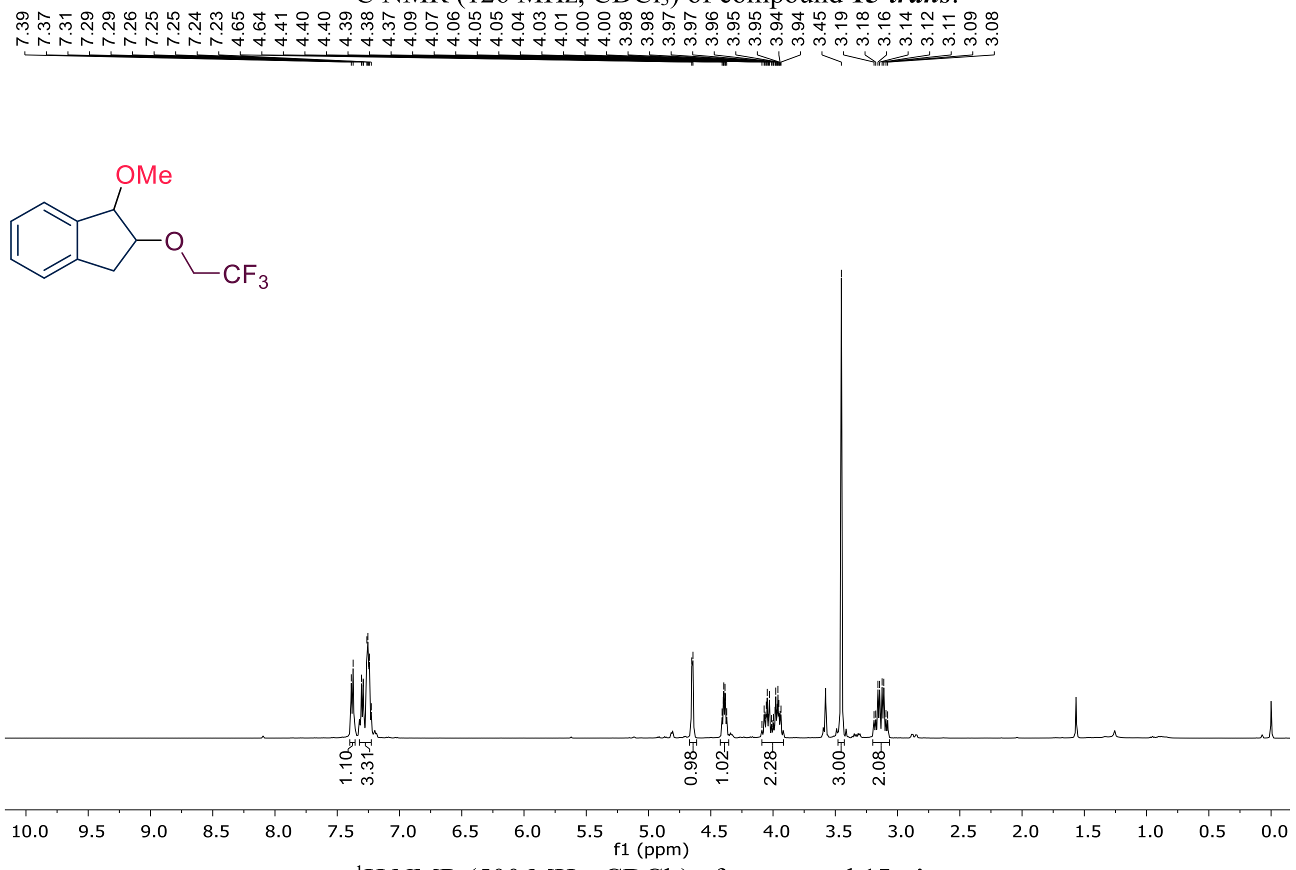

^{1}H NMR (500 MHz, CDCl$_3$) of compound **15-*cis***.

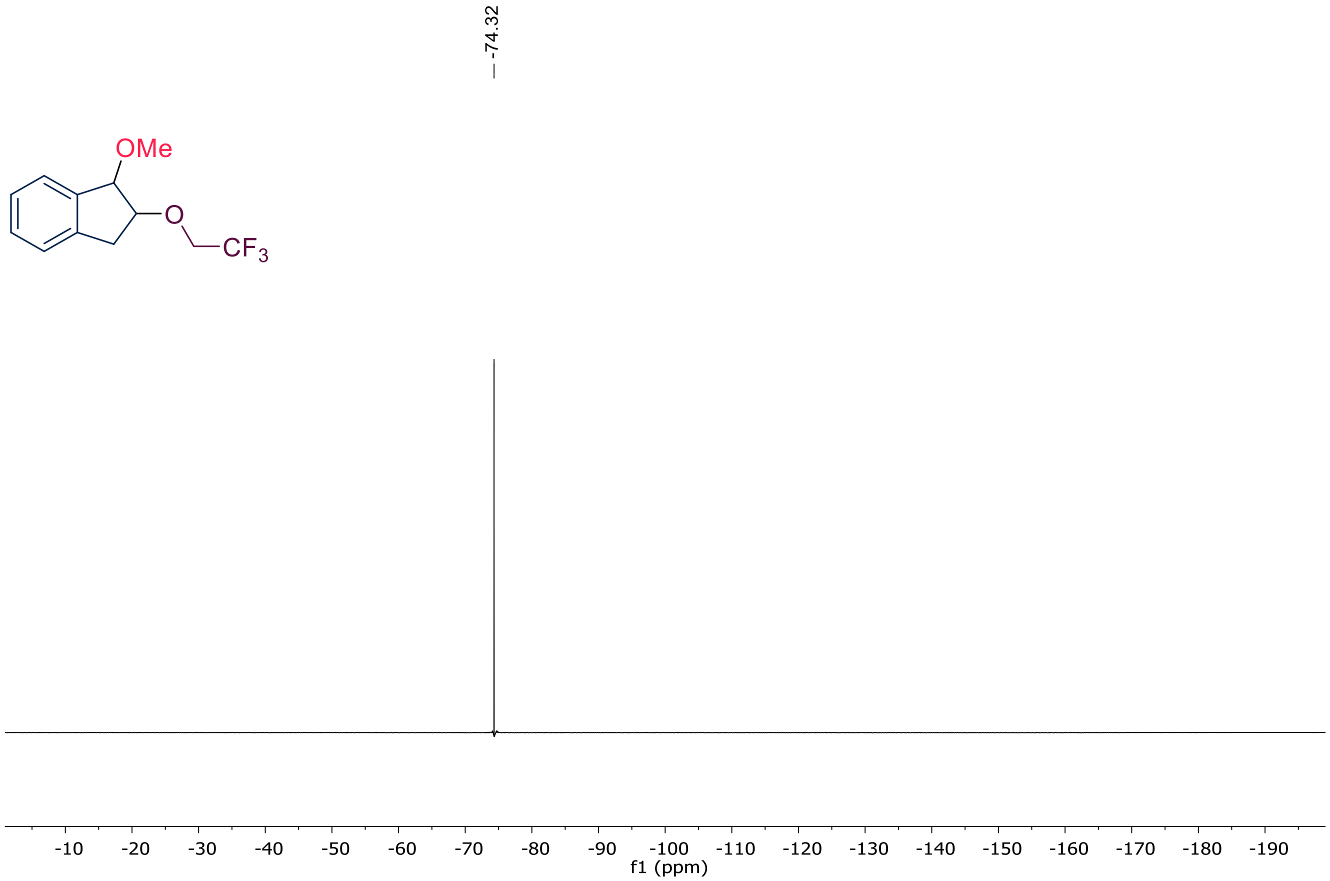

^{19}F NMR (377 MHz, $CDCl_3$) of compound **15-*cis***.

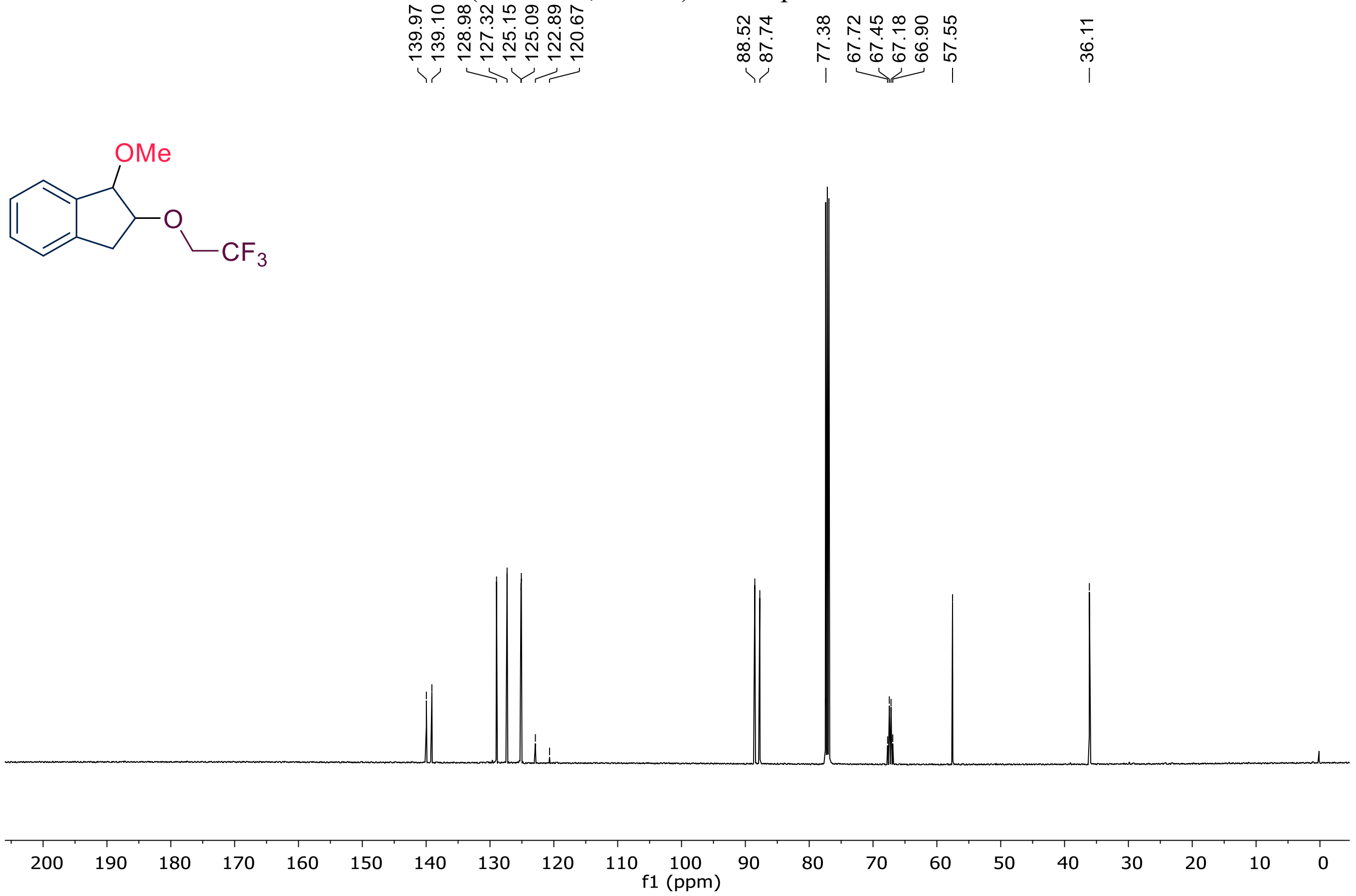

^{13}C NMR (126 MHz, $CDCl_3$) of compound **15-*cis***.

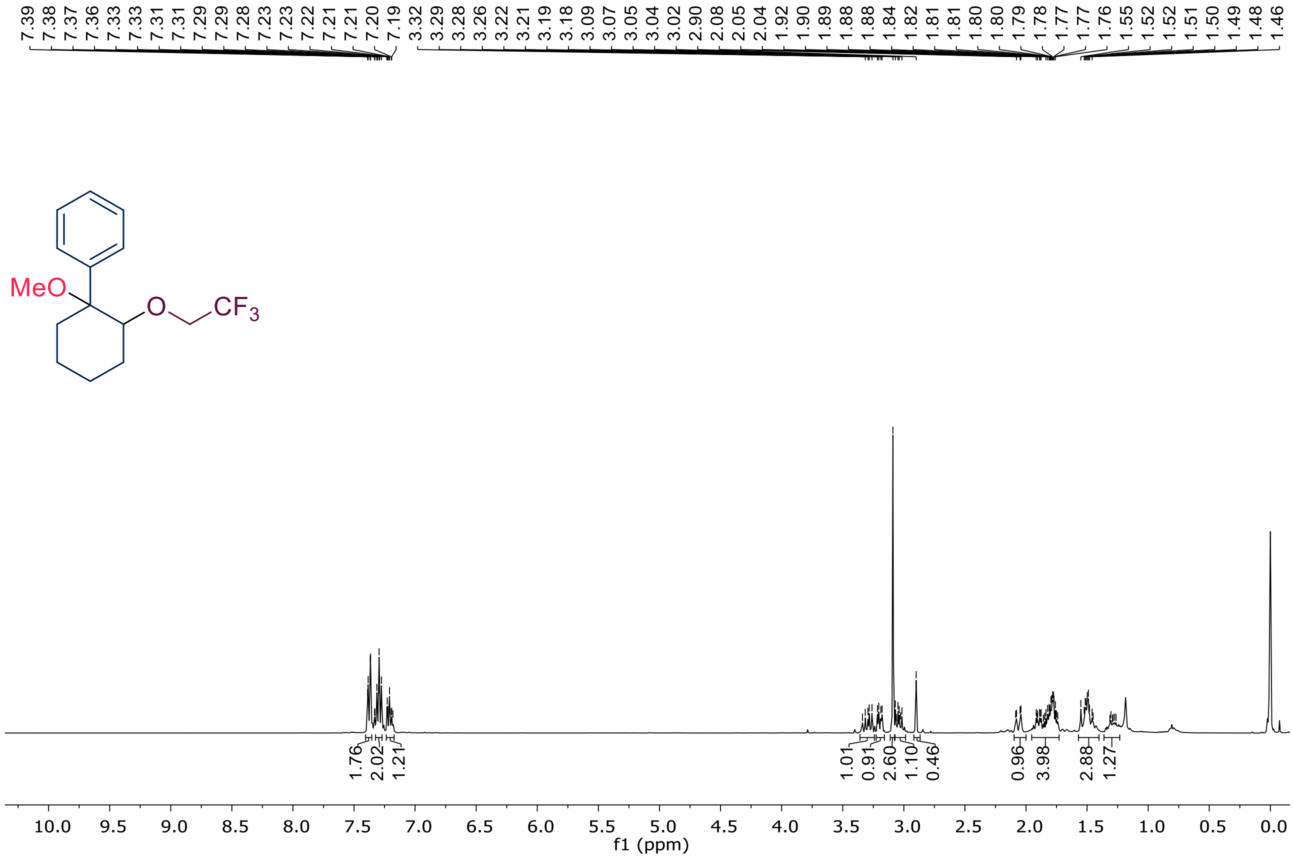

^{1}H NMR (400 MHz, $CDCl_3$) of compound **16**.

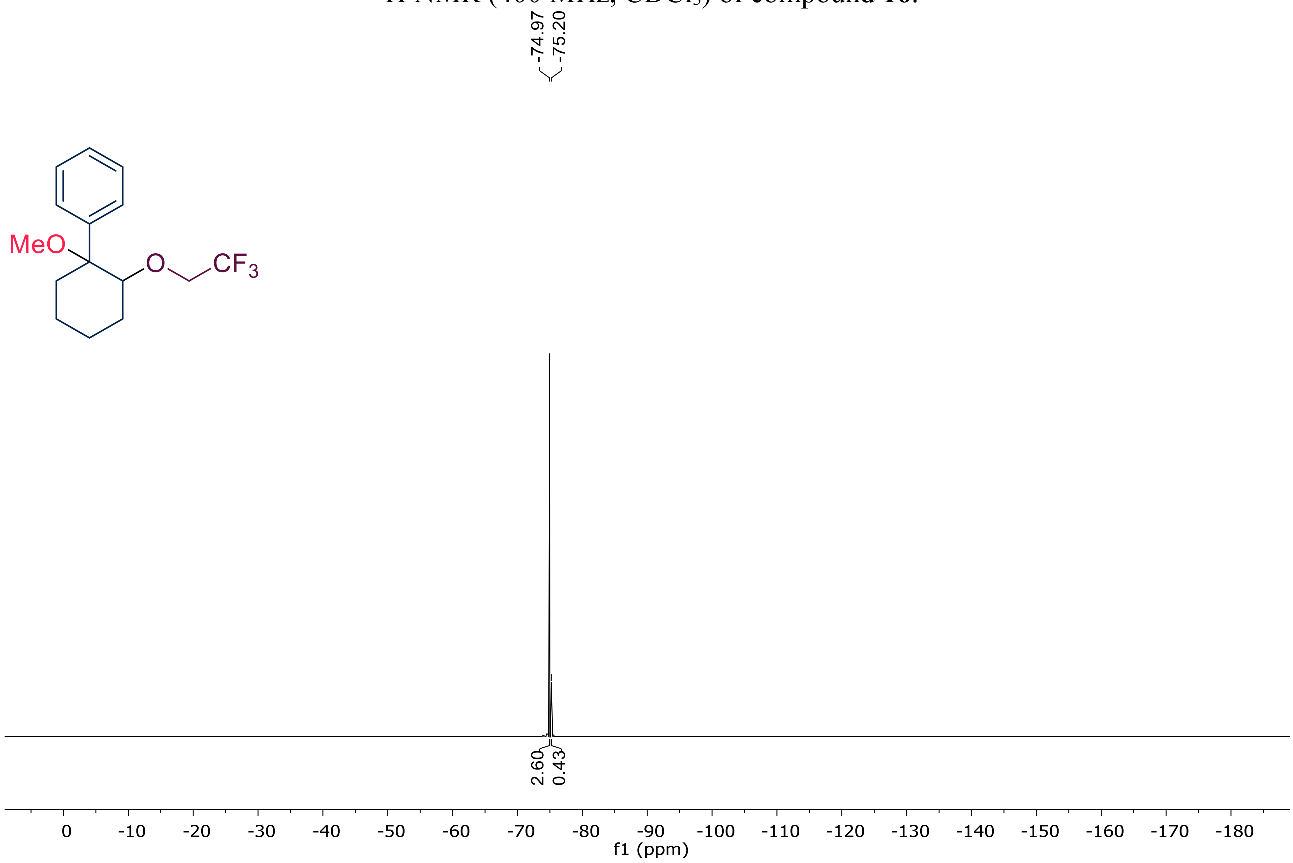

^{19}F NMR (377 MHz, $CDCl_3$) of compound **16**.

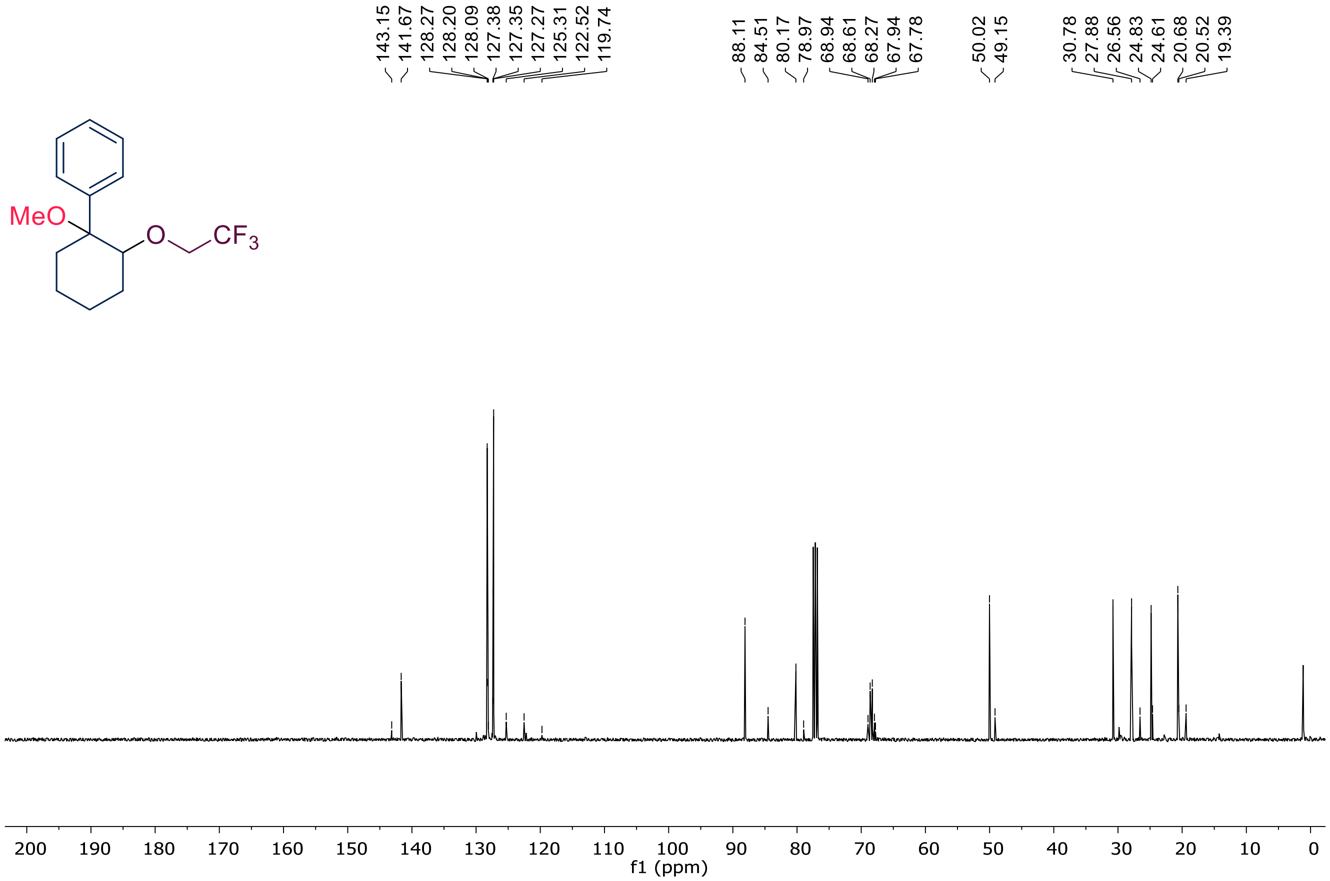

^{13}C NMR (101 MHz, CDCl$_3$) of compound **16**.

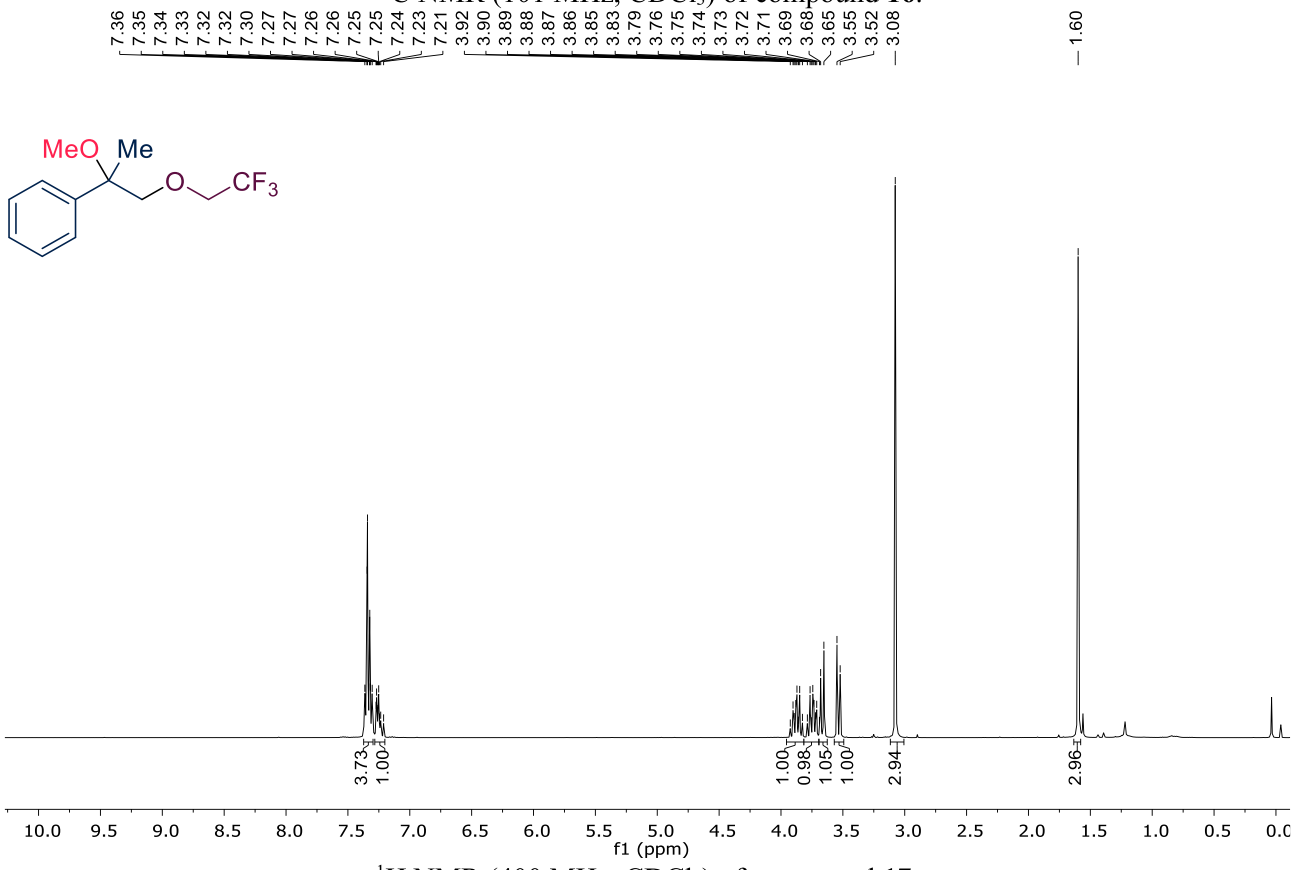

^{1}H NMR (400 MHz, CDCl$_3$) of compound **17**.

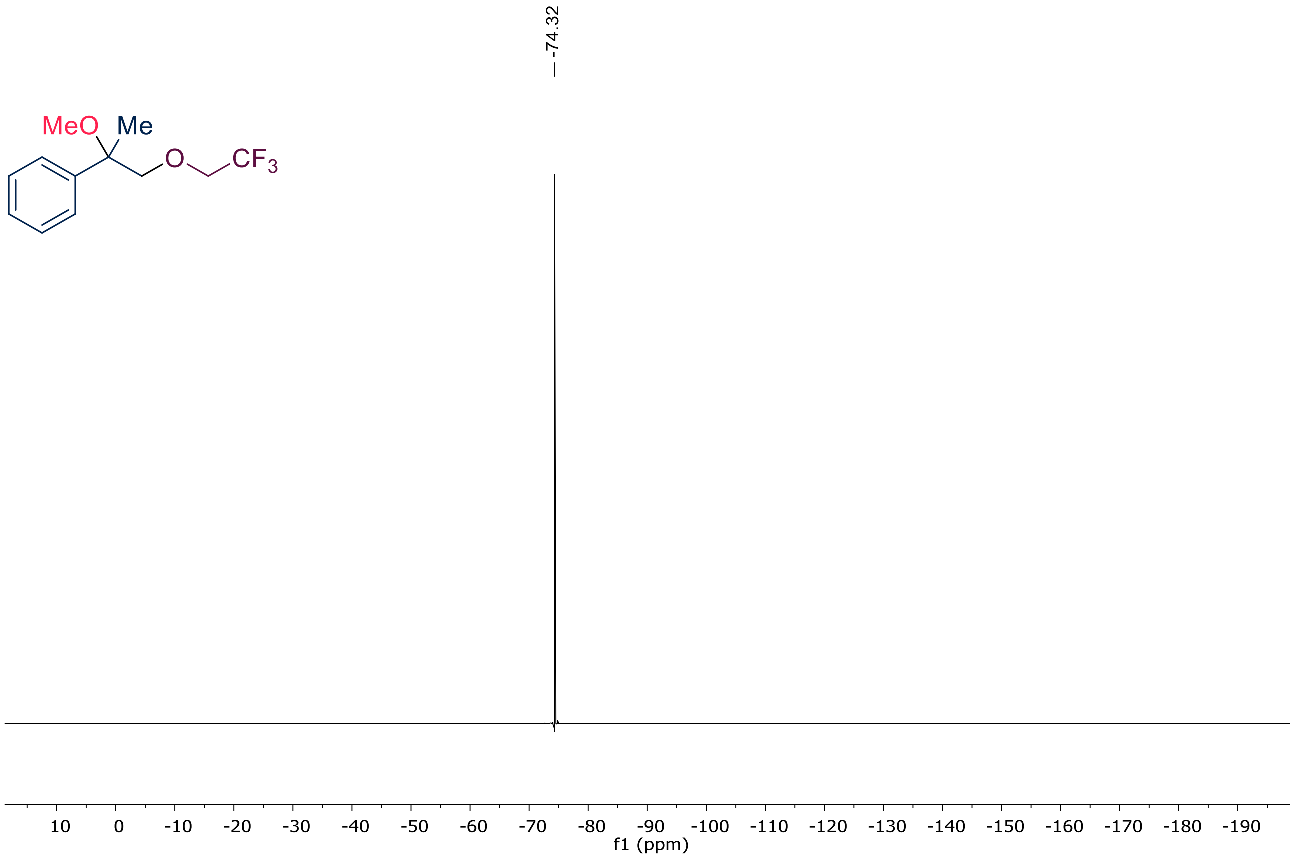

^{19}F NMR (377 MHz, $CDCl_3$) of compound **17**.

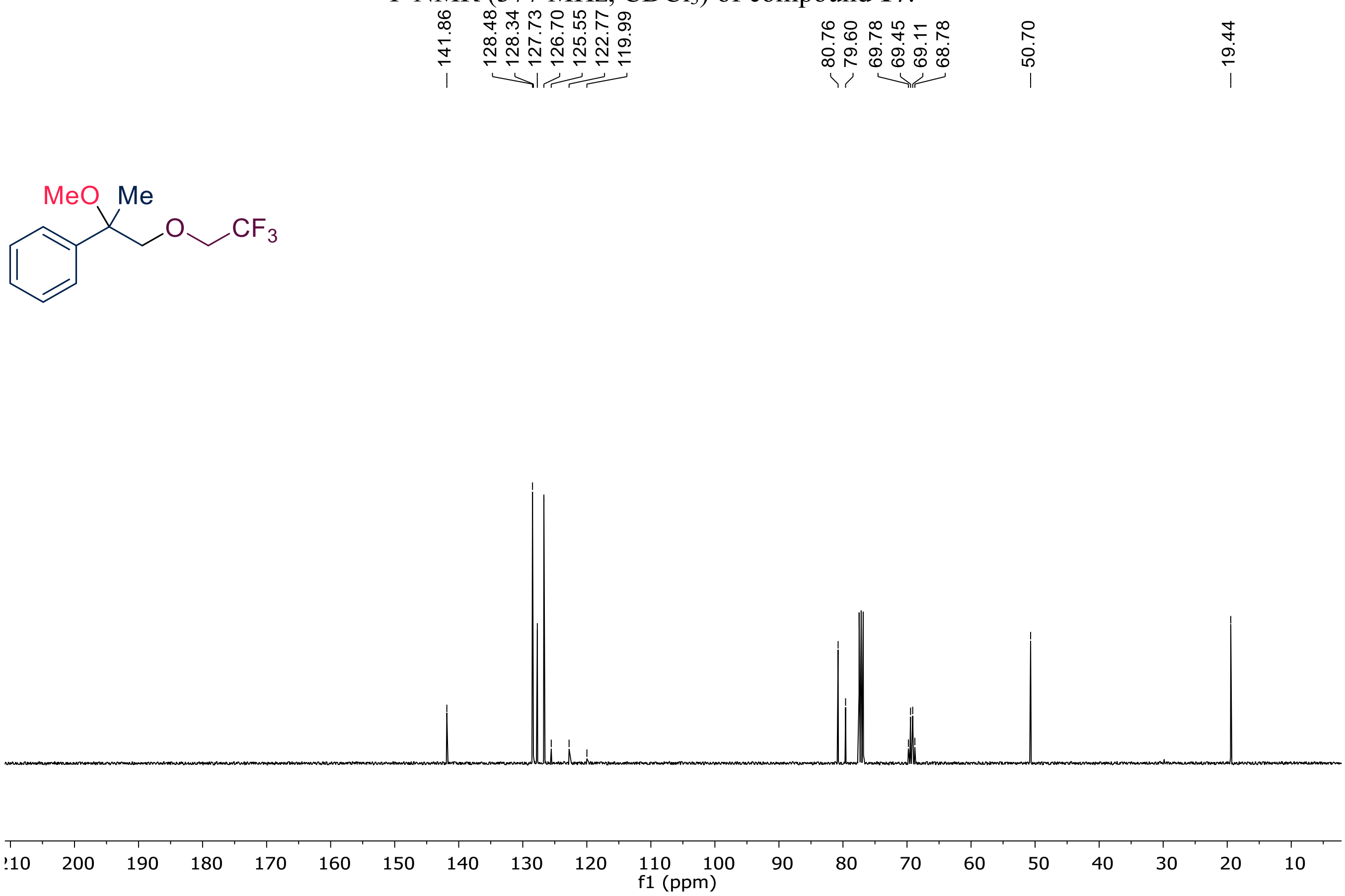

^{13}C NMR (101 MHz, $CDCl_3$) of compound **17**.

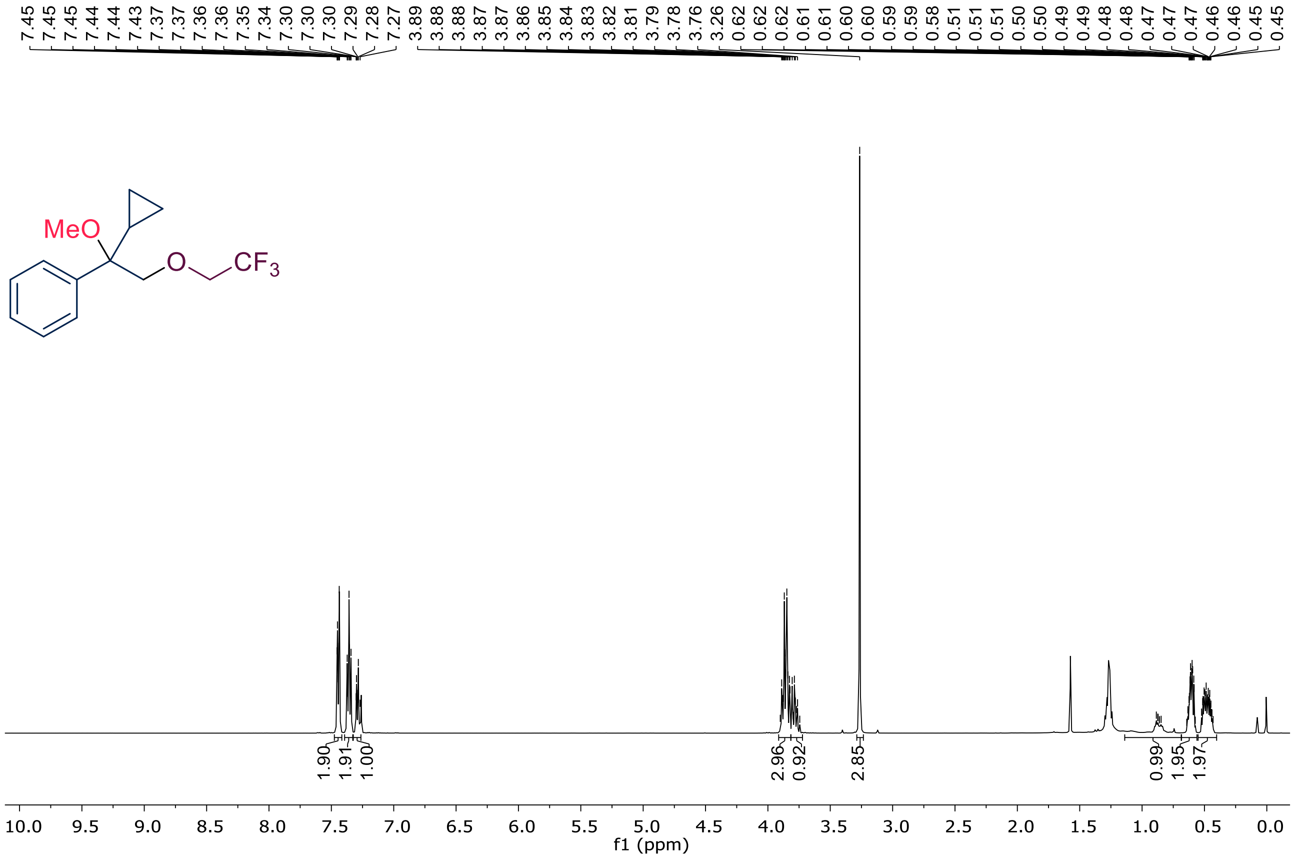

^{1}H NMR (500 MHz, CDCl$_3$) of compound **18**.

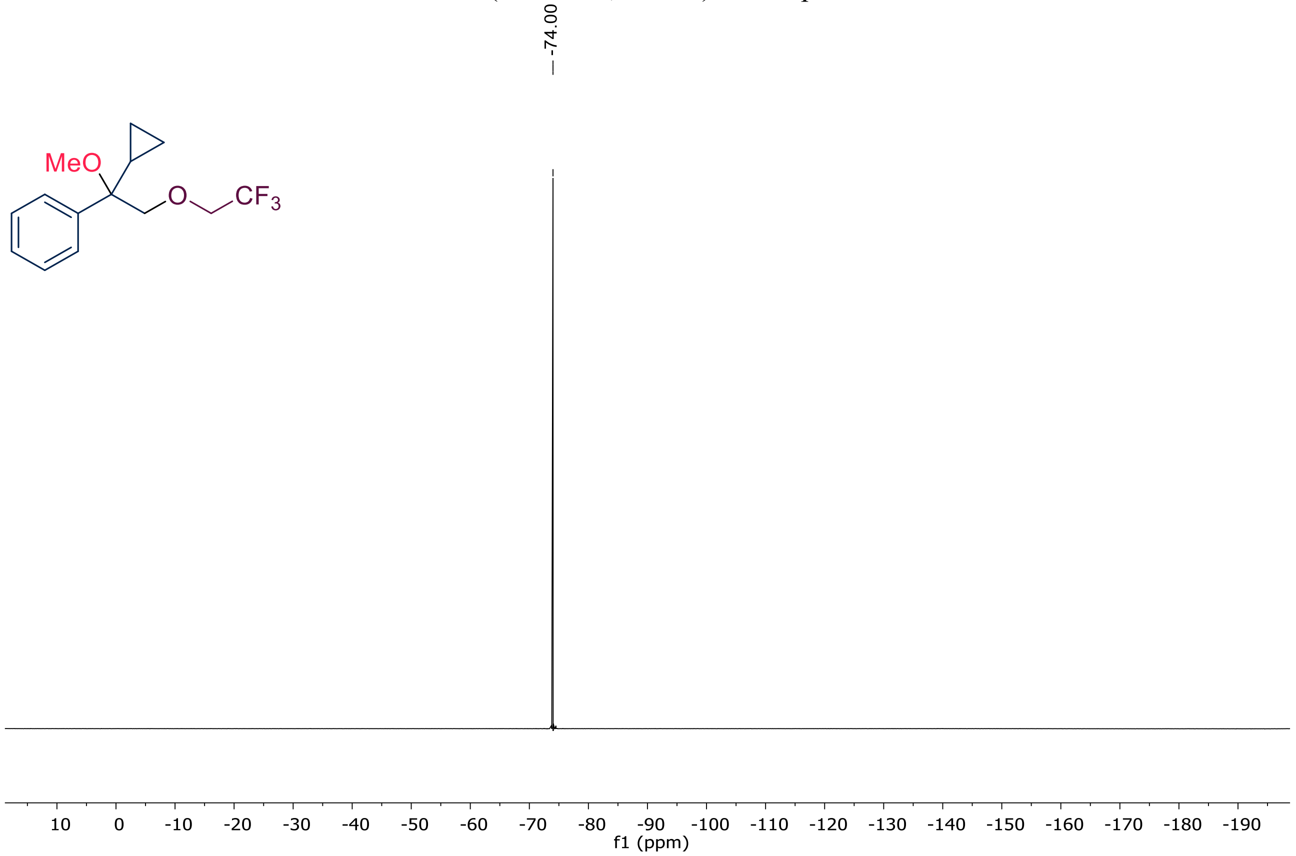

^{19}F NMR (377 MHz, CDCl$_3$) of compound **18**.

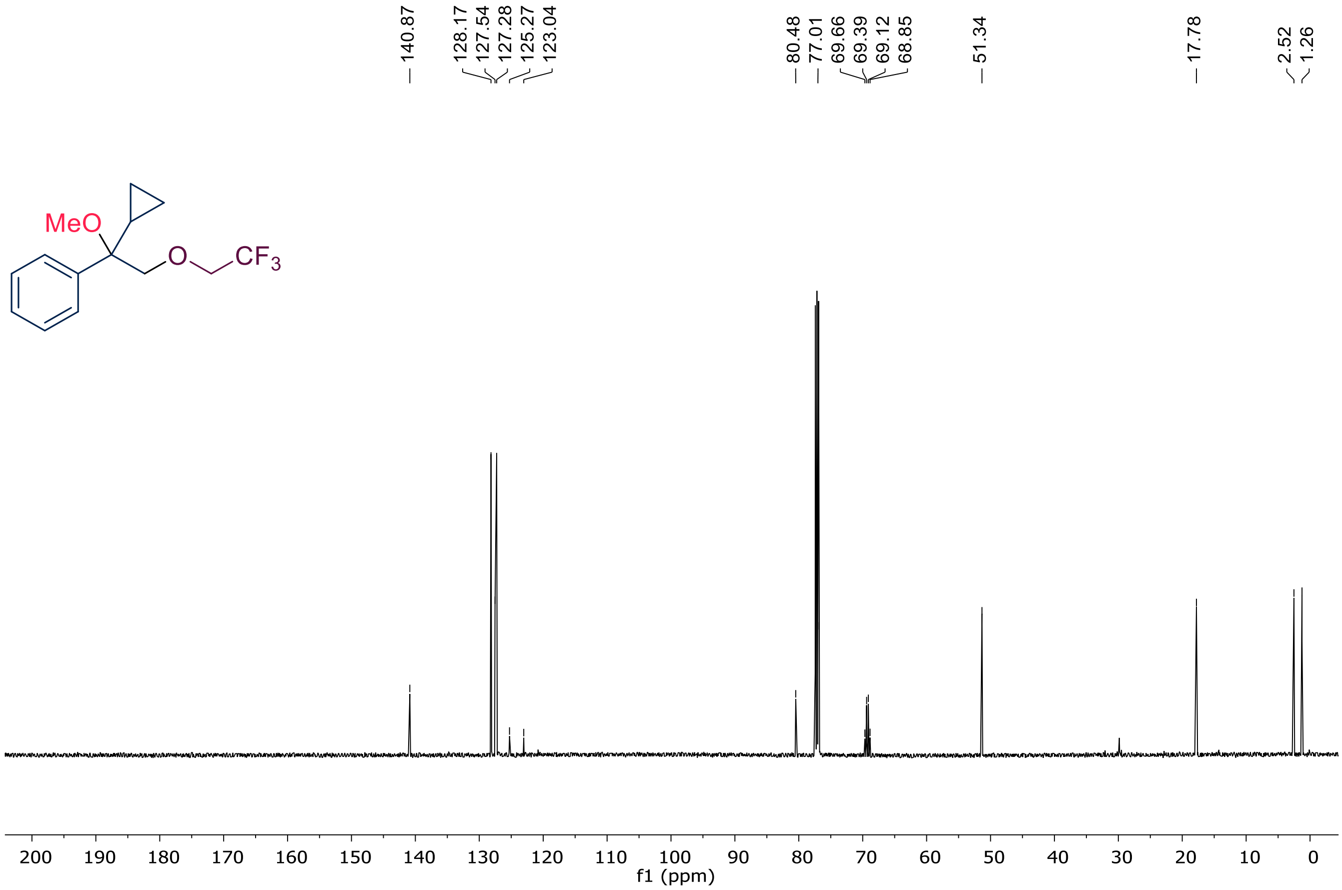

^{13}C NMR (126 MHz, $CDCl_3$) of compound **18**.

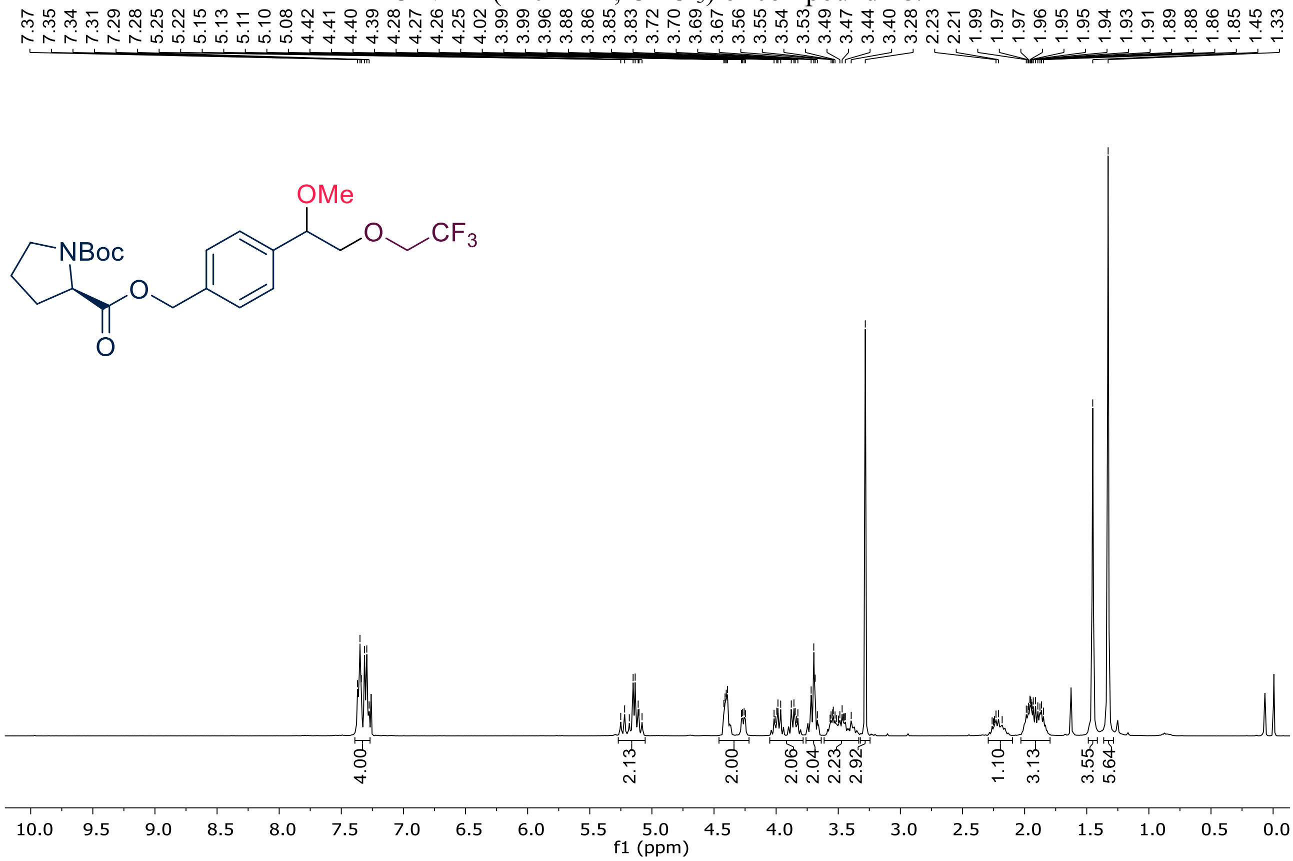

^{1}H NMR (400 MHz, $CDCl_3$) of compound **19**.

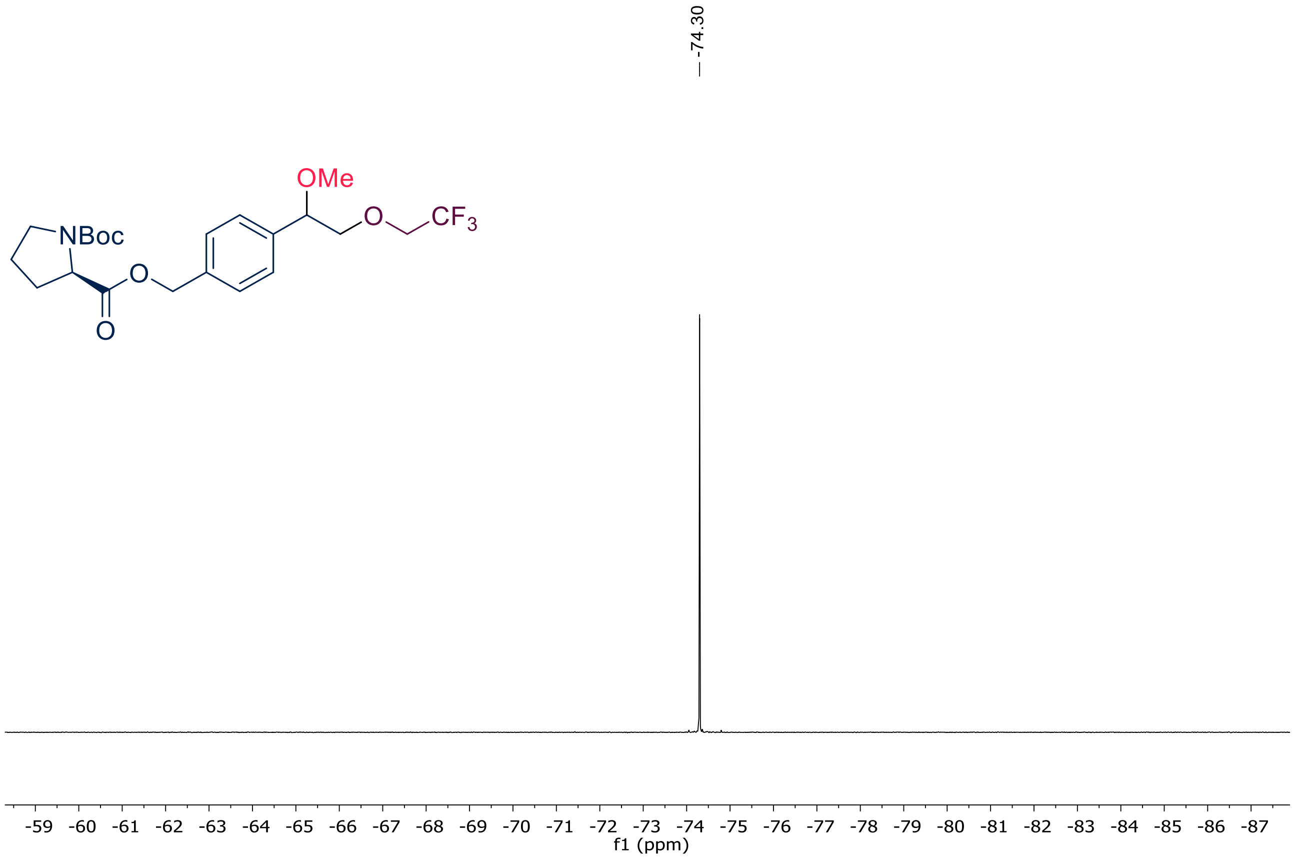

^{19}F NMR (377 MHz, $CDCl_3$) of compound **19**.

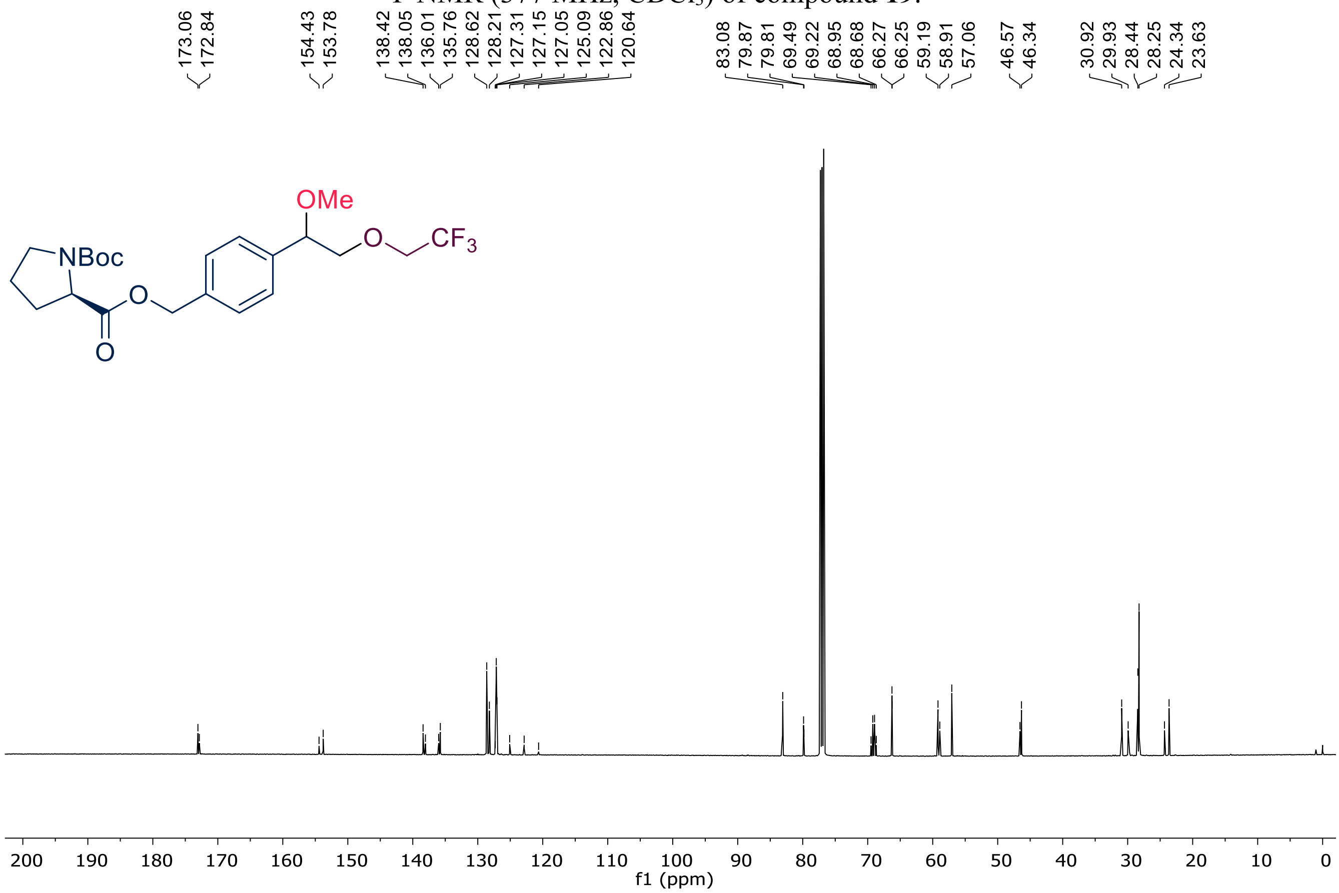

^{13}C NMR (101 MHz, $CDCl_3$) of compound **19**.

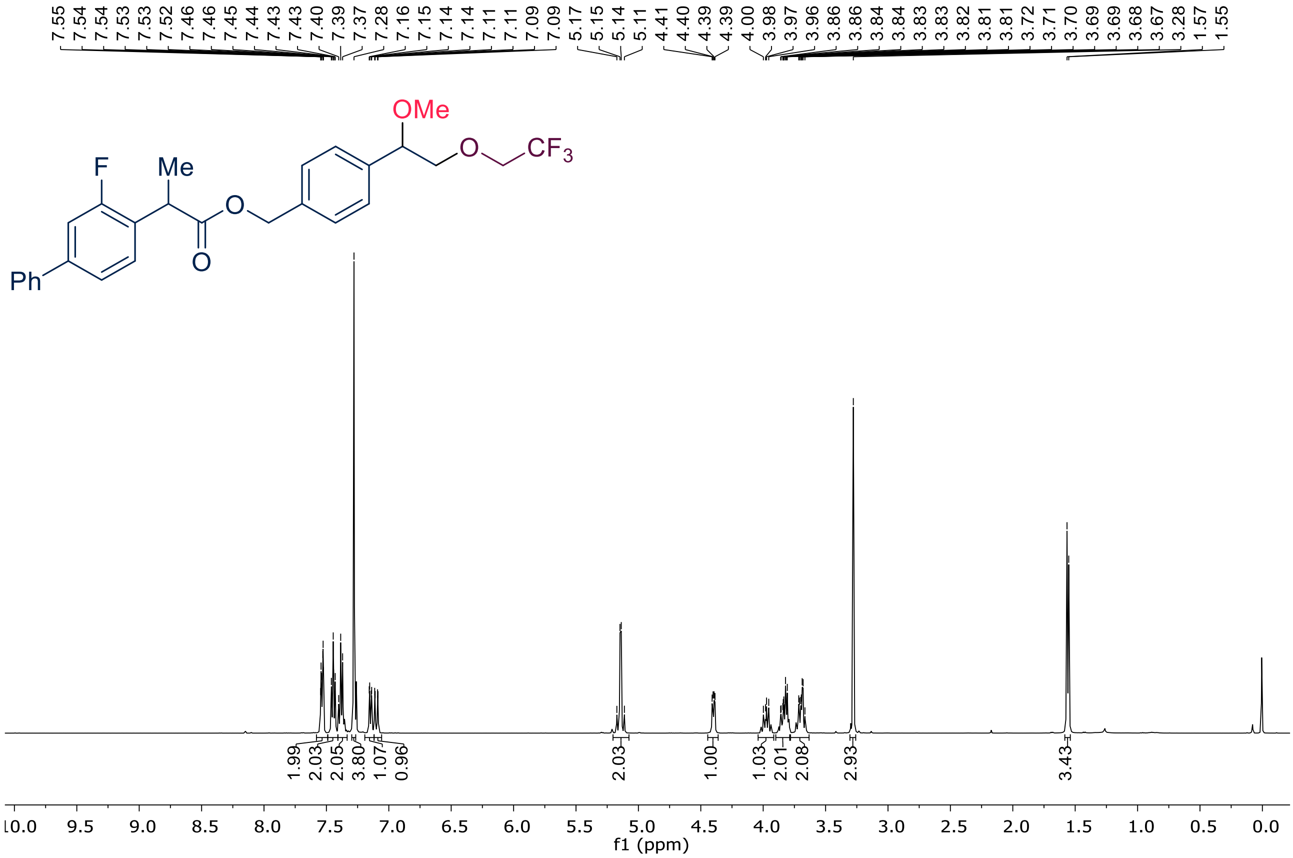

^{1}H NMR (500 MHz, CDCl$_3$) of compound **20**.

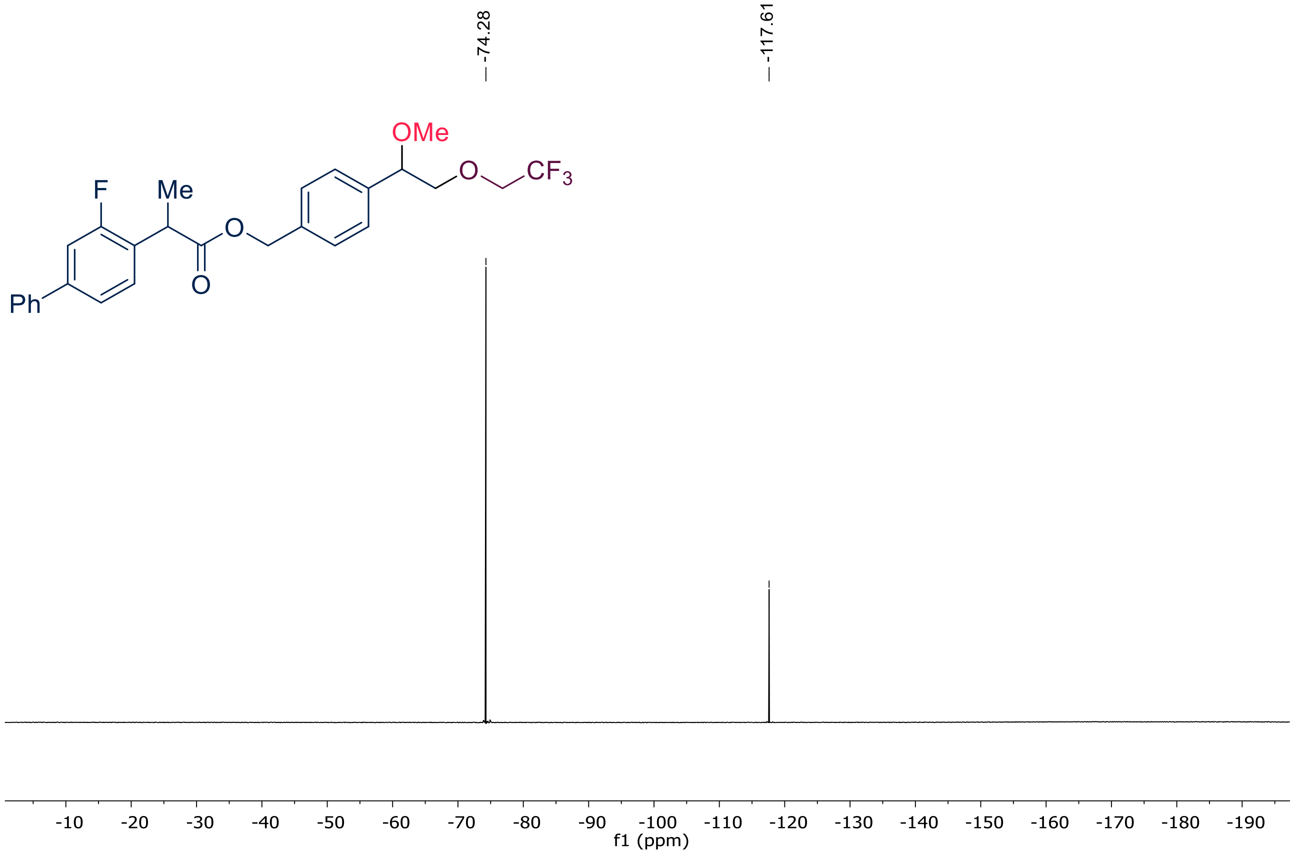

^{19}F NMR (282 MHz, CDCl$_3$) of compound **20**.

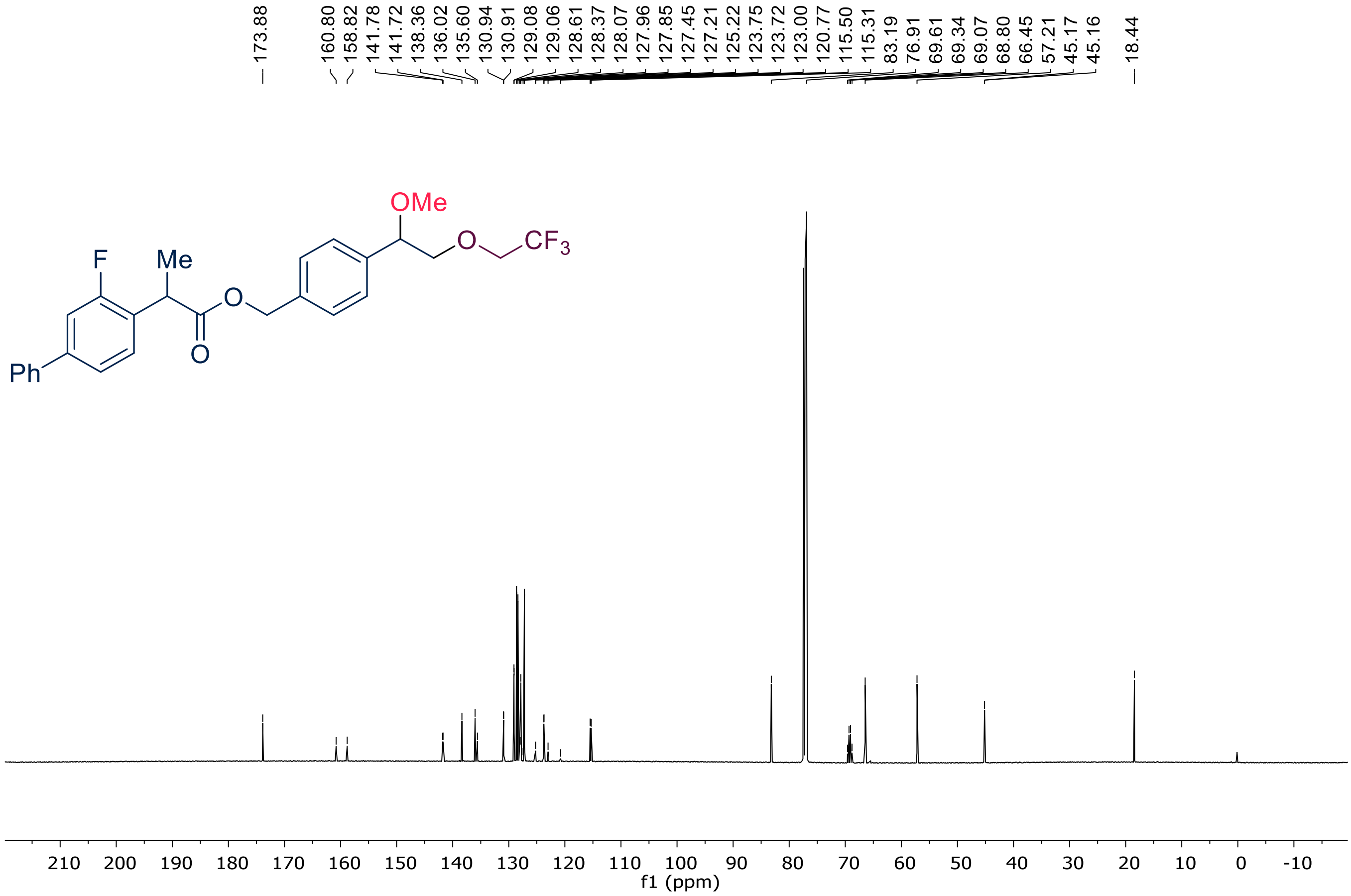

^{13}C NMR (126 MHz, CDCl$_3$) of compound **20**.

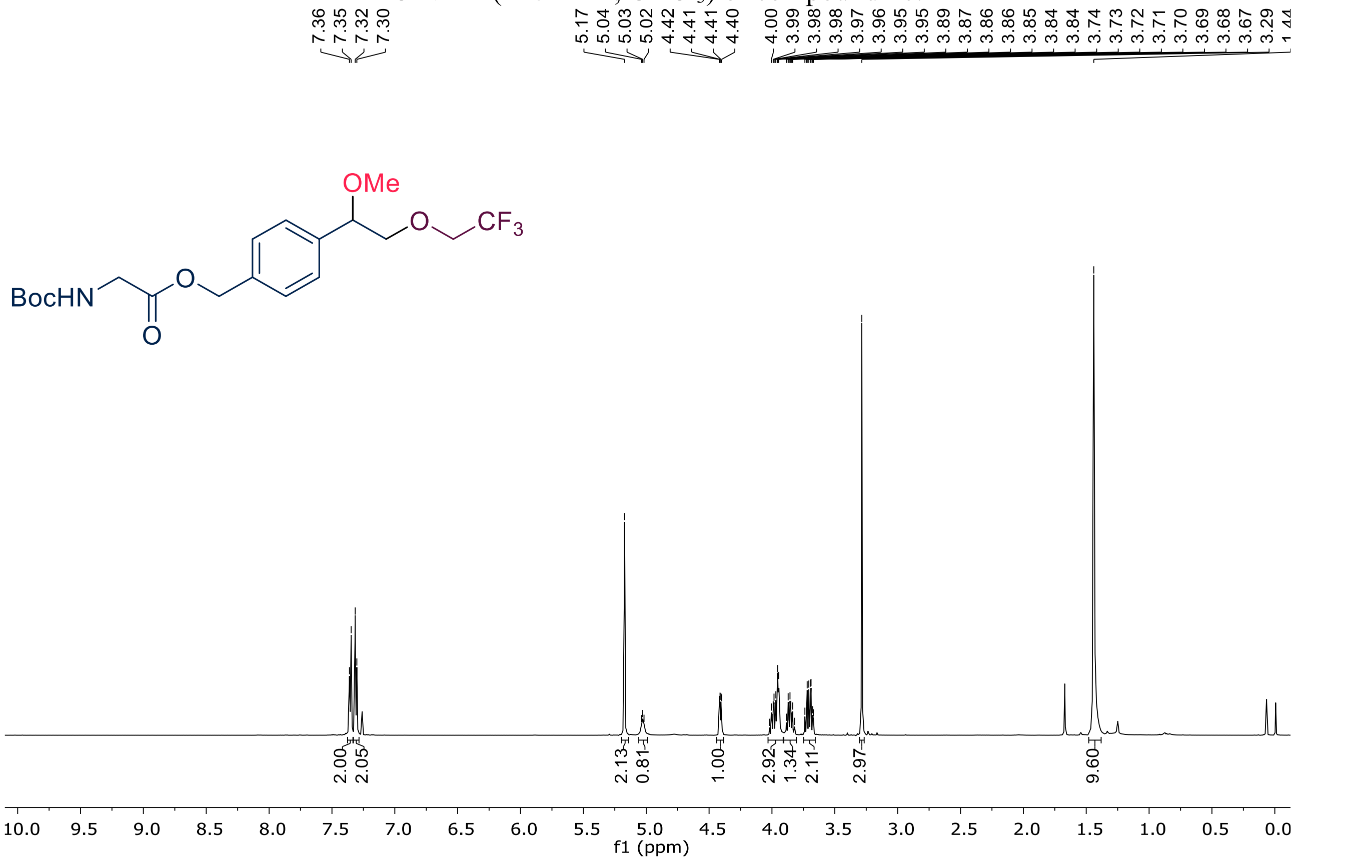

^{1}H NMR (600 MHz, CDCl$_3$) of compound **21**.

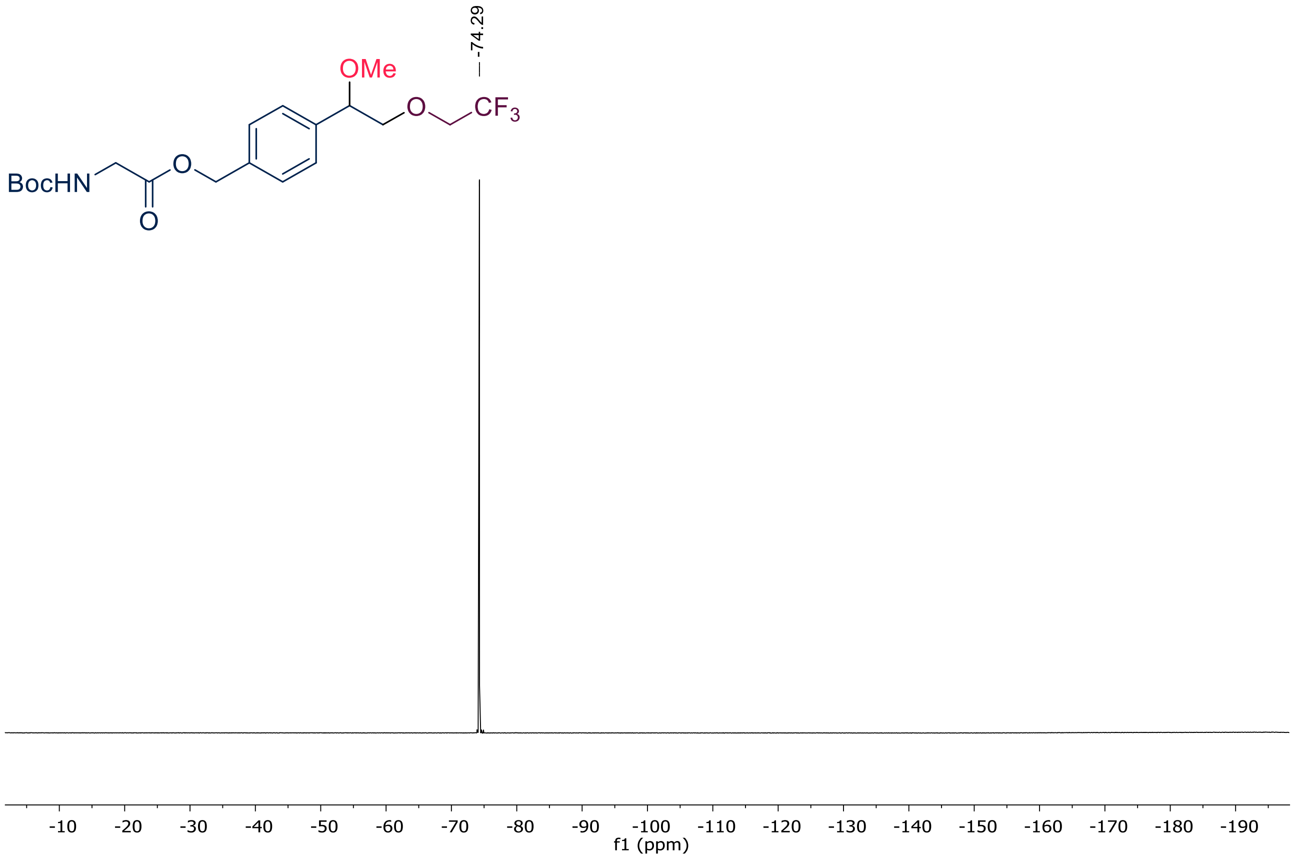

^{19}F NMR (282 MHz, $CDCl_3$) of compound **21**.

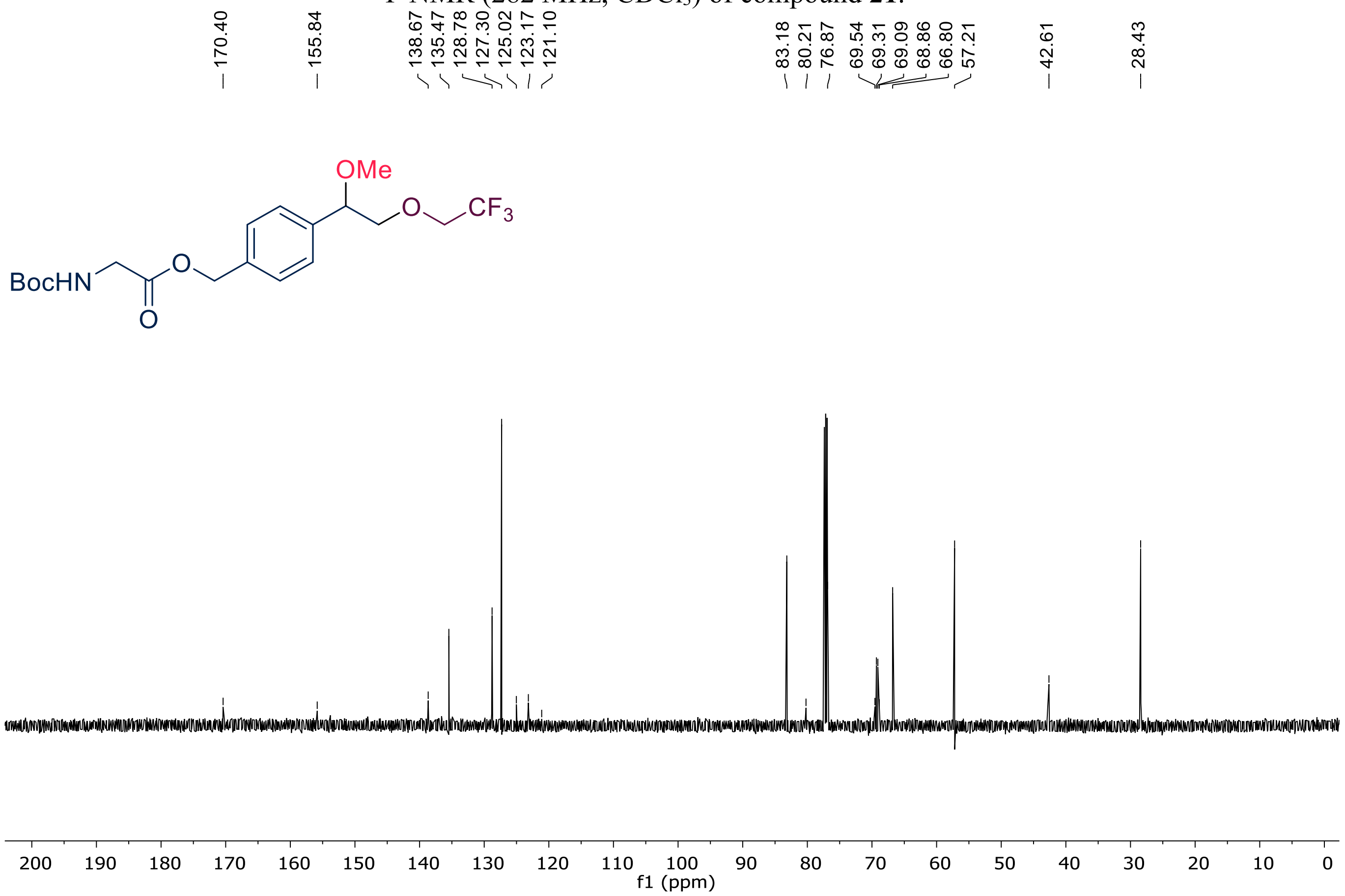

^{13}C NMR (151 MHz, $CDCl_3$) of compound **21**.

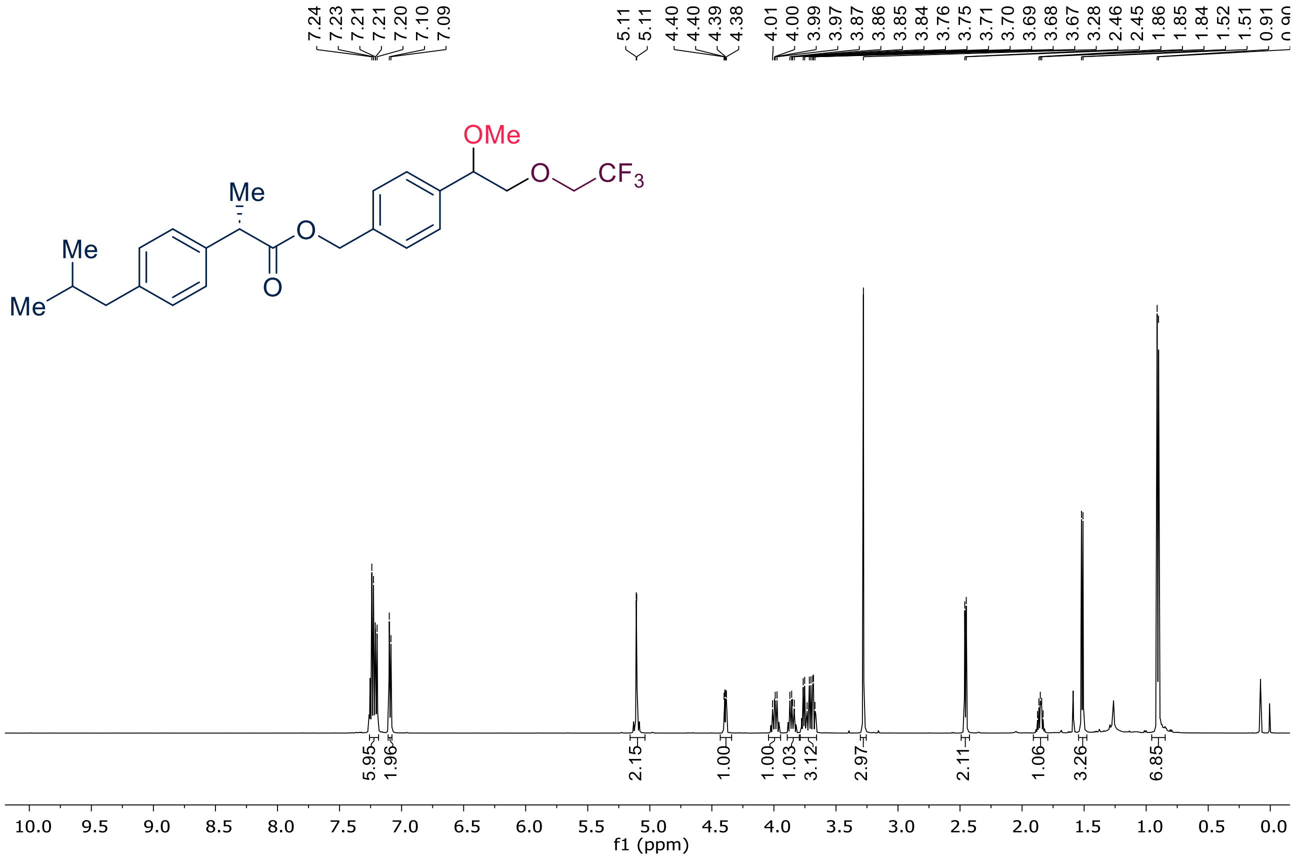

^{1}H NMR (600 MHz, $CDCl_3$) of compound **22**.

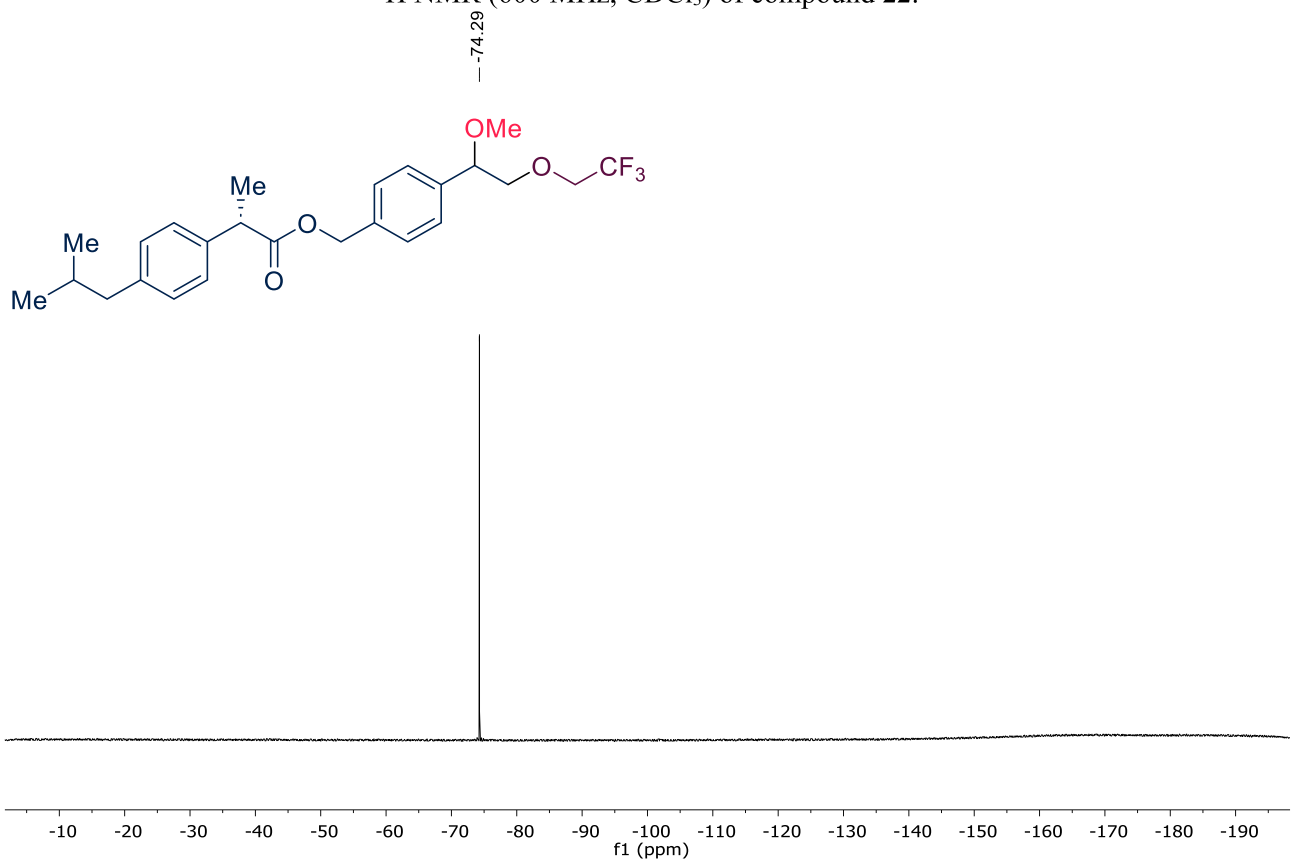

^{19}F NMR (282 MHz, $CDCl_3$) of compound **22**.

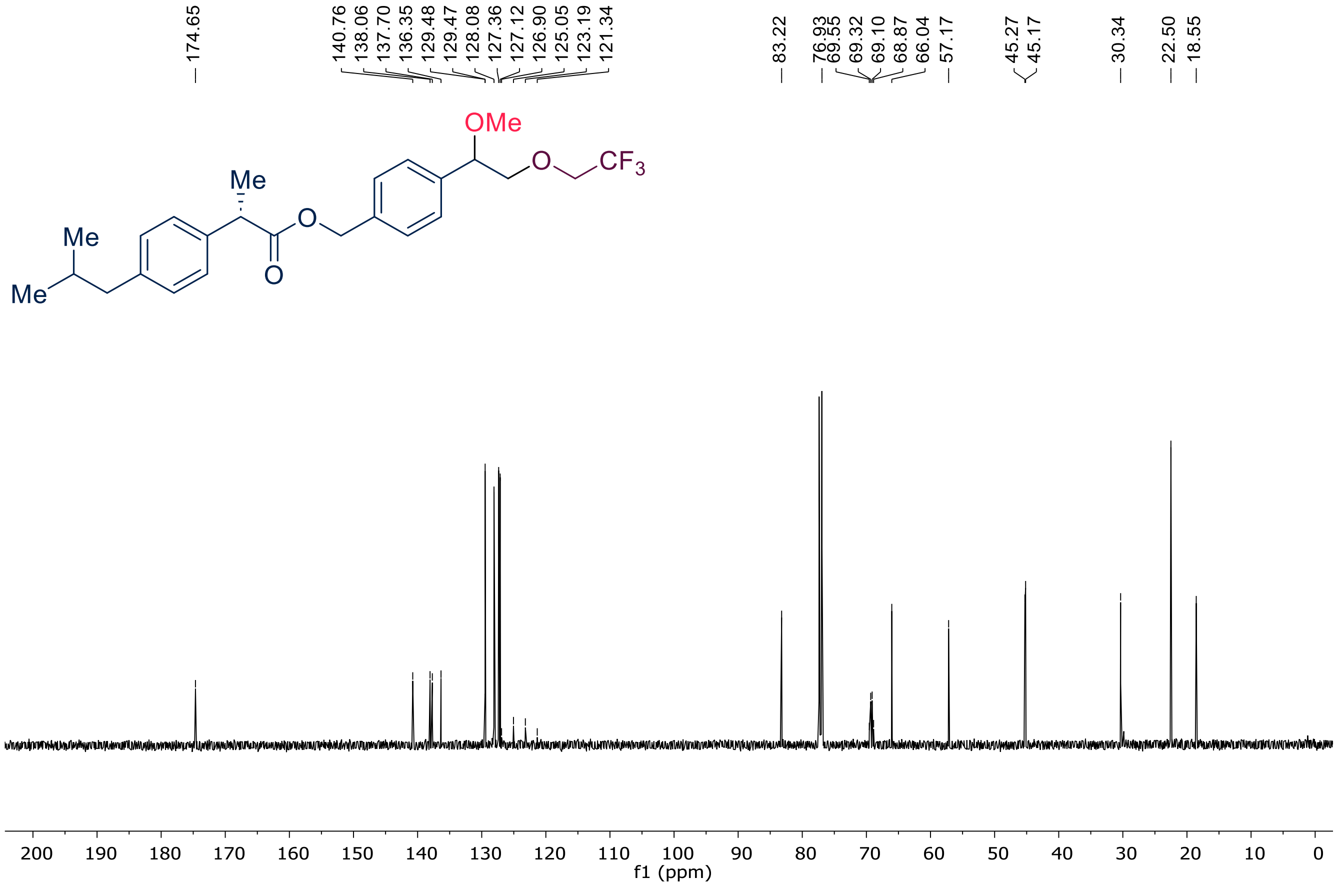

^{13}C NMR (101 MHz, $CDCl_3$) of compound **22**.

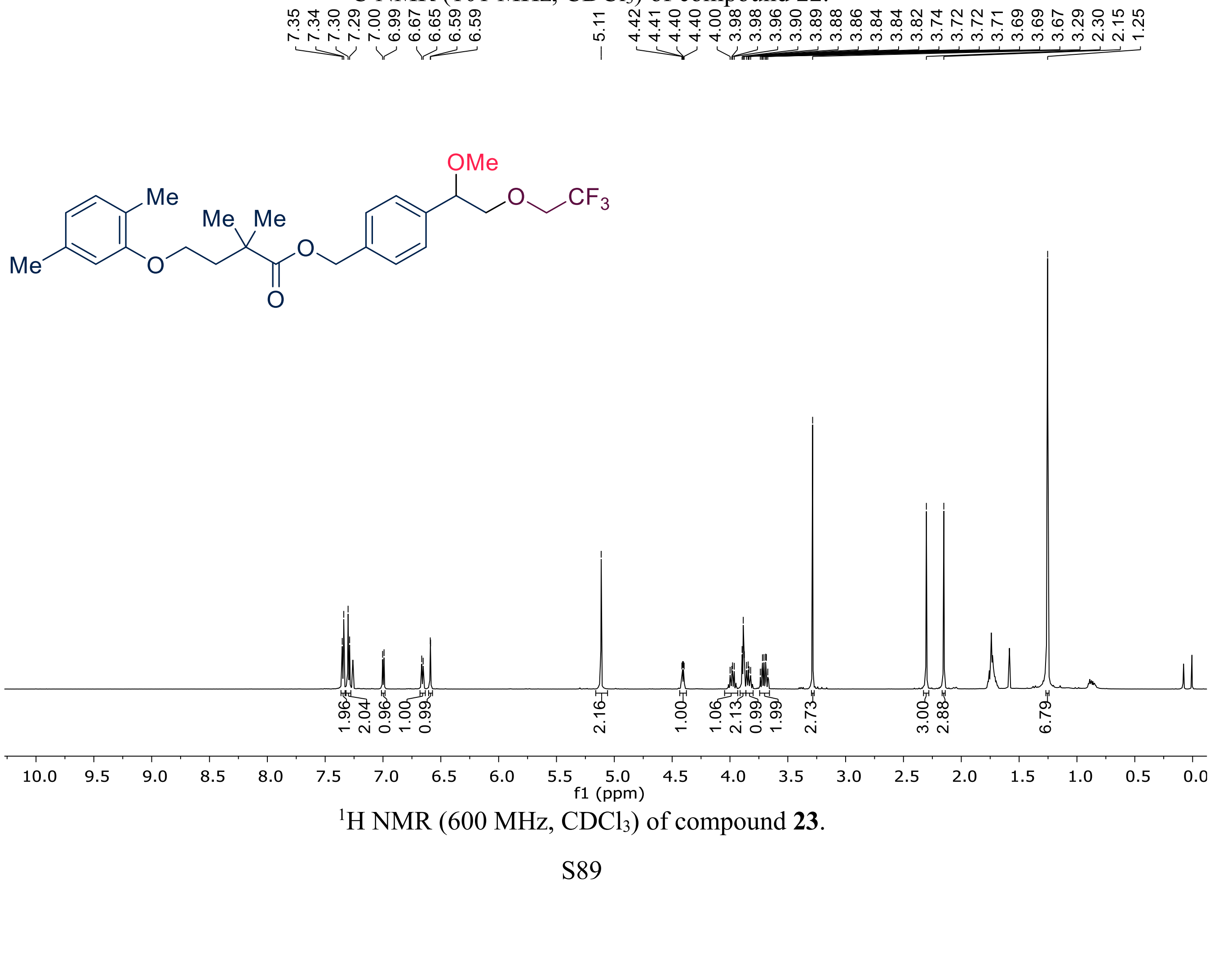

^{1}H NMR (600 MHz, $CDCl_3$) of compound **23**.

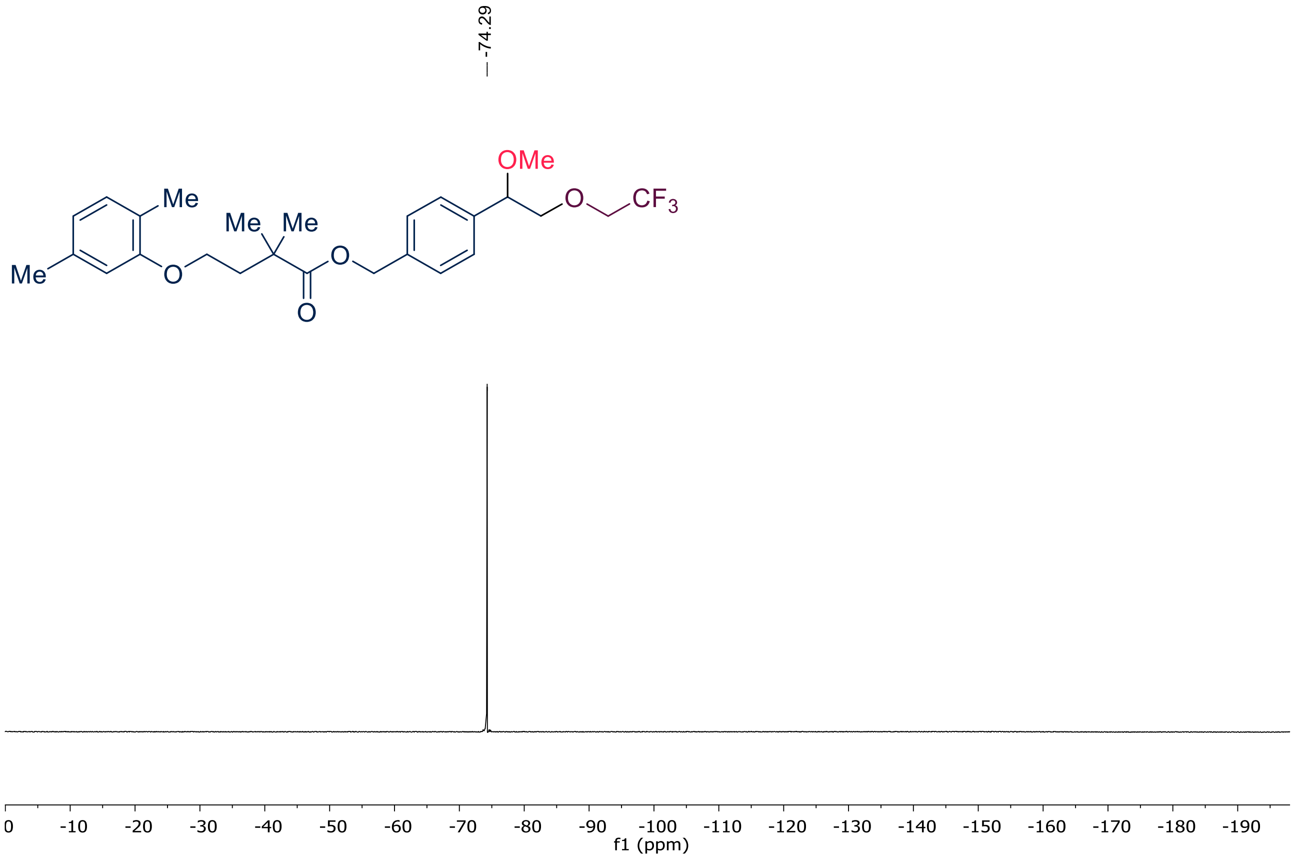

^{19}F NMR (377 MHz, CDCl$_3$) of compound **23**.

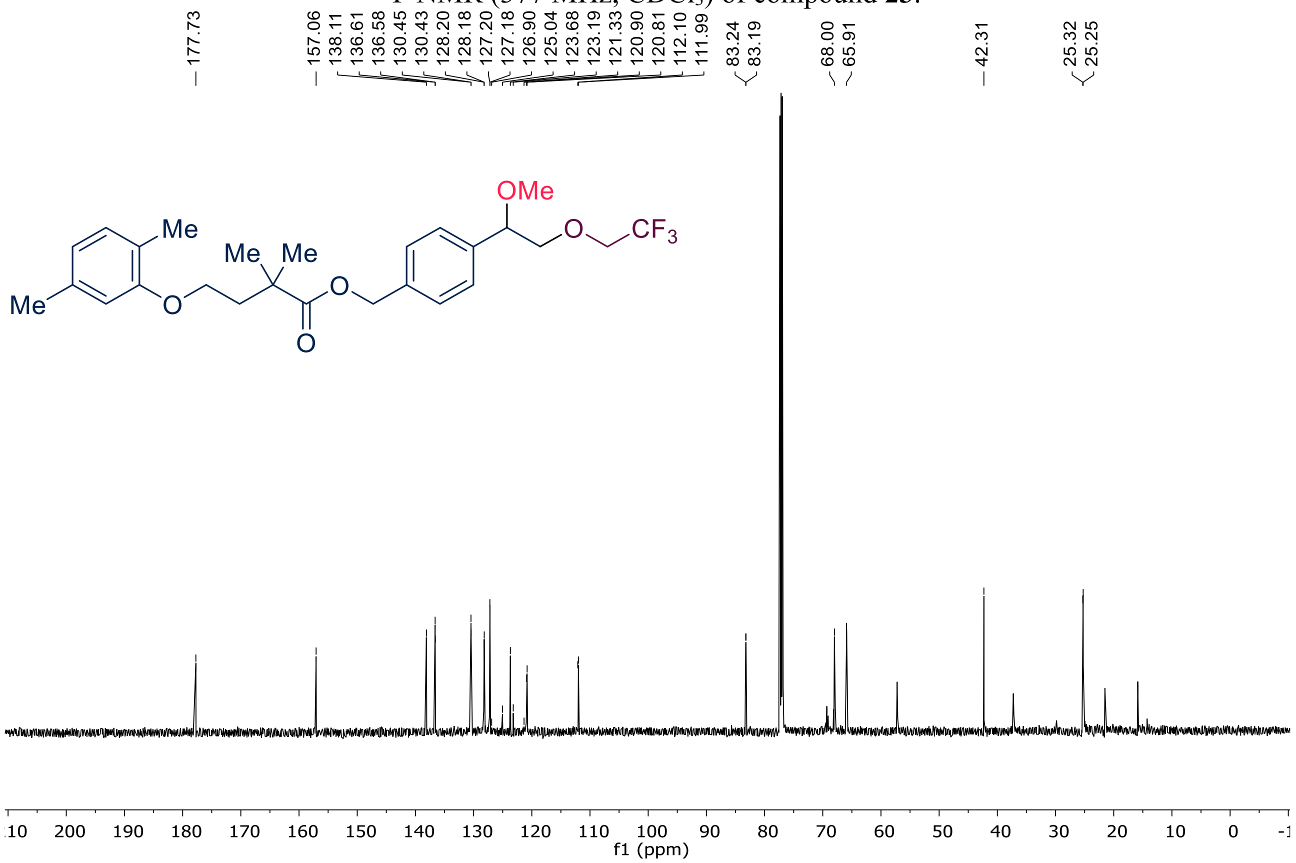

^{13}C NMR (126 MHz, CDCl$_3$) of compound **23**.

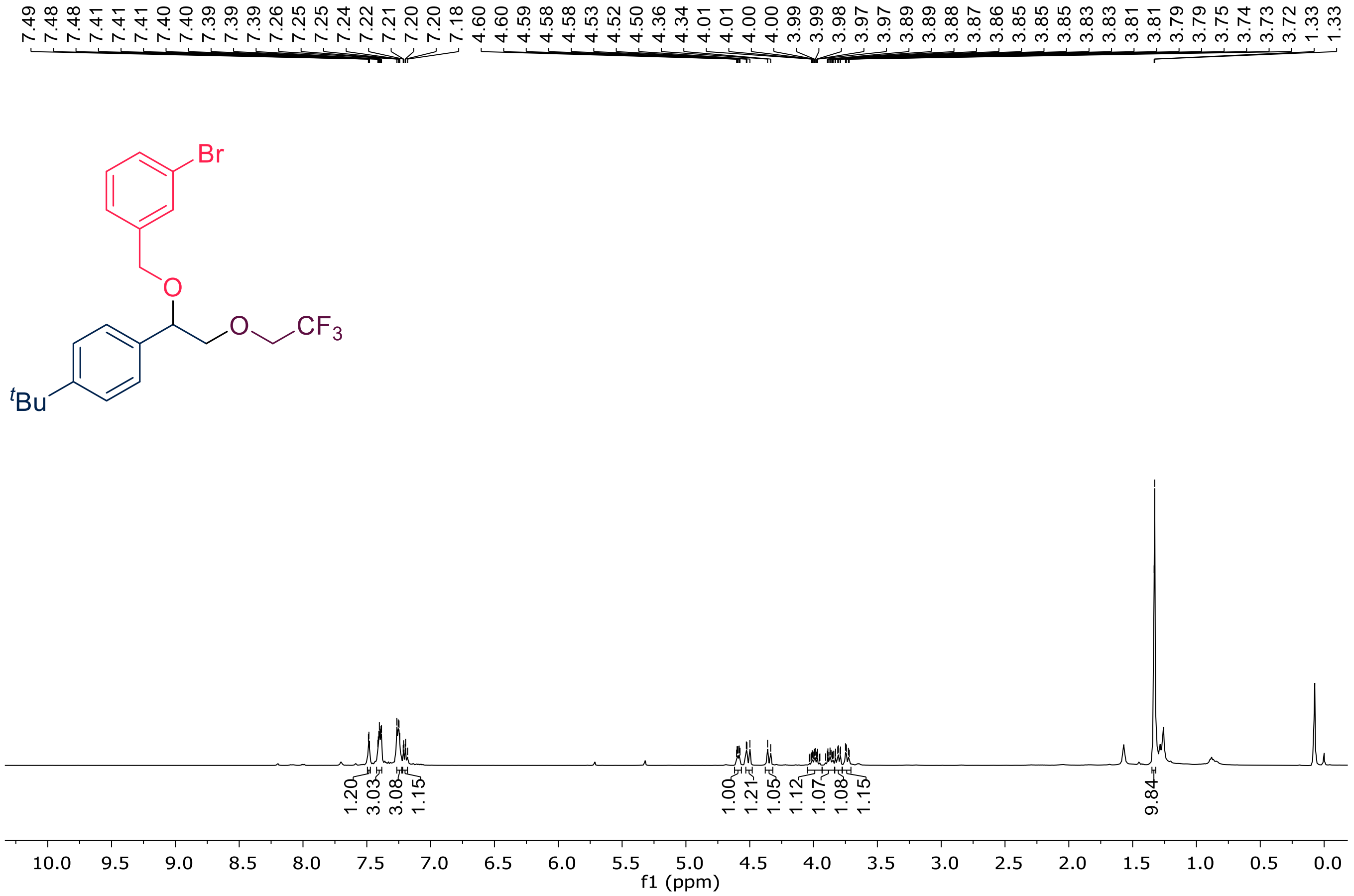

^{1}H NMR (500 MHz, $CDCl_3$) of compound **24**.

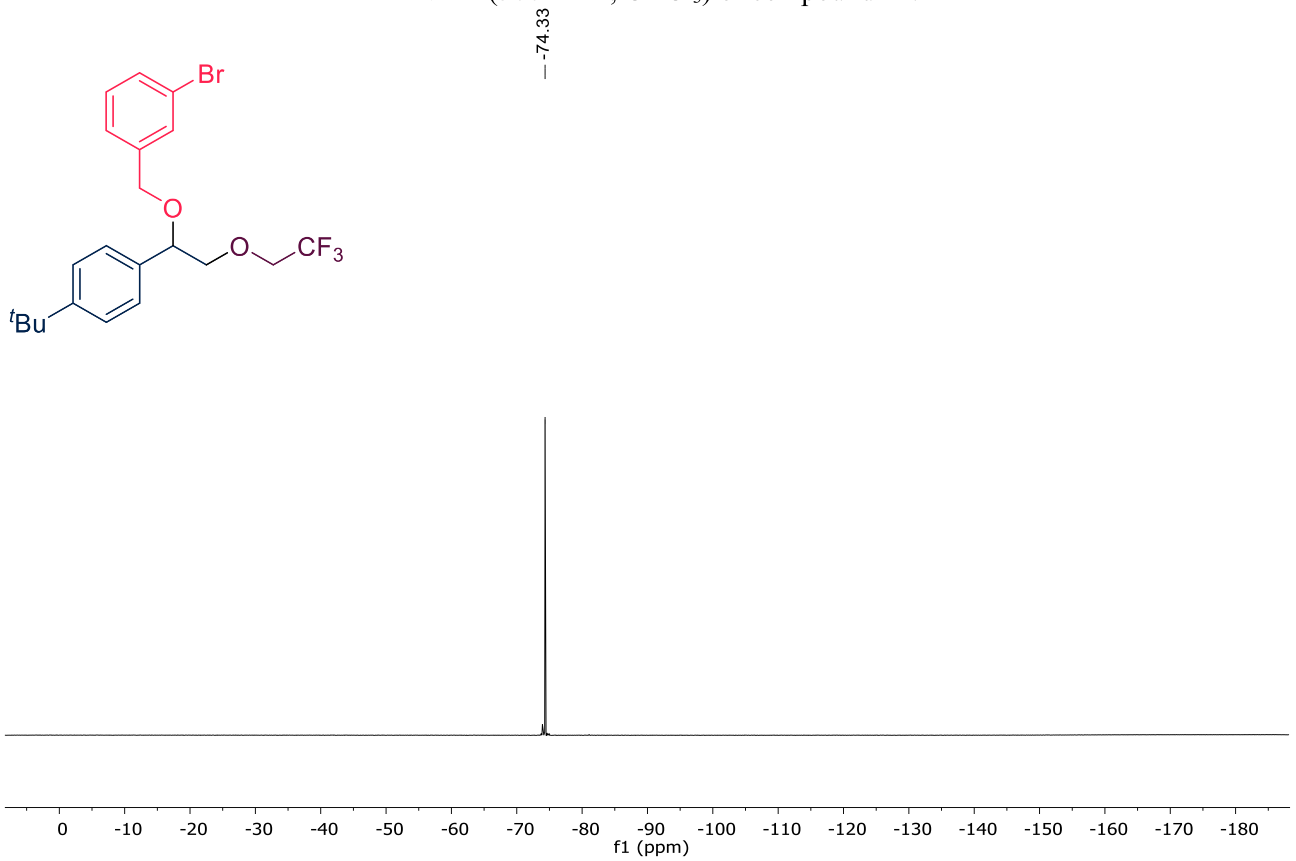

^{19}F NMR (377 MHz, $CDCl_3$) of compound **24**.

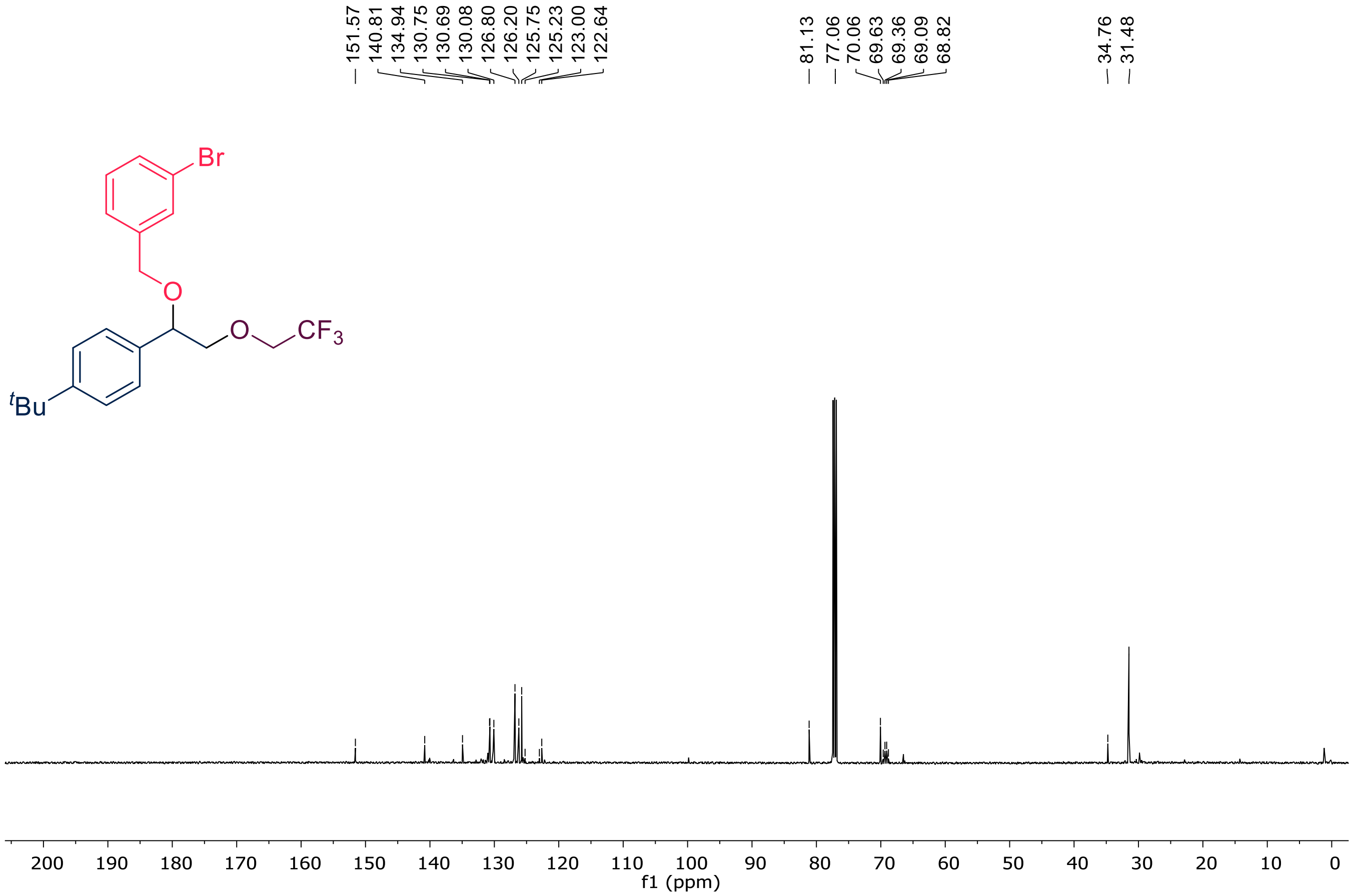

^{13}C NMR (126 MHz, CDCl$_3$) of compound **24**.

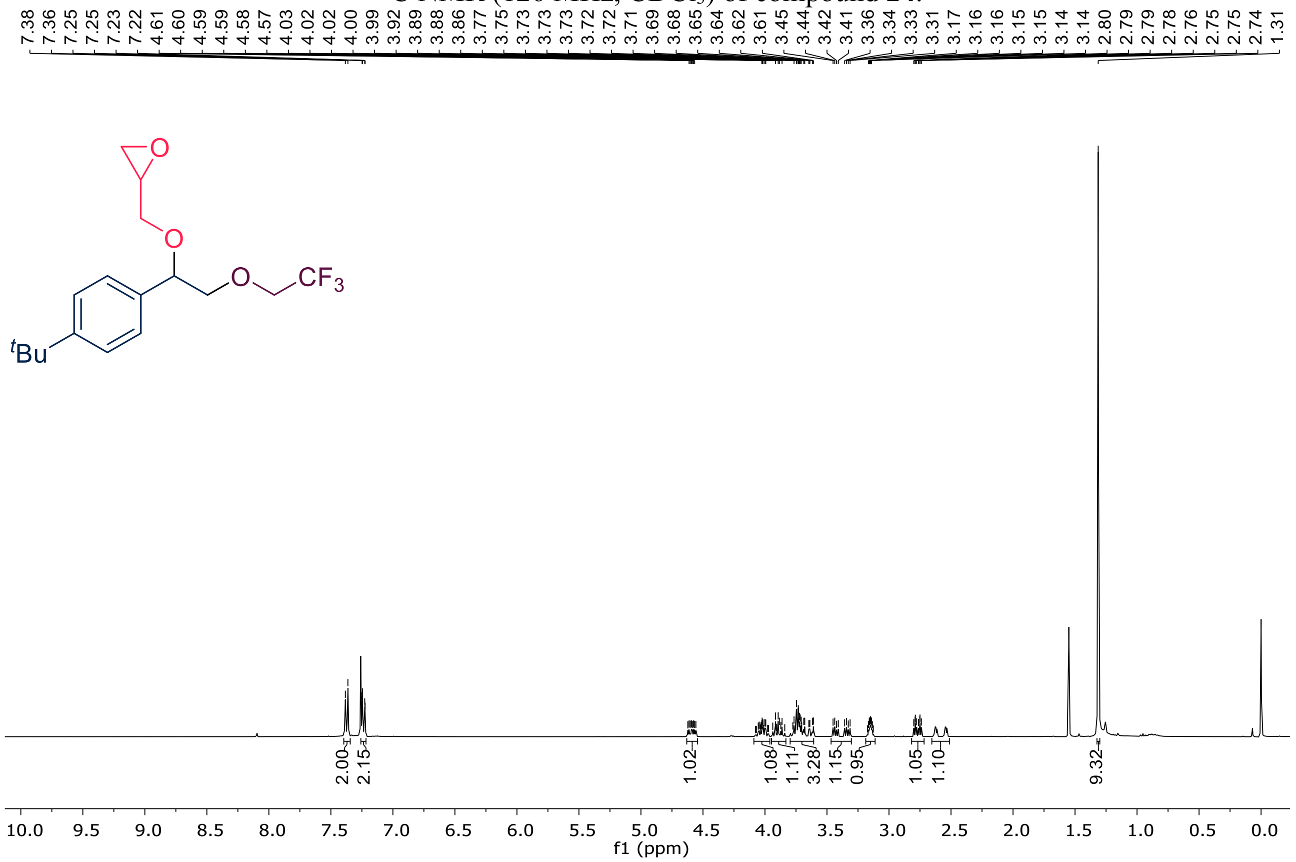

^{1}H NMR (400 MHz, CDCl$_3$) of compound **25**.

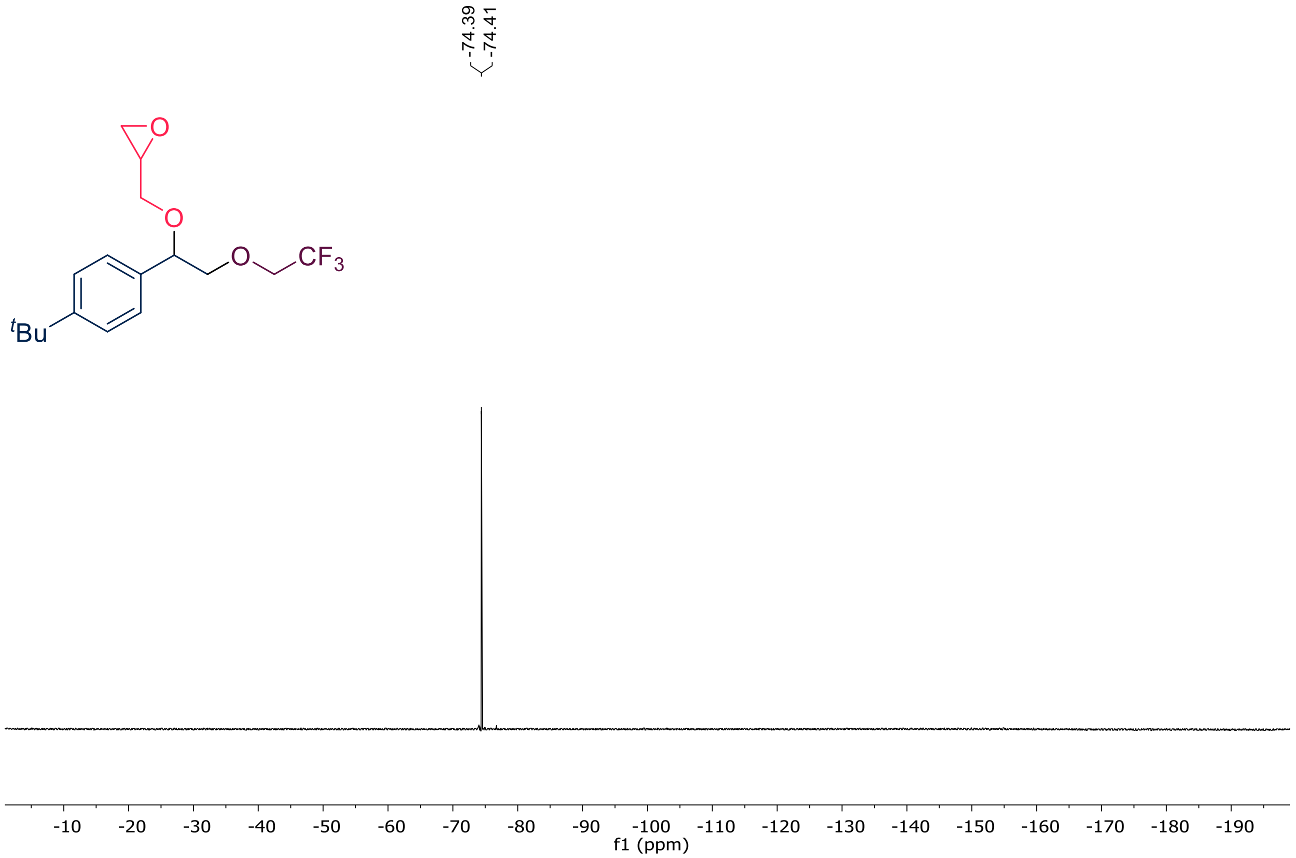

^{19}F NMR (377 MHz, $CDCl_3$) of compound **25**.

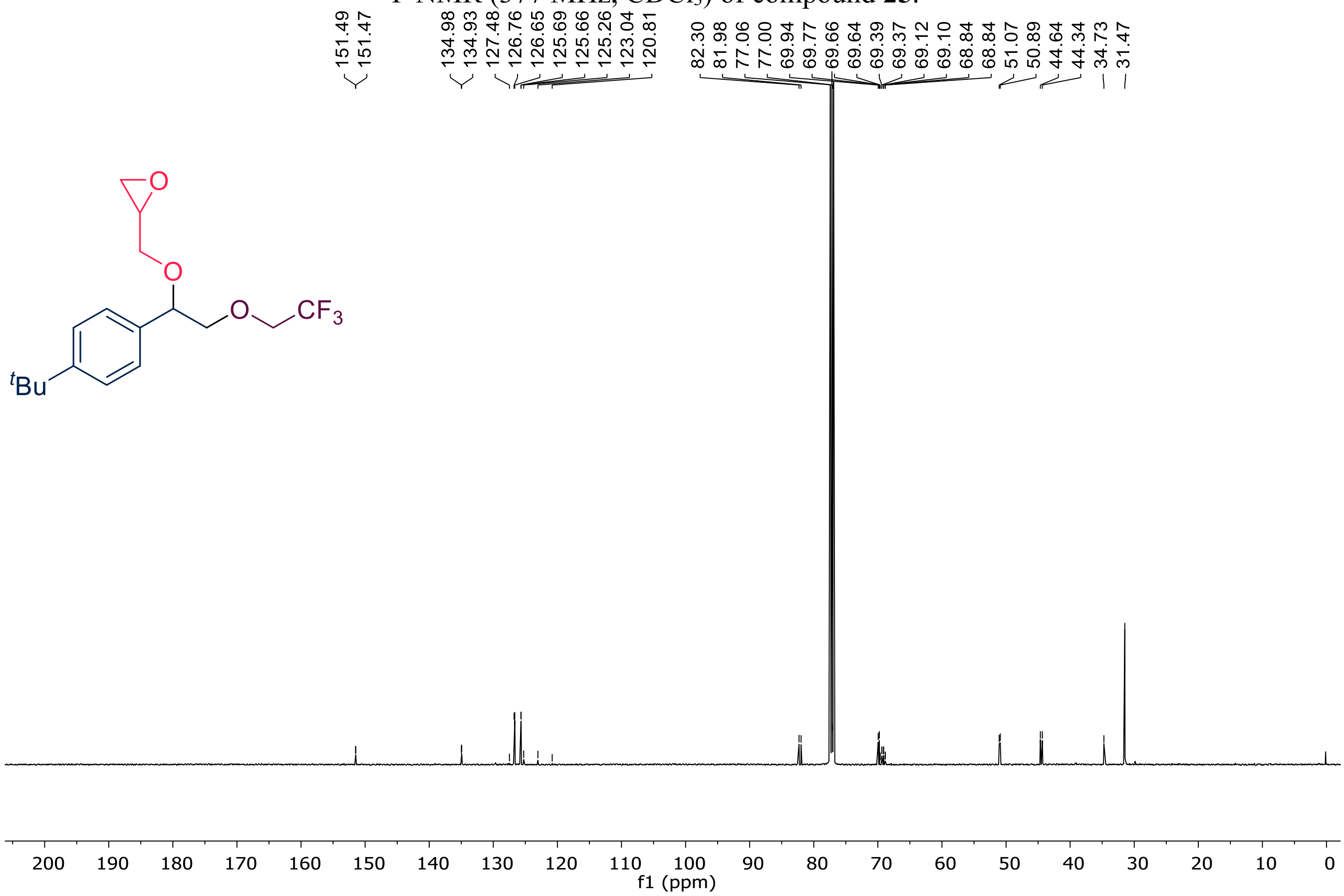

^{13}C NMR (126 MHz, $CDCl_3$) of compound **25**.

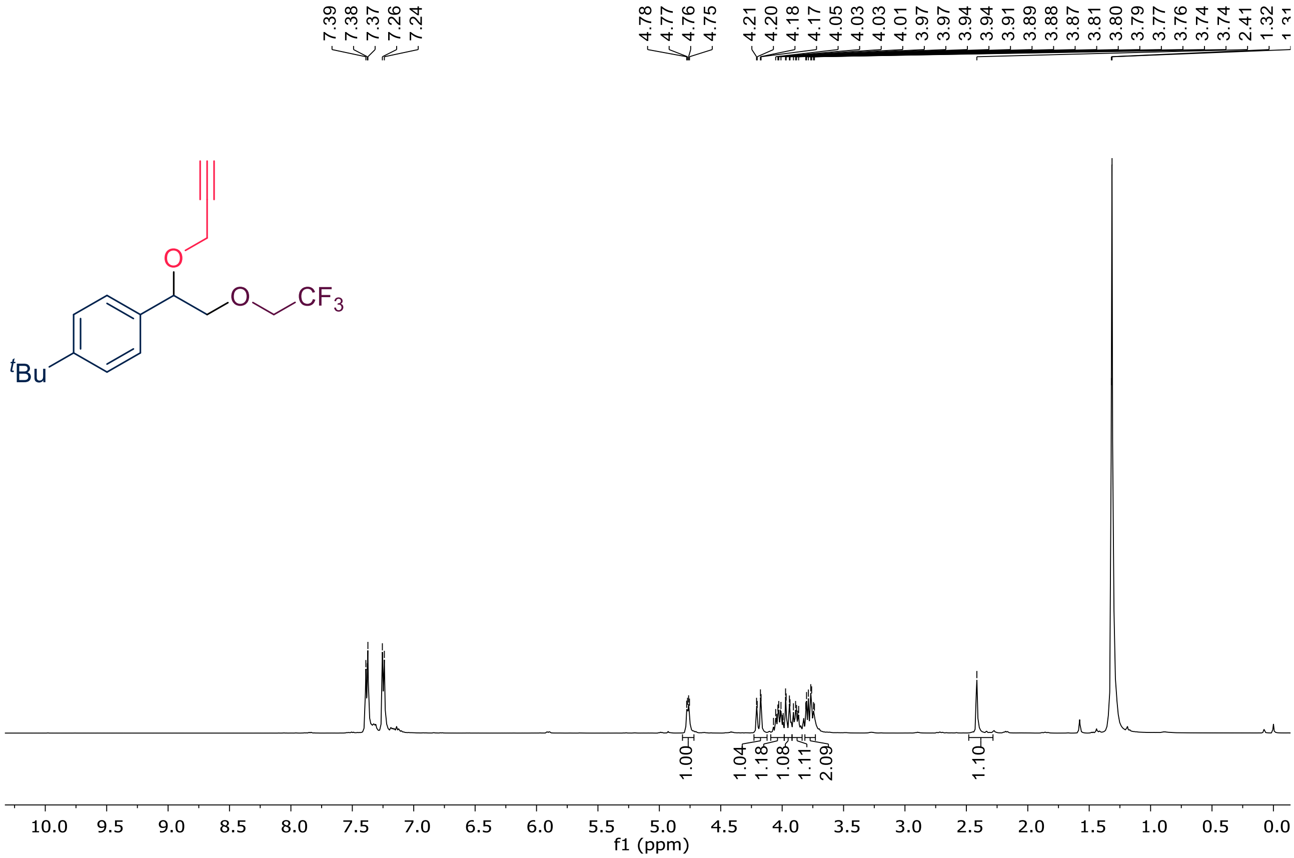

^{1}H NMR (500 MHz, CDCl3) of compound **26**.

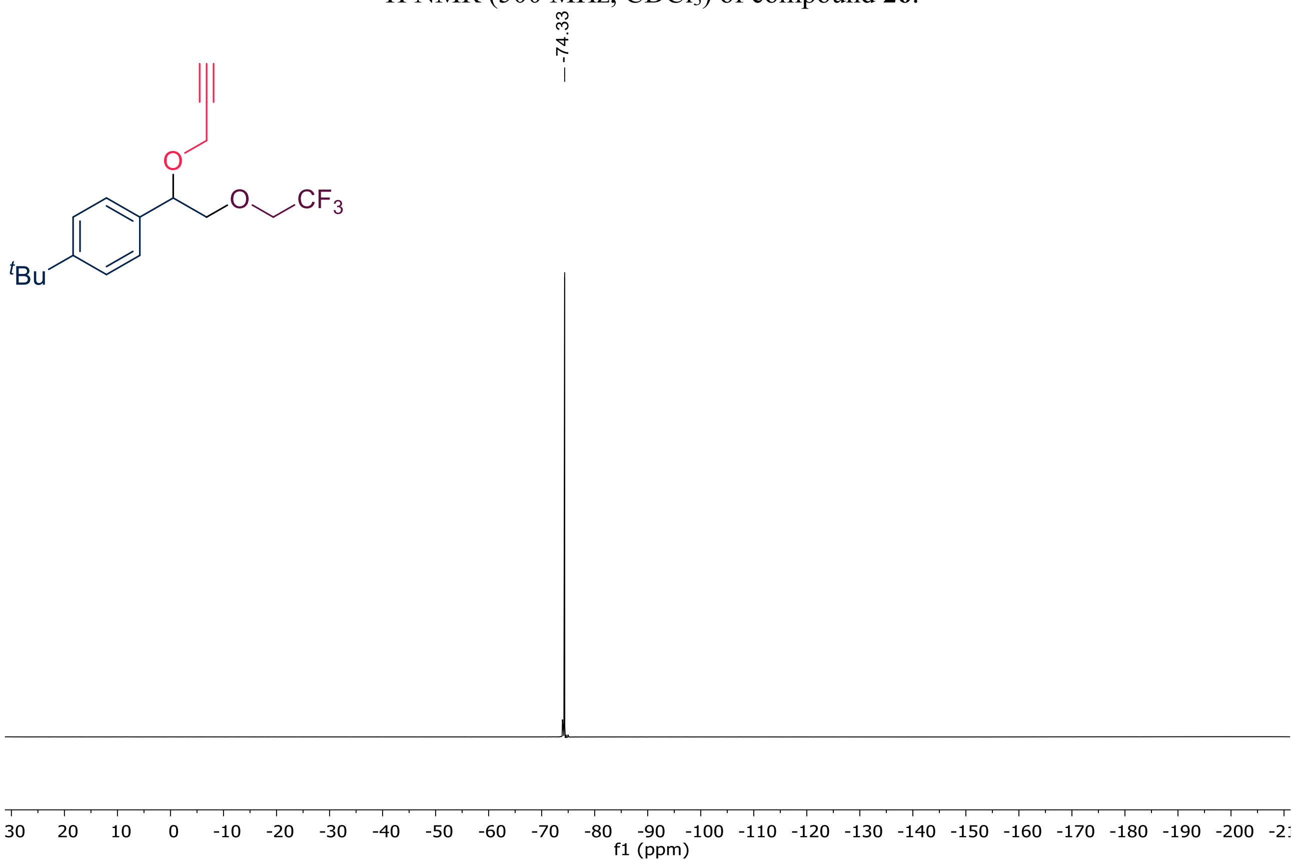

^{19}F NMR (282 MHz, CDCl3) of compound **26**.

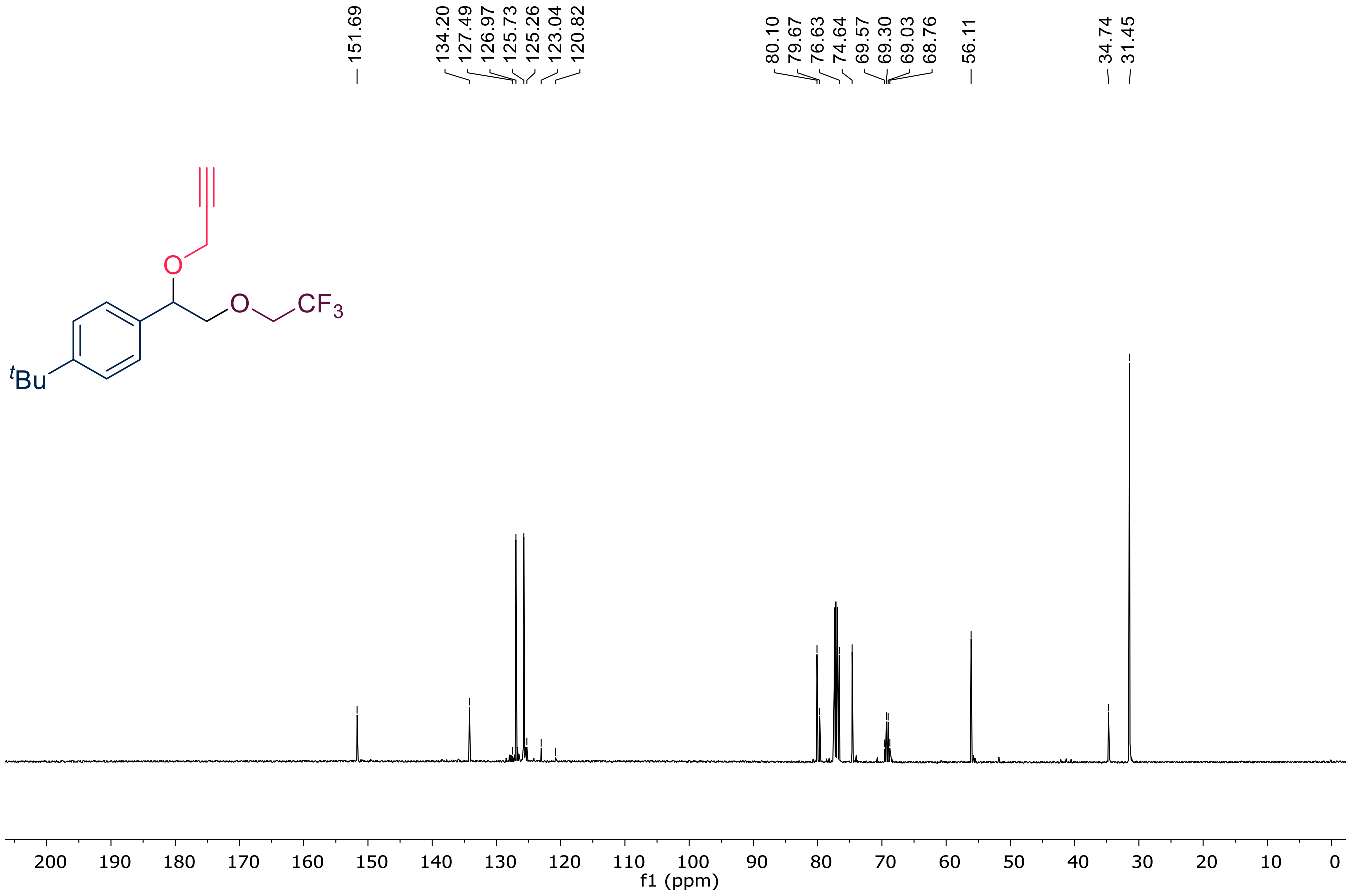

^{13}C NMR (101 MHz, $CDCl_3$) of compound **26**.

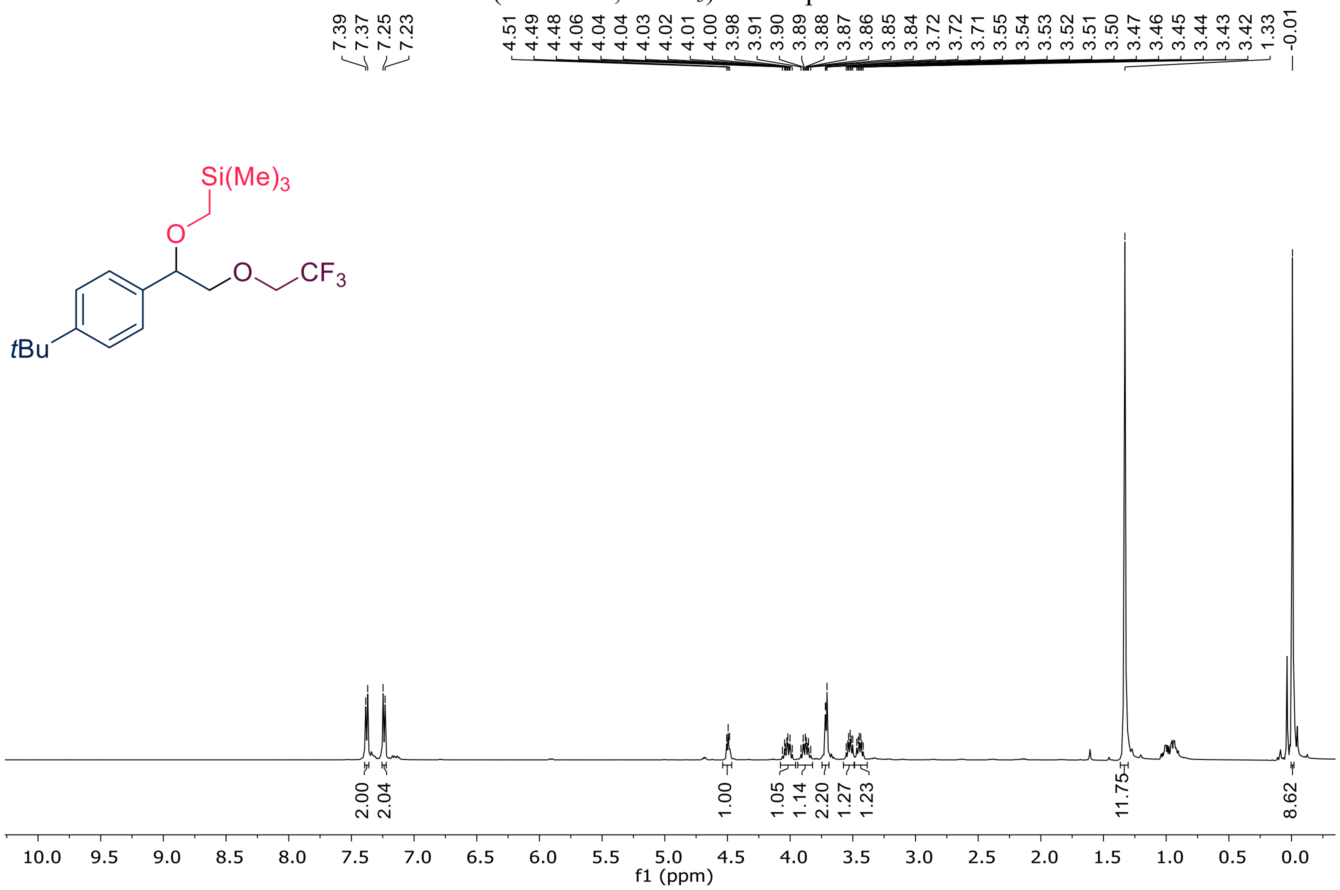

^{1}H NMR (500 MHz, $CDCl_3$) of compound **27**.

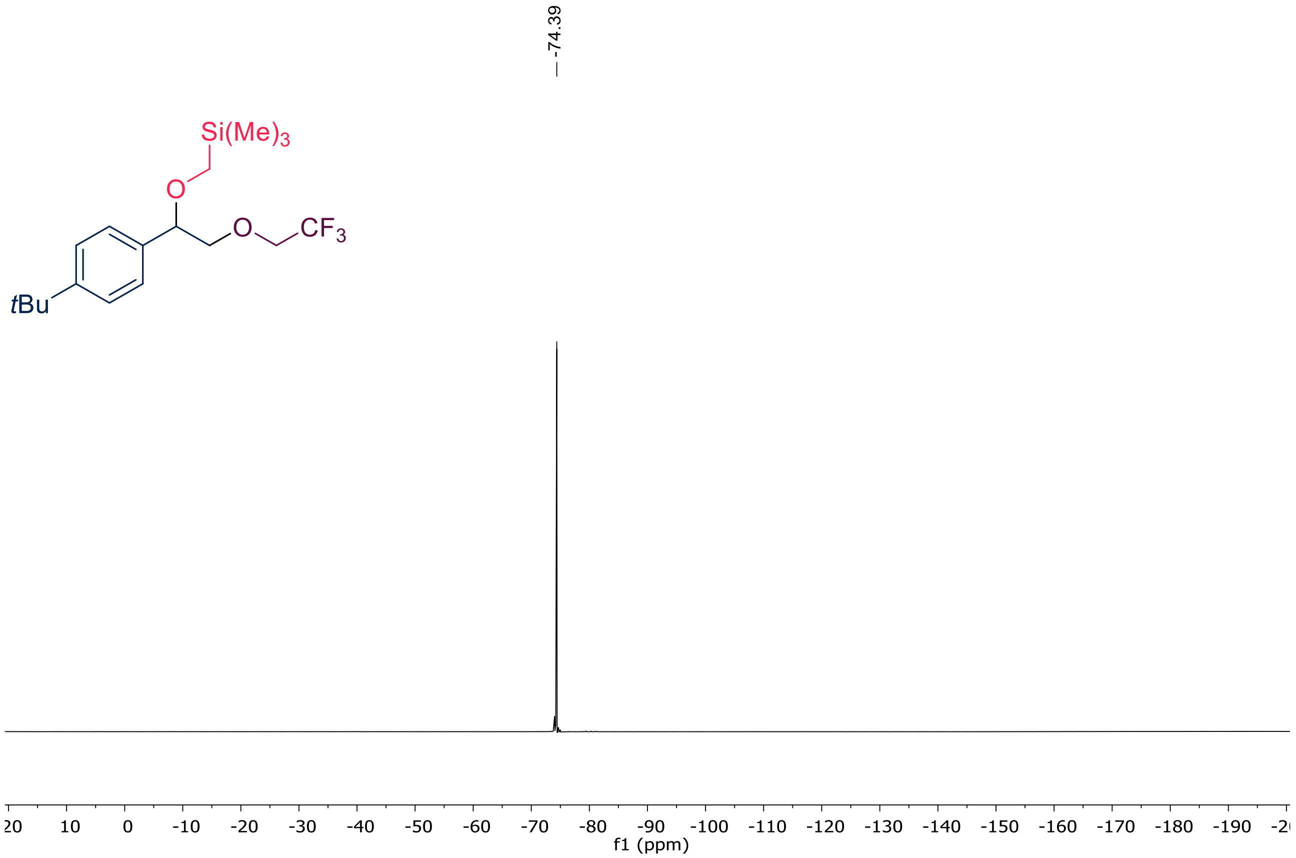

^{19}F NMR (282 MHz, CDCl$_3$) of compound **27**.

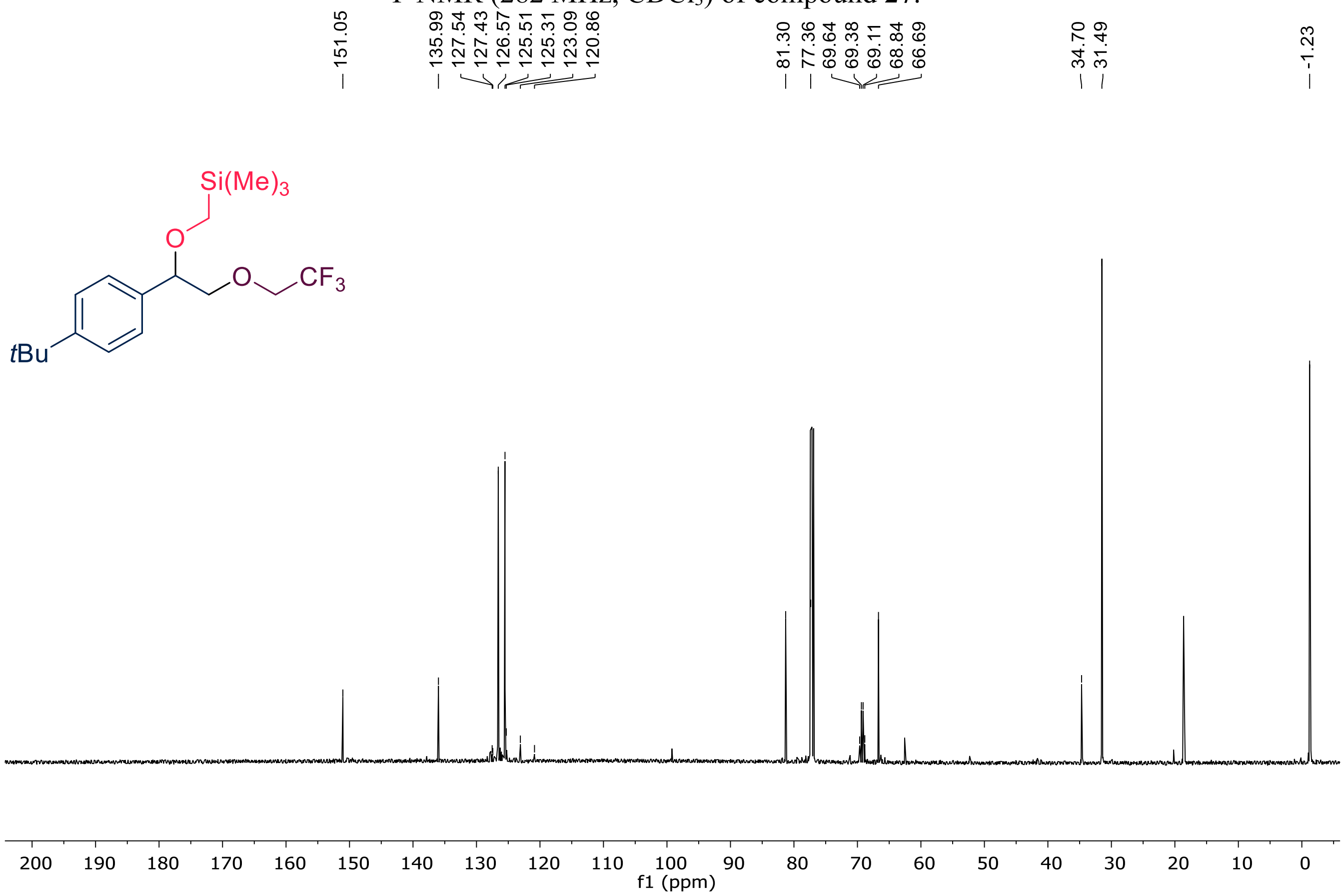

^{13}C NMR (126 MHz, CDCl$_3$) of compound **27**.

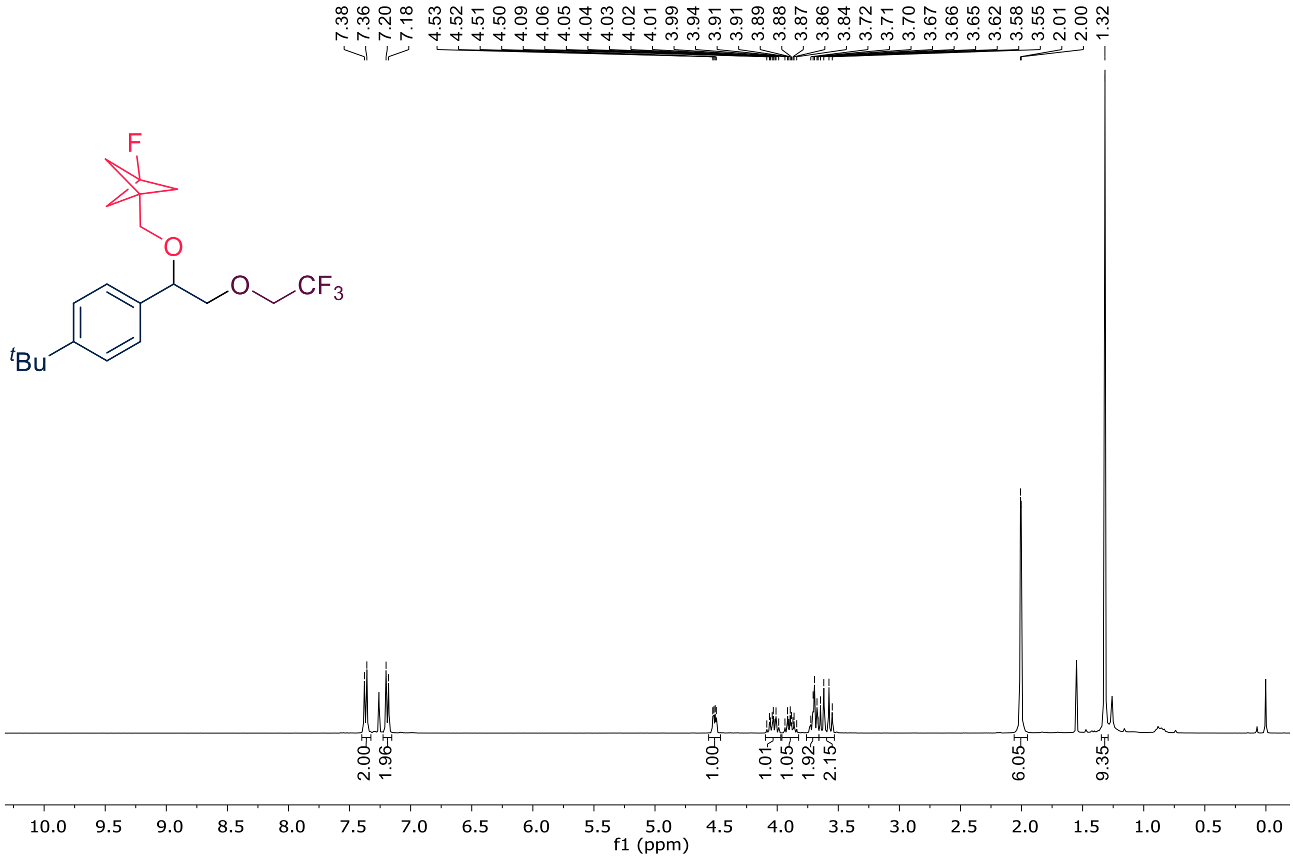

^{1}H NMR (500 MHz, $CDCl_3$) of compound **28**.

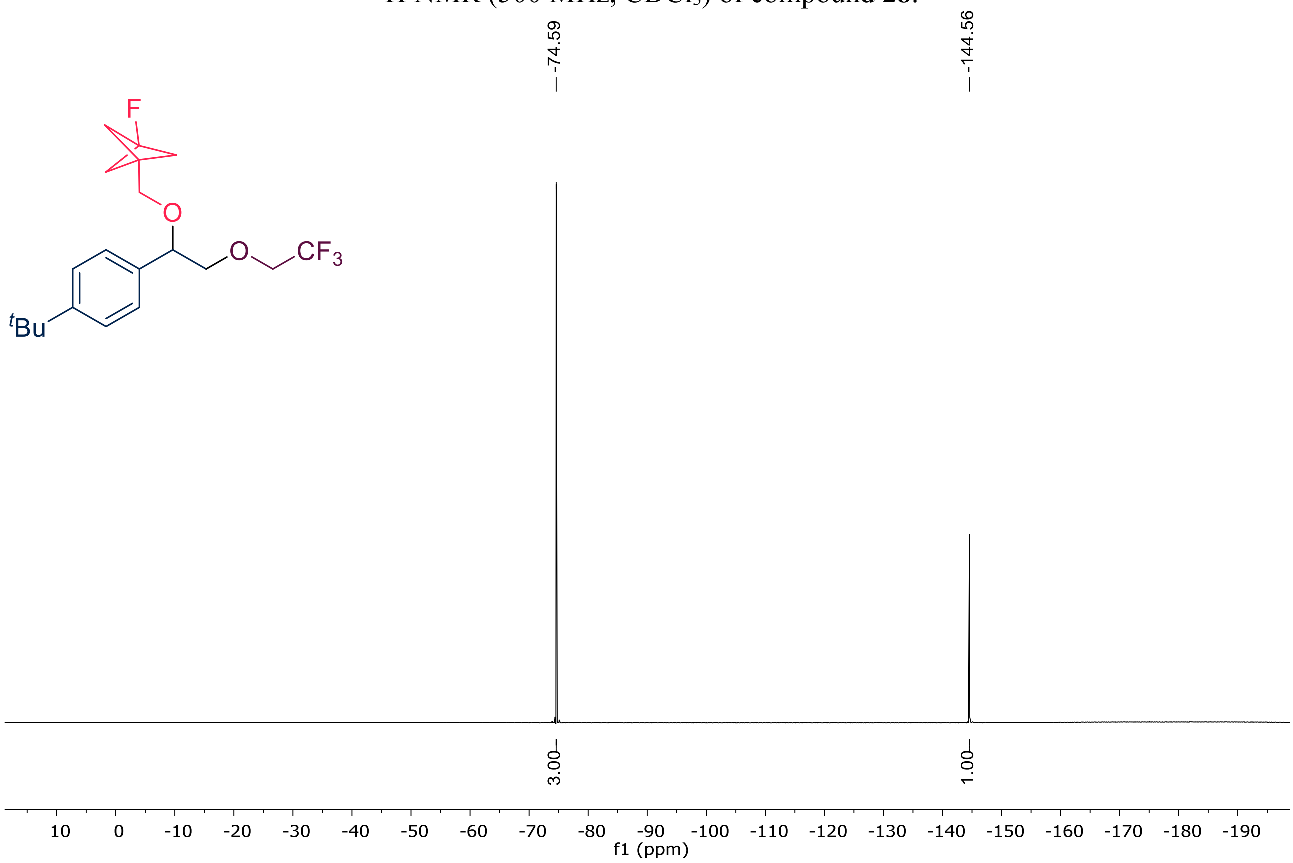

^{19}F NMR (377 MHz, $CDCl_3$) of compound **28**.

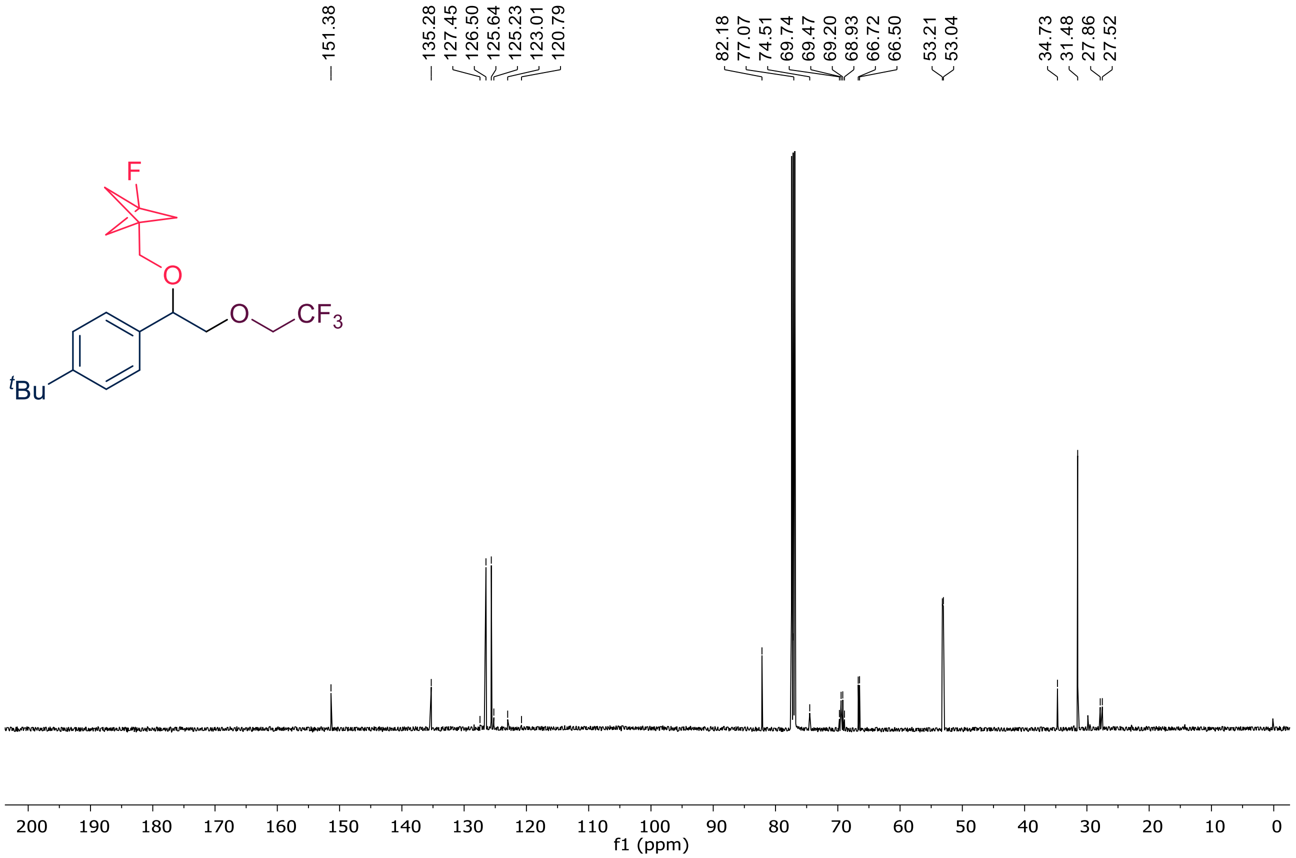

^{13}C NMR (126 MHz, $CDCl_3$) of compound **28**.

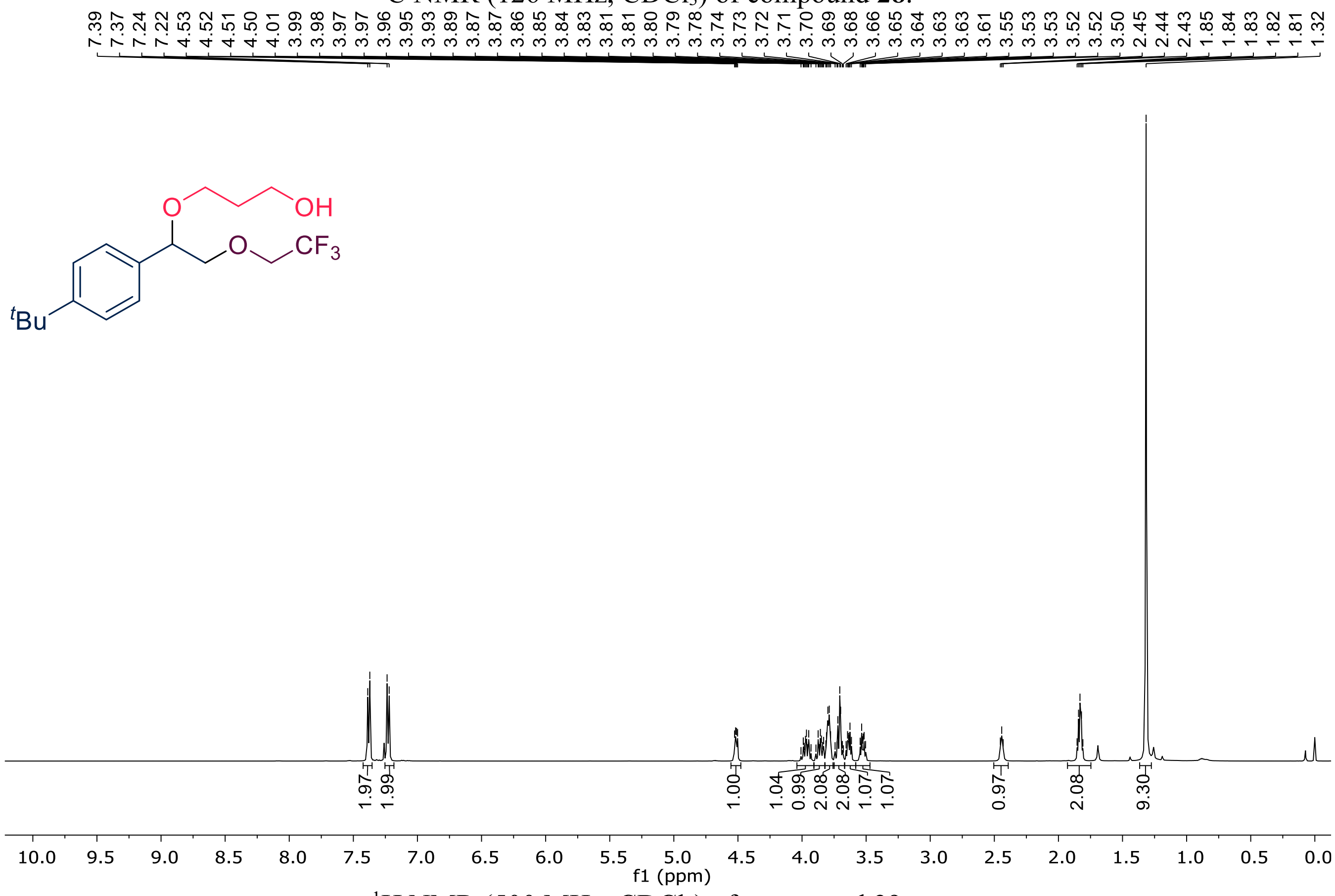

^{1}H NMR (500 MHz, $CDCl_3$) of compound **29**.

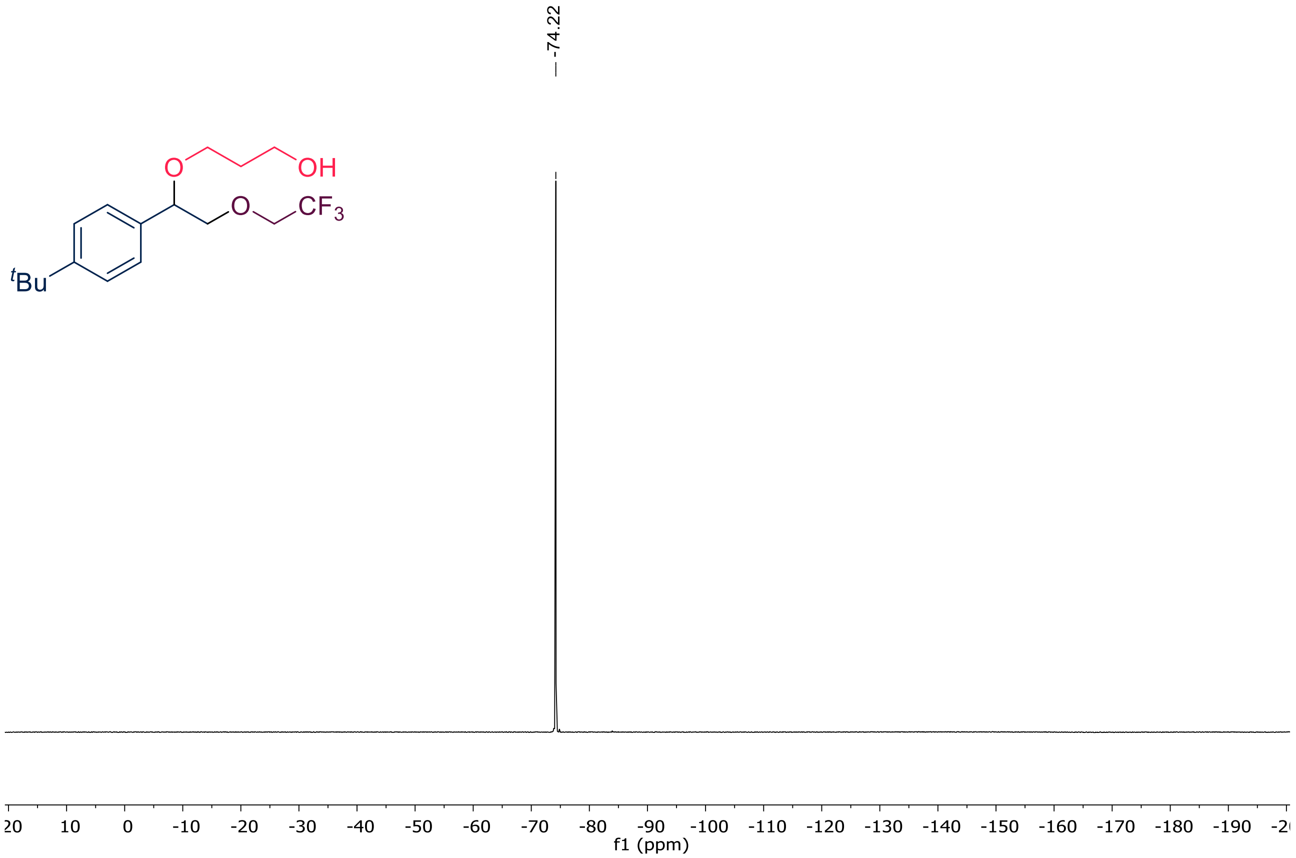

^{19}F NMR (282 MHz, $CDCl_3$) of compound **29**.

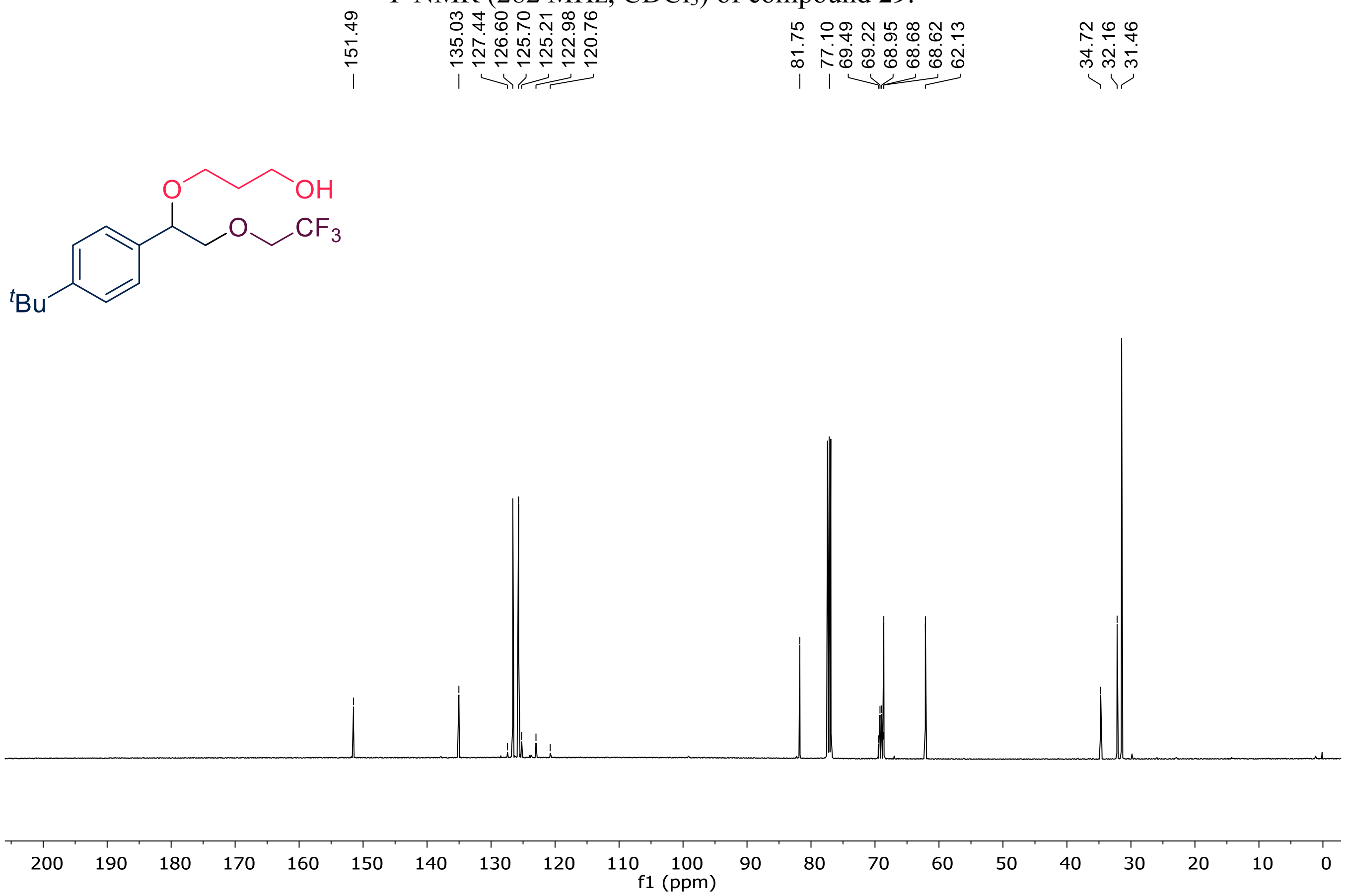

^{13}C NMR (126 MHz, $CDCl_3$) of compound **29**.

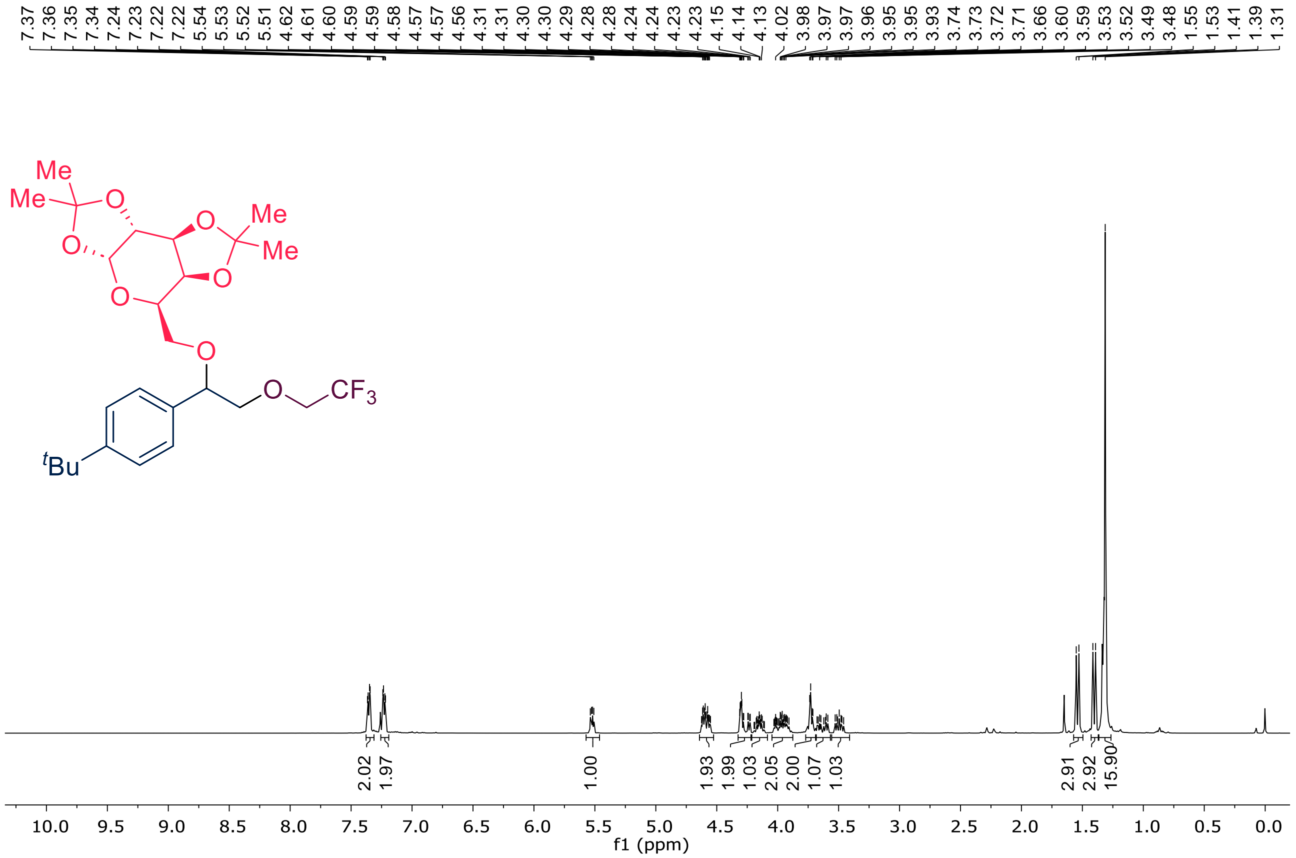

^{1}H NMR (500 MHz, $CDCl_3$) of compound **30**.

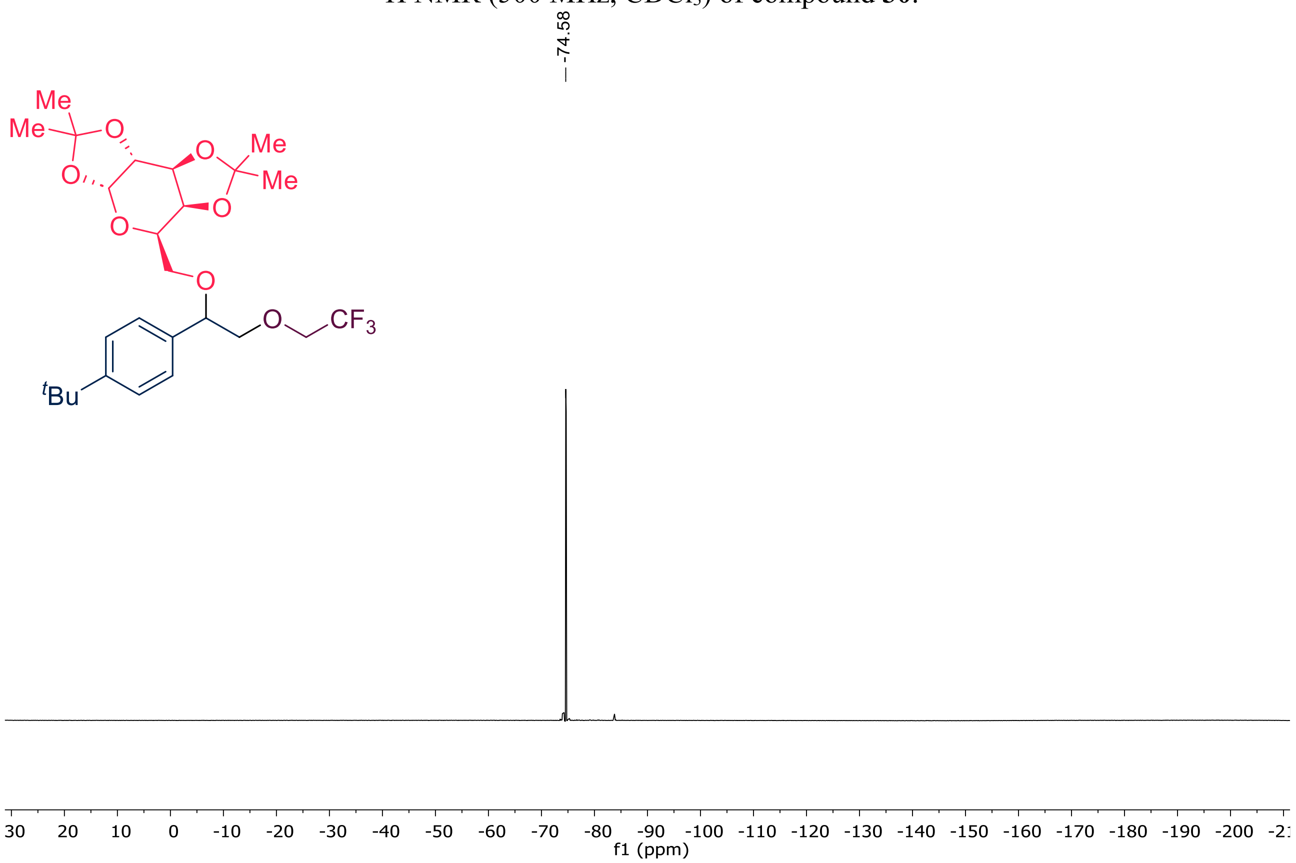

^{19}F NMR (282 MHz, $CDCl_3$) of compound **30**.

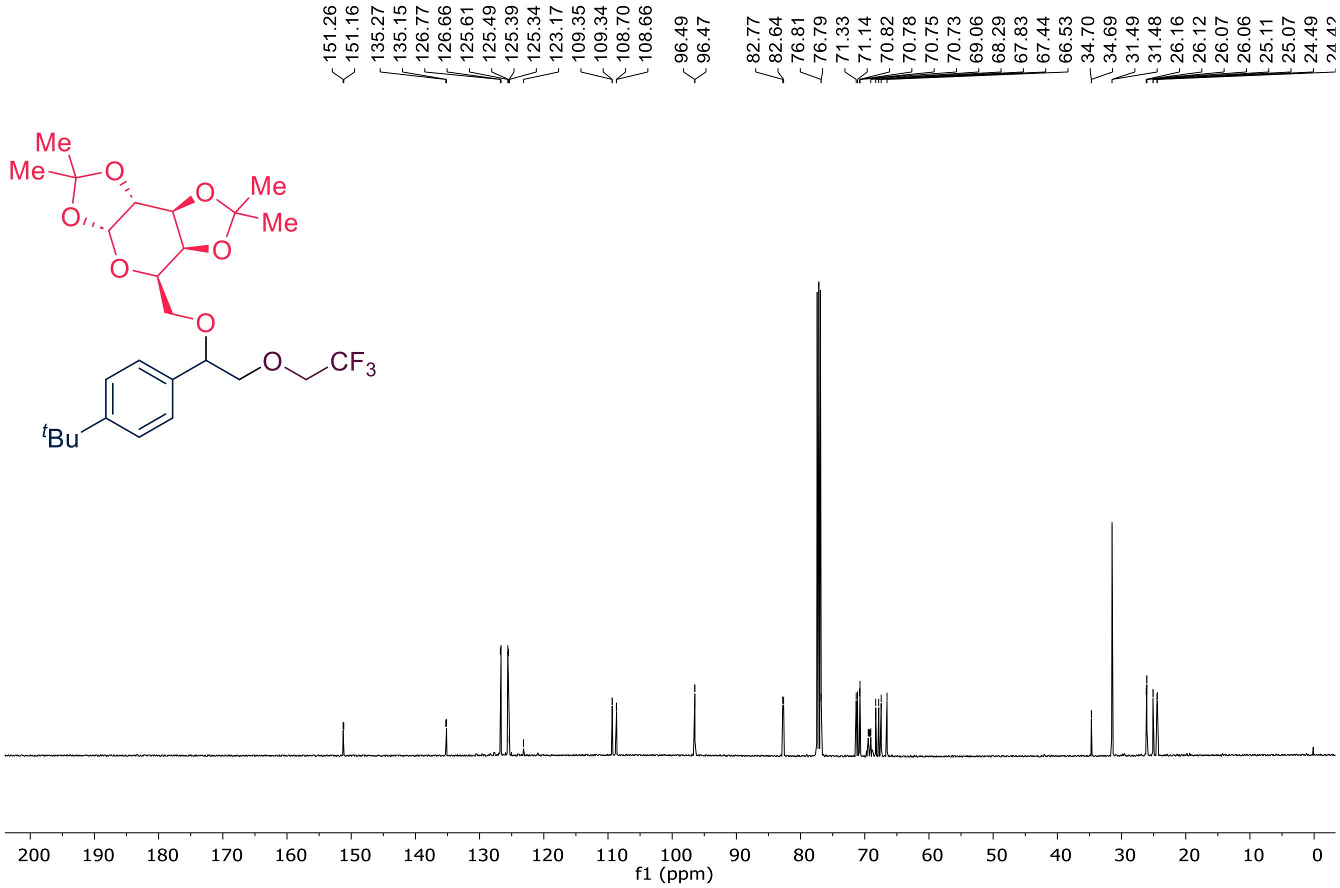

^{13}C NMR (101 MHz, $CDCl_3$) of compound **30**.

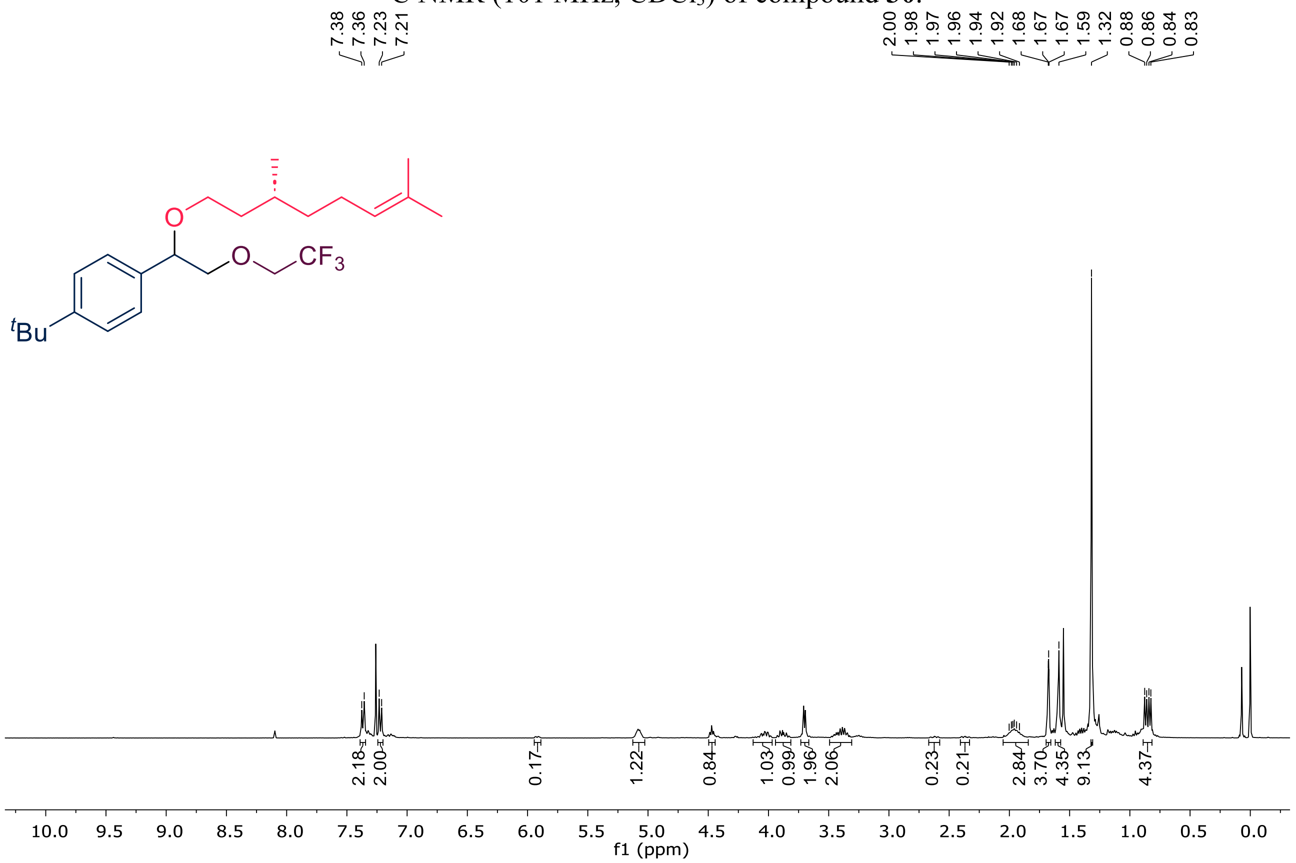

^{1}H NMR (400 MHz, $CDCl_3$) of compound **31**.

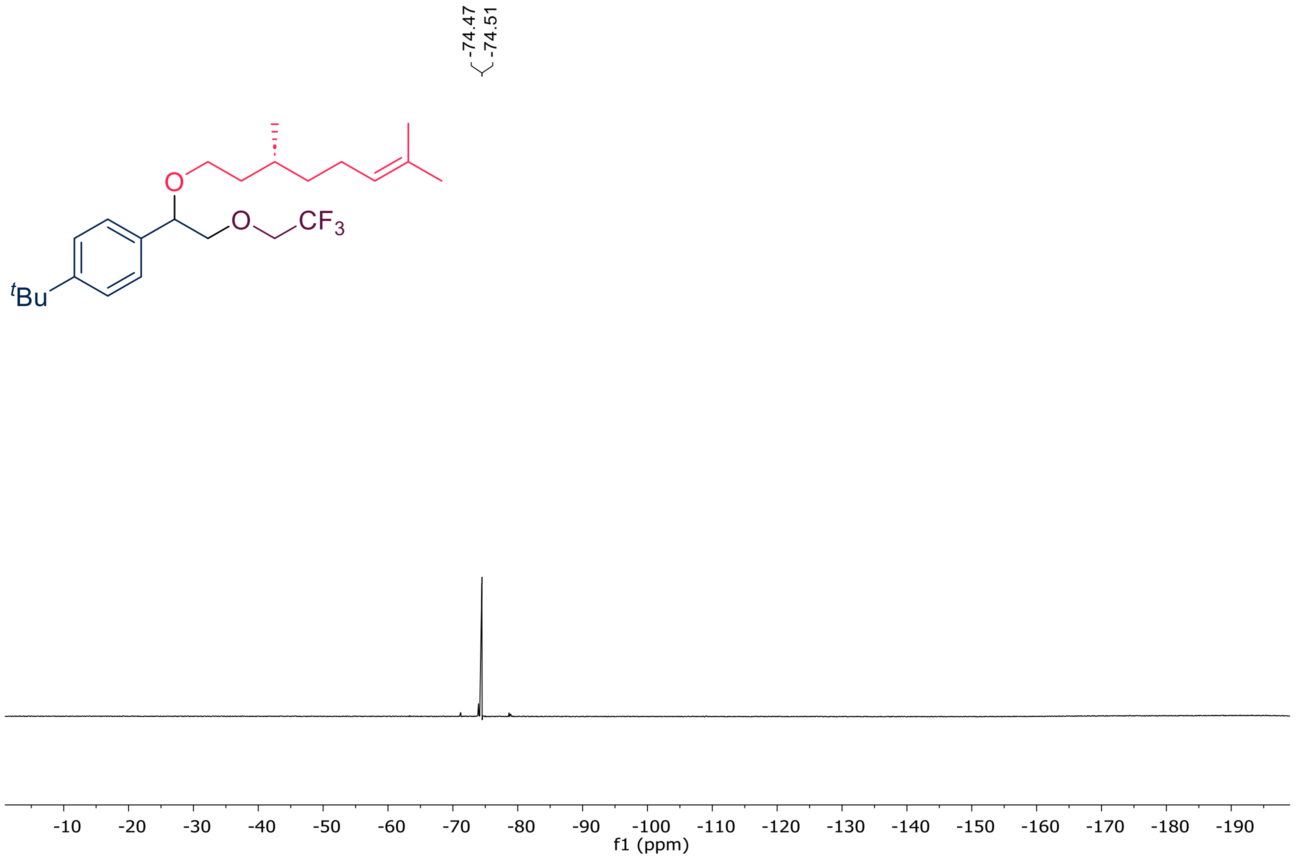

^{19}F NMR (282 MHz, CDCl$_3$) of compound **31**.

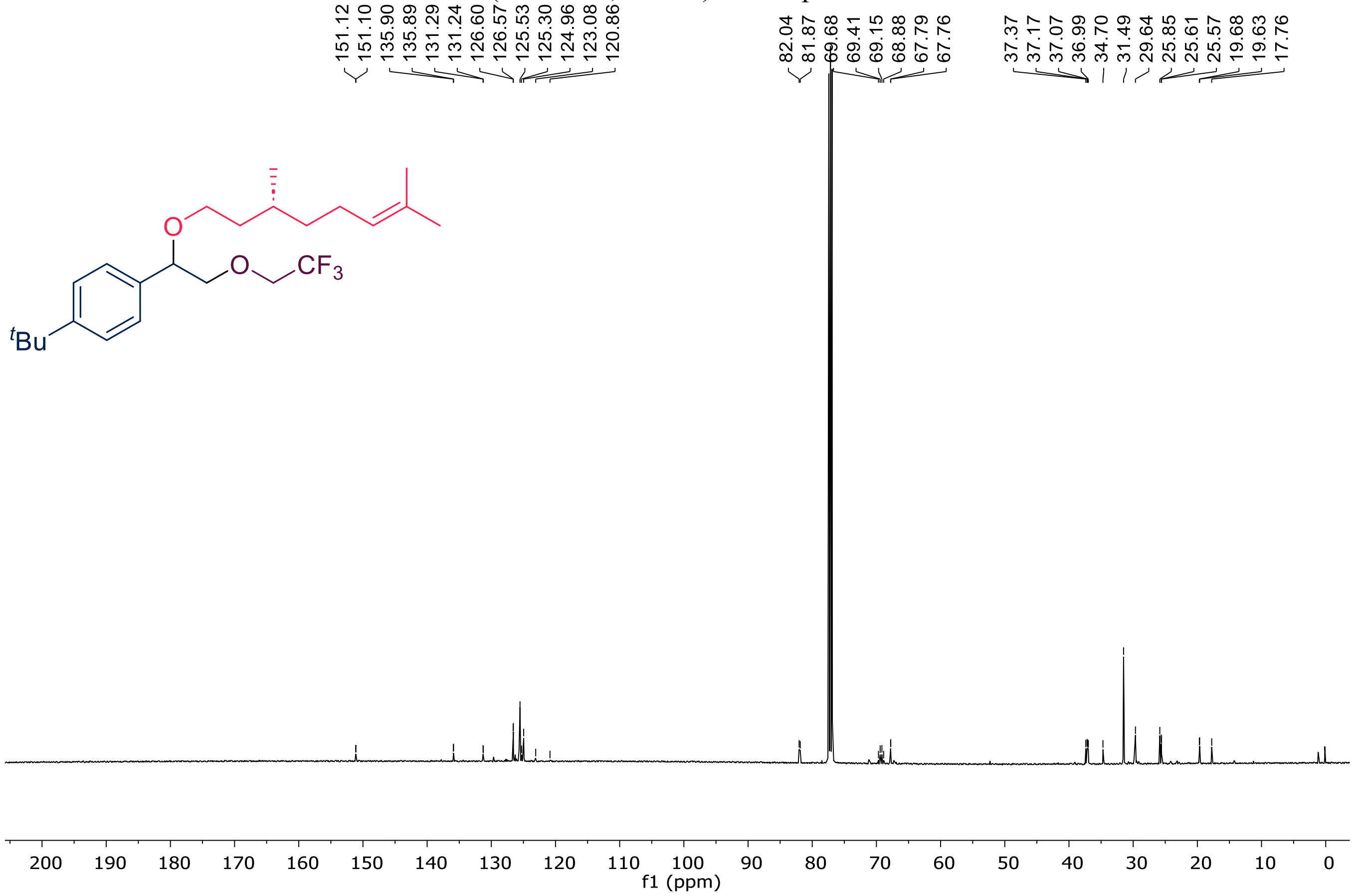

^{13}C NMR (126 MHz, CDCl$_3$) of compound **31**.

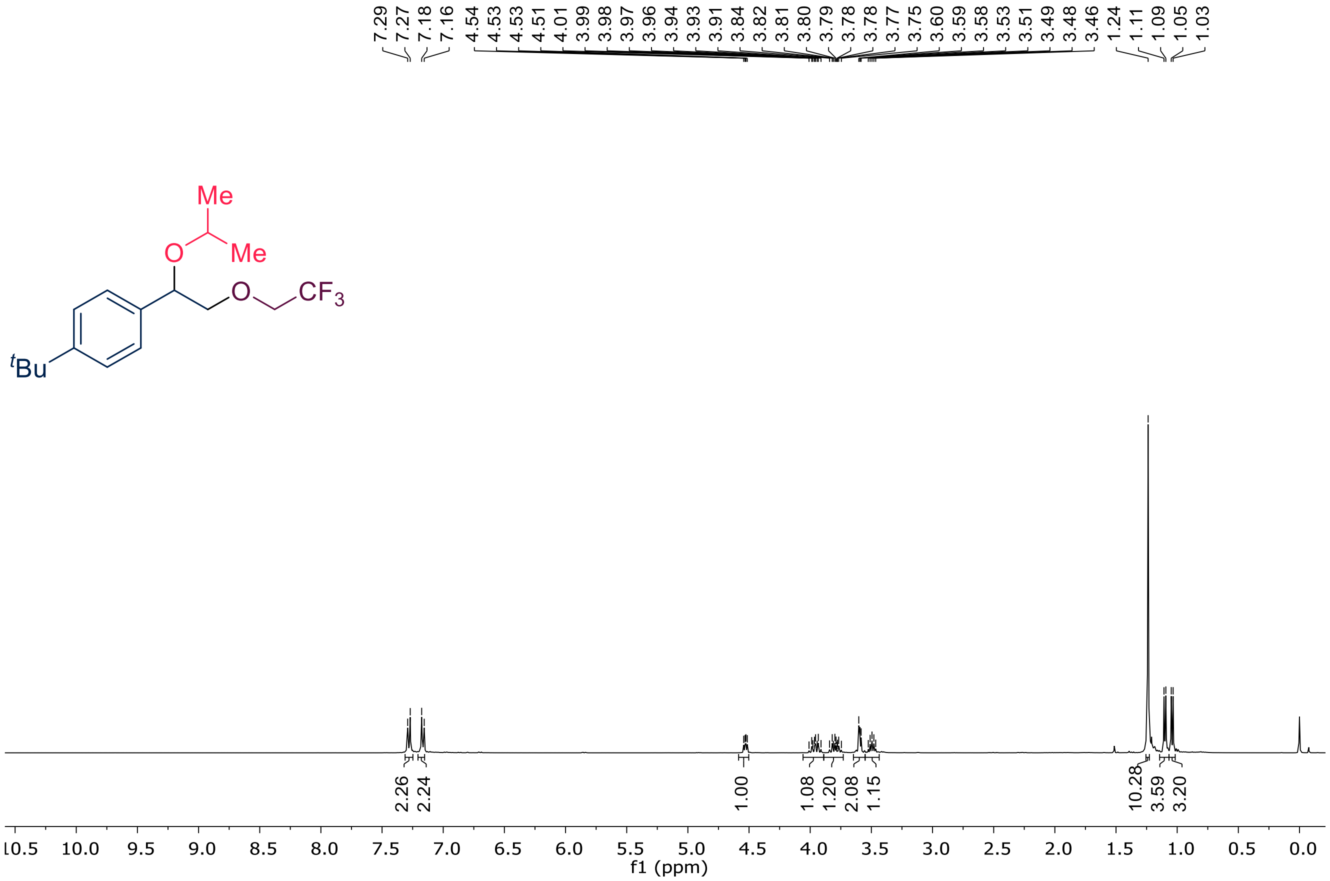

^{1}H NMR (400 MHz, $CDCl_3$) of compound **32**.

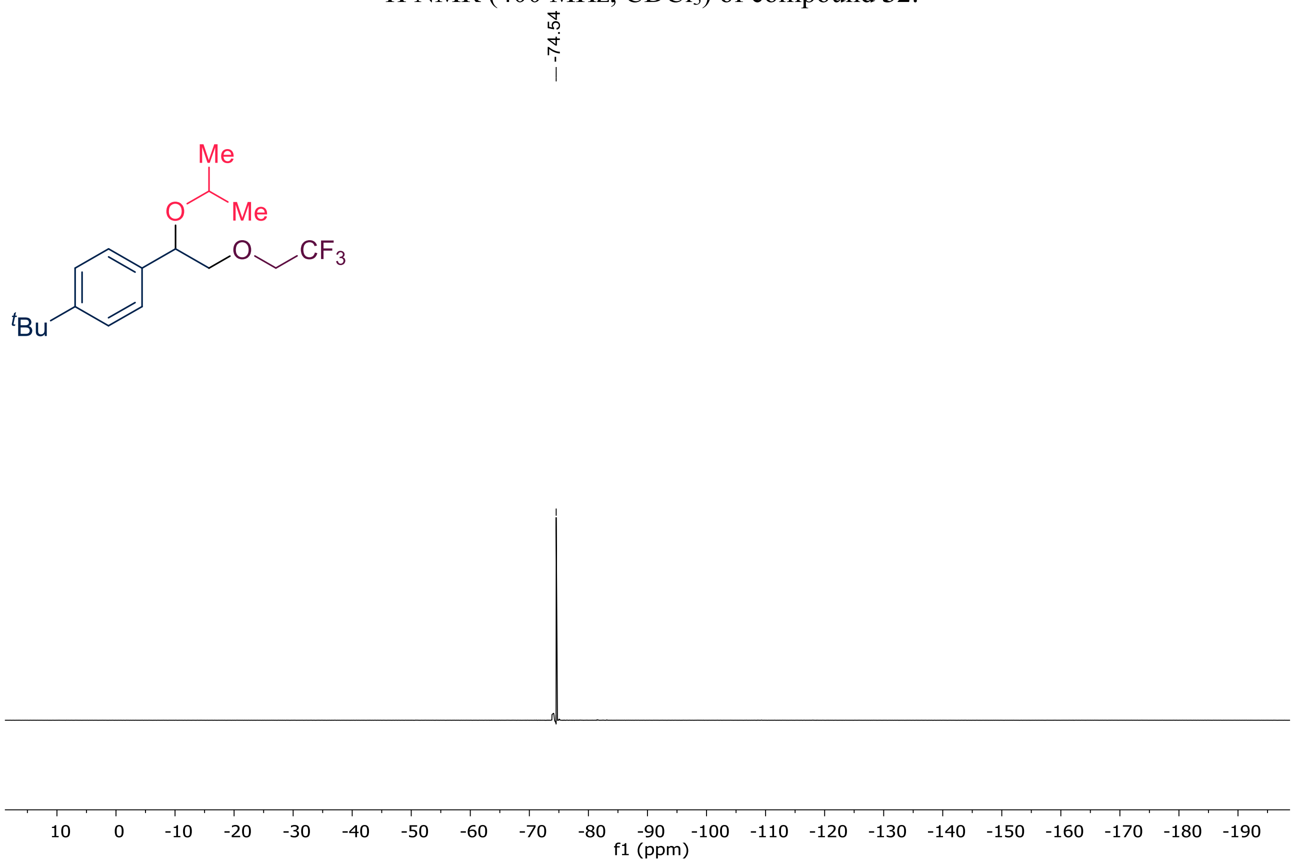

^{19}F NMR (377 MHz, $CDCl_3$) of compound **32**.

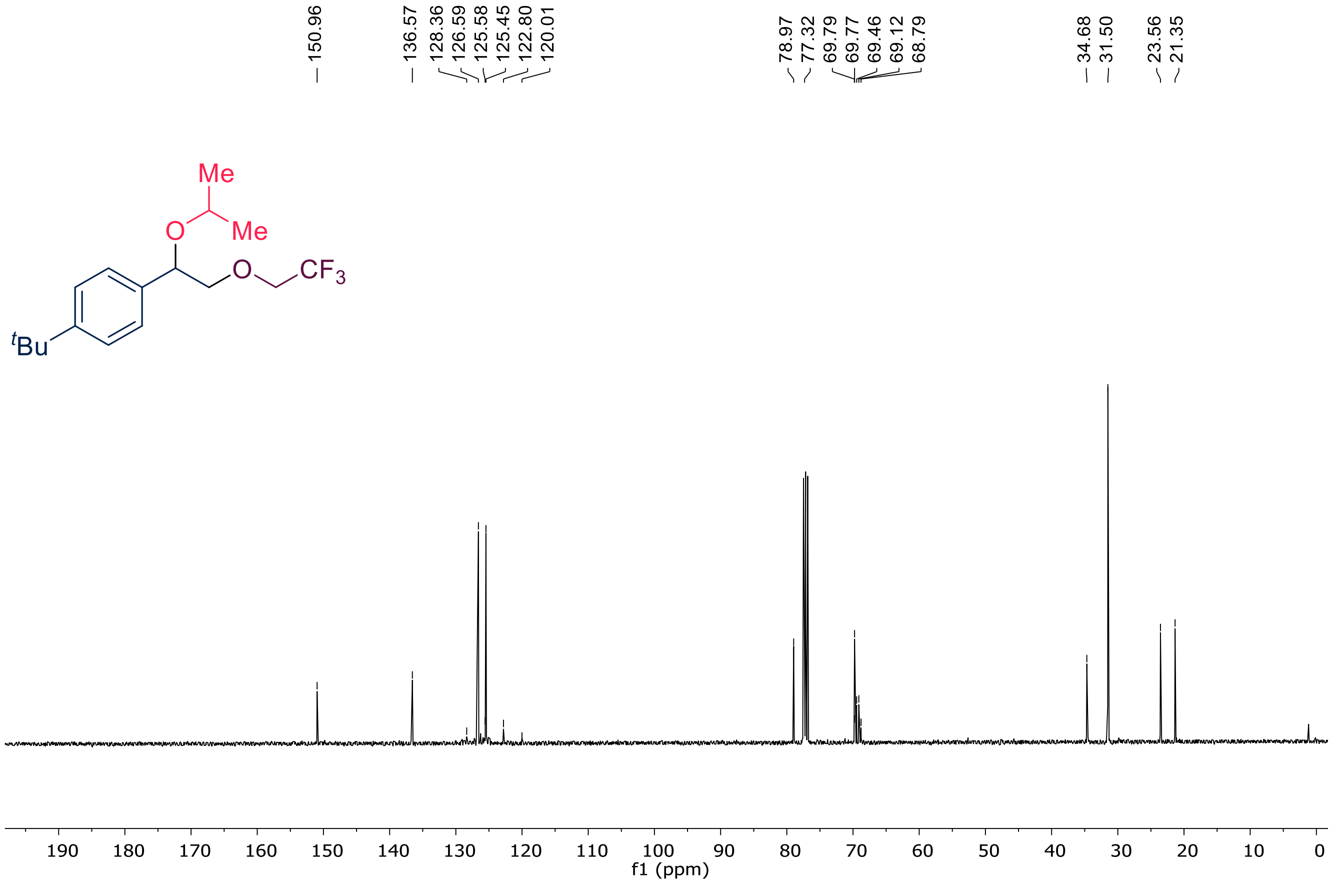

^{13}C NMR (101 MHz, CDCl$_3$) of compound **32**.

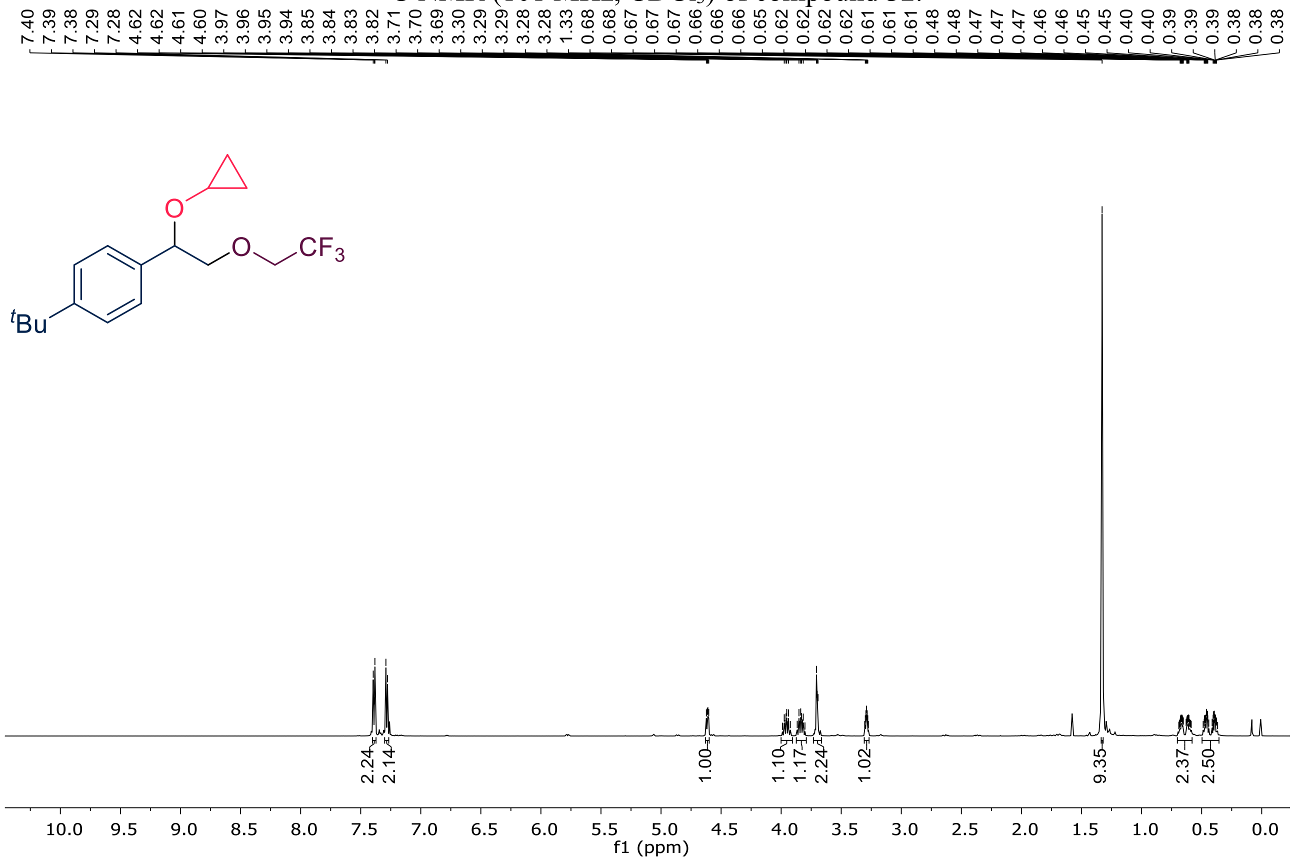

^{1}H NMR (600 MHz, CDCl$_3$) of compound **33**.

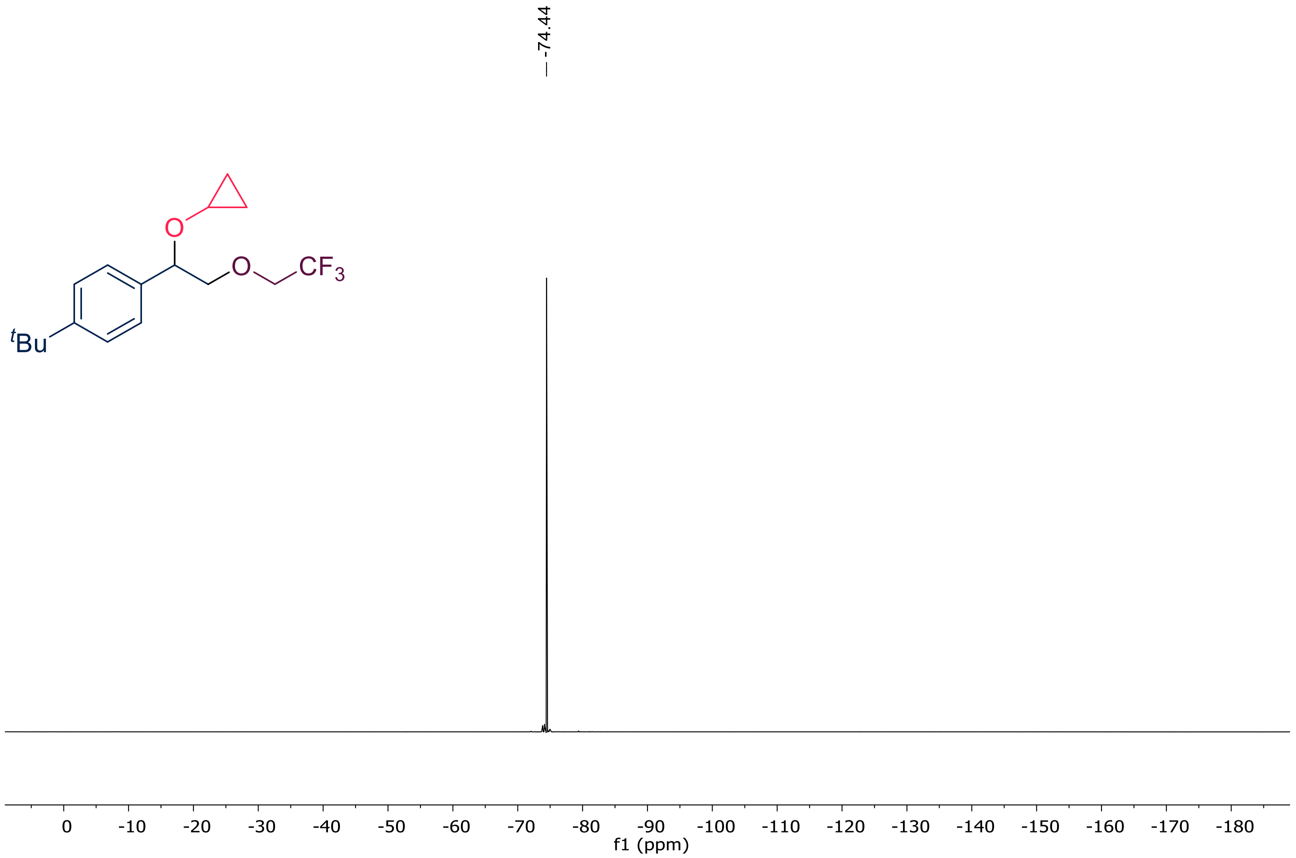

^{19}F NMR (377 MHz, $CDCl_3$) of compound **33**.

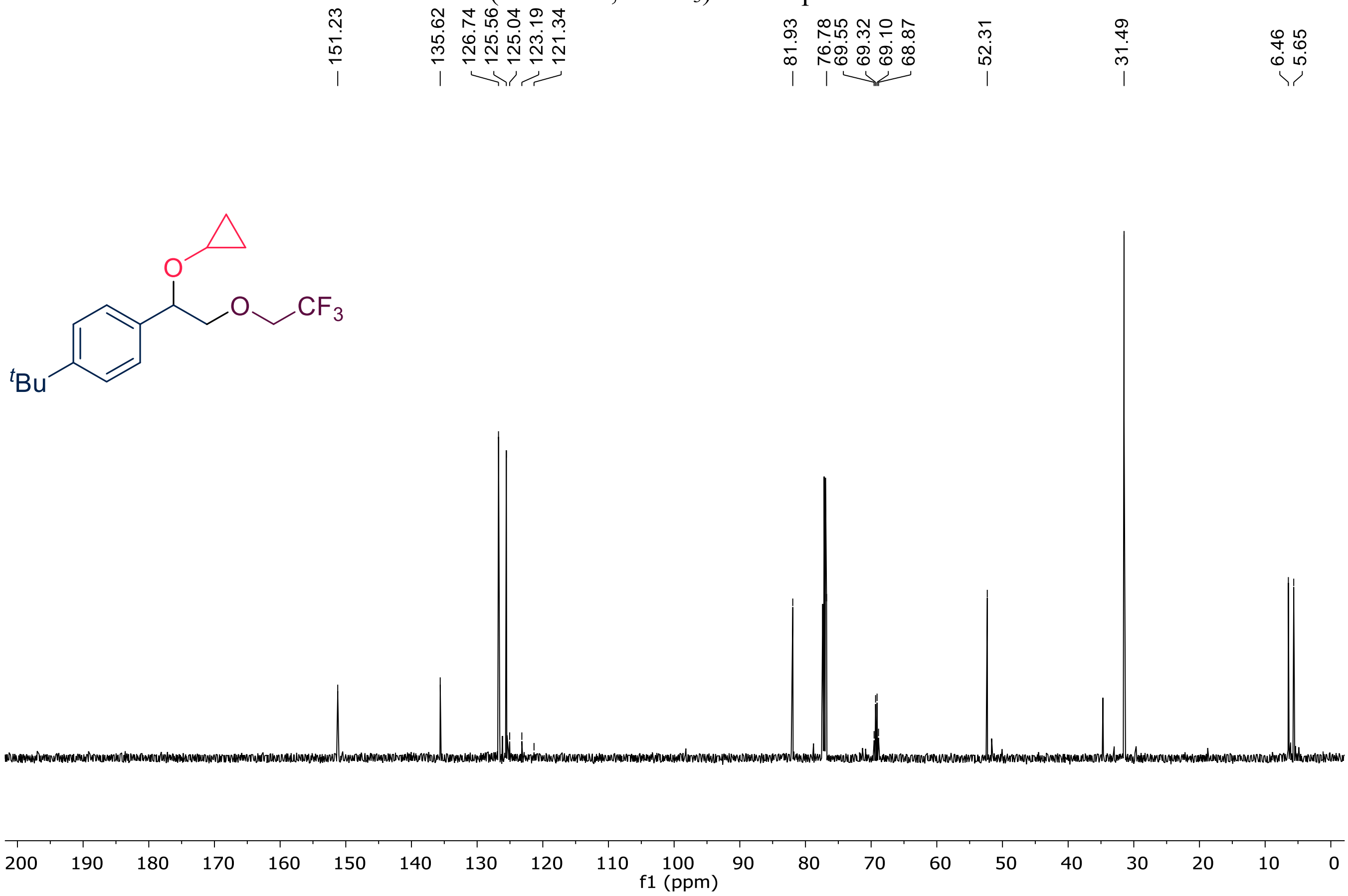

^{13}C NMR (101 MHz, $CDCl_3$) of compound **33**.

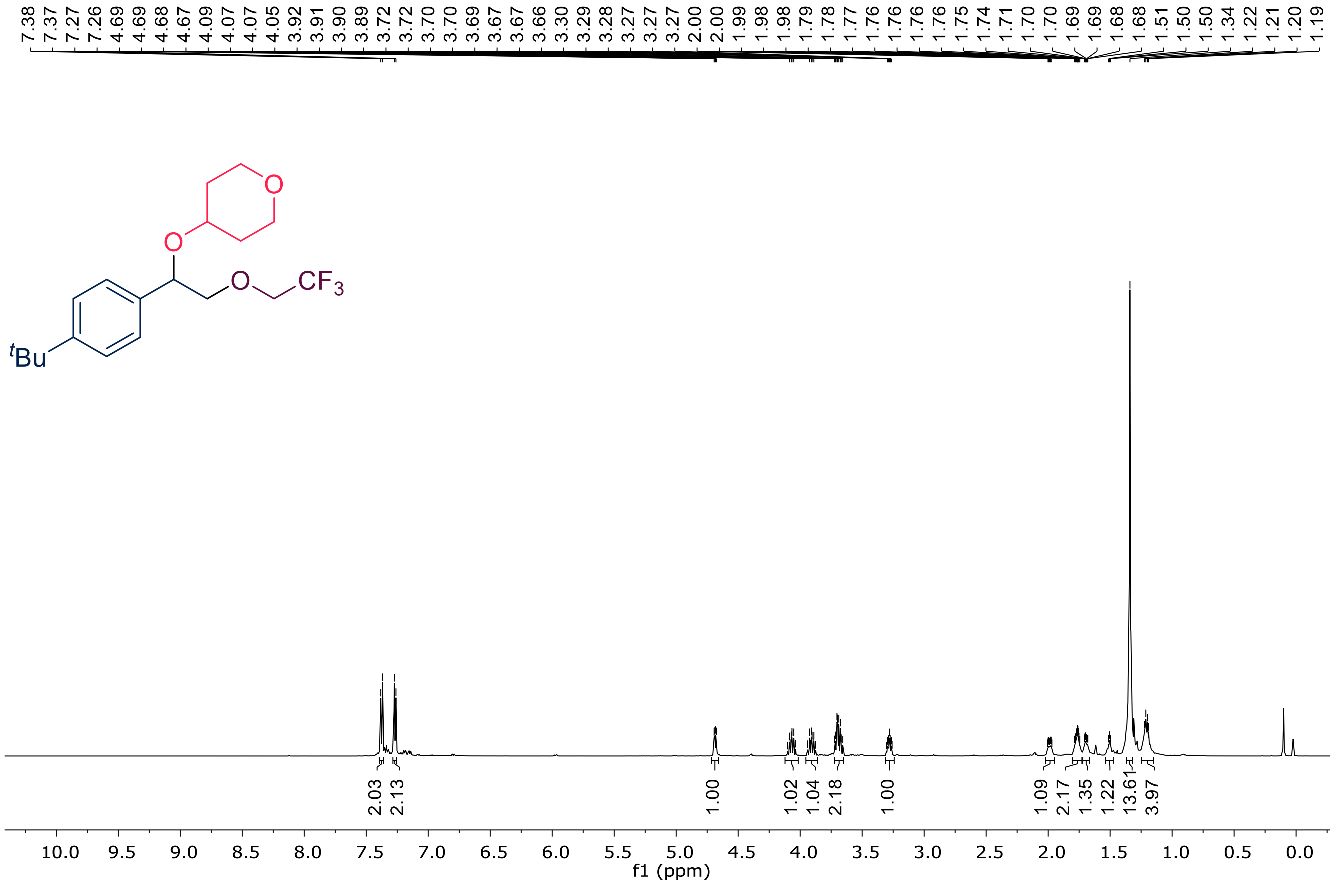

^{1}H NMR (600 MHz, $CDCl_3$) of compound **34**.

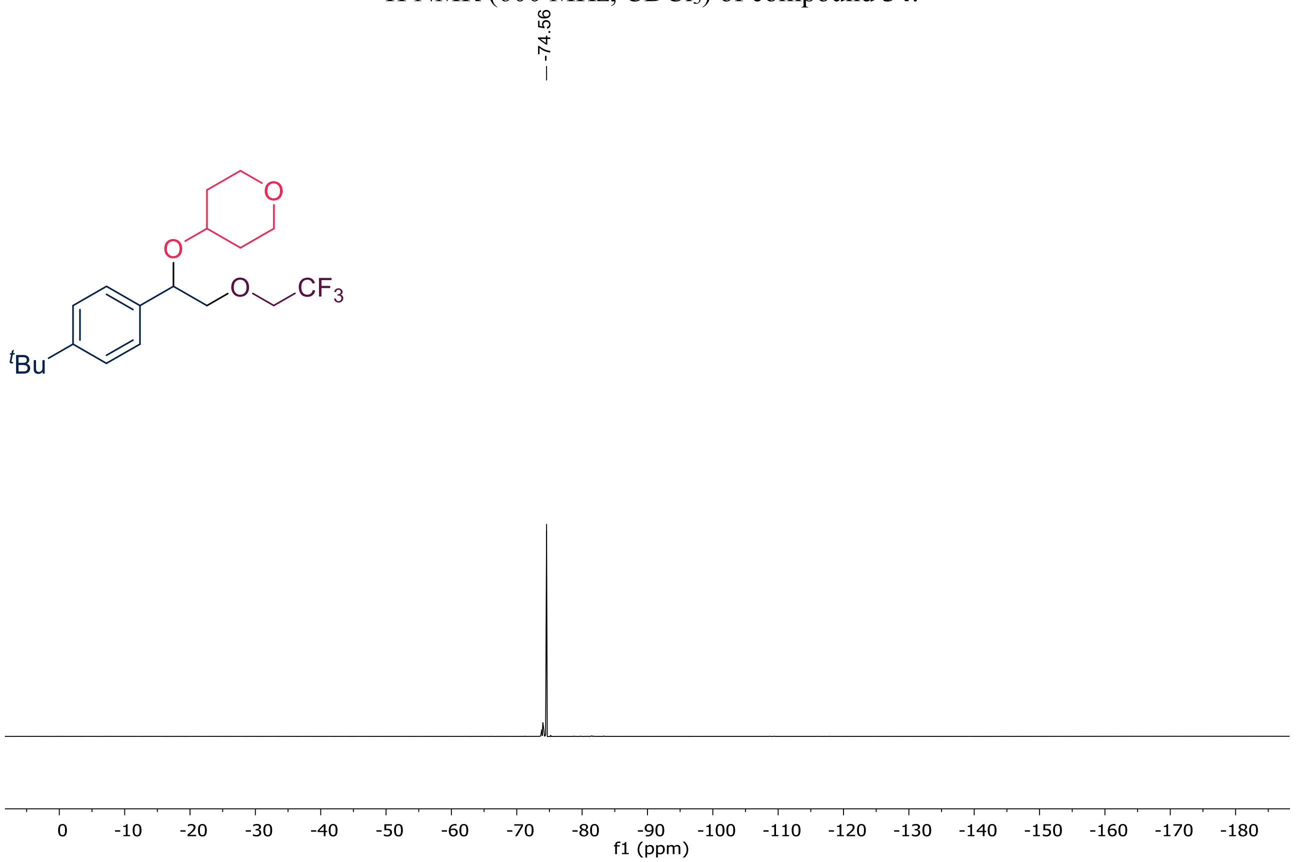

^{19}F NMR (377 MHz, $CDCl_3$) of compound **34**.

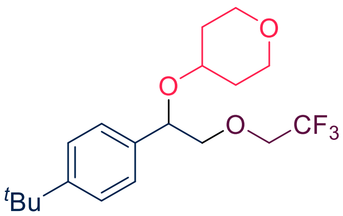

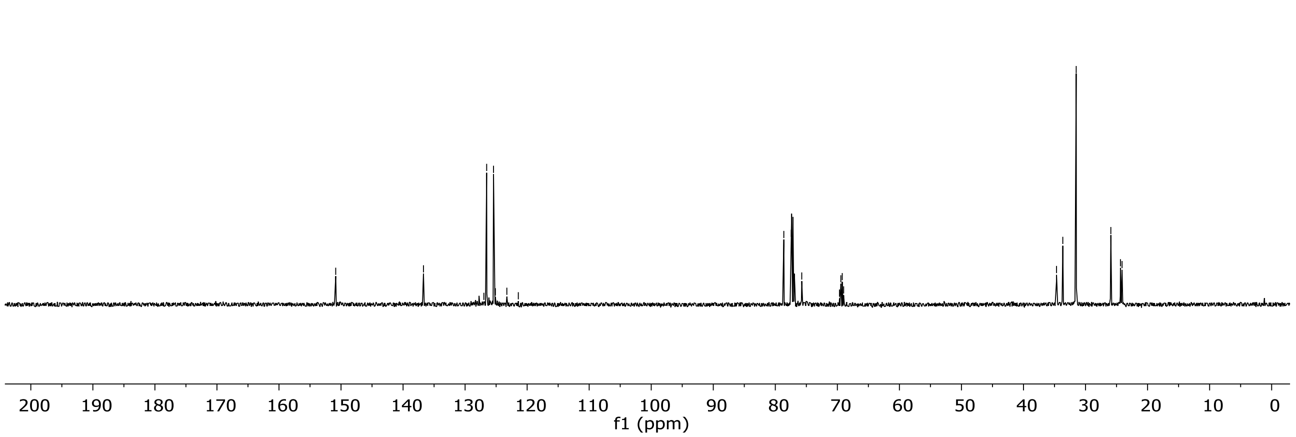

^{13}C NMR (151 MHz, $CDCl_3$) of compound **34**.

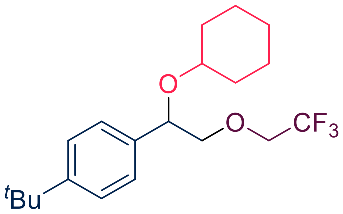

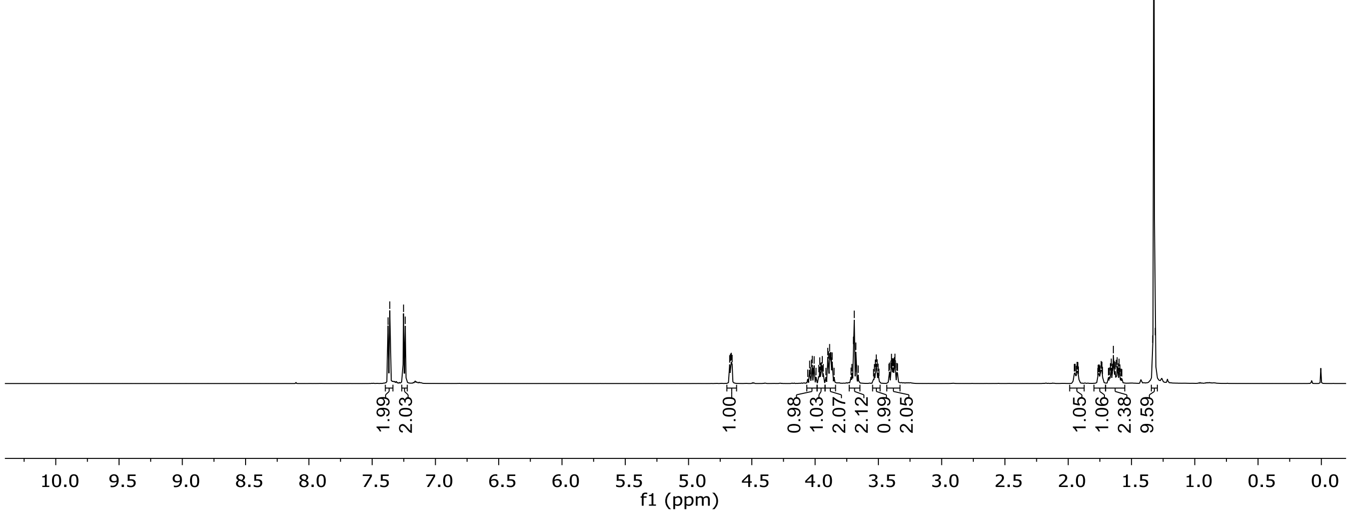

^{1}H NMR (600 MHz, $CDCl_3$) of compound **35**.

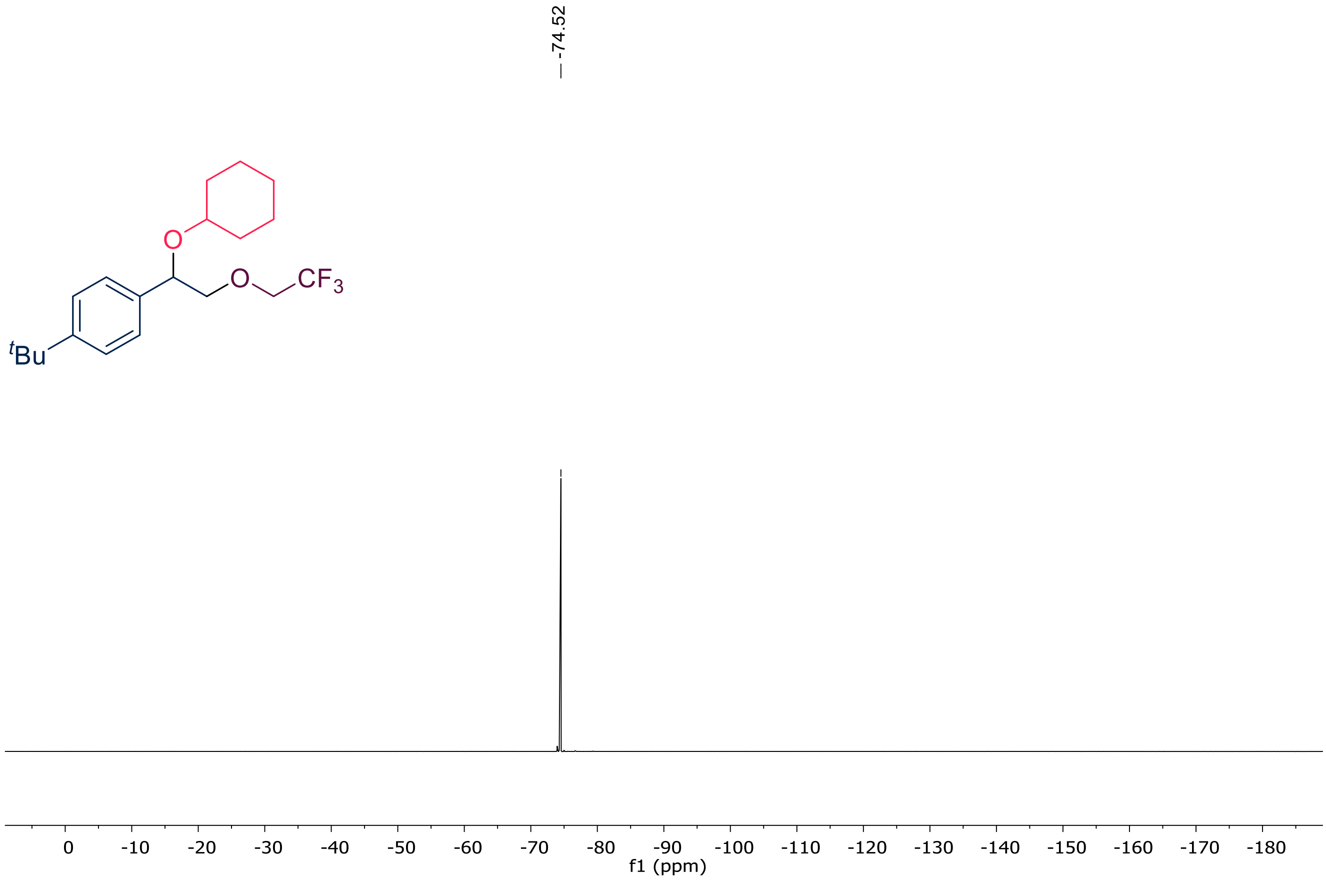

^{19}F NMR (282 MHz, $CDCl_3$) of compound **35**.

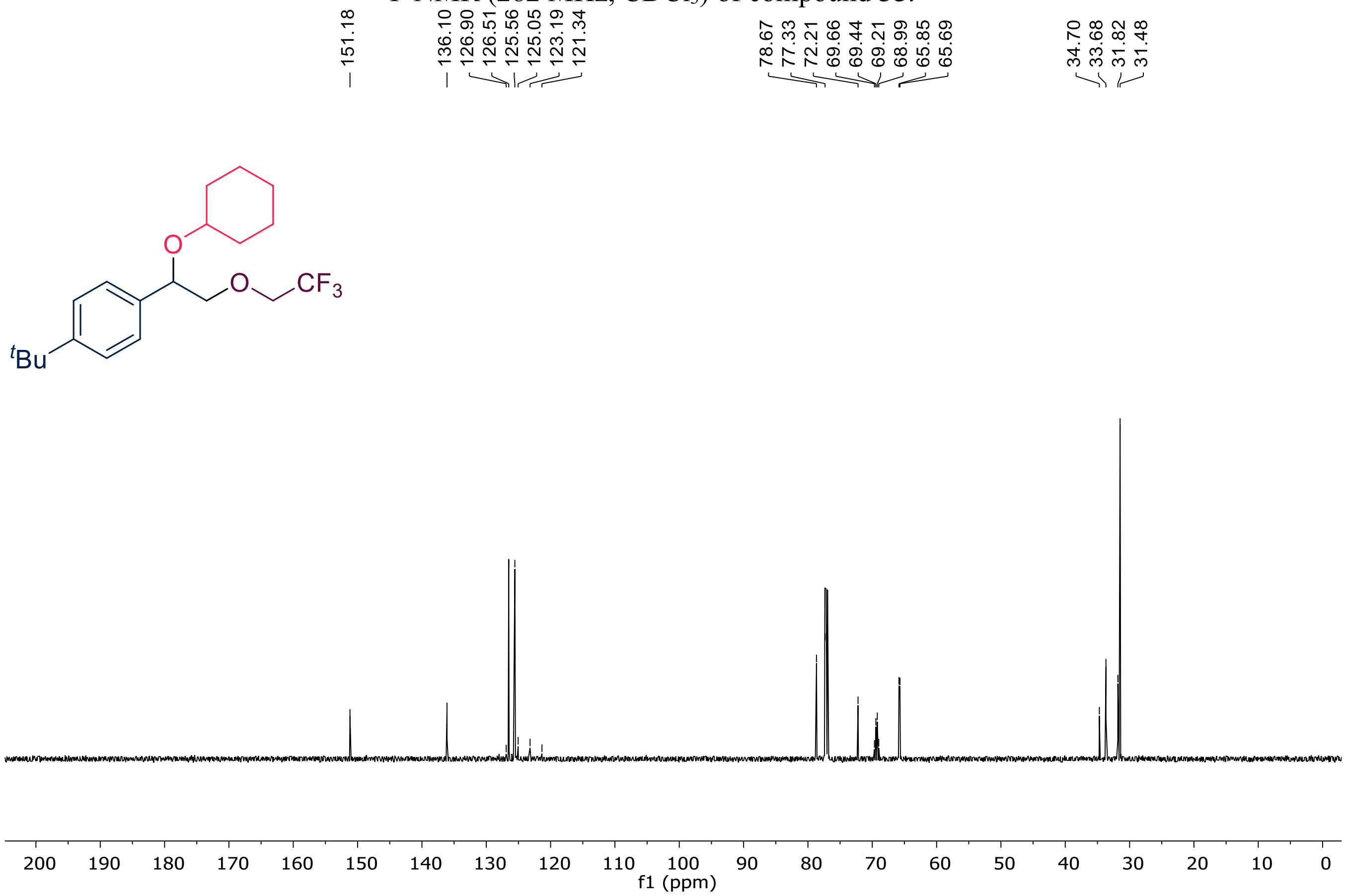

^{13}C NMR (151 MHz, $CDCl_3$) of compound **35**.

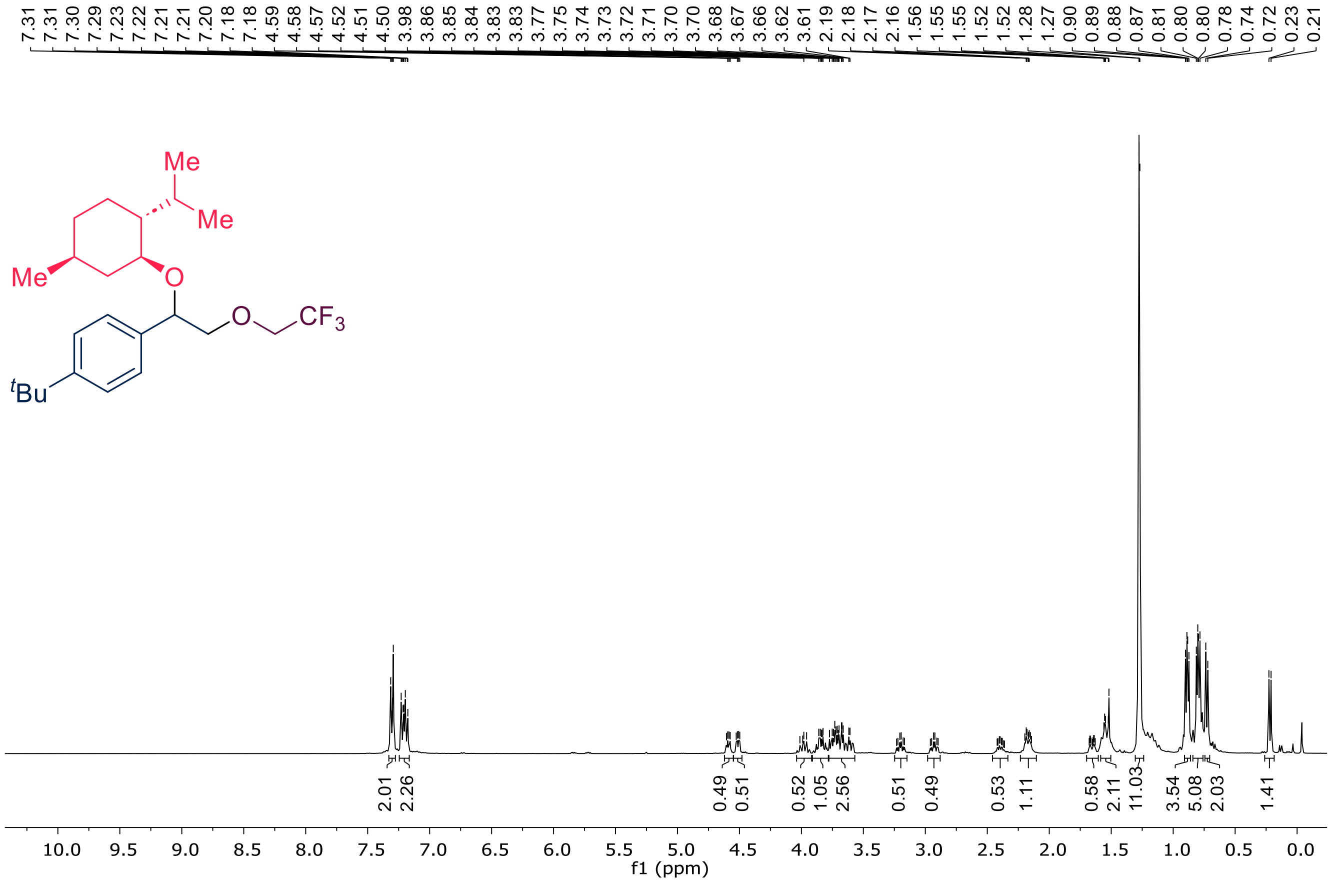

^{1}H NMR (400 MHz, $CDCl_3$) of compound **36**.

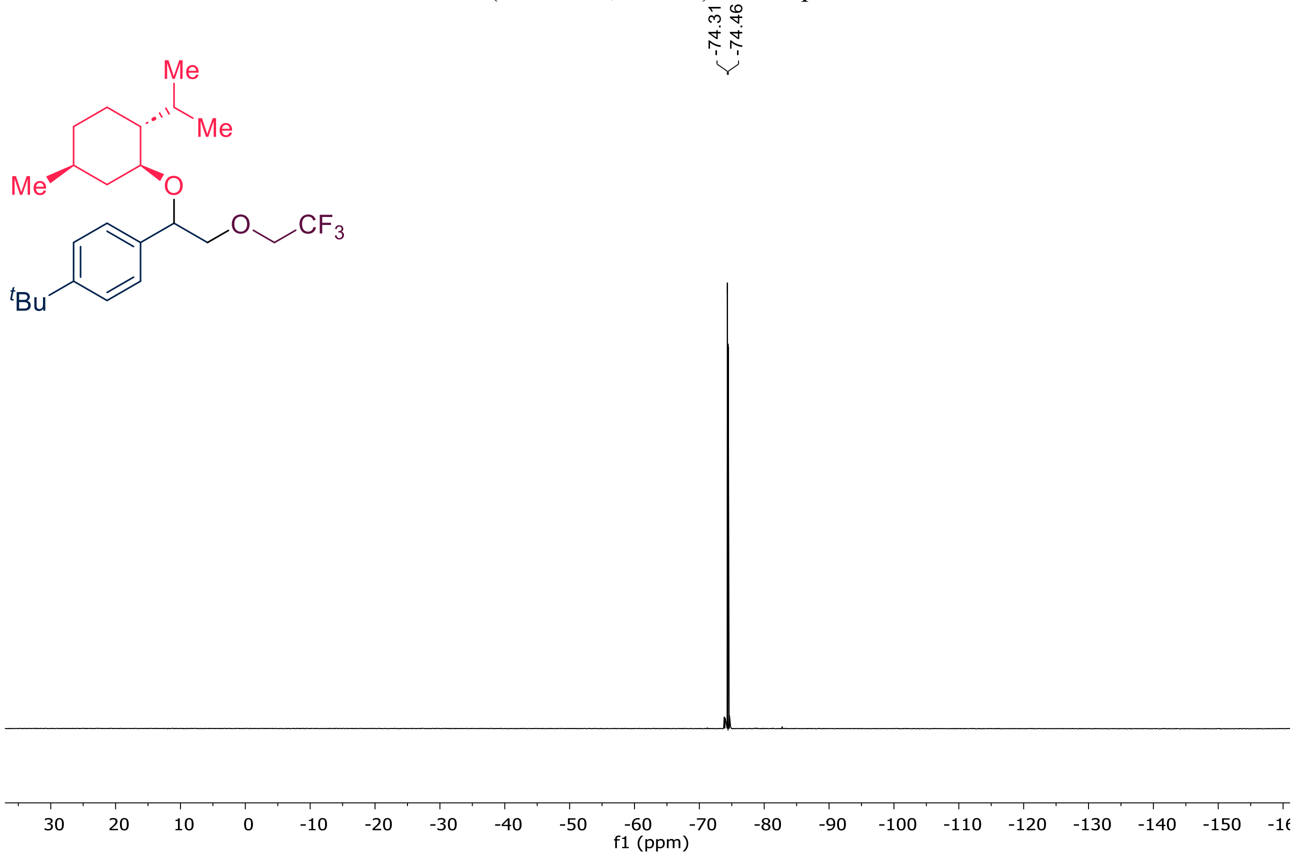

^{19}F NMR (377 MHz, $CDCl_3$) of compound **36**.

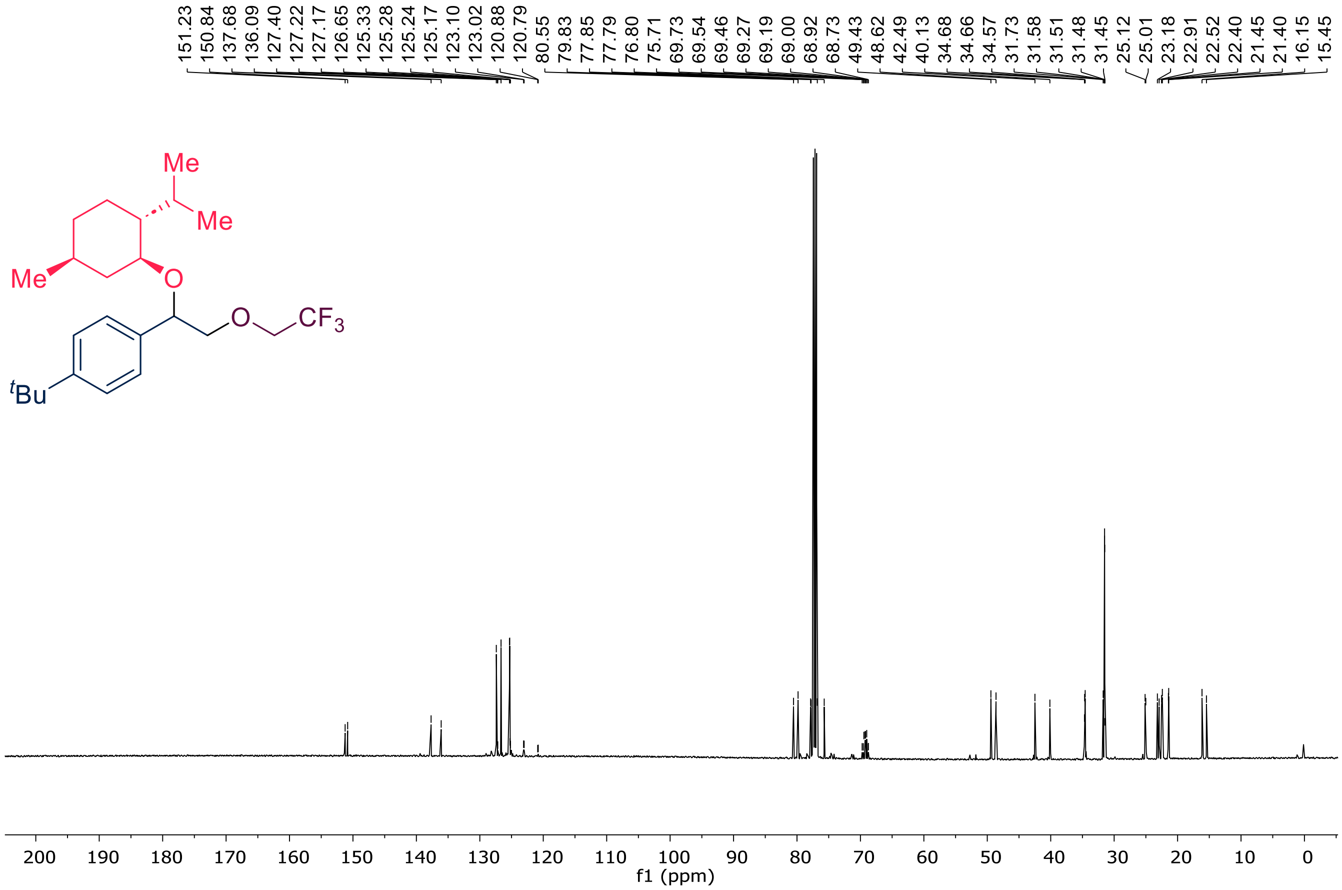

^{13}C NMR (101 MHz, $CDCl_3$) of compound **36**.

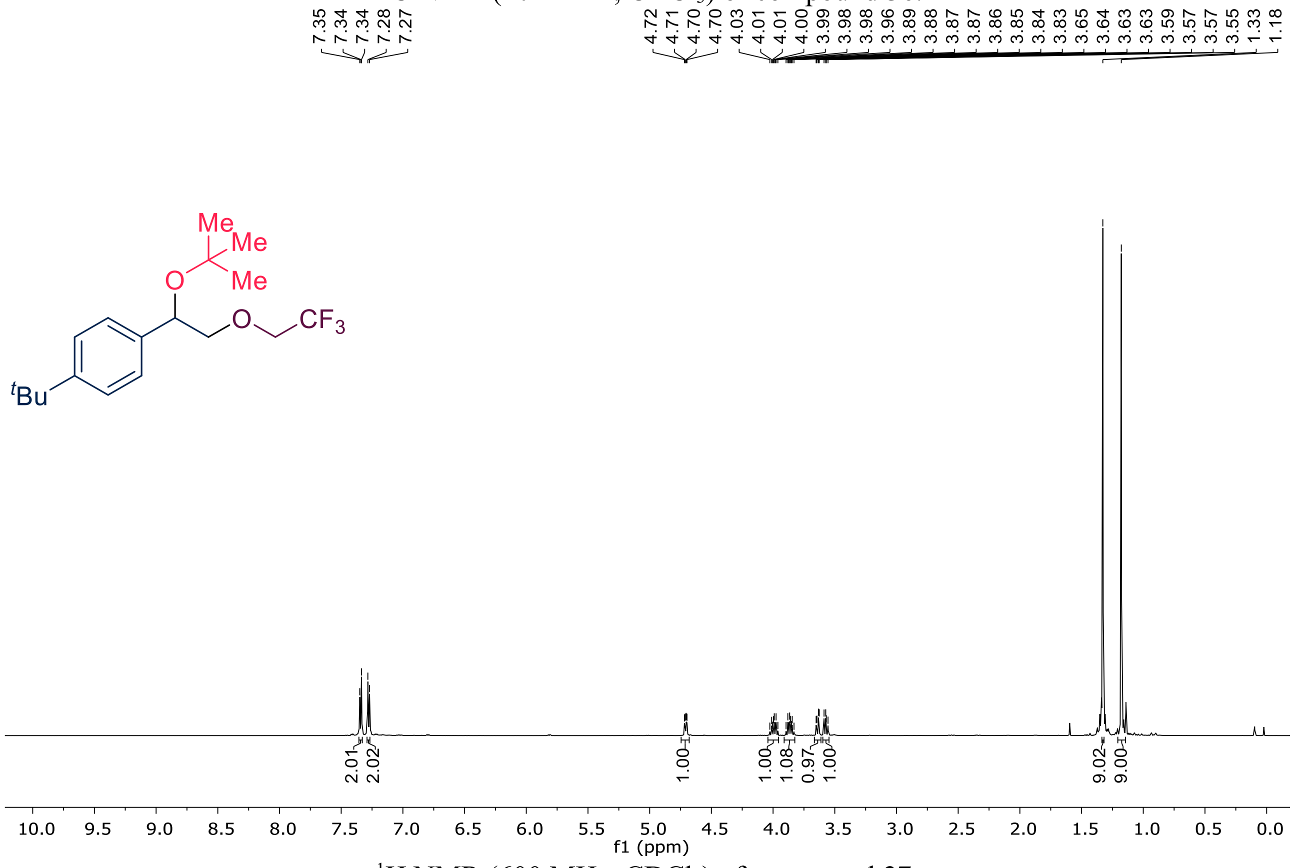

^{1}H NMR (600 MHz, $CDCl_3$) of compound **37**.

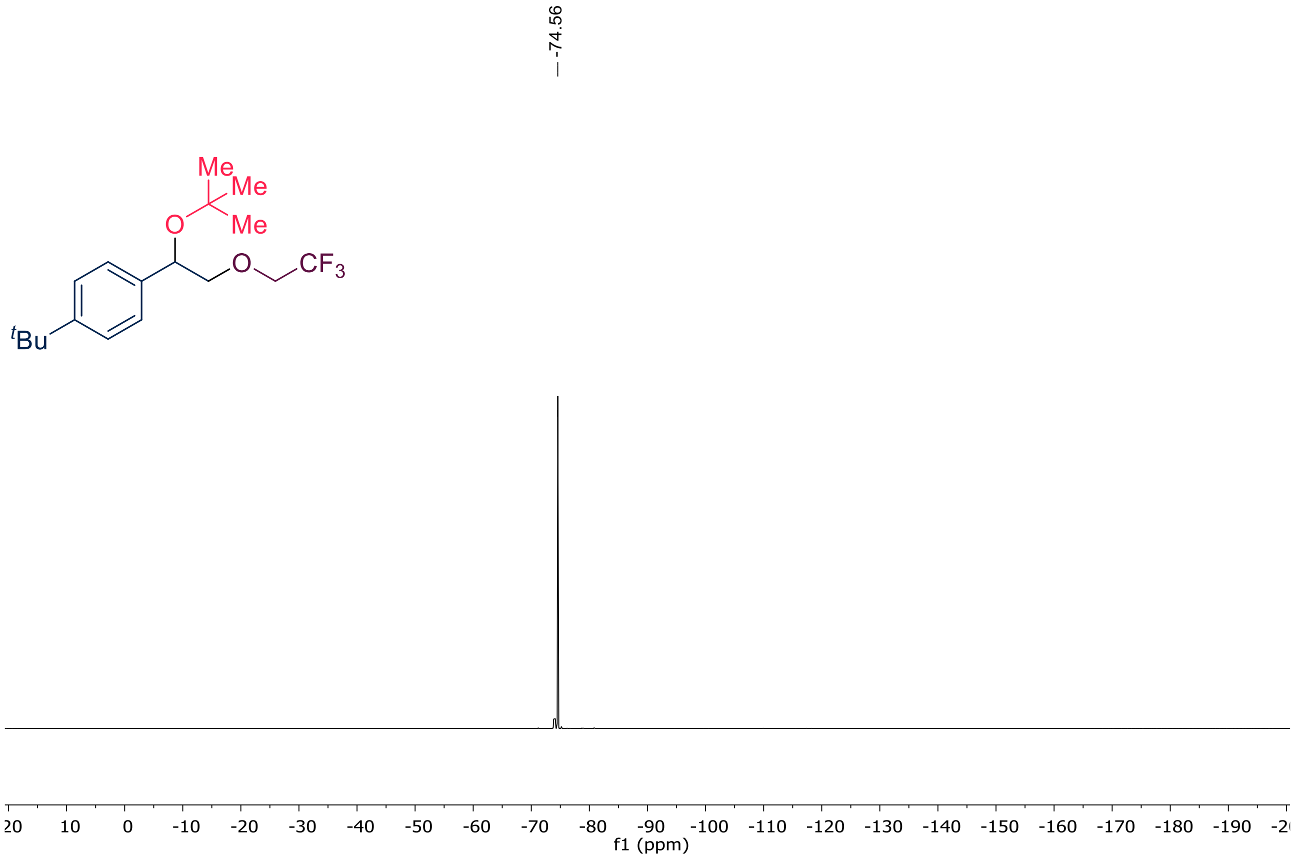

^{19}F NMR (282 MHz, CDCl$_3$) of compound **37**.

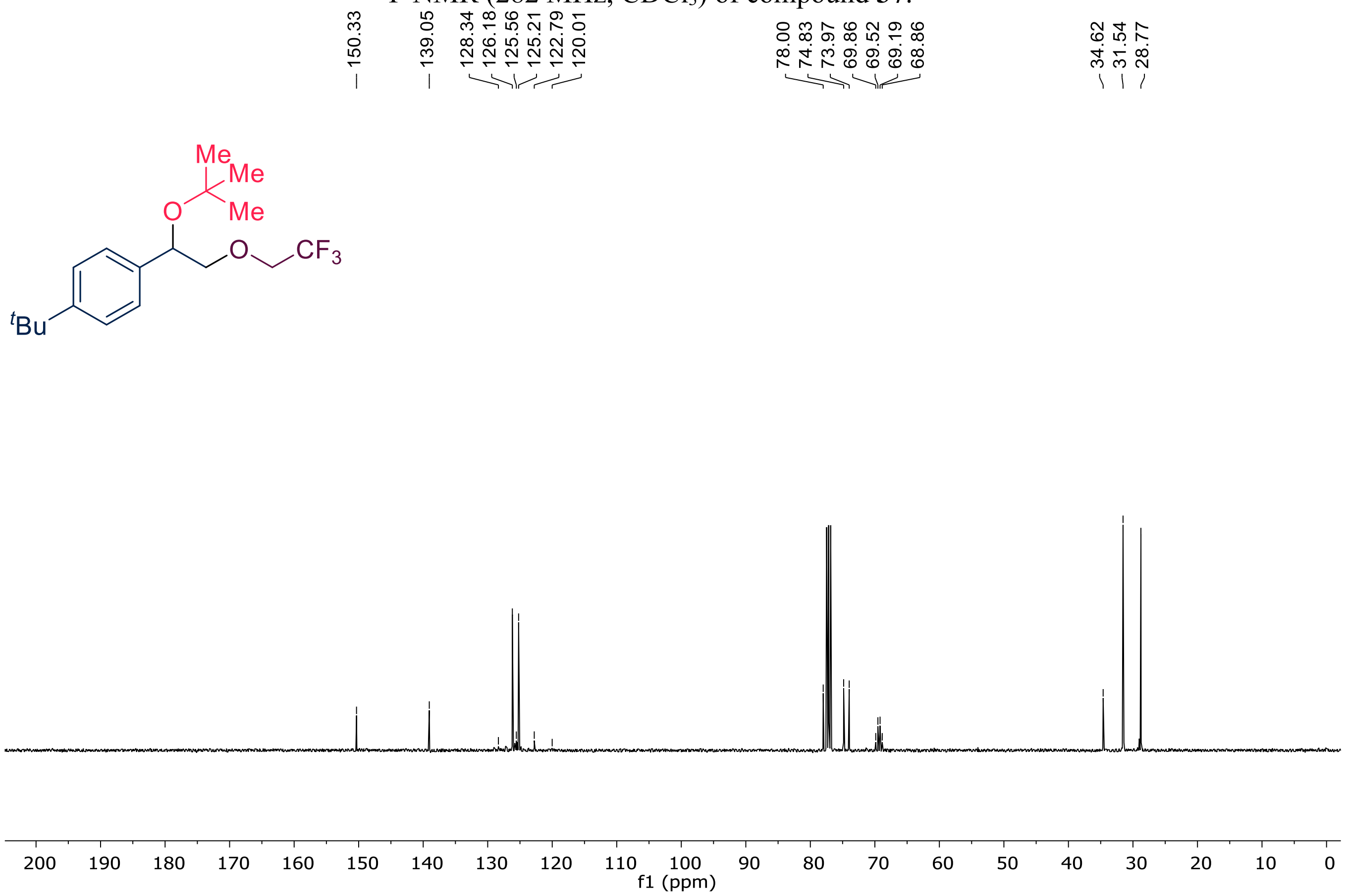

^{13}C NMR (101 MHz, CDCl$_3$) of compound **37**.

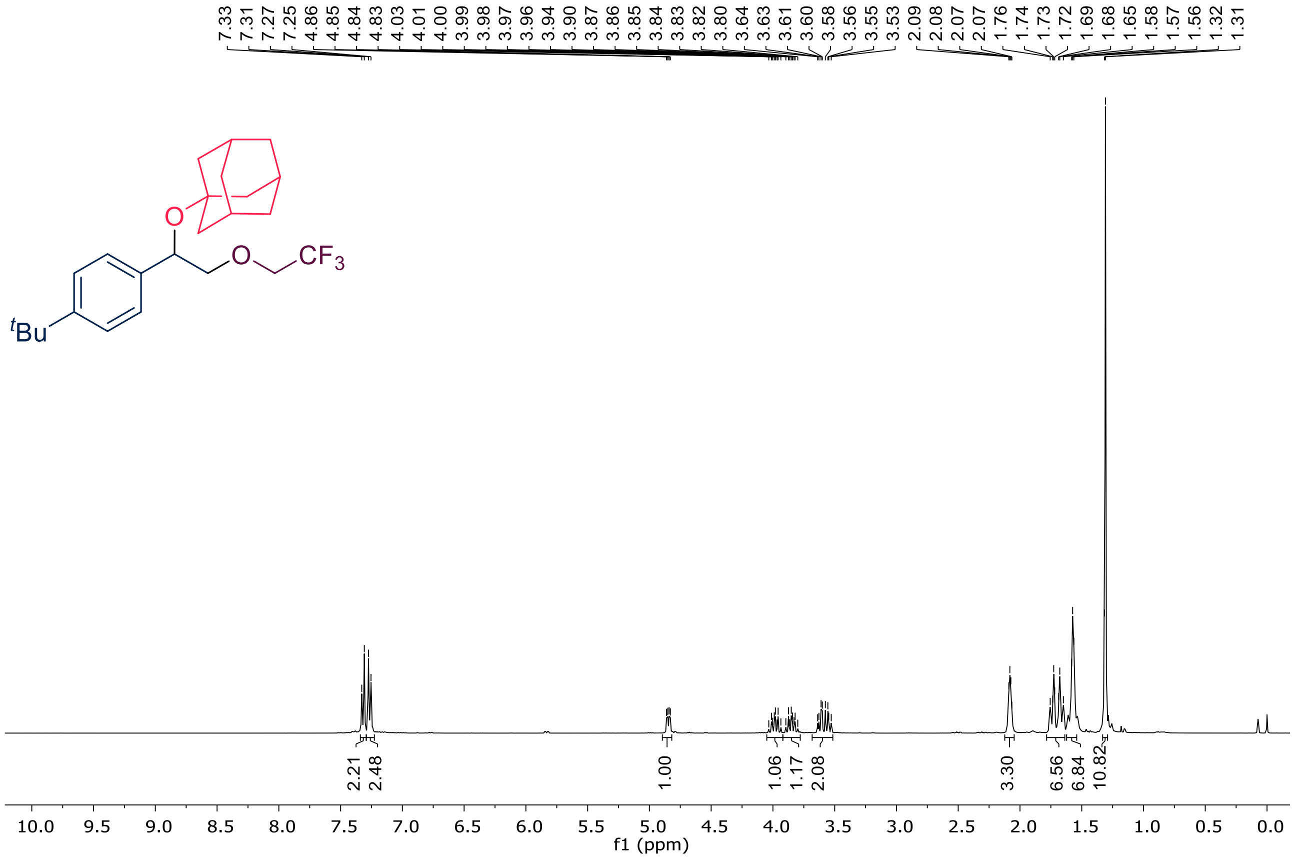

^{1}H NMR (400 MHz, CDCl3) of compound **38**.

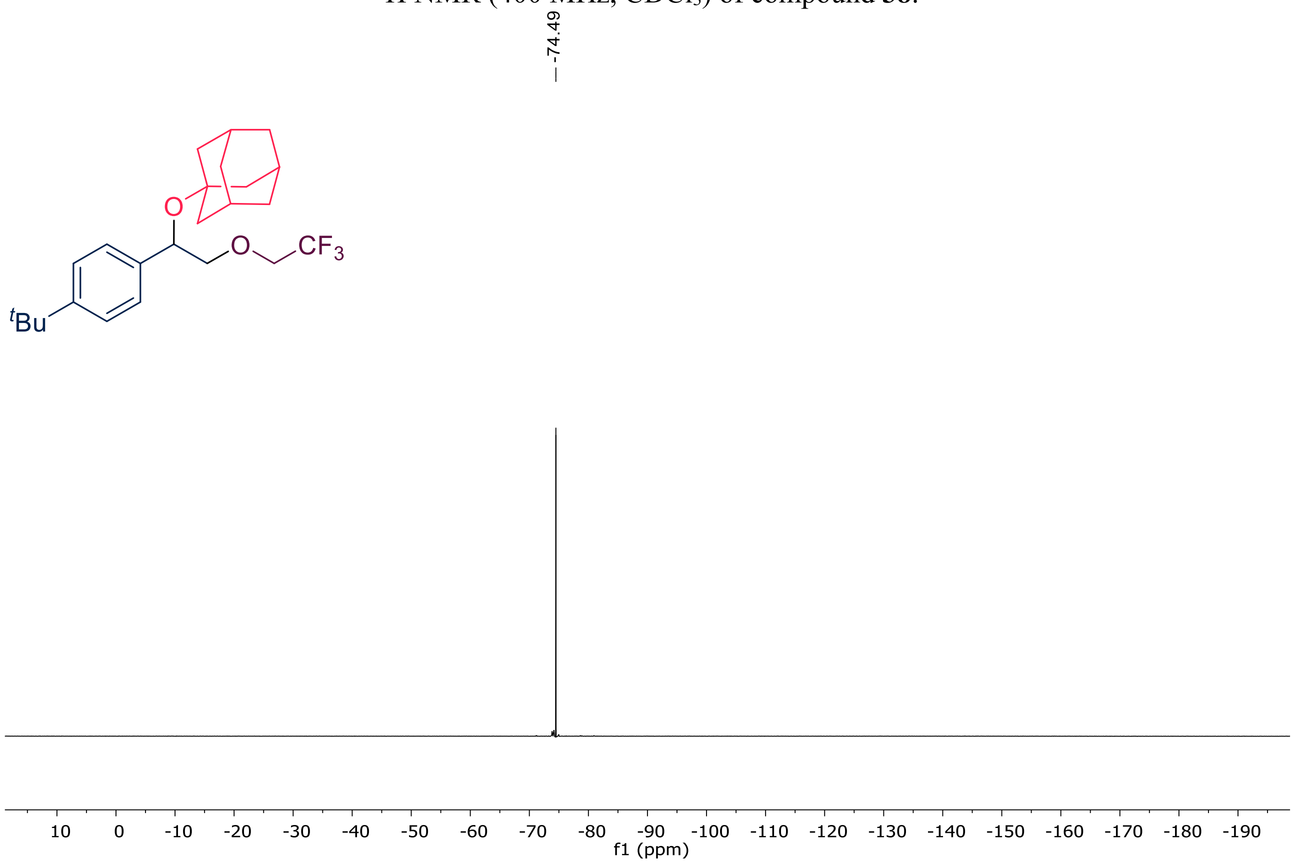

^{19}F NMR (377 MHz, CDCl3) of compound **38**.

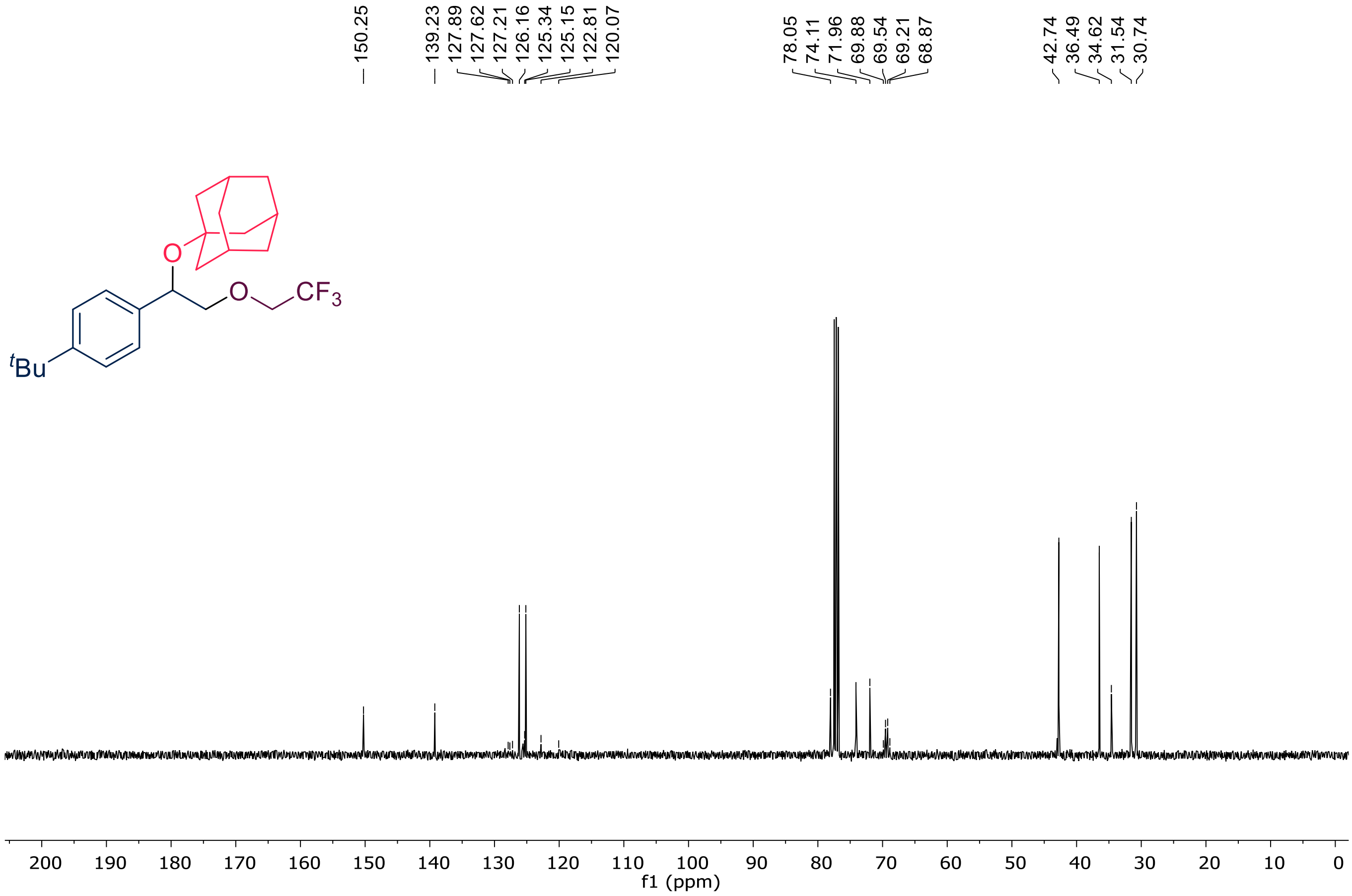

^{13}C NMR (101 MHz, CDCl$_3$) of compound **38**.

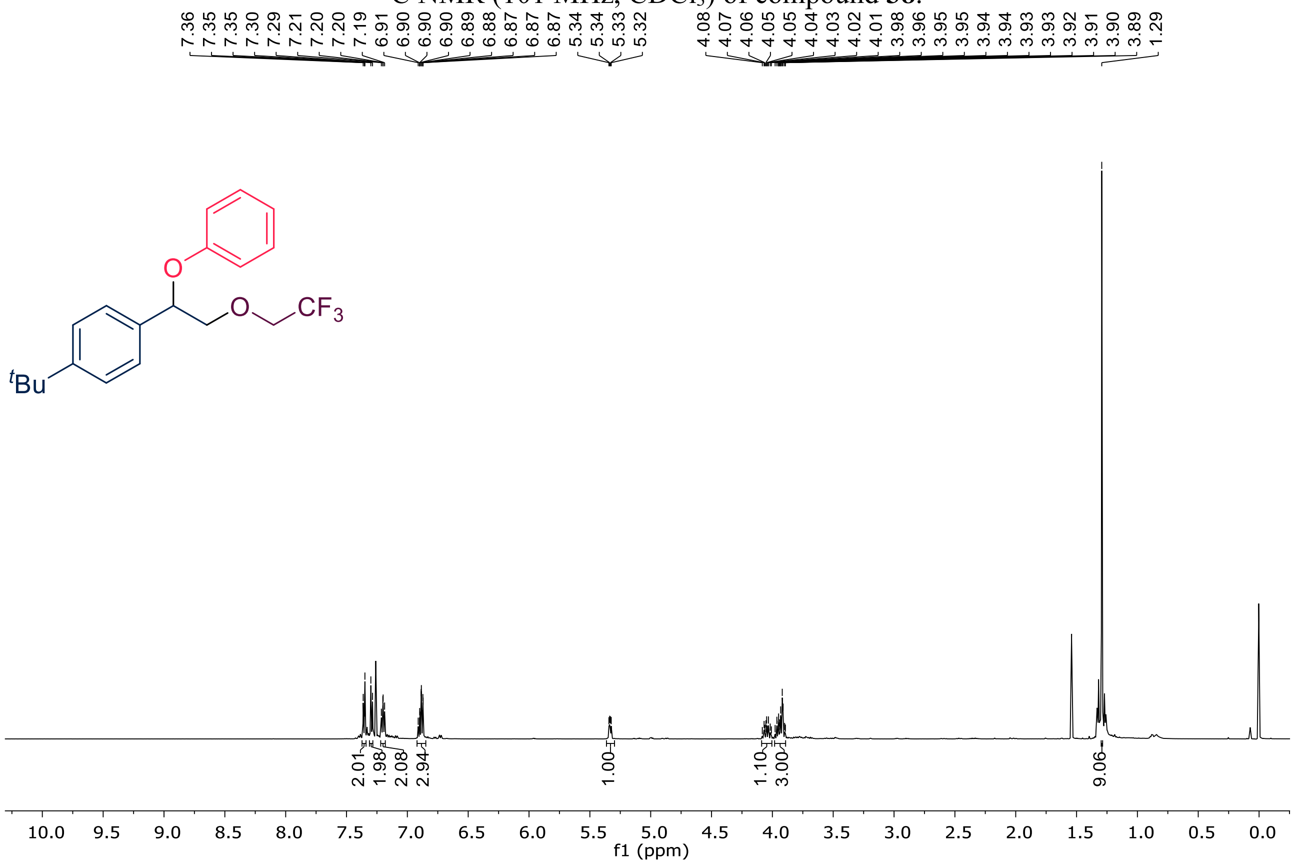

^{1}H NMR (600 MHz, CDCl$_3$) of compound **39**.

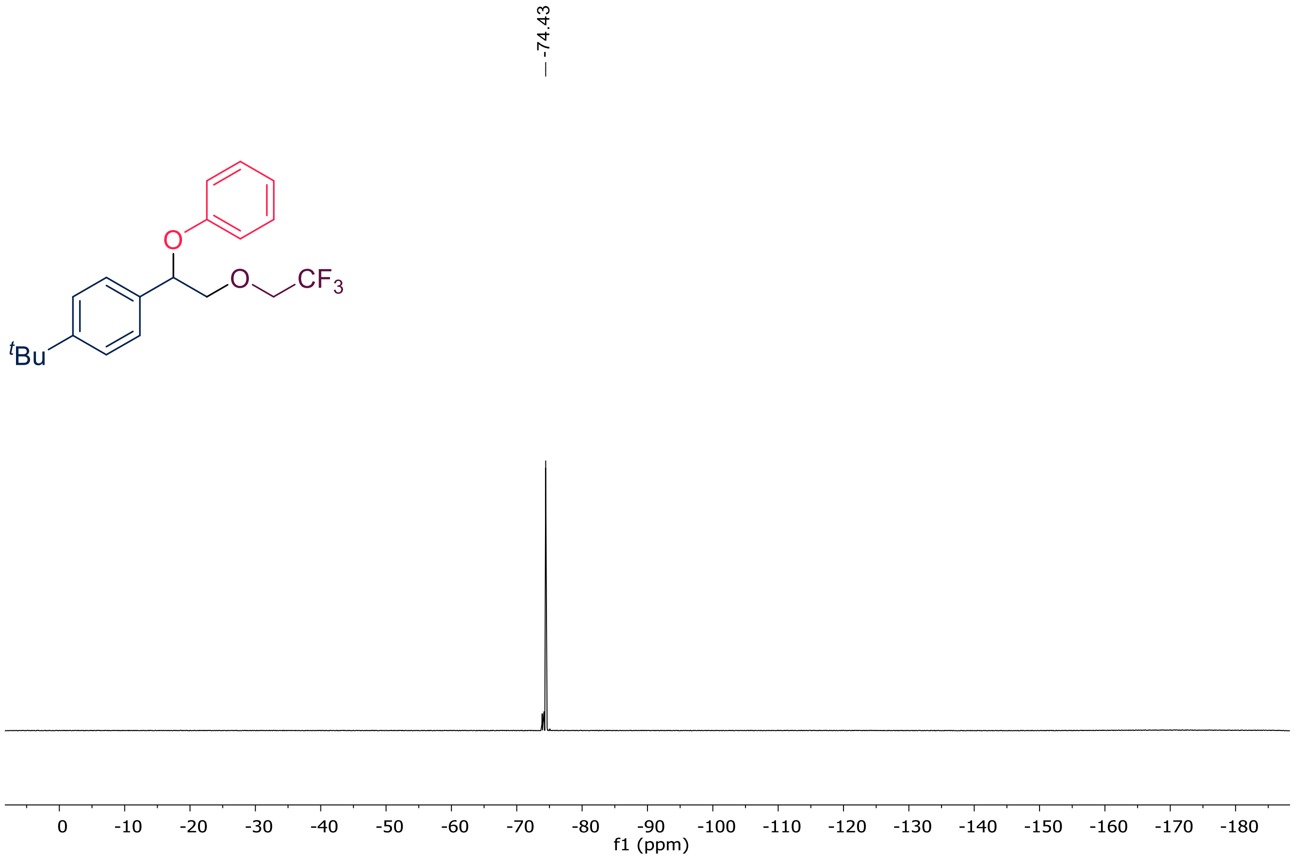

^{19}F NMR (282 MHz, CDCl$_3$) of compound **39**.

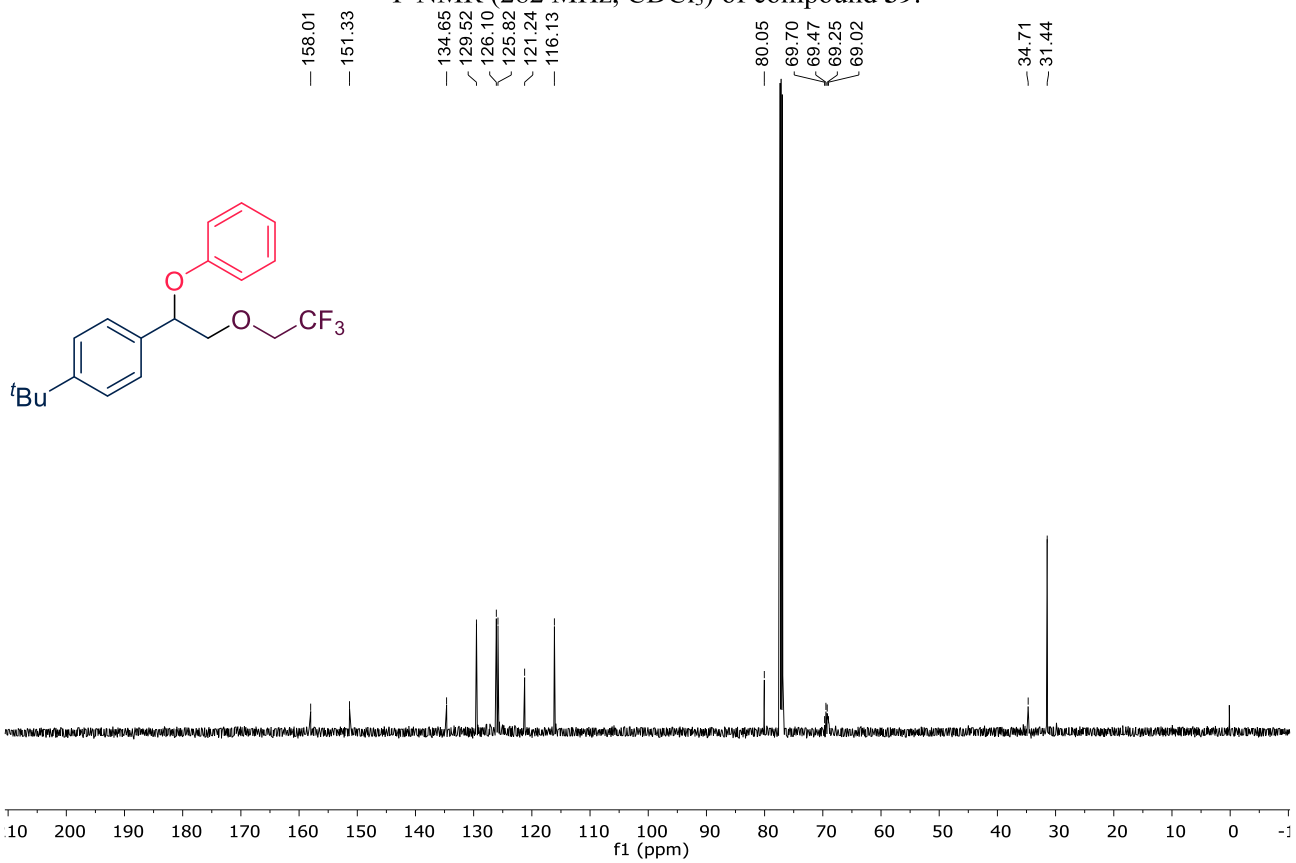

^{13}C NMR (151 MHz, CDCl$_3$) of compound **39**.

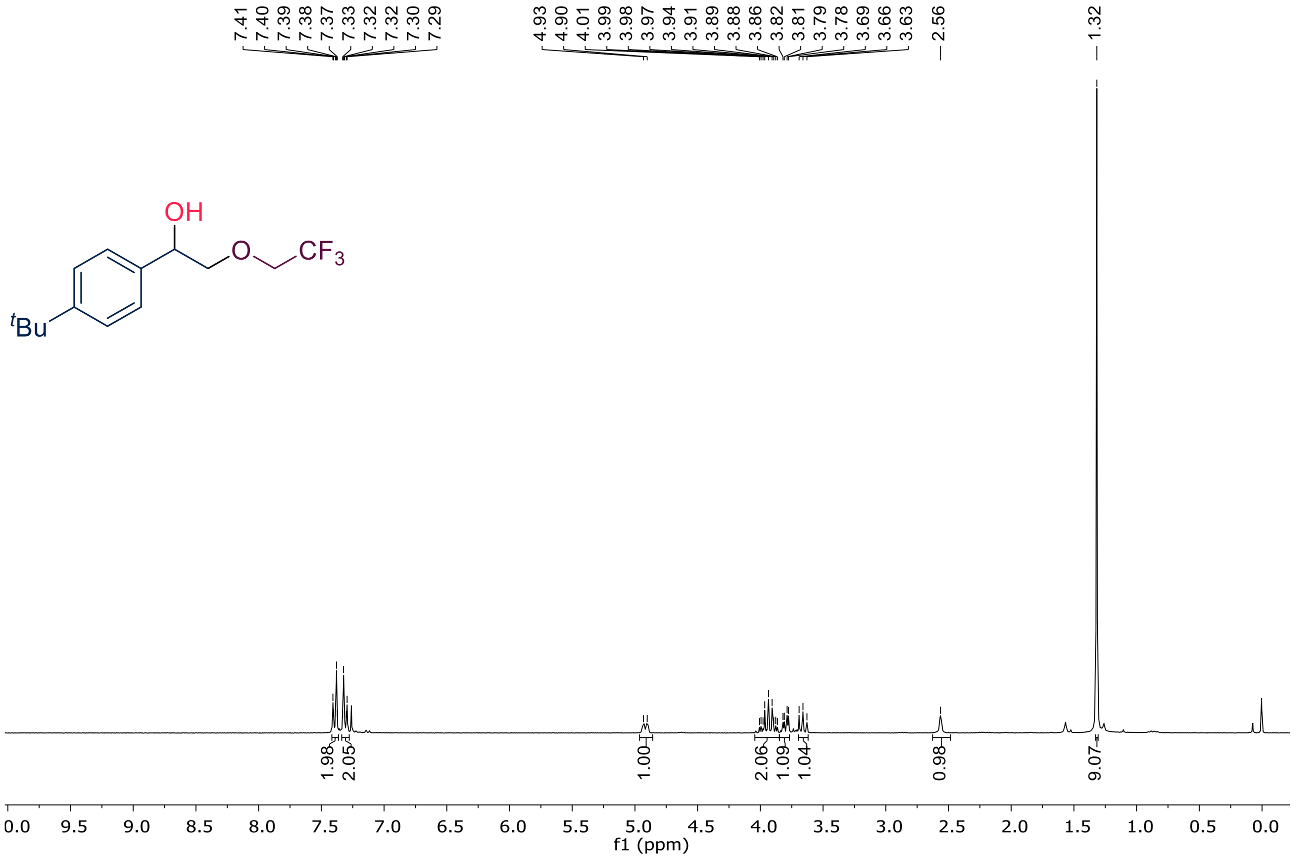

^{1}H NMR (600 MHz, $CDCl_3$) of compound **40**.

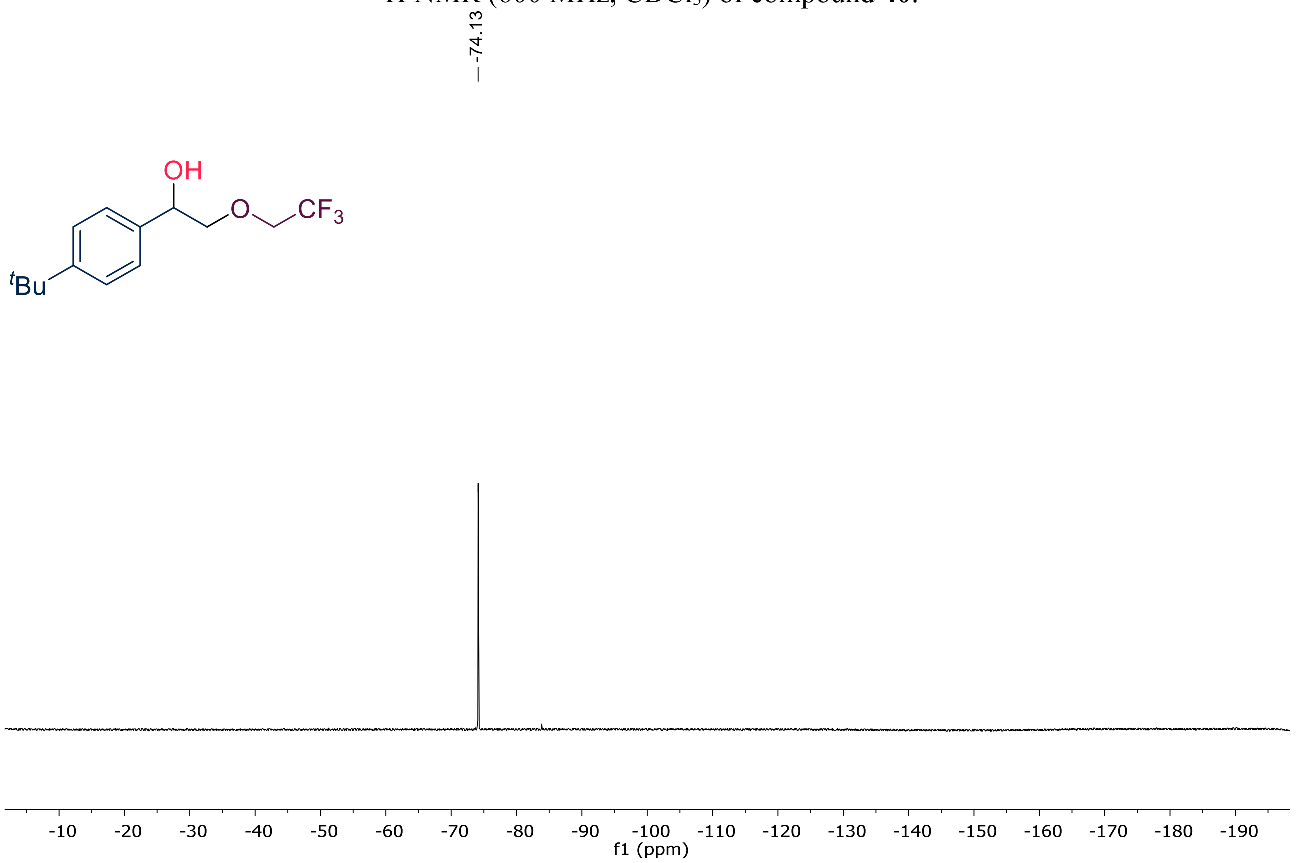

^{19}F NMR (282 MHz, $CDCl_3$) of compound **40**.

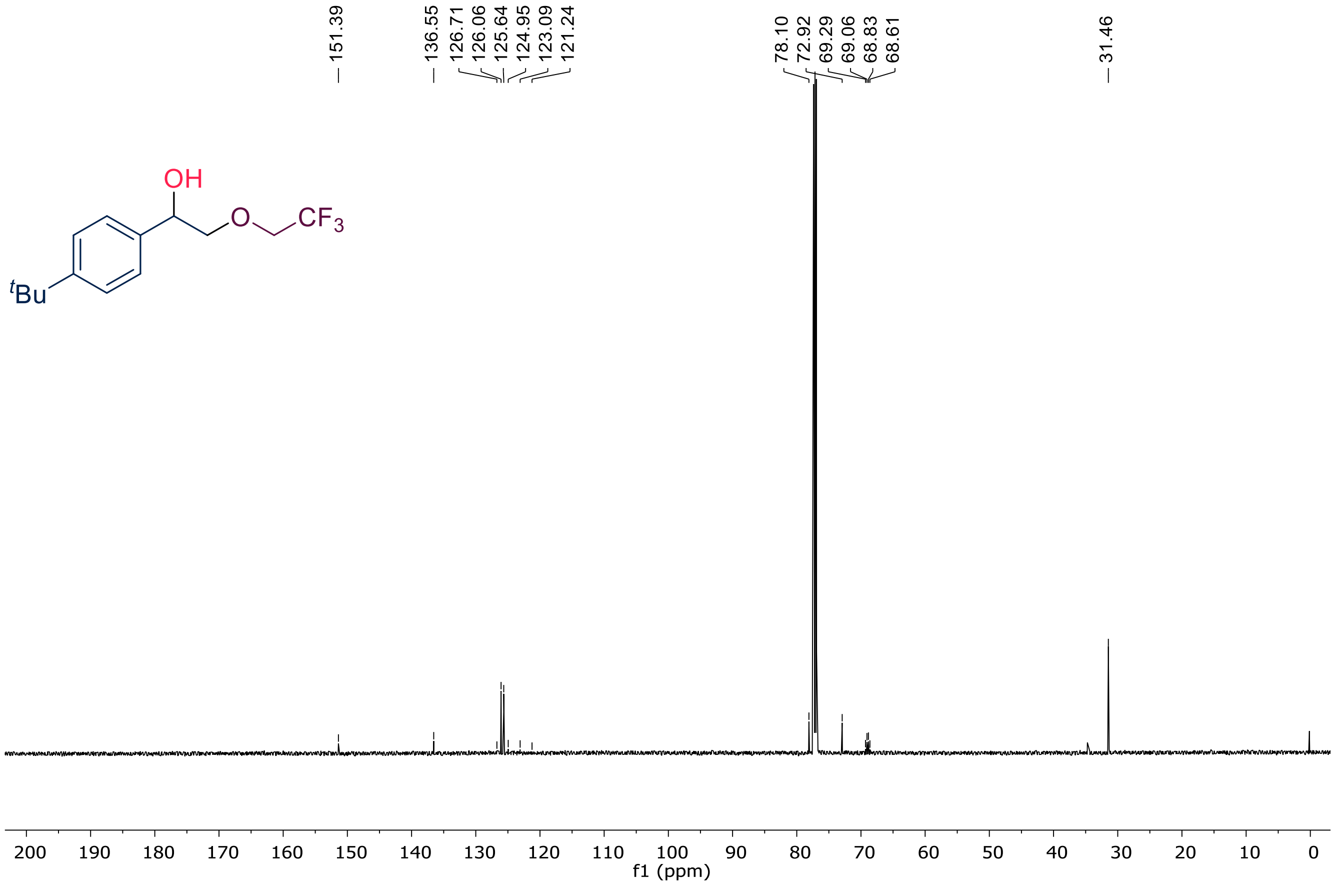

^{13}C NMR (151 MHz, $CDCl_3$) of compound **40**.

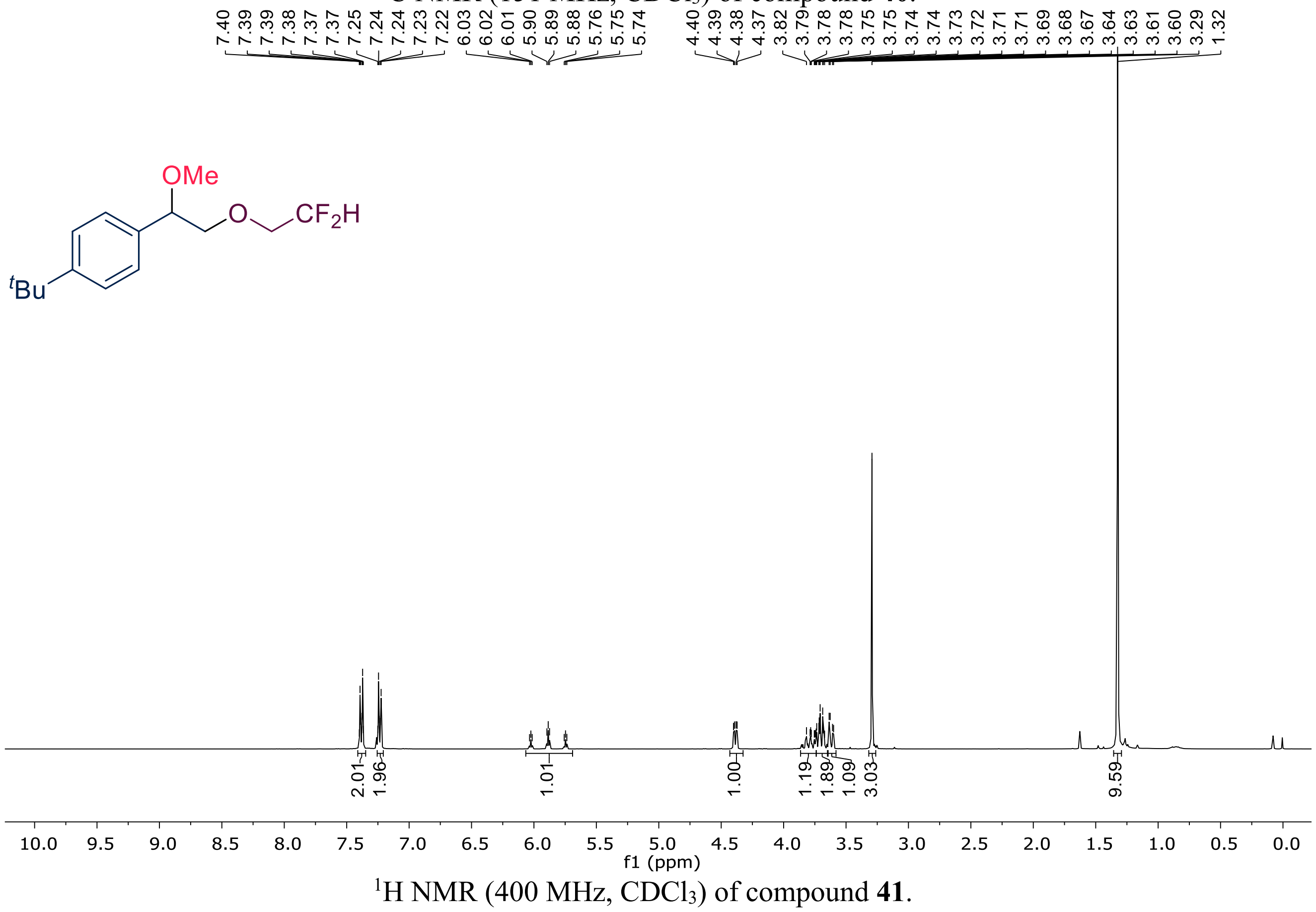

^{1}H NMR (400 MHz, $CDCl_3$) of compound **41**.

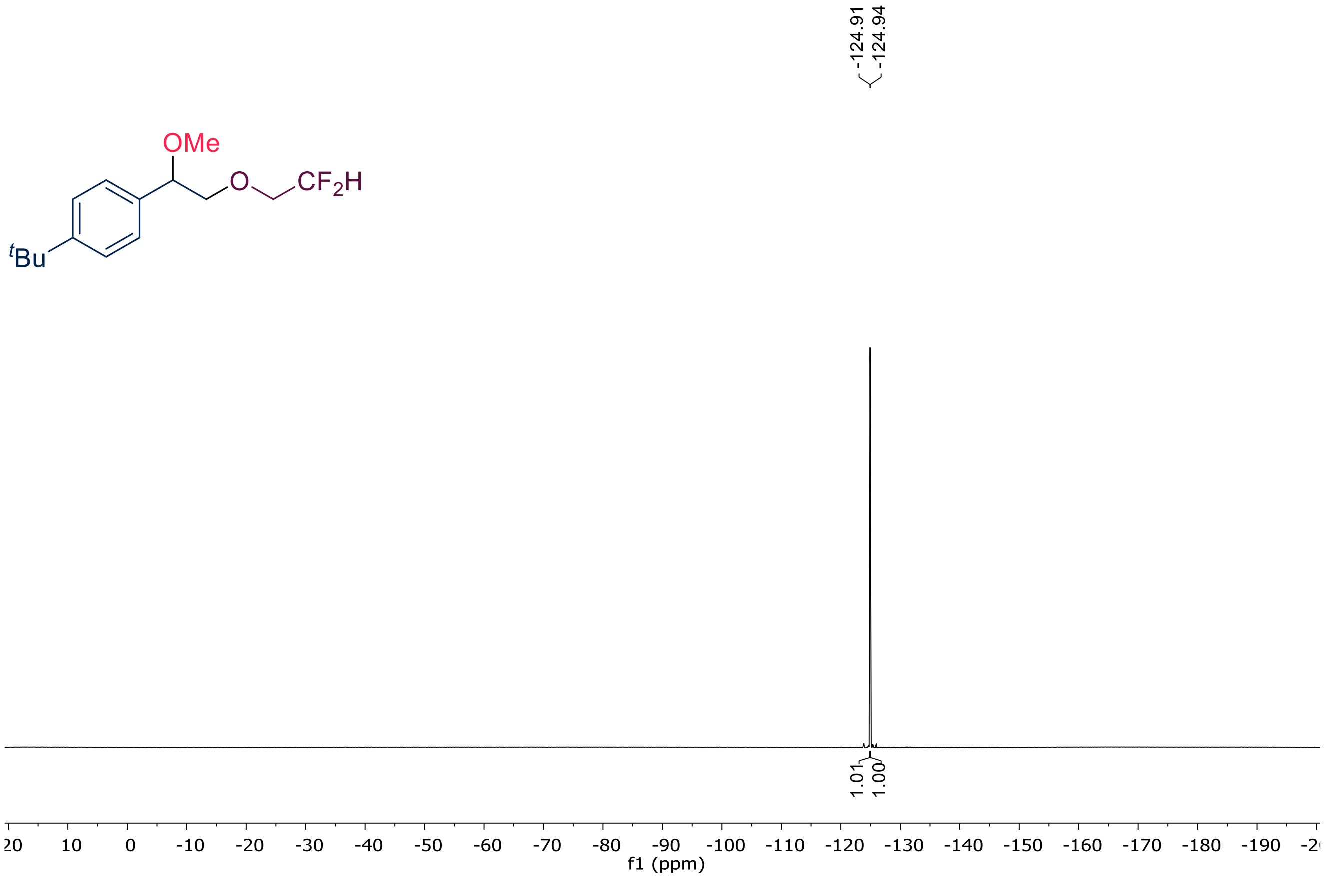

^{19}F NMR (282 MHz, $CDCl_3$) of compound **41**.

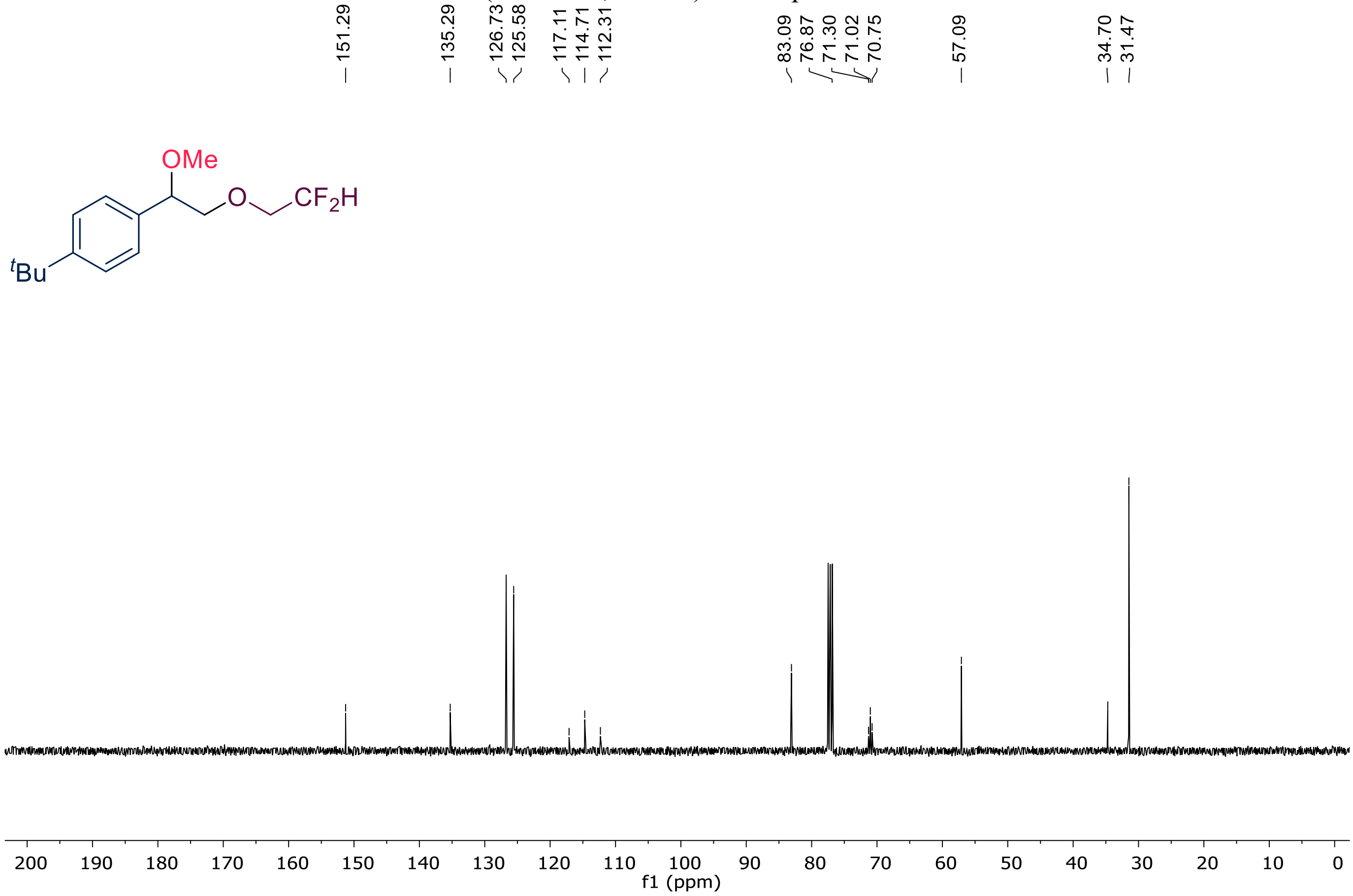

^{13}C NMR (101 MHz, $CDCl_3$) of compound **41**.

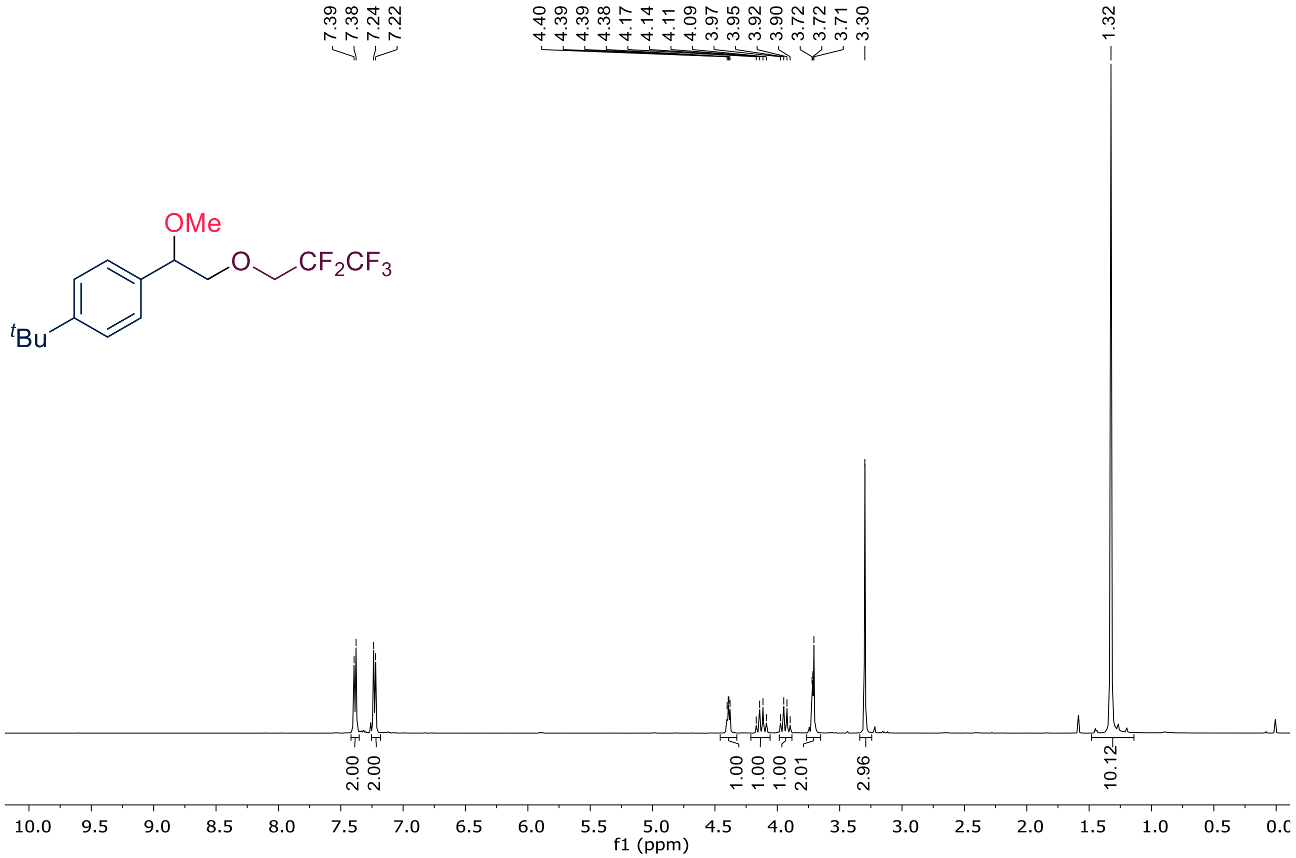

^{1}H NMR (500 MHz, $CDCl_3$) of compound **42**.

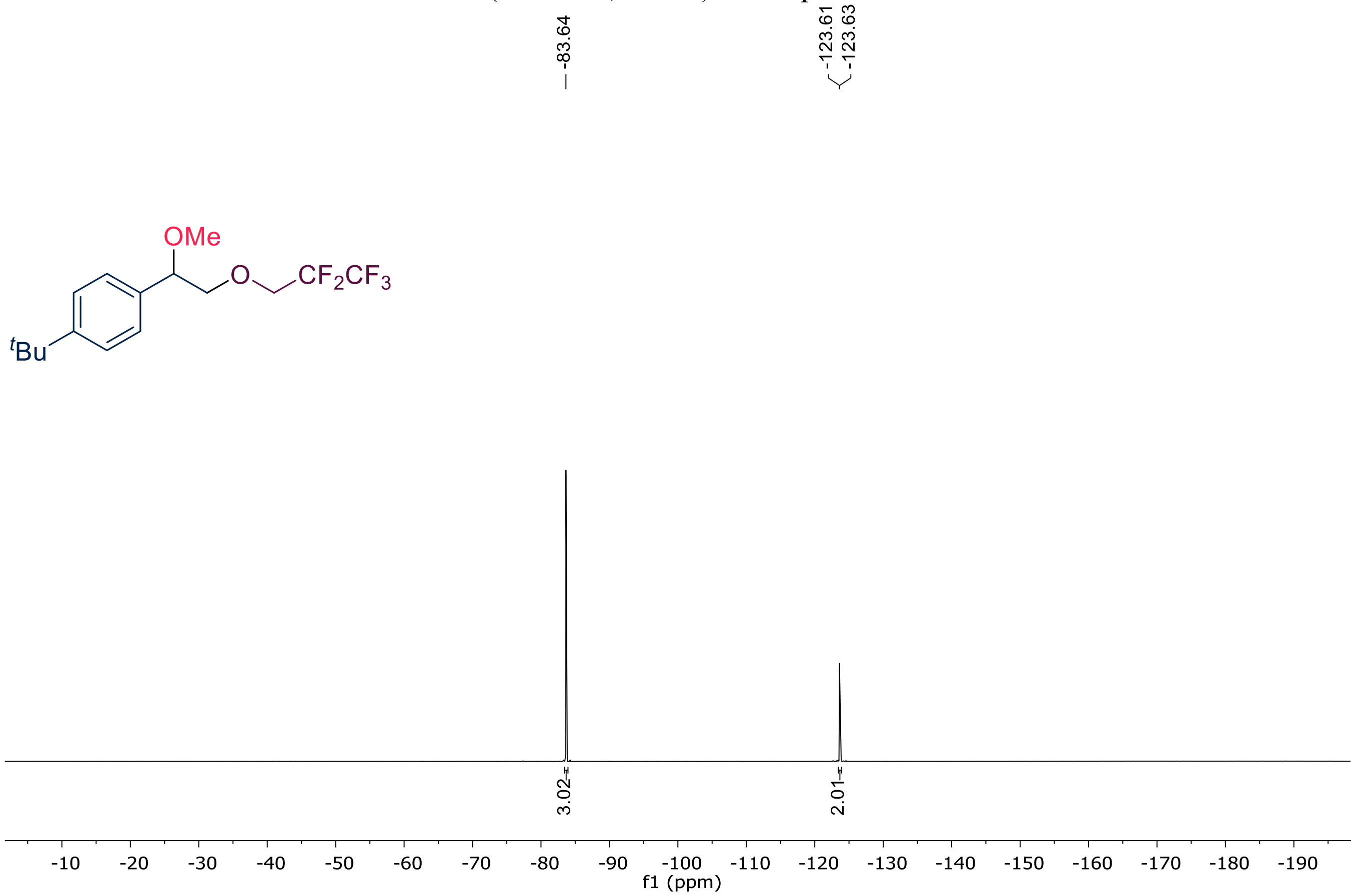

^{19}F NMR (282 MHz, $CDCl_3$) of compound **42**.

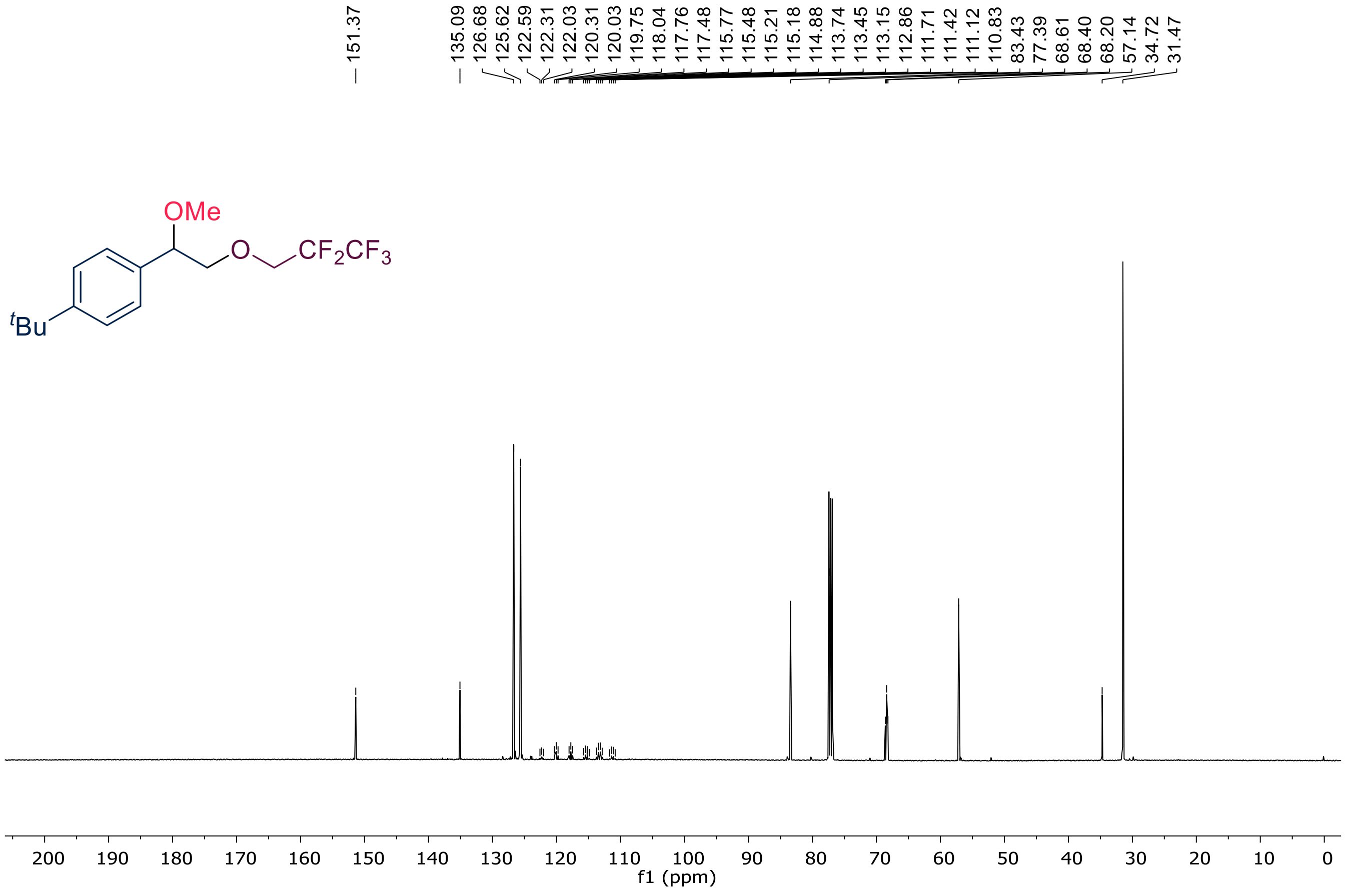

^{13}C NMR (126 MHz, $CDCl_3$) of compound **42**.

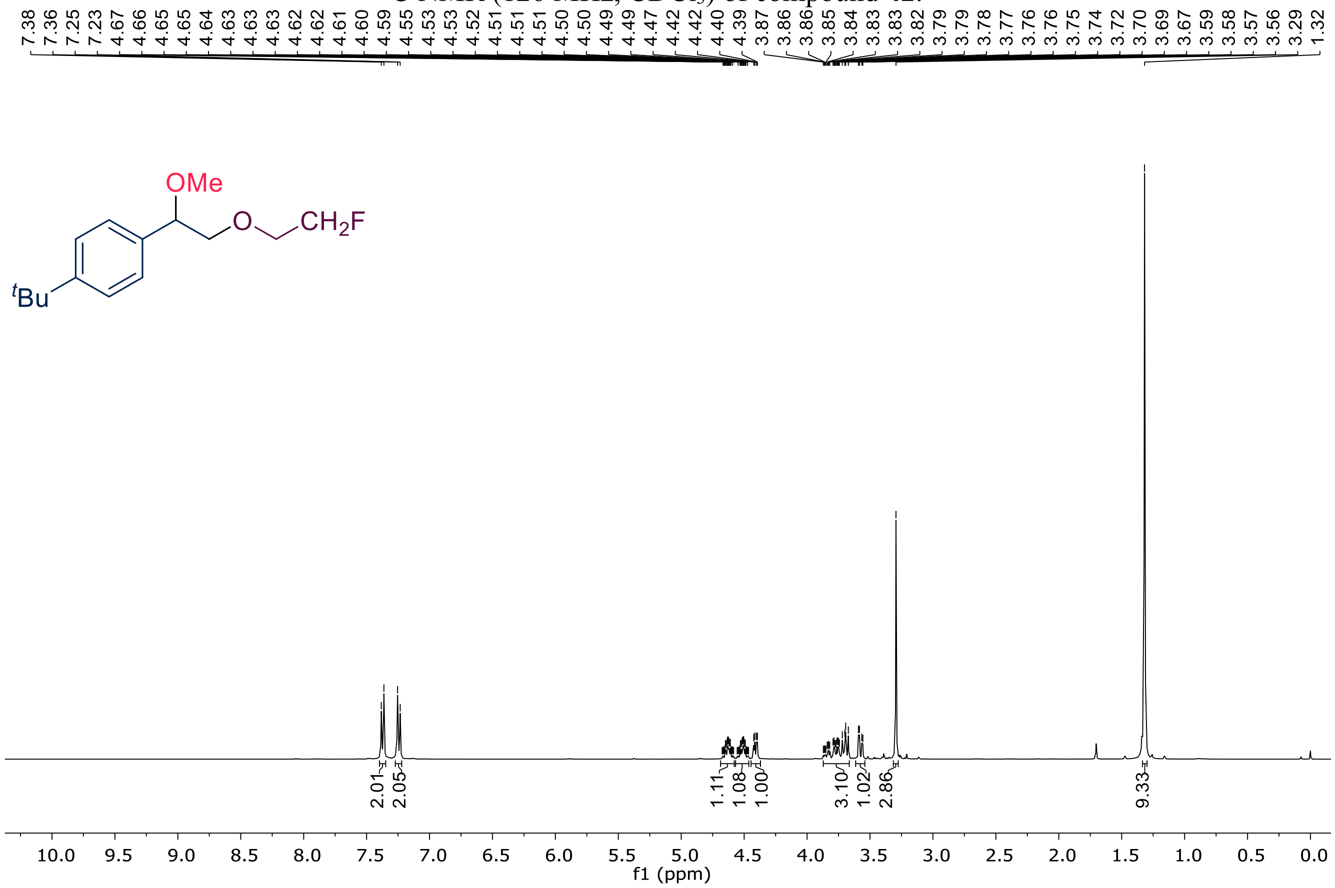

^{1}H NMR (400 MHz, $CDCl_3$) of compound **43**.

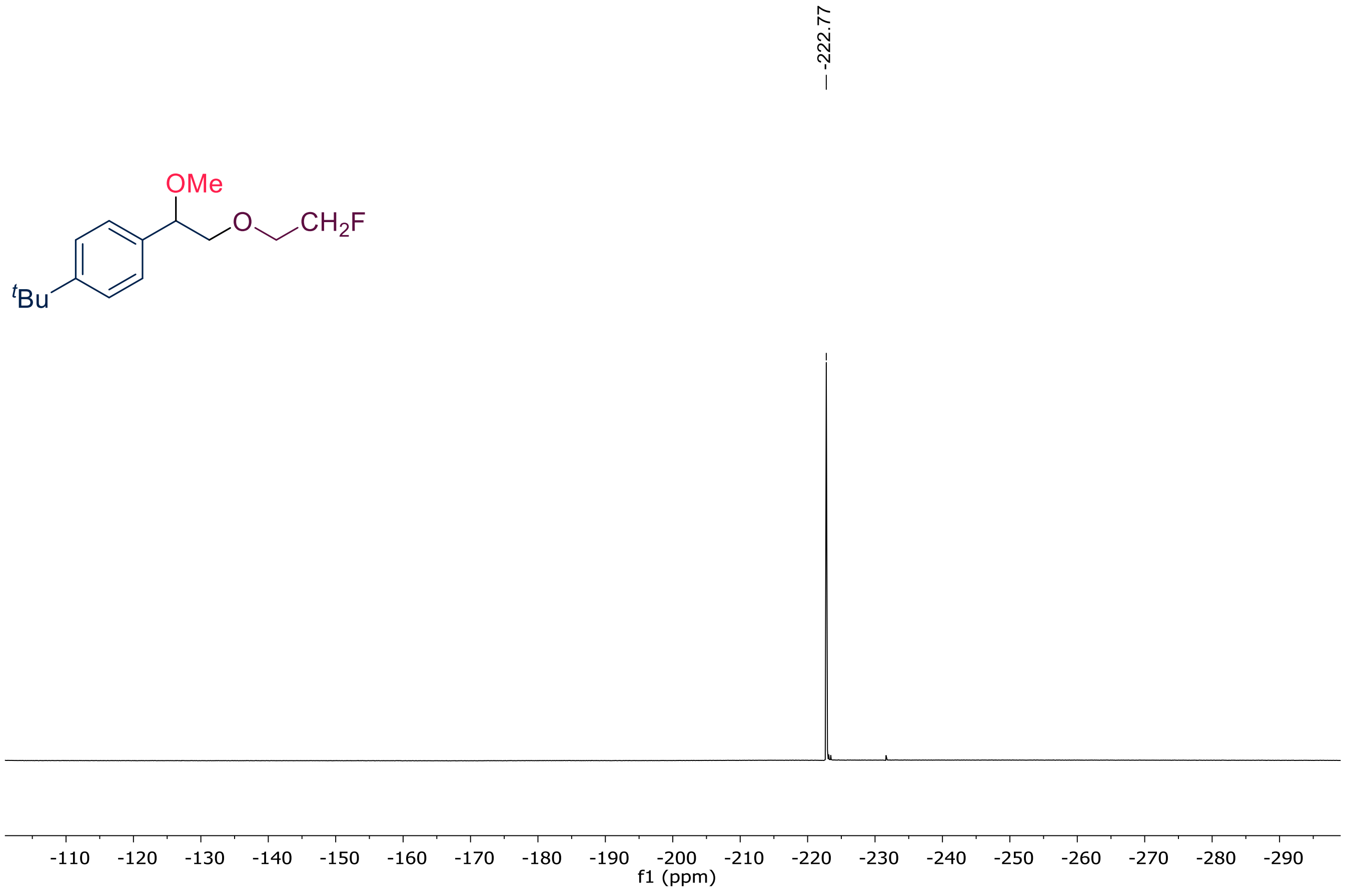

^{19}F NMR (377 MHz, $CDCl_3$) of compound **43**.

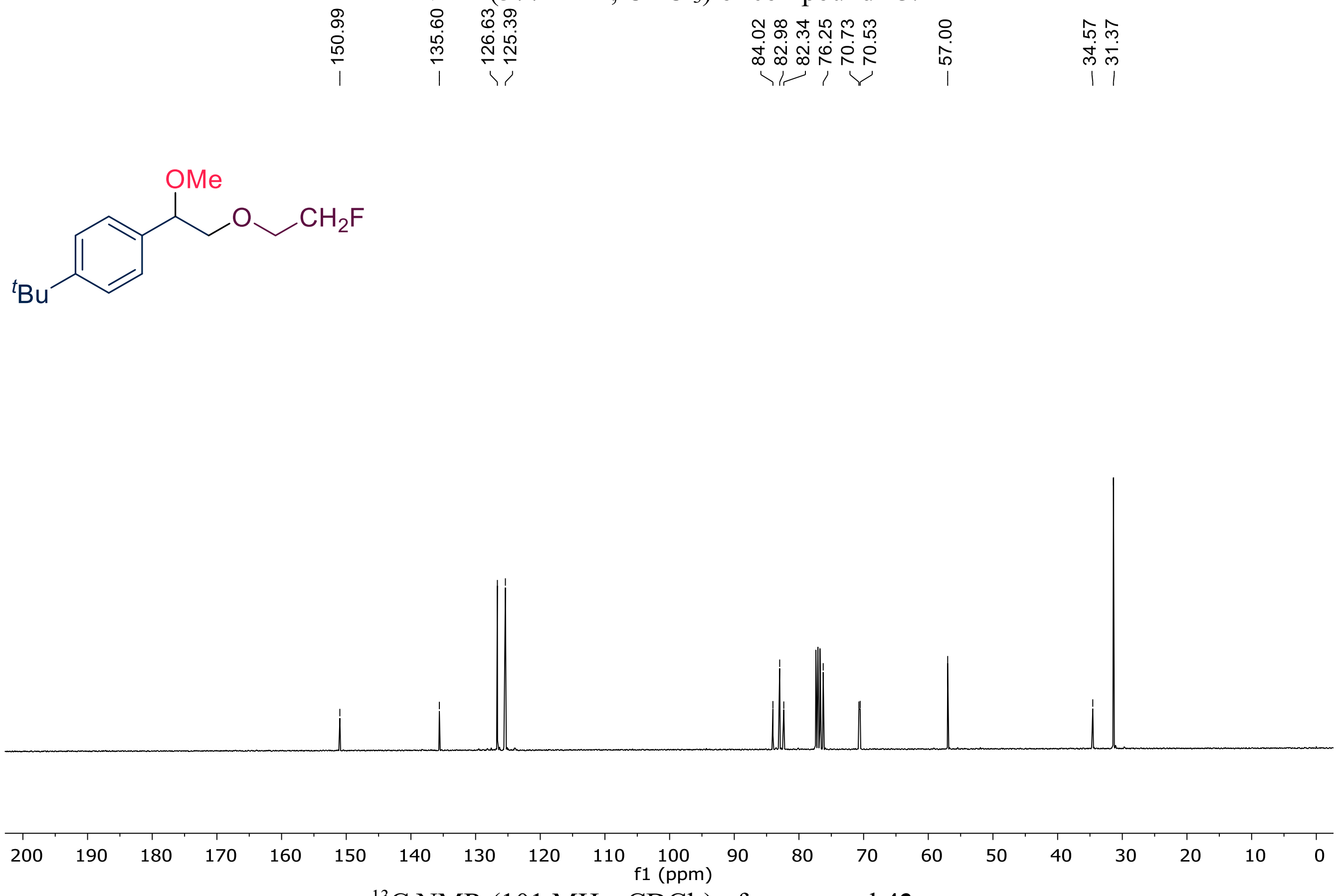

^{13}C NMR (101 MHz, $CDCl_3$) of compound **43**.
